# A novel repeat sequence-based PCR (rep-PCR) using specific repeat sequences of *Mycobacterium intracellulare* as a DNA fingerprinting

**DOI:** 10.3389/fmicb.2023.1161194

**Published:** 2023-04-06

**Authors:** Jeong-Ih Shin, Jong-Hun Ha, Kyu-Min Kim, Jeong-Gyu Choi, Seo-Rin Park, Hyun-Eui Park, Jin-Sik Park, Jung-Hyun Byun, Myunghwan Jung, Seung-Chul Baik, Woo-Kon Lee, Hyung-Lyun Kang, Jung-Wan Yoo, Min-Kyoung Shin

**Affiliations:** ^1^Department of Microbiology and Convergence Medical Sciences, Institute of Health Sciences, College of Medicine, Gyeongsang National University, Jinju, Republic of Korea; ^2^Fastidious Specialized Pathogen Resources Bank, A Member of the National Culture Collection for Pathogens, Gyeongsang National University Hospital, Jinju, Republic of Korea; ^3^Department of Laboratory Medicine, Gyeongsang National University Hospital, Jinju, Republic of Korea; ^4^Department of Internal Medicine, Gyeongsang National University Hospital, Jinju, Republic of Korea

**Keywords:** *Mycobacterium intracellulre*, nontuberculous mycobacteria, repetitive sequences based-PCR (rep-PCR), mycobacterial pulmonary disease, epidemiology, diagnostics, mycobacterium strain typing, genotype fingerprinting

## Abstract

Repetitive sequence-based PCR (rep-PCR) is a potential epidemiological technique that can provide high-throughput genotype fingerprints of heterogeneous *Mycobacterium* strains rapidly. Previously published rep-PCR primers, which are based on nucleotide sequences of Gram-negative bacteria may have low specificity for mycobacteria. Moreover, it was difficult to ensure the continuity of the study after the commercial rep-PCR kit was discontinued. Here, we designed a novel rep-PCR for *Mycobacterium intracellulare*, a major cause of nontuberculous mycobacterial pulmonary disease with frequent recurrence. We screened the 7,645 repeat sequences for 200 fragments from the genome of *M. intracellulare* ATCC 13950 *in silico*, finally generating five primers with more than 90% identity for a total of 226 loci in the genome. The five primers could make different band patterns depending on the genome of three different *M. intracellulare* strains using an *in silico* test. The novel rep-PCR with the five primers was conducted using 34 bacterial samples of 7 species containing 25 *M. intracellulare* clinical isolates, compared with previous published rep-PCRs. This shows distinguished patterns depending on species and blotting assay for 6 species implied the sequence specificity of the five primers. The Designed rep-PCR had a 95–98% of similarity value in the reproducibility test and showed 7 groups of fingerprints in *M. intracellulare* strains. Designed rep-PCR had a correlation value of 0.814 with VNTR, reference epidemiological method. This study provides a promising genotype fingerprinting method for tracing the recurrence of heterogeneous *M. intracellulare*.

## 1. Introduction

*Mycobacterium avium* complex (MAC) is a group of nontuberculous mycobacteria (NTM) responsible for MAC-pulmonary disease (MAC-PD), which has an increasing prevalence worldwide, especially in North America and East Asia (Prevots and Marras, 2015; Koh et al., 2017; Daley and Winthrop, 2020). MAC includes *M. avium, Mycobacterium intracellulare*, *M. arosiense, M. bouchedurhonense, M. chimaera, M. colombiense, M. marseillense, M. timonense, M. vulneris, and M. yongonense* (Parker et al., 2020; Keen et al., 2021). Among them, *M. intracellulare* has been reported to be responsible for the largest proportion of MAC-PD cases (Prevots and Marras, 2015; Chang et al., 2020; Ratnatunga et al., 2020). *Mycobacterium intracellulare* can be found in any environment, including soil, water, and air, and molecular epidemiology has revealed considerable genetic variation in its strains (Hruska and Kaevska, 2012; Parker et al., 2020; Lari and Rindi, 2022). MAC reinfection and redevelopment of MAC-PD can result from exposure to these environmental sources or regrowth of previously infected strains. The rate of the redevelopment of MAC-PD has been reported as approximately 10–48% and reinfection should be considered before deciding whether to proceed with the treatment (Kwon et al., 2019; Furuuchi et al., 2020; Kim et al., 2021).

MAC has been epidemiologically analyzed using whole genome sequencing (WGS), pulsed-field gel electrophoresis (PFGE), variable number tandem repeats (VNTR), repetitive sequence-based PCR (rep-PCR), and other molecular techniques. WGS offers comprehensive data including information of single nucleotide polymorphism (SNP) that can be used to identify allelic variation and distinguish strains. This WGS and interpretation of sequencing data requires a specialized technique for data analysis (Hatherell et al., 2016; Anyansi et al., 2020; Furuuchi et al., 2022). PFGE uses a whole-genome restriction enzyme site analysis approach that is labor-intensive and time-consuming. VNTR provides differentiated profiles of strains based on the number of copies of tandem repeats present at a specific locus in the genome, which requires at least 16 PCR reactions per *M. intracellulare* strain (Jagielski et al., 2016; Shin et al., 2020a,b). Rep-PCR is a fingerprinting method for comparing the electrophoretic patterns of PCR products of scattered repetitive sequences throughout the genome. It can reflect the site variation of the repetitive sequences in the bacterial genome, and there is economical merit that it can be conducted with a single PCR per strain (Warren et al., 2011; Rampadarath et al., 2015; Jagielski et al., 2016; Shin et al., 2020a,b). The repetitive sequences for rep-PCR are typical ‘BOX’ derived from *Streptococcus pneumoniae*, ‘Enterobacterial intergenic consensus (ERIC)’, and ‘Repetitive extragenic palindromes (REP)’ derived from *Escherichia coli* and *Salmonella typhimurium*. Since of their palindromic nature and capacity to form stable stem-loop structures in transcribed RNA, REP elements were first proposed as potential regulatory sequences within untranslated regions of operons. In addition, there is a similar PCR technique that detects a trinucleotide repeat sequence (TRS) that is repeated within the genome. At first, rep-PCR was used for species identification of Gram-negative intestinal bacteria and intraspecies distinction of *S. pneumoniae* (Versalovic et al., 1991; Koeuth et al., 1995; Healy et al., 2005). Rep-PCR also has been used for the classification of *M. avium*, *E. coli*, and *S. pneumoniae*, and TRS-PCR has been used to analyze MAC (Versalovic et al., 1995; Otsuka et al., 2004; Wojtasik et al., 2012). In 2003, a rep-PCR-based commercial kit was released for mycobacteria strain typing. The strain distinction in *M. avium* carried out using the kit was highly consistent with RFLP; however, the profiles of *M. intracellulare* could not be evaluated (Cangelosi et al., 2004).

To develop a new strategy for molecular fingerprinting method of *M. intracellulare*, this study seeks to explore repetitive sequences within the genome of *M. intracellulare* and develop *M. intracellulare*-based rep-PCR. We focused on the concepts from a previous study on *M. bovis*, in which repetitive sequences were searched through hybridization between genomic DNA libraries and chromosome digests, and subsequent sequencing of the identified DNA (Doran et al., 1993). As the complete genomic sequence of *M. intracellulare* ATCC 13950 is available at NCBI (Iakhiaeva et al., 2013), the The DNA library method can be modified *in silico* in this study. To evaluate this developed method, this study uses bacterial samples including *M. intracellulare* strains that had already been analyzed in their molecular epidemiologic profiles (PFGE and VNTR) in reference study (18). This evaluation can discover a correlation between novel rep-PCR and another molecular epidemiological method.

In conclusion, we hope to confirm whether the novel rep-PCR can be a potential molecular tool for disease epidemiology of *M. intracellulare* using repetitive sequences originating from the whole genome of *M. intracellulare* ATCC 13950.

## 2. Materials and methods

### 2.1. Bacterial samples

*Mycobacterium intracellulare* clinical isolates used in this study (n = 25) were originated from patients (*n* = 23) with pulmonary disease. Among the collection, there are isolates (n = 2) originated from a patient A on the same day (*M. intracellulare* KBN12P06800 and *M. intracellulare* KBN12P06801) and isolate (*n* = 2) from a patient B one week apart (*M. intracellulare* KBN12P06798 and *M. intracellulare* KBN12P06799). Patient labels were arbitrarily marked with letters A and B*. M. intracellulare* clinical isolates were collected between 2016 and 2017 at Gyeongsang National University Hospital (GNUH), Jinju, Korea and provided from the Fastidious Specialized Pathogen Resources Bank (a member of the National Culture Collection for Pathogens, GNUH), Jinju, Korea. For experimental control, two *M. intracellulare* reference strains (Asan 37128^T^ and ATCC 13950^T^), two *M. avium* reference strains (MAH 101^T^ and MAH 104^T^), *M. abscessus* KCTC 19621, *M. fortuitum* KBN12P06244, *E. coli* O157 NCCP 14541, *S. aureus* NCCP 14780, and *K. pneumoniae* NCCP 14764 were used in this study. Two *M. intracellulare* reference strains and *M. abscessus* KCTC 19621 were provided from the Korean Collection for Type Cultures (KCTC, Jeongeup, Korea). *M. fortuitum* KBN12P06244, *E. coli* O157 NCCP 14541, *S. aureus* NCCP 14780, and *K. pneumoniae* NCCP 14764 were provided from Fastidious Specialized Pathogen Resources Bank, Jinju, Korea. ^T^: Type strain.

### 2.2. *In silico* analysis for the development of rep-PCR using specific repeat sequences of *Mycobacterium intracellulare*

#### 2.2.1. Investigation of repetitive sequences and design of primers for rep-PCR

**Figure 1:**
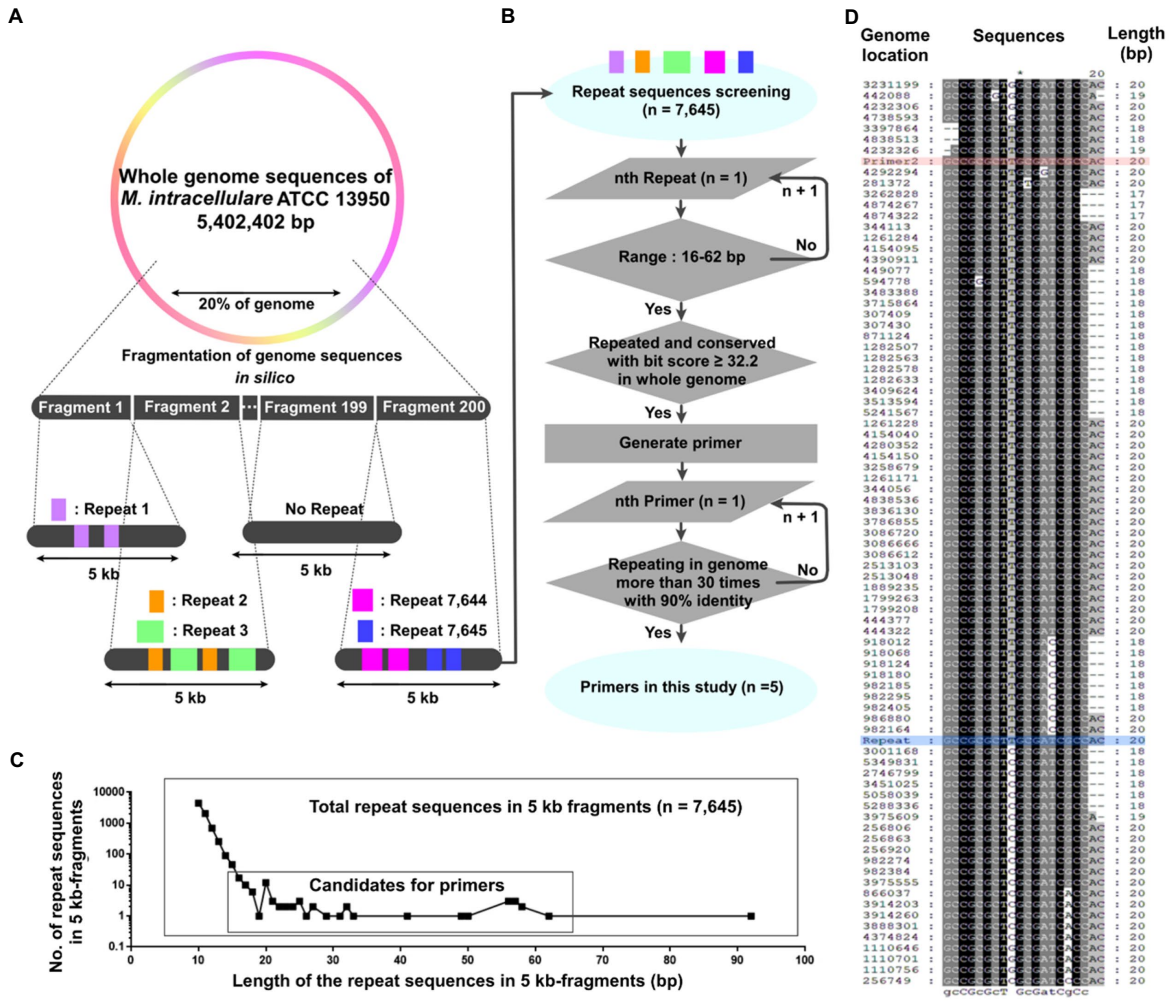
Generation of repetitive sequence-based primers across the whole genome using short 5 kb fragments. **(A**, **B)** A schematic diagram for generating repetitive primers within a genome. **(A)** 20% of the *Mycobacterium intracellulare* ATCC 13950 genome was cut into 200 fragments (5 kb-scaled). The repetitive sequences in each 5 kb fragment were screened, yielding 7,645 repetitive sequences. **(B)** The 7,645 repetitive sequences were screened based on their length and the number of repeats in the genome. Primers were generated using the screened sequences, and the number of repeats and identities in the genome of the primer was evaluated. **(C)** The number of the repetitive sequences according to their length and repeats within the 5 kb fragments and the range for primer candidates. Up to 19 bp, the number of repetitive sequences decreased in proportion to log10, but from 20 bp onwards, the number increased or stayed the same regardless of this trend. We set the range of primer screening at 16 to 62 bp. **(D)** Alignment (5′ to 3′) of Primer 2 with the *M. intracellulare* ATCC 13950 genome. Genome location: the genome position annealing by primers in NCBI database. Red: Primer 2, Blue: Repeat (it means repeated sequences within each 5 kb fragment).

This study mimicked the method of DNA library in the reference study using 200 *E. coli* recombinants and containing approximately 3–8 kb insert DNA sequences (28, Supplementary Table S1). Experimental design has an assumption that among short fragmented genomic DNA, there are repetitive sequences of the whole genome. Two hundred recombinants of reference study (28) were modified into 200 FASTA files and 3-8 kb insert DNA sequences were modified into 5 kb-scaled fragments. A total of 200 FASTA files were generated by dividing 20% of the genome (region from 1 to 965,101) of *M. intracellulare* ATCC 13950^1^ by non-overlapping 5 kb. Subsequently, the Repeats sequences finder^2^ was used to search for repetitive sequences greater than 10 bp within each 5 kb-scale FASTA file (Supplementary Table S2). Using rationale of sequence-length (Figure 1C and Supplementary Table S3), candidate sequences were chosen and BLAST^3^ was used to confirm whether these candidates aligned with the *M. intracellulare* ATCC 13950 genome. The alignment result was evaluated using BLAST bit score. If 16 bp matches between candidate sequences and genome without a gap, the score on BLAST was 32.2 bits. Therefore, each candidate sequence was evaluated using a frequency with a bit score of 32.2 or higher (Supplementary Table S3). Using ClustalX (version 1.8) and EditSeq (version 7.0.0), we found conserved sequences among the region with bit score ≥ 32.2 and designed a primer based on it. %Identity and matching frequency of primer candidates in the *M. intracellulare* ATCC 13950 genome were confirmed. Through this process, new primers for designed rep-PCR were generated.

#### 2.2.2. Demonstration the designed rep-PCR using *In silico* PCR

*In silico* PCR amplification^4^ was used to predict the PCR products amplified from genomic DNA (San Millán et al., 2013) in 45 strains, including three *M. intracellulare* strains (ATCC 13950, MOTT-02, and MOTT-64), as well as strains of *M. africanum*, *M. avium*, *M. avium* subsp. *paratuberculosis*, *M. bovis*, *M. canetti*, *M. indicus*, *M. tuberculosis*, and *M. yongonense*. The criteria were set to allow 2 mismatches between primers and template and a maximum amplicon length of 10,000 nucleotides. To investigate whether there were variations in band patterns among the three *M. intracellulare* strains, we compared the band lengths of the expected *in silico* PCR products for the five designed primers (Table 1).

**Table 1:** The list of the primers used in this study.

No.	Primer	Primer sequences (5′ -3′)	Sequences origin	Reference
1	Primer 1	GATGGTGGCGACCCGCTGCG (20 bp)	*M. intracellulare* ATCC 13950	*this study*
2	Primer 2	GCCGCGCTTGCGATCGCCAC (20 bp)		*this study*
3	Primer 3	CGACGATGCAGAGCGAAGCGATG (23 bp)		*this study*
4	Primer 4	CGCCGCGCTCGCGATCGCCACT (22 bp)		*this study*
5	Primer 5	GGCGACCCGCTTCGCCCGGCTCCG (24 bp)		*this study*
6	BOX A1R	CTACGGCAAGGCGACGCTGACG (22 mer)	*Streptococcus pneumoniae*	(Rampadarath et al., 2015)
7	ERIC 1R	ATGTAAGCTCCTGGGGATTCAC (22 mer)	*E. coli* and *Salmonella typhimurium*	(Warren et al., 2011)
8	ERIC 2	AAGTAAGTGACTGGGGTGAGCG (22 mer)		(Warren et al., 2011)
9	REP 1RI	IIIICGICGICATCIGGC (11 mer)		(Warren et al., 2011)
10	REP 2I	ICGICTTATCIGGCCTAC (15 mer)		(Warren et al., 2011)
11	(CCG)_4_	NNNNNNCCGCCGCCGCCG (18 mer)	*M. avium*	(Healy et al., 2005)

### 2.3. Conduction of novel rep-PCR for *Mycobacterium intracellulare*

#### 2.3.1. Experimental condition of the designed rep-PCR

Including the *M. intracellulare* clinical isolates, all bacterial samples mentioned above were used. Genomic DNA was extracted using an AccuPrep Genomic DNA Extraction Kit (Bioneer, Korea). The DNA template (120 ng per tube) and five designed primers (10 pmol/primer) per tube were simultaneously added, and the designed rep-PCR reaction was conducted as follows: initial denaturation at 95°C for 5 min; 35 cycles of 95°C for 30 s, 65°C for 1 min, and 72°C for 2 min; and a final extension at 72°C for 7 min.

#### 2.3.2. Experimental conditions of the previous published rep-PCRs

The genomic DNA extracted above was used as a DNA template (120 ng per tube). List of primer are BOX A1R, ERIC 1R, ERIC 2, REP 1RI, REP 2I, and (CCG)_4_ (n = 6). Set of primers were used in the PCR experiments, single primer (BOX A1R or CCG_4_), in pairs (ERIC 1R + ERIC 2), or in combination (BOX A1R + ERIC 1R + ERIC 2, BOX A1R + REP 1RI + REP2I, and BOX A1R + ERIC 1R + ERIC 2 + REP 1RI + REP 2I) (Table 1). Previous published rep-PCR reaction was conducted as follows: initial denaturation at 95°C for 5 min; 30 cycles of 95°C for 30 s, 40°C for 1 min, and 72°C for 2 min; and a final extension at 72°C for 7 min (Chen et al., 2021; Jena et al., 2021; La China et al., 2021; Teixeira et al., 2021).

### 2.4. Validation of designed rep-PCR for *Mycobacterium intracellulare*

#### 2.4.1. Reproducibility test

Reproducibility of the designed rep-PCR was confirmed using cultured strains of different periods of *M. intracellulare*. Four *M. intracellulare* strains (KBN12P07945, KBN12P06800, KBN12P6801, KBN12P07346) were selected randomly for the reproducibility test. DNA templates of four strains were extracted before and after subculturing, respectively. Using the extracted DNA template, the designed-PCR and the previously published rep-PCRs were performed under the conditions described in 2.3.1 and 2.3.2, respectively. We yielded %similarity (reproducibility) by comparing the band patterns before and after the subculture in each strain. The band patterns were analyzed under the conditions of Pearson coefficient, UPGMA clustering, and a tolerance value of 1.0, using the GelJ software (version 2.0), and a dendrogram was generated according to the values of the similarity calculated using the GelJ software (Arais et al., 2018; Heidari et al., 2018). In addition, the reproducibility of the designed-repPCR was confirmed using various species including *M. intracellulare*. Confirmation for % similarity between various species (Supplementary Figure S1), DNA templates were extracted before and after the subculture in each strain (Four *M. intracellulare* stains, two *M. avium* strains, one *M. fortuitum* strain, one *M. abscessus* strain, one *E. coli* strain, one *S. aureus* strain, and one *K. pneumoniae* strain). PCR such as the designed-PCR and the previously published rep-PCRs was performed under the conditions of the 2.3.1 and 2.3.2, respectively. The %similarity (reproducibility) of the band patterns were yielded under the conditions of Dice, UPGMA clustering, and a tolerance value of 1.0, using the GelJ software.

#### 2.4.2. Confirmation of nucleotide sequence specificity of designed primers using southern blot

The electrophoretic bands of the six species (*M. intracellulare* KBN12P06800, *E. coli*, *K. pneumoniae*, *M. abscessus*, *M. fortuitum*, and *M. avium* MAH 104) using the novel rep-PCR were transferred from agarose gel to the GeneScreen Plus membrane (NEN, NEF988). For instructions on transfer, we referred to the ECL Direct Labeling and Detection System (GE, RPN3000). Primers 1–5 were designed with biotin at the 5′ terminal (synthesized by Bioneer Inc., Korea) and hybridized with the membrane using hybridization and blocking buffers (GE, RPN 3000). Hybridization, washing, and detection were performed according to the instructions provided by the North2SouthChemilinescent Hybridization and Detection Kit (Thermos, 17,097). Hybridization and washing were performed at 50°C and 40°C, respectively, and streptavidin-HRP (Sigma, N100) was used for biotin detection of the probe. The absolute value of the Southern blot signal was measured in an image of the X-ray film using ImageJ (1.53e) (Gautam, 2022; Wang et al., 2022).

#### 2.4.3. Verification of correlation with reference method

The electrophoresis results were normalized according to the reference molecular weight lanes using GelJ software (version 2.0), and the normalized rep-PCR bands were grouped based on their Pearson %similarity. For the confirmation of the correlation between designed rep-PCR and other defined epidemiological methods, we referred to data from a previous study (Shin et al., 2020a,b) that conducted PFGE and VNTR on the almost same *M. intracellulare* clinical isolates as in this study. To calculate the correlation coefficient, Cramer’s V coefficient were calculated using IBM SPSS software (version 21) (Akoglu, 2018).

## 3. Results

### 3.1. Repetitive sequences within the genome can be identified using 5 kb-scaled genomic fragments

A total of 7,645 repetitive sequences were yielded within each 5 kb-fragment, which originated from the 20% genome of *M. intracellulare* ATCC 13950 (Figure 1A and Supplementary Table S2). Among them, there are 402 duplication sequences and 7,243 unique sequences. As the length of the repetitive sequences increased from 10 bp to 19 bp, the number of repetitive sequences decreased in proportion to log10. And the number of 21 bp to 92 bp repetitive sequences remained below four (Figure 1C). We selected 51 sequences ranging from 16 bp to 62 bp, which are considered to be highly conserved in the genome relative to their length (Supplementary Table S3). 51 sequences have 2 to 95 regions with a score ≥ 32.2 per candidate sequence and we generated primers using these regions (Figures 1B,D). As a result, a total of 5 primers were determined for the sequences repeated throughout the genome (Table 1). Primer 1 originates from 57 bp of the sequence encoding a hypothetical protein in *M. intracellulare* ATCC 13950. The sequence of Primer 2 is shared by *M. tuberculosis*, *M. kansasii*, *M. ostraviense*, *M. avium*, *M. malmoense*, *M. ulcerans*, and *M. shottsii*, and is identical to a 20 bp sequence found in the genes that encode a hypothetical protein, ABC transporter efflux protein, DrrB family protein, and lipoprotein LpqB in *M. intracellulare* ATCC 13950. Primer 3 is derived from a 50 bp sequence in the gene encoding the PbpA protein in *M. intracellulare* ATCC 13950, and primers 4 and 5 are derived from a 62 bp sequence in the gene encoding the rfbE protein in *M. intracellulare* ATCC 13950 (Supplementary Table S4). Each primer was aligned with 90% identity in both forward and reverse orientations, at more than 31 to 63 loci in the *M. intracellulare* ATCC 13950 genome. The locations of aligned loci in the genome were investigated (Supplementary Table S5). This means that PCR polymerization is possible due to forward and reverse pairs of primers located in 31 to 63 loci per sequence in the *M. intracellulare* ATCC 13950 genome.

### 3.2. *In silico* PCR with designed primers demonstrate genomic variation within The *Mycobacterium intracellulare* strains

For the three *M. intracellulare* strains (ATCC 13950, MOTT-02, and MOTT-64), The amplicons of similar sizes were integrated into single band (Table 2). In the *M. intracellulare* ATCC 13950, a total of five PCR bands of approximately 4.2, 4.7, 5.5, 6.7, and 8.7 kb-lengths were yielded from eight genome positions. Likewise, a total of five PCR bands of approximately 4.2, 5.5, 6.2, 6.7, and 8.5 kb-lengths were yielded in MOTT-02, and four bands of approximately 4.7, 5.5, 6.7, and 8.5 kb-lengths were yielded in MOTT-64. Therefore, the band patterns of the *in silico* PCR amplicons for the designed five primers between the three *M. intracellulare* strains were different from each other, indicating that the designed primers can distinguish the *M. intracellulare* strains from each other. In the same way as above, the lengths of virtual PCR amplicons were predicted in various mycobacteria species registered in NCBI (Supplementary Table S6). As a result, the *in silico* PCR amplicons showed different patterns between various mycobacterial species (*M. avium*, *M. bovis*, *M. canettii*, *M. tuberculosis*, *M. yongonense*, etc.) implying the sequence specificity of primers.

**Table 2:** Different *in silico* PCR bands between the *M. intracellulare* strains using designed primers.

*M. intracellulare* ATCC 13950			*M. intracellulare* MOTT-02			*M. intracellulare* MOTT-64		
Primer pair	Length of *in silico* amplicon*^a^*	*in silico* PCR band*^b^*	Primer pair	Length of *in silico* amplicon	*in silico* PCR band	Primer pair	Length of *in silico* amplicon	*in silico* PCR band
Primer 1 and 4	4180	Approx.^c^ 4.2kbp	Primer 1 and 2	4179	Approx. 4.2kbp	Primer 1 and 2	4663	Approx. 4.7kbp
Primer 1 and 2	4179		Primer 1 and 4	4180		Primer 2 and 3	4663	
Primer 1 and 2	4746	Approx. 4.7kbp	Primer 1 and 3	5490	Approx. 5.5kbp	Primer 2 and 5	4663	
Primer 2 and 3	4717		Primer 2 and 3	5490		Primer 2 and 4	4664	
Primer 2 and 4	4717		Primer 3 and 4	5490		Primer 1 and 3	5490	Approx. 5.5kbp
Primer 2 and 5	4717		Primer 3 and 5	5490		Primer 2 and 3	5490	
Primer 1 and 3	5490	Approx. 5.5kbp	Primer 2 and 3	6212	Approx. 6.2kbp	Primer 3 and 4	5490	
Primer 2 and 3	5490		Primer 2 and 4	6212		Primer 3 and 5	5490	
Primer 3 and 4	5490		Primer 2 and 5	6212		Primer 1 and 3	6730	Approx. 6.7kbp
Primer 3 and 5	5490		Primer 1 and 2	6241		Primer 2 and 3	6730	
Primer 1 and 3	6730	Approx. 6.7kbp	Primer 1 and 3	6730	Approx. 6.7kbp	Primer 3 and 4	6730	
Primer 2 and 3	6730		Primer 2 and 3	6730		Primer 3 and 5	6730	
Primer 3 and 4	6730		Primer 3 and 4	6730		Primer 2 and 3	8508	Approx. 8.5kbp
Primer 3 and 5	6730		Primer 3 and 5	6730		Primer 1 and 3	8538	
Primer 2 and 3	8677	Approx. 8.7kbp	Primer 1 and 3	8538	Approx. 8.5kbp	-	-	-
Primer 1 and 3	8651		Primer 2 and 3	8564		-	-	-

a Length of in silico amplicon: distance of sequences between pair of primer.

b in silico PCR band: Expected result of virtual PCR bands on agarose gel.

c Approx.: approximately.

### 3.3. The novel rep-PCR produce reproducible band patterns

**Figure 2:**
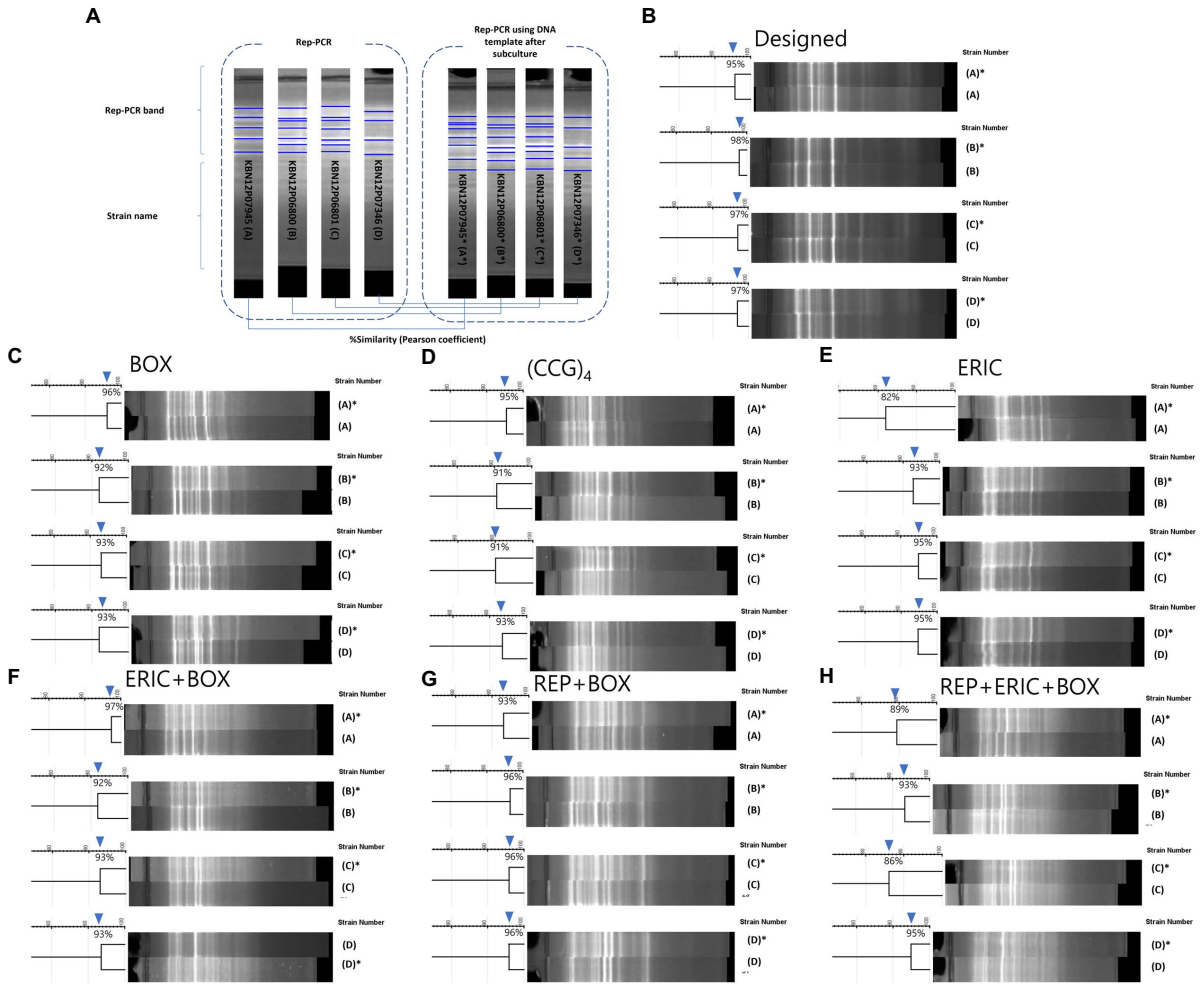
Comparison of reproducibility tests for designed and previous published rep-PCRs. **(A)** A diagram of the reproducibility test. PCR bands were compared from one *M. intracellulare* before (no asterisk) or after subculture (with an asterisk). 4 *M. intracellulare* strains were used to PCR and pair of PCR bands were analyzed by the similarity of the Pearson coefficient. **(B–H)** Results of the reproducibility test for Designed, BOX, (CCG)4, ERIC, ERIC+BOX, REP+BOX, REP+ERIC+BOX rep-PCR. **(B)** The similarities of Designed rep-PCR for same clone of *M. intracellulare* (with or without subculture) were 95–98%. **(C)** The similarities of BOX-PCR for same clone of *M. intracellulare* were 92–96%. **(D)** The similarities of (CCG)_4_-PCR for same clone of *M. intracellulare* were 91–95%. **(E)** The similarities of ERIC-PCR for same clone of *M. intracellulare* were 82–95%. **(F)** The similarities of ERIC+BOX-PCR for same clone of *M. intracellulare* were 92–97%. **(G)** The similarities of REP+BOX-PCR for same clone of *M. intracellulare* were 93–96%. **(H)** The similarities of REP+ERIC+BOX-PCR for same clone of *M. intracellulare* were 86–95%.

First, the purpose of this test was to present the reproducibility value (% value, similarity using Pearson coefficient) of this new rep-PCR method and to use it as a criterion for the genetic clone of *M. intracellulare* strains (Yamamura et al., 2004; Healy et al., 2005; Güvenir et al., 2018; Hwang et al., 2021). In the designed rep-PCR, the *M. intracellulare* samples (4 pairs) showed reproducibility with approximately 95–98% similarity (Figure 2). Therefore, in this study, strains that shares 95 to 98% similarity are defined as a genetic clone.

Second, it was conducted to compare the results of similarity between the newly developed rep-PCR and the results of the previous published rep-PCR (Cangelosi et al., 2004). In previous published rep-PCRs (BOX-, (CCG)_4_–, ERIC-, ERIC+BOX-, REP+BOX-, and REP+ERIC+BOX-PCR), the *M. intracellulare* samples (4 pairs) showed reproducibility with approximately 92–96%, 91–95%, 82–95%, 92–97%, 93–96%, 86–95%, respectively. This value showed slightly lower level than Designed rep-PCR. Furthermore, the background noise of previous published rep-PCRs increases as the number of primers increases. However, the designed rep-PCR using the five primers simultaneously showed a clear background and patterns (Figure 2).

The reproducibility test results including other species (*M. abscessus*, *M. fortuitum*, *M. avium*, *S. aureus, K. penumoniae* and *E. coli*) are shown in Supplementary Figure S1. In the designed rep-PCR, 27 *M. intracellulare* strains showed bands of various patterns with a similarity of 56–90%. The band patterns between *M. intracellulare* and the other six species could be distinguished by two clusters with 50% similarity. In previous published rep-PCR, however, *M. intracellulare* and other species were difficult to distinguish. This is because the similarity between *M. intracellulare* and the other species was same or high with similarity between *M. intracellulare* strains. This test shows the reproducibility and band patterns of designed rep-PCR for bacteria that can be contaminated with *M. intracellulare.* In addition, showing different patterns between species may suggest the sequence specificity of the designed primers.

### 3.4. The novel rep-PCR primers can produce different bands with sequence specificity

**Figure 3:**
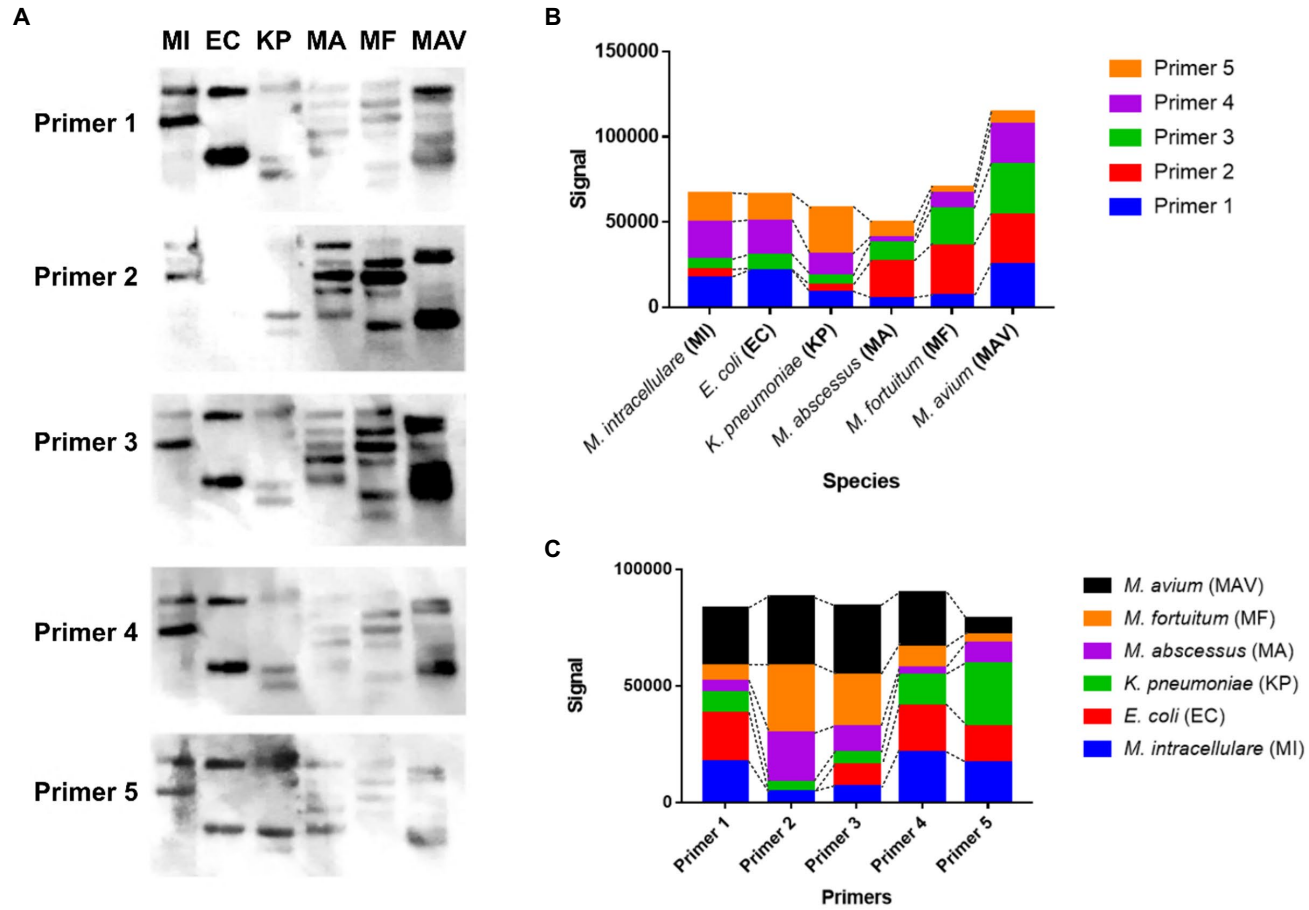
The hybridization pattern and the signal value between the designed rep-PCR product of the six species and each designed primer. **(A)** Southern blotting results of each designed primer (Primers 1 to 5) against the designed rep-PCR products of *M. intracellulare, E. coli, K. pneumoniae, M. abscessus, M. fortuitum*, and *M. avium*, denoted as MI, EC, KP, MA, MF, and MAV, respectively. **(B)** The hybridization signals of 5 primers for each species. The hybridization rate of each primer is different depending on the species. The hybridization ratio of *E. coli* is similar to that of *M. intracellulare*, but there is no hybridization with Primer 2. The total amount of signals for *M. avium* is the highest among the species with primer 2. **(C)** The hybridization signal of each species for each primer. Although the hybridization ratio is different, the total signals corresponding to one primer are similar.

To show the sequence specificity of the primers, Southern blot was conducted using six species (*M. intracellulare*, *E. coli*, *K. pneumoniae*, *M. abscessus*, *M. fortuitum*, and *M. avium*; denoted as MI, EC, KP, MA, MF, and MAV, respectively). MI showed 2–3 signal bands for each primer (Figure 3A). EC showed 2 signal bands for each primer, except Primer 2. KP showed 3–4 signal bands for each primer, MA showed 4–5 signal bands for each primer, MF showed 3–6 signal bands for each primer, and MAV showed 3–5 signal bands for each primer. Each strain showed a strong or weak hybridization level for the five primers as follows, using the ImageJ software: Primers 1, 4, and 5 in case of MI; Primers 1, 4, and 5 in case of EC (Primer 2 showed no signal); Primer 5 in case of KP; Primer 2 in case of MA; Primers 2 and 3 in case of MF; and Primers 1, 2, 3, and 4 in case of MAV showed signals with an absolute value of more than 15,000 (Figure 3B). In addition, while each of the five primers had similar total signals, species with high specificity were different for each of them, and the rankings were as follows (Figure 3C): Primer 1 was hybridized in the order of MAV, EC, and MI; Primer 2 was hybridized in the order of MAV, MF, and MA; Primer 3 was hybridized in the order of MAV and MF; Primer 4 was hybridized in the order of MAV, MI, and EC; Primer 5 was hybridized in the order of KP, MI, and EC (only the species with an absolute value of the signal greater than 15,000 have been mentioned). In other words, each of the five designed primers hybridized with DNA at a similar total level, and the specificity and hybridization intensity differed depending on the species. This shows that for at least six species, each primer can make different bands with sequence specificity.

### 3.5. The novel rep-PCR can provide molecular fingerprints alternating existing epidemiological method

**Figure 4:**
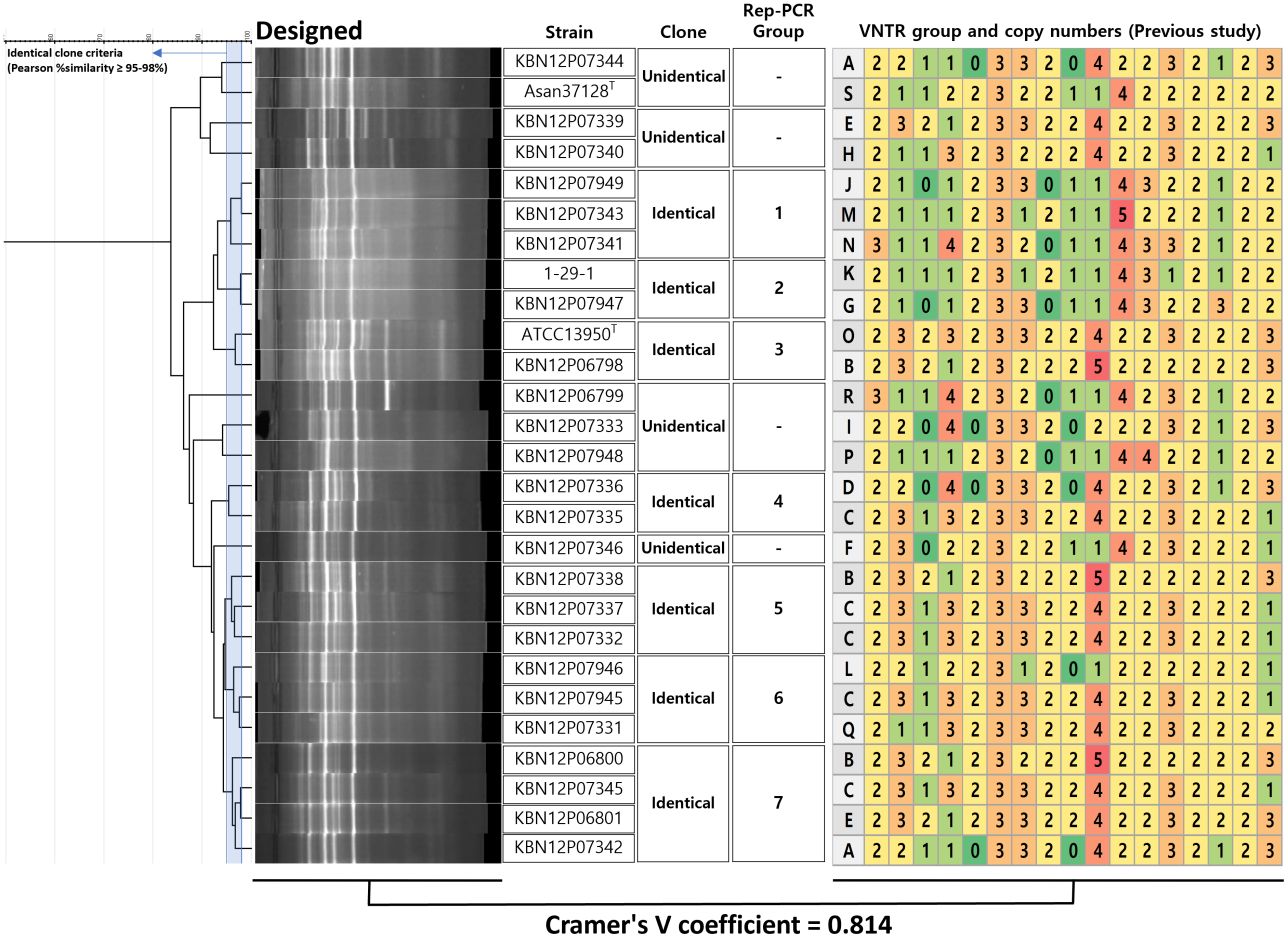
Decision of clone of *M. intracellulare* using designed rep-PCR and coefficient with VNTR method. For the decision of identical clones using designed rep-PCR, a criterion was used that Pearson similarity is 95–98%. If the similarity is higher or the same with the criterion between two or more strains, it is defined as an identical clone. The identical clone was assigned to the rep-PCR groups (*n* = 7). The VNTR (from a previous study) type means VNTR numerical patterns (copy number of VNTR 1 to 16 and VNTR-MIRU 3, 18, 19, 20, 22, 31, 33). There was a significant correlation between the groups of designed rep-PCR and VNTR, showing Cramer’s V coefficient = 0.814.

As described in 3.3, strains that shares 95 to 98% similarity are defined as a genetic clone in this study. Previously analyzed epidemiological profiles were imported from the previous study (Hatherell et al., 2016) and there were 19 PFGE profiles (Supplementary Figure S2) and 27 VNTR profiles (Figure 4 and Supplementary Figure S2) for a correlation analysis with rep-PCR. VNTR profiles were presented by alphabet type according to the combination of copy numbers of VNTR 1 to 16 and VNTR-MIRU 3, 18, 19, 20, 22, 31, 33. Designed rep-PCR generated fingerprint groups (*n* = 7) of 27 *M. intracellulare* strains, showing Cramer’s V value (correlation efficient) of 0.814.

Strains from patient A (*n* = 2) were included in rep-PCR group 7 (KBN12P06800 and KBN12P06801) and shared the VNTR profiles on 22/23 loci (96%) (Figure 4). Strains from patient B (*n* = 2) were included in different rep-PCR groups (KBN12P06798 in group 3 and KBN12P06799 in no group) and shared VNTR profiles on 8/23 (35%). This shows the correlation between the designed rep-PCR and the VNTR method.

There is an alternative approach for determining identical clones using visual discrimination in Supplementary Figure S2. The patterns of designed rep-PCR were grouped (*n* = 8) according to the visual patterns. Among the strain belonging to group 1, five strains had almost the same PFGE pattern. Some of the strains belonging to group 1 also had the same VNTR types B (*n* = 3) and C (n = 5), showing Cramer’s V value (correlation efficient) of 0.979 and *p* = 0.004. (Their PFGE patterns were difficult to compare due to the lack of identified pattern information.) This visual discrimination can be an alternative method for researchers that omit the using gel analyzing software. For reference, the entire data of the rep-PCR band for all samples are in Supplementary Figure S3-S9.

## 4. Discussion

In the MAC-PD, a recurrence caused by strains having different genotypes are frequent (Koh et al., 2017; Akoglu, 2018; Heidari et al., 2018; Gautam, 2022; Wang et al., 2022), requiring a more advanced approach than naïve species identification. Various studies on the efficacy of NTM treatment have employed epidemiological approaches of the intraspecies classification level using (Englund, 2003; Bussone et al., 2012; van der Zanden et al., 2012; San Millán et al., 2013; Thomson et al., 2013, 2014; Koh et al., 2017; Heidari et al., 2018; Karauz et al., 2018; van Weezep et al., 2019; Gautam, 2022; Zhao et al., 2022). Since WGS, PFGE, and VNTR require a specialized technique, labor-intensive or time-consuming approaches, several clinical studies used rep-PCR (Cangelosi et al., 2004; Jagielski et al., 2016; Jhun et al., 2018a,b; Shin et al., 2020a,b; Furuuchi et al., 2022) for diagnosis of reinfection/relapse, recurrent after latency, evaluation of antibiotics therapy in recurrent MAC-PD, and MAC bacteremia follow-up study.

In this study, we developed a novel rep-PCR intending to use it as an efficient genotype fingerprinting technique for studying the epidemiology of *M. intracellulare.* We designed five primers derived from repetitive sequences of the bacterial genome using *in silico* approach. Unlike previous published Gram-negative bacillus-derived primers, a novel rep-PCR was designed based on the mycobacterial genome. Therefore, the novel rep-PCR has sequences-based rationales for revealing fingerprinting patterns in *M. intracellulare* clinical isolates.

The designed rep-PCR showed a statistical correlation with the VNTR profiles (Cramer’s V value of 0.814) (Figure 4). While the VNTR technique performed 23 PCR reactions for one test of one sample, rep-PCR performed 2 PCR reactions including reproducibility tests. Therefore, this rapid method can be an alternative to an existing epidemiologic technique that can be used to determine whether the redevelopment of MAC-PD results from relapse or re-infection of *M. intracellulare*.

In this study, the designed rep-PCR were proven to be nucleotide-specific responses. First, since the Southern blot has been used to confirm whether genetic diversity across samples can be distinguished according to the nucleotide sequence of the primer (Englund, 2003; Gautam, 2022), in this study, southern blotting was used to confirm that the nucleotide characteristics of the five primers contributed to the distinction of species, based on the hybridization intensities (Figure 3). Second, *in silico* verification can be used to monitor the usefulness of PCR diagnosis before *in vitro* experiments, and the rationale is based on the real genetic sequence (San Millán et al., 2013; van Weezep et al., 2019). Differences across strains were identified when the designed five primers were applied to three *M. intracellulare* strains using *in silico* PCR (Table 2). This reflects a shift in the position of the repetitive sequences in the genomes of the three strains, and the actual PCR also produced varied electrophoretic patterns depending on the strain. Third, since the reproducibility tests for samples before and after subculturing can reflect that the PCR is not the non-specific response (Cangelosi et al., 2004; Zhao et al., 2022), it was confirmed using samples before and after subculturing that designed rep-PCR has a greater level of reproducibility (Pearson similarity 95–98%) than previous published rep-PCR (Figure 2).

In this study, designed rep-PCR showed 7 identical groups and statistical correlation with the VNTR profiles (Cramer’s V value of 0.814) (Figure 4). There were two sets of serial isolated *M. intracellulare* strains; the first set (KBN12P06800 in group 7 and KBN12P06801 in group 7 from a patient A) showed same rep-PCR group and shared on 22/23 VNTR loci (96%) (Figure 4). The second set (KBN12P06798 in group 3 and KBN12P06799 in no group from patient B) showed different rep-PCR groups and shared on 8/23 VNTR loci (35%). This can emphasize correlation between designed rep-PCR and VNTR profiles. While the VNTR technique performed 23 PCR reactions for one test of one sample, rep-PCR performed only 2 PCR reactions including reproducibility tests. Therefore, this rapid method can be an alternative to an existing epidemiologic technique that can be used to determine whether the redevelopment of MAC-PD results from relapse or re-infection of *M. intracellulare*.

The discovery of *M. intracellulare*-derived highly repeated DNA sequences in this study was motivated by previously uncovered 24 bp-scaled direct repeat (DR) sequences in *M. bovis (*Doran et al., 1993*)*. Research on *M. bovis* has indicated the possibility of searching for novel repetitive sequences and developing a new rep-PCR for *M. intracellulare* strain typing, which is expected to be more effective in classifying *M. intracellulare* than previous published rep-PCR from Gram-negative bacteria-derived sequences. The classical genomic DNA library approach of investigating the repetitive sequences in *M. bovis* was replaced by an *in silico* approach in this study and employed to identify the repetitive sequences of *M. intracellulare*. Interestingly, the sequences of the five designed primers were included in specific genes or hypothetical protein sequences within the *M. intracellulare* ATCC 13950 genome. Primer 2, which did not hybridize with the rep-PCR product from *E. coli* (Figure 3), included genomic sequences of *M. tuberculosis, M. kansasii, M. ostraviense, M. avium, M. malmoense, M. ulcerans,* and *M. shottsii.* We specified Primer 2 as a ‘Mycobacteria specific sequence’ in this study (Supplementary Table S4). Therefore, this study proposes an approach to explore genetically preserved repetitive sequences between specific bacterial species or highly related species, which is expected to minimize the time spent compared to the traditional DNA library methods.

Among the technologies related to rep-PCR, a commercial kit for rep-PCR-based mycobacteria strain typing was released in 2003 and has been used in many clinical epidemiological studies on the therapeutic efficiency of MAC (Bussone et al., 2012; van der Zanden et al., 2012; Thomson et al., 2013, 2014; Koh et al., 2017; Jhun et al., 2018a,b; Karauz et al., 2018), including *M. intracellulare*. However, due to the discontinuation of the product, follow-up research is impossible. Subsequently, in 2019, a method was reported to conduct species-level identification and strain-level differentiation in real-time through a combination of nanopore sequencing and rep-PCR using Oxford Nanopore Technologies (Krych et al., 2019; Thomassen et al., 2021, 2022). Newly developed commercial DNA chips are also used to analyze the rep-PCR patterns (Bernasconi et al., 2020). These trends reveal significant interest in and necessity of clinical use of rapid epidemiological techniques for studying MAC, including *M. intracellulare*. These also suggest that the novel rep-PCR should be developed continuously for the sustainability of follow-up studies, combined with cutting-edge technologies as a rapid diagnostic tool in further study.

However, this study has some limitations. First, the *M. intracellulare* strains in this study were isolated from patients with pulmonary disease, and only 2 sets of strains derived from the same patient that could determine re-infection/reactivation were available. Based on the results of this study, a new rep-PCR was developed and validated as DNA fingerprinting for *M. intracellulare*, however, further studies are needed to diagnose relapse. Second, the NTM strains used as controls in this study were not related to pulmonary diseases, and were used to verify the specific fingerprinting for *M. intracellulare* by comparing the fingerprint patterns of each species. Third, since the method in this study distinguish the strains by reading PCR band patterns, this result may vary depending on conditions such as electrophoresis. Therefore, further study needs to be combined with advanced techniques (e.g., nanopore sequencing) (Krych et al., 2019; Thomassen et al., 2021, 2022) that can reduce differences between experimental conditions using *M. intracellulare* isolated from clinically diagnosed patients with relapse and NTM control isolated from patients with pulmonary disease.

## 5. Conclusion

We developed a new rep-PCR primer that enables rapid fingerprint profiling of *M. intracellulare* as an alternative to rep-PCR derived from Gram-negative bacteria. Bacterial repetitive sequences through the *M. intracellulare* genome were screened using a strategy of utilizing 5 kb-scaled genomic fragments *in silico*. The Novel designed rep-PCR can make identical clone groups (*n* = 7) in 27 *M. intracellulare* strains, with a 95–98% of reproducibility. And these clone groups had a correlation value (0.814) with the VNTR, reference epidemiological method. This method can be a promising diagnostic technique for the *M. intracellulare* clinical isolates from patients with relapsed pulmonary disease.

## Data availability statement

The raw data supporting the conclusions of this article will be made available by the authors, without undue reservation.

## Author contributions

J-IS analyzed, and interpreted data as well as developed study concept and wrote manuscript. J-HH, K-MK, J-GC, S-RP, H-EP, J-SP, and J-HB contributed to part of the experiments. M-HJ, S-CB, W-KL, and H-LK wrote manuscript. J-WY and M-KS developed the study concept, obtained funding and ethics, interpreted data, and wrote manuscript. All authors contributed to the article and approved the submitted version.

## Funding

This research was supported by the Basic Science Research Program through the National Research Foundation (NRF) funded by the Ministry of Education (NRF-2021R1I1A2045131), and by biomedical research institute fund (GNUHBRIF-2021-0011) from the Gyeongsang National University Hospital, and by the Research Program (2022ER250300) funded by the Korea Centers for Disease Control and Prevention, the Ministry of Health and Welfare, Korea.

## Conflict of interest

The authors declare that the research was conducted in the absence of any commercial or financial relationships that could be construed as a potential conflict of interest.

## Publisher’s note

All claims expressed in this article are solely those of the authors and do not necessarily represent those of their affiliated organizations, or those of the publisher, the editors and the reviewers. Any product that may be evaluated in this article, or claim that may be made by its manufacturer, is not guaranteed or endorsed by the publisher.

## Footnotes

^1^ https://www.ncbi.nlm.nih.gov/nuccore/NC_016946.1

^2^ https://www.novoprolabs.com/tools/repeats-sequences-finder

^3^ https://blast.ncbi.nlm.nih.gov/Blast.cgi

^4^ http://insilico.ehu.es/PCR/index.php?mo=Mycobacterium
