# Supplementary material for: A novel repeat sequence-based PCR (rep-PCR) using specific repeat sequences of Mycobacterium intracellulare as a DNA fingerprinting
Source: Front Microbiol. 2023 Apr 6;14:1161194. doi: 10.3389/fmicb.2023.1161194 (PMC10117815; doi:10.3389/fmicb.2023.1161194)
Supplement: Supplementary file 1 [file Data_Sheet_1.PDF]

Supplementary Material

A Novel Repeat Sequence-Based PCR (rep-PCR) using Specific Repeat Sequences of *Mycobacterium intracellulare* as a DNA Fingerprinting

Jeong-Ih Shin, Jong-Hun Ha, Kyu-Min Kim, Jeong-Gyu Choi, Seo-Rin Park, Hyun-Eui Park, Jin-Sik Park, Jung-Hyun Byun, Myung-Hwan Jung, Seung-Chul Baik, Woo-Kon Lee, Hyung-Lyun Kang, Jung-Wan Yoo* and Min-Kyoung Shin*

*** Correspondence:** Min-Kyoung Shin and Jung-Wan Yoo

: mkshin@gnu.ac.kr and chareok-sa@daum.net

1 Table Contents

1.1 Supplementary Table S1. Rationale of this study comparing classical repetitive sequences screening

1.2 Supplementary Table S2. 7,645 repeat sequences from 5 kb-scaled fragments

1.3 Supplementary Table S3. 51 Selected sequence (16-92 bp) from 7,645 repeat sequences

1.4 Supplementary Table S4. Sequences, length, primer, and coding region of candidates repeats for generating primers

1.5 Supplementary Table S5. The Locus, Identity, and Strand of designed primers aligned with NCBI genome sequences of *M. intracellulare* ATCC 13950

1.6 Supplementary Table S6. *In silico* PCR amplicons using various mycobacteria species

2 Figure Contents

2.1 Supplementary Figure S1. Comparison of reproducibility between novel and previously published rep -PCRs

2.2 Supplementary Figure S2. Analysis of correlation between the designed rep-PCR and other molecular epidemiological methods (PFGE and VNTR) techniques using 27 *M. intracellulare* strains

2.3 Supplementary Figure S3. Applications of designed rep-PCR on *M. intracellulare, S. aureus, E. coli, K. pneumoniae, M. fortuitum, M. abscessus*, and *M. avium*

2.4 Supplementary Figure S4. Applications of previous published rep-PCR(BOX-PCR) *on M. intracellulare, S. aureus, E. coli, K. pneumoniae, M. fortuitum,M. abscessus*, and *M. avium*

2.5 Supplementary Figure S5. Applications of previous published rep-PCR($(CCG)_4$-PCR) on *M. intracellulare, S. aureus, E. coli, K. pneumoniae, M. fortuitum,M. abscessus*, and *M. avium*

2.6 Supplementary Figure S6. Applications of previous published rep-PCR(ERIC-PCR) on *M. intracellulare, S. aureus, E. coli, K. pneumoniae, M. fortuitum,M. abscessus*, and *M. avium*

2.7 Supplementary Figure S7. Applications of previous published rep-PCR(ERIC+BOX-PCR) on *M. intracellulare, S. aureus, E. coli, K. pneumoniae, M. fortuitum,M. abscessus*, and *M. avium*

2.8 Supplementary Figure S8. Applications of previous published rep-PCR(REP+BOX-PCR) on *M. intracellulare, S. aureus, E. coli, K. pneumoniae, M. fortuitum,M. abscessus*, and *M. avium*

2.9 Supplementary Figure S9. Applications of previous published rep-PCR(REP+ERIC+BOX-PCR) on *M. intracellulare, S. aureus, E. coli, K. pneumoniae, M. fortuitum,M. abscessus*, and *M. avium*

Supplementary Table S1. Rationale of this study comparing classical repetitive sequences screening

	Reference method (26)	Modified method in this study
**Step 1. Method of DNA library construction**	**Genomic DNA library**	**Modified DNA library *in silico***
Methods for DNA fragmentation	Fragmentation for inserts by genomic digestion	Fragmentation of genome *in silico*
Size of DNA fragments	3-8 kb	5 kb
No. of DNA fragments	No. of *E. coli* recombinants: 200	No. of fragments: 200
**Step 2. Searching method for repetitive sequence**	**DNA-DNA hybridization**	**Finding repeats in fragments *in silico***
No. of 1st screened recombinants or sequences	20 recombinants (Strong signal)	7,645 sequences (All of repeats in 5kb fragments)
No. of 2nd screened recombinants or sequences	5 recombinants (Similar patterns in strains)	50 sequences (Size range: 16-62bp)
No. of 3rd screened recombinants or sequences	1 recombinant	15 sequences (More than 10 regions Bit score ≥ 32.2)
**Step 3. Finding repetitive sequences**	**Finding repeats in insert**	**Genome-primers hybridization *in silico***
1st screening	Sequencing repeats in an insert sequence	Repeat times in genome ≥ 30
2nd screening	Finding repeats in an insert sequence	Identity with genomic sequences ≥ 90%
Result	Finding 24bp-DR sequence	Generating five primers

Supplementary Table S2. 7,645 repeat sequences from 5 kb-scaled fragments

No.	from nth 5 kb-fragment	Sequence	Position in the fragment	Length (bp)
1	1	GCGGTCGTTTCC	42, 3472	12
2	1	TGGCCGCCACCG	1261, 2603	12
3	1	CCGACGACGTC	1072, 3005	11
4	1	CCTGGCGATCG	581, 3033	11
5	1	CGCCCTGATCC	326, 1133	11
6	1	CGCCGCCGGCA	497, 668	11
7	1	CGCGGTGTCGT	2142, 4565	11
8	1	CGCTGGCCGAA	1189, 2699	11
9	1	GCCCGGGCCGG	2713, 4619	11
10	1	GCCGCCCGGGC	2710, 3463	11
11	1	GGCGATCAGCC	2508, 4123	11
12	1	TCTACGTCGAC	2369, 4373	11
13	1	CAAGCGCAGC	785, 2808	10
14	1	CCCGGCGGGC	4347, 4458	10
15	1	CCGACCGCGG	69, 2942	10
16	1	CCGCCGTCGC	490, 2879	10
17	1	CCGGGTGGCG	2385, 4586	10
18	1	CGACGCCGCG	383, 3978	10
19	1	CGCCGACGCC	1220, 3975	10
20	1	CGCGCGGTGT	3607, 4563	10
21	1	CGGCCGAGCA	2867, 4304	10
22	1	CGGCGACGAC	377, 2078	10
23	1	CTGCTGTCCG	183, 2206	10
24	1	GACCGATGAC	2, 2217	10
25	1	GATCGCCGAC	1217, 4751	10
26	1	GGGACGTTCG	1849, 4358	10
27	1	GGGCGCGGGT	2754, 4436	10
28	1	GTCATCGGCG	543, 3592	10
29	1	GTTCTCGTTG	2415, 4170	10
30	1	TTCGAAAACG	411, 2042	10
31	2	GGCATCCACGACG	2025, 4548	13
32	2	ACCTCGATCGC	3394, 4686	11
33	2	ACGGCGTGGGC	55, 853	11
34	2	ACTCCGTCGAC	640, 2602	11
35	2	CAACGGAACTC	3289, 4084	11
36	2	CCGTCCTGCCC	3664, 4324	11
37	2	GACGTCGGTGG	1463, 3204	11
38	2	GATGCAGCGCA	2642, 3272	11
39	2	GCGGAATCGAC	46, 1203	11
40	2	GCTGCGCTATC	2054, 2885	11
41	2	GGGCGGCACCC	1427, 3446	11
42	2	AACACCGAGG	1986, 4218	10
43	2	AACCTCACCG	1110, 1521	10
44	2	CCCACCGTCC	2373, 4320	10
45	2	CGACGTCGTC	773, 3017	10
46	2	CGCCGGCGGC	797, 2941	10
47	2	CGGCCAGAAT	287, 328	10
48	2	GACGACGGCA	696, 2301	10
49	2	GACGTCGACG	2091, 2454	10

50	2	GCCGTTCGGG	230, 2990	10
51	2	GCGTTCCTCA	1083, 4188	10
52	2	GGTGGTCAAC	869, 1706	10
53	2	GTCGACGTCG	771, 3201	10
54	2	GTTCTCGATC	2432, 3069	10
55	2	TCGCGATGCA	742, 1357	10
56	2	TTCGGCGGTG	810, 3849	10
57	3	CGCGGCGGCAAGGGCGTC	688, 1306	18
58	3	TACGCCAAGCGCACC	646, 1264	15
59	3	TAGACTCAGCCCGAT	1603, 1661	15
60	3	GATCGCCGACCTCG	456, 4427	14
61	3	GCTGGTCGGCGCG	1047, 1356	13
62	3	AACCTGCGCGAC	1027, 4173	12
63	3	ACGACGAGCTG	512, 1040	11
64	3	CATCGTGCGGC	189, 732	11
65	3	CCGGATCATCG	561, 4121	11
66	3	CGGCTACGCCA	642, 4289	11
67	3	CGTCGACAAAC	531, 4268	11
68	3	GATCGTCGACG	114, 1371	11
69	3	GCCGCGTGGAT	3234, 3369	11
70	3	GCGGCACCCGG	1885, 2001	11
71	3	GCGGCGCCGTC	1821, 2488	11
72	3	GGCCGCGGCGC	1627, 3509	11
73	3	GGGCCGCGGCG	1302, 1626	11
74	3	TGCTGAACAAC	53, 2431	11
75	3	TTCTTCGTGTG	745, 2340	11
76	3	ACCTCCGGCG	1162, 1252	10
77	3	CCCGCACCGC	836, 2126	10
78	3	CCTGCTGGCG	867, 3668	10
79	3	CCTGGCCAAG	435, 477	10
80	3	CGCCGGATCG	499, 2283	10
81	3	CGCCGTCGCC	36, 2357	10
82	3	CGCGGCCGGC	1431, 4508	10
83	3	CGCTCGGCCG	2679, 3528	10
84	3	CGGTCGCGCG	3689, 3709	10
85	3	CTGATCGCCG	2824, 4425	10
86	3	GCAAGGCGGG	1448, 2956	10
87	3	GCCCGAGGAG	882, 1639	10
88	3	GCCCGGGGAC	1856, 4025	10
89	3	GCGCGGCCGG	1701, 4507	10
90	3	TCGGCGGCAT	2383, 2581	10
91	3	TGTATGACCG	1334, 3844	10
92	4	TTGGTCCCGATCACCT	4125, 4326	16
93	4	CACGTCGGGCACCT	4442, 4643	14
94	4	CGCCGCGCTGGCC	90, 2638	13
95	4	CGTGCTGCTCAGT	1986, 3339	13
96	4	CCGCGATGTCGG	485, 4234	12
97	4	GGCCGGCGGGTT	3908, 4316	12
98	4	ACCAACGGCTC	1205, 2868	11
99	4	CACCCCGGAGC	3363, 3896	11
100	4	CCCCGAACTGC	1147, 3492	11
101	4	CGCGATGGCGT	2042, 4613	11
102	4	GCGCAACGACA	1943, 3777	11
103	4	TCGACCGCGCC	29, 3482	11
104	4	TGCCTGGGGCA	417, 3439	11
105	4	ACAAGACGCC	1236, 1772	10

106	4	ACTCGATGAT	1565, 2400	10
107	4	AGCAGACCCC	1696, 3416	10
108	4	CACCGTCGAC	1228, 3477	10
109	4	CCCGACCGGC	1093, 3101	10
110	4	CCTGGTGGTC	2716, 3007	10
111	4	CGAGTCGATC	1369, 3711	10
112	4	CGCCGCGATG	483, 4610	10
113	4	CGCGCTCATC	663, 828	10
114	4	CGGCGACCCG	1087, 3572	10
115	4	CTGCGTGGGC	81, 2899	10
116	4	GACGTCGGCG	758, 3959	10
117	4	GATCCGGGGC	1063, 2509	10
118	4	GCCCGACCGG	3100, 3153	10
119	4	GCGCGGGCGC	2838, 3374	10
120	4	GGACACCTGC	4070, 4628	10
121	4	GGACGCGCAG	2734, 3073	10
122	4	TGACGGTGAT	4279, 4483	10
123	4	TTCGGCGTCC	2435, 4013	10
124	4	TTTTGCAGCG	4572, 4602	10
125	5	CCGGCTTGACGTCGCGGTGGA	554, 1813	21
126	5	AGCACCTTGACCGCGACG	853, 2115	18
127	5	CCGCGATGATGA	1177, 4085	12
128	5	CGCGCAGCACCT	848, 3111	12
129	5	GATCGGCGCGCA	161, 3609	12
130	5	GGCAGCGGCCCG	646, 715	12
131	5	GGCGTCGACGGC	393, 1742	12
132	5	GTGGTGCTGATC	532, 2573	12
133	5	TGCACCCGGACC	148, 4240	12
134	5	CCCGCCGGTGG	1274, 4158	11
135	5	CCGCCGCGATC	2020, 3746	11
136	5	CCTGCAGCGCG	1855, 3015	11
137	5	CGCGTCGGCGA	18, 612	11
138	5	CGCTCGGGGTG	1069, 2781	11
139	5	CGGGGGCGGGC	54, 1259	11
140	5	CGGGGTGGTCA	966, 3873	11
141	5	GACCAGACCGA	4302, 4665	11
142	5	GACGGCGACGA	756, 3179	11
143	5	GAGGTCGCGGG	885, 4449	11
144	5	TCGACGCGGCG	1295, 4103	11
145	5	ACCGCCGCGA	1425, 2019	10
146	5	ATCACCTTGA	514, 3038	10
147	5	CCCAGGCGGC	2139, 3279	10
148	5	CCGGGGGCGG	1258, 1378	10
149	5	CGCCAGGCCC	3951, 4050	10
150	5	CGCGGATCGG	2414, 3605	10
151	5	CGGCGCGGCG	2287, 2977	10
152	5	CGGTCTGGGT	458, 1723	10
153	5	GACCGGCGCG	1414, 3460	10
154	5	GATGACGATG	1163, 4020	10
155	5	GCCGGTGTCG	744, 1112	10
156	5	GCGACCGGCG	310, 1412	10
157	5	GGACCGCCGC	2017, 3251	10
158	5	GGACGGCGAC	1407, 3178	10
159	5	GGCGGGGCAG	35, 4460	10
160	5	GGGATCGACG	1291, 2969	10

161	5	GGGCAGCGGC	714, 1532	10
162	5	GTAGCGCGCG	1222, 4695	10
163	5	TCCCGCCGGT	992, 4157	10
164	5	TCGGGATCGT	3101, 3143	10
165	5	TCGGGCGAGA	241, 421	10
166	5	TGATCTCGAT	623, 1492	10
167	5	TTCGGGGTCT	2087, 2360	10
168	6	TGCCCCTGGTCGGGGTAGCC	3330, 3390	20
169	6	GGGTAGCCGCCCTGCTC	3474, 3522	17
170	6	CGGGTAGCCGCCCTGC	3317, 3473	16
171	6	GTATCCCCCGCCCTG	2969, 3056	15
172	6	CCGTAGTCGGCGGG	2865, 2883	14
173	6	CGCCGTAGCCGCCC	2818, 2842	14
174	6	GACCGTAGTCGGCG	2863, 3166	14
175	6	GCCAGCGCGGCGA	955, 1705	13
176	6	GCGGCGGGTAGCC	3451, 3499	13
177	6	GTAACCGCCCTGG	3233, 3422	13
178	6	TGGCCGTAGTCGG	2880, 2916	13
179	6	GGGGATCCGGGC	574, 4333	12
180	6	GGGTCACGGGCG	1862, 1930	12
181	6	TGACCGTAGTCG	2862, 2934	12
182	6	ACCCACCACCC	913, 3088	11
183	6	CCGCCAGCGCG	953, 2184	11
184	6	CCGCCGTAGCC	2841, 3186	11
185	6	CCGGCTGGCCG	2154, 3211	11
186	6	CGGGCCACCGC	3200, 3581	11
187	6	GCCGTCGGCCA	1761, 2573	11
188	6	GGCGGCCTCGG	1233, 2957	11
189	6	GGTCCGACAGC	1292, 2638	11
190	6	TCCCGCATGGT	1342, 4228	11
191	6	TCCTGGCGGGC	3045, 3147	11
192	6	TGCGGCGGGTA	3372, 3450	11
193	6	TGGTAGCCCGG	3360, 3438	11
194	6	CACCAGGCCA	85, 1821	10
195	6	CAGCCGGGCG	1779, 2080	10
196	6	CCGGCGCGCG	1330, 2366	10
197	6	CCTCGCGAAT	1516, 3893	10
198	6	CGCAGCGCCA	1207, 2258	10
199	6	CGCGGCCGAT	2731, 4653	10
200	6	CGGGGCGTAG	2296, 3128	10
201	6	CGTCGTCGAG	1691, 2782	10
202	6	GACCAGGGTC	1449, 1857	10
203	6	GCAGCAGGTA	617, 1505	10
204	6	GCCCAGCAGC	286, 834	10
205	6	GCCGCCCGGC	672, 1677	10
206	6	GCGGCCGCTG	2771, 3295	10
207	6	GGCGGGGCGT	3110, 3126	10
208	6	GGGCTGGCGC	496, 3491	10
209	6	GGGGCTGGCG	495, 2951	10
210	6	GTACCAATCC	1535, 2385	10
211	6	GTTGCGCTGG	23, 900	10
212	7	GAGGATCAGCGG	3446, 3602	12
213	7	TCAGCACGGTAT	1408, 2016	12
214	7	AGATCAGGACC	98, 340	11
215	7	CCGGAGGCCGT	2210, 3887	11
216	7	GCGATCGCCCC	1729, 3489	11

217	7	GGGATGGCCGA	1805, 2618	11
218	7	GTCGGGGTCGA	1677, 3380	11
219	7	TCAACCTGCGC	2630, 3630	11
220	7	TCGACGGTGAG	1358, 2901	11
221	7	ACTCCGGCCG	2472, 3152	10
222	7	CATCCCCGGT	245, 2731	10
223	7	CCTGTTGACC	1631, 2320	10
224	7	CGAGGCCGCT	2095, 4333	10
225	7	CGCGGCTCAT	1423, 2804	10
226	7	CGGAATCGGA	2005, 2688	10
227	7	CGGCTCGGCG	1155, 3765	10
228	7	GACGCGCTGC	4607, 4643	10
229	7	GCCGAGGCCG	2093, 4184	10
230	7	GCGTCGACGT	576, 630	10
231	7	GCGTTCCAGC	591, 4307	10
232	7	GCTGGCCGAT	3270, 4474	10
233	7	GTCGTGTTCG	925, 3314	10
234	7	TCGACGATCA	318, 1465	10
235	7	TGCGGTCGGT	2921, 3028	10
236	7	TGGCGGCGAA	716, 2524	10
237	7	TGGTTGCGGT	372, 3024	10
238	7	TTGACGAGCG	886, 4035	10
239	8	GAGTTTTTCCTCC	1266, 3879	13
240	8	GCAGCGGCGGGG	2206, 3359	12
241	8	GCCCCACCGTCT	30, 2067	12
242	8	GGCGAGGCGGCC	186, 540	12
243	8	TTCGCCGGAAGG	176, 4664	12
244	8	ATGGGCGCGCC	3697, 4579	11
245	8	CGCCTCCTCGA	1963, 4440	11
246	8	CGCTCGGTGGC	3520, 3718	11
247	8	GCTCAAGGCGG	215, 527	11
248	8	GGCGTGCCGCC	2361, 4000	11
249	8	TGCCCTCGACG	236, 2079	11
250	8	TGCTCGAAGGT	2511, 3799	11
251	8	ACCGGCCCTG	1371, 2748	10
252	8	ACCTTGCTGG	1257, 4428	10
253	8	ACTGCCGAGC	477, 4648	10
254	8	ATGGCCAGCC	3226, 4318	10
255	8	CAGCTCGCCG	2618, 2810	10
256	8	CCCCGACTAC	260, 4618	10
257	8	CCCGATGGCC	3765, 4034	10
258	8	CGGCCAGCTC	359, 2614	10
259	8	CGGTTGTCAT	829, 2145	10
260	8	CTCGCCGCGA	2041, 2928	10
261	8	GCACACCGGA	448, 1918	10
262	8	GCAGCCGAGC	2191, 3629	10
263	8	GGCGATCCCG	3759, 4083	10
264	8	TCGCGCGCCT	1946, 3853	10
265	8	TCGTCGACCA	94, 4051	10
266	9	GCCGTGCCCGCGA	2811, 4008	13
267	9	GGACGCCATCGCC	2284, 2894	13
268	9	CGCGATGCCGGC	737, 4283	12
269	9	ACCGACGCCGA	3306, 4561	11
270	9	CGATCCCAACG	2681, 3406	11
271	9	CGCCGGCGGCG	3247, 3997	11

272	9	CTCGCGGTGCG	147, 1760	11
273	9	CTGATGGCCGC	714, 3180	11
274	9	GATCCGCCGAC	2065, 2780	11
275	9	GCACGACGCCT	1320, 3617	11
276	9	GGAGCGCGCGA	2417, 3084	11
277	9	TGCGCCGCCGC	1608, 4733	11
278	9	ACCGCCGAAC	1738, 2864	10
279	9	CAGGCCGCCG	3759, 4172	10
280	9	CCGAAGCCGC	2911, 3040	10
281	9	CCGAGACCGC	1504, 2666	10
282	9	CGCCGTACTC	137, 2013	10
283	9	CGCGTGCGCG	1185, 1665	10
284	9	CGGCGCCGTG	2200, 2807	10
285	9	CGGCGGACGC	2890, 4746	10
286	9	CGGCGGCCGG	1455, 3679	10
287	9	CTCGAGGCGA	1005, 2760	10
288	9	CTGTTGCGCC	183, 1367	10
289	9	GCAGCGCCGC	5, 198	10
290	9	GCAGGCGCGC	215, 1516	10
291	9	GCCCGGCAGC	111, 1040	10
292	9	GCGATCCAGG	3753, 4235	10
293	9	GGCGCAGCGG	171, 1591	10
294	9	TCCGAGACCG	430, 2665	10
295	9	TCCTGAACGC	2977, 3142	10
296	9	TTGTCGCGGC	1778, 4646	10
297	10	GCCGAACCGGTCGAA	756, 792	15
298	10	GACGAGCCGGCCG	1053, 1242	13
299	10	GATCGCGCGCCGG	1149, 4057	13
300	10	CCAGCAGCGAGT	3921, 4494	12
301	10	CGAGCCGGTCGA	776, 947	12
302	10	CGCGCTCATCGC	2352, 2592	12
303	10	CGTCGGGTCGGC	3140, 3269	12
304	10	GAACAGCAGCGC	3856, 4570	12
305	10	CCCCCGCCGCC	622, 723	11
306	10	CCGGCGGTCCC	2758, 4453	11
307	10	CCGGCTGAACC	1352, 2261	11
308	10	CCGGTCGTCTC	894, 2400	11
309	10	CGAACCCGTCG	1181, 1286	11
310	10	CGAGCAGCTCC	3037, 3082	11
311	10	CGCGGTGGCCA	2969, 4243	11
312	10	CGGACCGCCAG	4382, 4671	11
313	10	CGGCCGAGGCG	523, 1273	11
314	10	CGGCGACACGG	1547, 1892	11
315	10	CTCGAGGAGAC	873, 1086	11
316	10	GCCAGCGCCGC	2644, 4586	11
317	10	GCGACGAGCCG	1012, 1240	11
318	10	GCGCCCGCGGA	1209, 4086	11
319	10	GGACCAGCAGC	3714, 3918	11
320	10	GGGCGACGCGG	1515, 3205	11
321	10	GTGACGCCGAG	2913, 3677	11
322	10	TCGCCGCGACG	1609, 3440	11
323	10	TGGTCACCGAC	1111, 1441	11
324	10	ACCCCCGCCG	722, 1041	10
325	10	AGCCACGGCT	39, 1390	10
326	10	AGCCCGCCGC	961, 3840	10
327	10	AGGCCGAGGC	2131, 2296	10

328	10	ATCCGGGTTG	4561, 4706	10
329	10	CAACTCTCGC	107, 415	10
330	10	CCCGCAGGCG	1002, 1488	10
331	10	CCCGCGTTGG	2385, 4427	10
332	10	CGCCACCATG	978, 3700	10
333	10	CGCGGCACCA	4193, 4280	10
334	10	CGGGAATCGC	309, 4131	10
335	10	CGGTCACCGC	397, 3190	10
336	10	CGGTGACCCC	1324, 3028	10
337	10	GAGGTCGACG	699, 1767	10
338	10	GCCGATCGCG	1146, 4399	10
339	10	GCCGTGCGCC	561, 2942	10
340	10	GCGCCGGCGA	3728, 4478	10
341	10	GGCCGCCACC	975, 1868	10
342	10	GTCCTGCACG	1, 3589	10
343	11	GCCGGCGCCGGGGCG	1342, 1441	15
344	11	CGAGCTGGTCGCCG	3072, 3552	14
345	11	GCCGGGGCGGCCG	1288, 2023	13
346	11	AATCCCCGACCA	1754, 2492	12
347	11	CCGCGGCCAGCG	812, 878	12
348	11	ACGACGCCGAC	4085, 4181	11
349	11	CACCGACCCGC	3582, 4332	11
350	11	CCACCGGCGCC	898, 3416	11
351	11	CGACCCCGACG	461, 4173	11
352	11	CGACGTGCCGG	404, 4143	11
353	11	CGGCGAAGCCG	313, 3618	11
354	11	CGGCGAGGCGG	1419, 3456	11
355	11	CTGGCGCCGCC	2522, 2656	11
356	11	GACCAACCTGT	302, 2434	11
357	11	GCAGCCGGCGC	735, 1339	11
358	11	GCCGGGGCCGC	1234, 1993	11
359	11	GCGGGACTGGG	3120, 3822	11
360	11	GGCGGGTGCCG	1377, 2037	11
361	11	AACGTGCTGC	285, 2809	10
362	11	CCCGCGACAC	835, 4274	10
363	11	CCGGAGCGGC	1358, 2012	10
364	11	CCGGCTGGTC	3174, 4545	10
365	11	CCTGGAGTCC	2053, 3984	10
366	11	CGCCGCCGCT	765, 4202	10
367	11	CGCGCAACGG	134, 2418	10
368	11	CGGCGGCGCC	2073, 4446	10
369	11	CGTGCTGGCC	3228, 3768	10
370	11	CGTGGCGCCG	963, 1589	10
371	11	GATCACCGCC	3321, 3972	10
372	11	GATCGGCCGG	67, 3405	10
373	11	GCAGCGTGAT	202, 1886	10
374	11	GCCCGCGGCC	810, 4391	10
375	11	GCGCCGCCGC	764, 2548	10
376	11	GGGAGCCGGG	1225, 1989	10
377	11	GGGCTACATC	2757, 4560	10
378	11	GGTGCCGCCG	92, 1112	10
379	11	GGTGTGGTGC	87, 246	10
380	11	GTTCTGCGGC	1911, 2145	10
381	11	TGCCCGCCGA	2846, 4607	10
382	12	AGCGTCGACTTG	2528, 3442	12

383	12	CCCGGCGGCGGC	1753, 4520	12
384	12	CCGCGCGGCGGT	304, 1509	12
385	12	CGAGCTCGGCAA	1830, 2115	12
386	12	CGGCGAAATCCG	3500, 4076	12
387	12	GCCGATGCGCTG	2422, 3891	12
388	12	TCCCCGGCCGGC	603, 3754	12
389	12	CAGGGTGTCGA	1459, 4242	11
390	12	CCCGCGCGGCG	303, 4720	11
391	12	CGCCCGCGCGG	1547, 4718	11
392	12	GCCGATCGGAT	1203, 3141	11
393	12	GGCCAGCAGCA	1222, 3300	11
394	12	GGCGCACGGGC	400, 4484	11
395	12	GTCCGGCTCGT	835, 2494	11
396	12	TGCCGTCGGCA	2103, 3602	11
397	12	TTCCTGGCGGG	566, 1375	11
398	12	CAGCCCGGCG	3550, 4071	10
399	12	CCACACCACC	385, 4425	10
400	12	CGACCAGGAC	514, 1566	10
401	12	CGACCGCGGC	708, 2741	10
402	12	CGGCCGGGCC	1398, 3458	10
403	12	CTCGTCGACG	437, 1039	10
404	12	GCCAGCGTGA	986, 4402	10
405	12	GCCGGCCAGC	1219, 3259	10
406	12	GCGCGCCGGC	3255, 4477	10
407	12	GCGCGGGCAA	972, 2213	10
408	12	GCGTCGAGCC	1196, 3619	10
409	12	GGATCGACAG	1071, 1725	10
410	12	GGCCGCCGCG	532, 4386	10
411	12	GGGAGTCCGC	855, 1654	10
412	12	GTCGGGCCGG	2721, 4296	10
413	12	TCGCCACCGG	162, 3017	10
414	13	ACGCGGCCGCCCGG	2447, 4215	14
415	13	GTCGATCAGCGCG	2524, 4296	13
416	13	CGTACTCGTCGT	113, 1076	12
417	13	GACATCGCCGCG	1703, 1843	12
418	13	GCCCCCGCCGCC	945, 3760	12
419	13	GCGTCGACGTCG	3790, 4459	12
420	13	GGGTGGGCGCCG	977, 3255	12
421	13	ACGCCGACCGG	1799, 3142	11
422	13	CAACGCGTCGA	1900, 4455	11
423	13	CCCCCACCGAC	2794, 3580	11
424	13	CCGCGGCCCGC	3365, 3935	11
425	13	CGGCGACGGCC	3533, 3671	11
426	13	GCCGCGGCCCG	101, 3364	11
427	13	GCGCGGTCGCC	1025, 3007	11
428	13	GGTGAAGCTGT	888, 2777	11
429	13	GGTGCGGCGCG	3452, 4589	11
430	13	TCGCGTGCAGC	92, 4052	11
431	13	ACGGTGTCGC	2075, 2432	10
432	13	AGCACGACGG	2426, 2664	10
433	13	AGCGTGTTGG	1386, 2318	10
434	13	CCGGGTGGAC	342, 2281	10
435	13	CGCCGCGCGC	2499, 2949	10
436	13	CGCGACGGGC	2049, 4376	10
437	13	CGCGCACCAC	1488, 3977	10
438	13	CGGCGCACGG	1351, 2069	10

439	13	CGTCCTCGAC	2813, 3725	10
440	13	GACGTCGCCG	3327, 3507	10
441	13	GCCCGTCGGA	1130, 1914	10
442	13	GCCGGTCAAC	462, 1888	10
443	13	GCGACCGTCG	2877, 3736	10
444	13	GCGTCGTGGG	2929, 3208	10
445	13	GGCGCCGCGG	327, 4356	10
446	13	GGTCCAGCAT	1257, 4613	10
447	13	GGTTGGTCGC	233, 4312	10
448	13	GTTCGGCGAC	914, 2040	10
449	13	TCGTAGCCGT	1069, 2704	10
450	13	TGGCACCGAA	2650, 3283	10
451	14	GAGGGCCGCGTCGTCC	1615, 4002	16
452	14	AGTCGGGCGTGGTG	1134, 4246	14
453	14	TCGACGCGCTCGA	308, 1058	13
454	14	GAACTCGACGAT	811, 3843	12
455	14	GTTCGTCCTCGG	3126, 3653	12
456	14	CCAGGCCGAGG	3495, 4403	11
457	14	CCAGGTCGAAG	1473, 1751	11
458	14	CCGACATCAGG	969, 3321	11
459	14	CGCCGGCGCGC	1569, 2763	11
460	14	CGCCGTAGTCG	3555, 3716	11
461	14	CGGCCGCCGCG	2189, 4529	11
462	14	CGGCGATGTCG	2832, 4169	11
463	14	CGGGAGTCCAG	4124, 4607	11
464	14	CGTGGGCGTCG	801, 2231	11
465	14	GCGGCGCACGG	2086, 4476	11
466	14	GGCGTCGACGC	928, 1516	11
467	14	GGGTGTCCAGC	567, 636	11
468	14	GGTGTCGGCGT	94, 880	11
469	14	GTCACCGAAGG	1249, 1636	11
470	14	TGAAGTCGGCG	1386, 2694	11
471	14	ATGGCGCCGT	1813, 3712	10
472	14	CAGCCGGATC	10, 4311	10
473	14	CATGTTGGTG	775, 853	10
474	14	CCAGCCGGAT	9, 681	10
475	14	CCGTCGCCCA	3143, 3904	10
476	14	CCGTCGGCGT	3485, 4153	10
477	14	CGAAATCGCC	987, 1922	10
478	14	CGAGCCGCGA	3435, 4691	10
479	14	CGCGCCAGGC	132, 535	10
480	14	CGGCGCGTTC	492, 4073	10
481	14	CGGTGAGCAT	2156, 4100	10
482	14	CTCGCGCCAG	130, 3205	10
483	14	GACGGACAGC	2427, 3807	10
484	14	GCCGGTGAGC	1336, 2154	10
485	14	GCCTCGGCGG	2627, 3581	10
486	14	GCGGCTCGGC	715, 863	10
487	14	GGCCAATCCG	962, 3699	10
488	14	GGCGTGCAGG	2512, 2800	10
489	14	GGTGACGTTG	1303, 2415	10
490	14	GTAGCGCCCG	1898, 3297	10
491	14	GTCGGGCAGC	322, 4647	10
492	14	TCGAACGCCC	1190, 3110	10
493	14	TCGCGGTCGA	698, 1723	10

494	14	TTCGGTGTCC	3787, 4236	10
495	15	GTACGCCCTCAACCCCGGCCGAA	65, 88	23
496	15	AACCGTTTGCGCA	1804, 2432	13
497	15	CGAACTTCACGT	1093, 1123	12
498	15	CCCTGGCGCGG	2853, 4594	11
499	15	GACCGTGCCGC	4656, 4682	11
500	15	GCCCTCGTCGG	1647, 4314	11
501	15	GCCGCGGTCCT	290, 629	11
502	15	GCGGACCGCCG	3270, 3835	11
503	15	GCGTCGGGCCG	961, 3789	11
504	15	GGCGGCGCGCA	311, 3432	11
505	15	TGGCCGGCCCG	3712, 3854	11
506	15	AATGAGCTCG	1374, 3464	10
507	15	AGCCCACCGG	838, 1996	10
508	15	AGGTCGCCCA	2732, 3821	10
509	15	CACGTCGACG	863, 896	10
510	15	CACTGCTGGC	3080, 4531	10
511	15	CAGCGCGTTG	770, 2809	10
512	15	CAGCTCACCG	1626, 4509	10
513	15	CCGTCGACGC	1243, 3723	10
514	15	CGAGCGGGTG	2560, 3527	10
515	15	CGATCGACGA	3688, 3748	10
516	15	CGGCATCTCC	3398, 4463	10
517	15	CGTCACCGCC	2067, 2743	10
518	15	GATGTCGTCG	218, 692	10
519	15	GCCCCATCCG	2317, 2581	10
520	15	GCCGATGCCG	284, 947	10
521	15	GCCGCCTTGG	3705, 3765	10
522	15	GCCGGCGTCG	791, 3785	10
523	15	GCCTCGCGCA	876, 1926	10
524	15	GCGCTCACTG	1401, 3075	10
525	15	GCGGCACGTC	388, 994	10
526	15	GGCGATCTTG	302, 1004	10
527	15	GGCGCCGCGC	2218, 3385	10
528	15	GGCGGCGGCC	1514, 2623	10
529	15	GGTCCGACGC	748, 1918	10
530	15	GTGCACGTCC	476, 4155	10
531	15	TCGACGATCA	2162, 4284	10
532	16	CGCCGCCCCCGCCG	1097, 1277	14
533	16	CGCGCCTACTACG	184, 1599	13
534	16	CTGATCGGGTTGG	1491, 2088	13
535	16	GGACTGGCTGCGG	2855, 3192	13
536	16	CTGTGGCTGGTG	2544, 4122	12
537	16	GCCGCCCGGCGG	1209, 3568	12
538	16	GCCGGGCGGCGG	1242, 1938	12
539	16	TCACCGCGGCCG	1326, 3386	12
540	16	TCGACGCGCTGG	2011, 2611	12
541	16	AGGTCGTGTTC	2479, 3032	11
542	16	ATCGACATGGC	595, 3412	11
543	16	CACCGTCGTCG	4144, 4411	11
544	16	CGACGAACCGC	939, 4624	11
545	16	CGCCGCGCTCG	276, 1400	11
546	16	CGGCCAAGACC	123, 827	11
547	16	CGGCGGTGGTG	146, 4513	11
548	16	GCCGCCGAGAA	91, 4310	11
549	16	GCGGCGACGAC	2290, 4617	11

550	16	GCGGCGGCGGC	2193, 4500	11
551	16	GGCCGGATTGC	231, 2039	11
552	16	TCGACCGCCAG	3446, 3939	11
553	16	TCTTTCCGCGC	2245, 2963	11
554	16	ACAACGGAAT	557, 3074	10
555	16	AGCGCACCGT	3762, 4407	10
556	16	CACCGCCGCC	759, 1205	10
557	16	CCACCATCGA	65, 1139	10
558	16	CCCCAAGGCC	75, 735	10
559	16	CCCGCCGGGC	1224, 2837	10
560	16	CGAGATCGGC	1065, 4435	10
561	16	CGAGGTCGTG	1856, 3030	10
562	16	CGCCGACAAC	747, 1358	10
563	16	CGCCGTCGCG	3591, 4201	10
564	16	CGCGACCAGC	495, 4555	10
565	16	CGTGCCCGAG	2897, 3345	10
566	16	CTGCCCGAGC	1373, 2670	10
567	16	GCCGGCGCCG	1089, 3846	10
568	16	GCGCAGGCCG	2063, 3460	10
569	16	GCGGCGCCCG	4547, 4696	10
570	16	GCTCAAGGGC	444, 1026	10
571	16	GCTGTTCCTG	2126, 4279	10
572	16	GGATGGTCGG	881, 2256	10
573	16	GGGCACCGCC	924, 3046	10
574	16	GTCCGAGGAC	528, 708	10
575	16	TCACCACCAC	59, 2686	10
576	17	CGGCGATCGAGG	412, 2839	12
577	17	CCGAGCAGCGC	2336, 2829	11
578	17	CGTCGTCGCCG	500, 1229	11
579	17	GACGCCATCGC	1318, 1759	11
580	17	AGCTCGAGGT	2243, 2291	10
581	17	AGGTCGACGT	619, 3759	10
582	17	AGTCGTCGAA	3583, 3904	10
583	17	CCCGGTGGCG	593, 2723	10
584	17	CGCCGCCAAC	3722, 4491	10
585	17	CGCCGGATCG	1474, 3449	10
586	17	CGCGCACCAG	1371, 2523	10
587	17	CGCGGCGGAT	455, 2069	10
588	17	CGCGGGCGCC	1623, 4562	10
589	17	CGGATGAGTG	1161, 2038	10
590	17	GCGCTGCTGC	24, 141	10
591	17	GGCCACCAGA	2811, 4412	10
592	17	GGCGCGGTAG	164, 4334	10
593	17	GGCGCGGTGA	1412, 2559	10
594	17	GGCGGCCAGG	1863, 3230	10
595	17	GGTGGTCGAC	1653, 2190	10
596	17	GTATCTGCAC	930, 1494	10
597	17	TCGCTGAGCT	3789, 3990	10
598	18	GGCCGTCGGCCAGG	1674, 2031	14
599	18	ACCACCGCGTCGA	2315, 3535	13
600	18	CGTGATCAACAGC	741, 4415	13
601	18	GACGACGCGTCCG	2113, 4157	13
602	18	AGCCCGGCGTGT	3125, 4203	12
603	18	AGGATGCGGCGA	2006, 4366	12
604	18	CCGCCGCGGCGA	47, 3830	12

605	18	CCTGGGCGACCA	958, 1485	12
606	18	CGGCCGCCGCGG	44, 1464	12
607	18	CGGTGACGACGC	4153, 4477	12
608	18	GCCGCGGGCGGC	1756, 3366	12
609	18	GCGCCCCCGCCG	452, 2300	12
610	18	CCAGCGCGGCG	3737, 4000	11
611	18	CCATGCCGGAG	1548, 2565	11
612	18	GATGCCGATGG	1882, 4064	11
613	18	TCGATCACGAC	583, 2240	11
614	18	TCGCGATCACC	973, 1222	11
615	18	TGAGCAGCATG	167, 209	11
616	18	ACGACGGTGC	656, 1077	10
617	18	AGATCAGGTC	1815, 3500	10
618	18	ATCGCGATCA	646, 1221	10
619	18	ATGAGGTCGG	3589, 4470	10
620	18	CACCAGAGCC	1265, 2506	10
621	18	CACCAGGCCG	4055, 4139	10
622	18	CCAGTCCCCG	2821, 3342	10
623	18	CGACCGCGAG	1644, 2626	10
624	18	CGAGCGGCGG	139, 2254	10
625	18	CGGCCATGCC	1545, 3948	10
626	18	CTCGTCGAGG	1055, 3429	10
627	18	GATCATCGCC	4112, 4445	10
628	18	GCCACGATCT	349, 529	10
629	18	GCGGCCACCA	3979, 4398	10
630	18	GGCCGGTGGC	1869, 1992	10
631	18	TCGTCGTCGA	3580, 3915	10
632	18	TGCGCCGGCC	1447, 1459	10
633	19	ACGGGGTCAAG	1259, 1886	11
634	19	AGCAGCGGGAT	2397, 3399	11
635	19	CGAGGCGACCG	954, 4517	11
636	19	CTCGTCGAGGT	2219, 3876	11
637	19	GATGACGCCGA	1395, 3663	11
638	19	GGTGATGGTGT	111, 411	11
639	19	TCGGCGGGGGT	658, 769	11
640	19	TGTCACGCACC	1526, 2062	11
641	19	AGCCGGTCGC	4377, 4488	10
642	19	CCCACGACGG	829, 3987	10
643	19	CGCGGCGCCC	3980, 4007	10
644	19	CGCGTCGCCG	1728, 1861	10
645	19	CGGTGGCTAC	2543, 2983	10
646	19	GCCTGGGTGC	49, 61	10
647	19	GCGCGGCGCC	2769, 3979	10
648	19	GCGGATGCGG	2348, 3767	10
649	19	GTCAGCCCGG	1093, 3570	10
650	20	TCTGCGTCGGCT	1404, 3980	12
651	20	CAGTACCTCGA	637, 1838	11
652	20	CGCGGCCATCG	1484, 2412	11
653	20	CGTCGCGACCG	2021, 3810	11
654	20	CTTCGAGGACC	819, 3906	11
655	20	GCCGGCGCCGG	1562, 3643	11
656	20	GGCCAAGGGCG	912, 3126	11
657	20	ACGCCGCCCT	4287, 4449	10
658	20	ACGGCGACGA	602, 4371	10
659	20	ATCCTGGTGA	2398, 4436	10
660	20	ATCGACACCC	2704, 4514	10

661	20	ATCGTGTCGC	928, 3226	10
662	20	ATGTACAGCG	557, 1266	10
663	20	ATTCGGCCAG	1756, 1912	10
664	20	CCCGCCGTCC	1828, 1879	10
665	20	CGACGTATCG	2328, 3005	10
666	20	CGCGCCATCG	3220, 3259	10
667	20	CGTCGACAAG	3468, 4672	10
668	20	GATCGCCTCG	958, 1170	10
669	20	GATGCTCGCC	3378, 3873	10
670	20	GCGCCGCAGG	1437, 4332	10
671	20	GCGCTTCGAG	3774, 3903	10
672	20	GCTACGTCGC	3806, 4545	10
673	20	GGCCAACAGG	1553, 2575	10
674	20	GTCCCCGCCG	1132, 2530	10
675	20	GTCGTCGCCG	2278, 3637	10
676	20	GTGCGTAGTC	429, 450	10
677	20	GTGGCCTTCA	1860, 4601	10
678	20	TCGCGACGAA	3021, 3891	10
679	20	TCGGCGGCCG	1851, 2566	10
680	21	CGGCGGATCGCC	774, 3447	12
681	21	GGGGCGCCGTCG	1086, 3816	12
682	21	CCCCACGCGGC	2683, 3561	11
683	21	CGCCGACTGGG	2362, 3241	11
684	21	CGCGGCGATCT	605, 2620	11
685	21	GCAGCCCTCCG	2023, 4720	11
686	21	GCATCGAACTC	118, 2906	11
687	21	GCCGTGCGCGC	2304, 2673	11
688	21	TCGGCAACTGG	1150, 1547	11
689	21	TCGTCAACGTC	3189, 3336	11
690	21	TGGGCGGTCTT	363, 441	11
691	21	ACACCAGATG	1253, 3208	10
692	21	ACGGGCGCGT	1526, 2975	10
693	21	CCAGGCGGTC	4390, 4705	10
694	21	CCGACGCCGA	1780, 3237	10
695	21	CCGGCGGCCG	843, 3998	10
696	21	CGACGGGCAC	4263, 4632	10
697	21	CGCGCTGGGC	2722, 4300	10
698	21	CTGGGCGCTG	4432, 4603	10
699	21	GACGCTCATC	137, 2597	10
700	21	GCTCTTGCCG	545, 1814	10
701	21	GGCACCGCGG	271, 1341	10
702	21	GGCCGGCAAC	189, 1135	10
703	21	GGTCGAGCAG	913, 3148	10
704	21	GTGCGCGCCG	1473, 2007	10
705	21	TCTGGGCGCT	91, 4431	10
706	21	TGCGCACCGC	1974, 4581	10
707	21	TGCGCCGCCA	3392, 3720	10
708	21	TGGCCGGCGT	2561, 3069	10
709	21	TTCTGCGGGG	2847, 3810	10
710	22	CCAACGTGCGG	3083, 3184	11
711	22	CCGACGGGCGC	4118, 4509	11
712	22	CGCCGTGCGGA	809, 4498	11
713	22	CGGCACACCAA	292, 1232	11
714	22	GGCGGCGACGC	3828, 4455	11
715	22	ACCGTCTTCG	944, 1037	10

716	22	ACGCGGCGGC	2097, 3824	10
717	22	AGGTGATCGA	1515, 3211	10
718	22	ATCCCGCGGC	262, 3673	10
719	22	CCTACGCGGC	1694, 3821	10
720	22	CGAGGACGCG	755, 4661	10
721	22	CGCCCGCACC	3329, 3536	10
722	22	CGCCGTTGTG	2023, 2798	10
723	22	CGCGGTCGCC	889, 3530	10
724	22	GACCGCCACG	686, 1151	10
725	22	GCGGCTGGTC	1566, 2271	10
726	22	GCGGGCGTCG	87, 1745	10
727	22	GCTGTTGATC	1996, 2343	10
728	22	GGCGATCCCG	258, 4241	10
729	22	GTCCTTCGAG	4089, 4718	10
730	22	TATGTGCGGG	3057, 4698	10
731	23	GTTGTGGGACCA	482, 1019	12
732	23	CACCGCCGGCG	4176, 4206	11
733	23	CATCGGCCGTC	1477, 3487	11
734	23	CGCCAGAATCA	1881, 2750	11
735	23	ACATCGCCTG	4136, 4490	10
736	23	ATCGGGCCGT	413, 528	10
737	23	ATGACCGCCC	471, 3090	10
738	23	CAACGCTGCC	20, 243	10
739	23	CGCCGCCGCC	1635, 3605	10
740	23	CGCCTCGCTG	3191, 3458	10
741	23	CGGCCTCCGC	2033, 3965	10
742	23	CGTCGGCATC	1100, 1175	10
743	23	CGTGGCGCTG	614, 3626	10
744	23	GATCATCTCG	2543, 4278	10
745	23	GATCGTCGCG	893, 3755	10
746	23	GCCGTCGACC	1164, 2376	10
747	23	GGCAACTTCT	1386, 2058	10
748	23	GGCTTCACCC	277, 2352	10
749	23	GGTGCTGATG	3476, 3890	10
750	24	CCGGCGGCCTACGGC	299, 1439	15
751	24	CGCCGTCGCGGCCG	586, 3768	14
752	24	CGGCACCGGCGCC	1383, 4467	13
753	24	GGTGCAGCAGACC	2004, 2154	13
754	24	ATCCTGGCCGCC	578, 4270	12
755	24	CATCCTGGCCGC	532, 577	12
756	24	CGCATCGGTGAC	1774, 1902	12
757	24	GCCGAGCTGACG	209, 3970	12
758	24	ACCAGCTGATC	888, 2411	11
759	24	ACGCCGGCGCG	33, 630	11
760	24	CCACGGTCTCC	699, 1572	11
761	24	CCCGGTGGCGC	1555, 3589	11
762	24	CGTTCATCGTC	1859, 2606	11
763	24	CTCGACGAGCT	656, 4282	11
764	24	GCGCCGCCGGC	1376, 3875	11
765	24	GCTCGACAAGG	3469, 4701	11
766	24	GGCTCAACGAC	183, 2588	11
767	24	TCCGCAACGGG	453, 4559	11
768	24	TCGACGATCAC	2167, 3272	11
769	24	AAGGTACGCG	1630, 4405	10
770	24	ACACCCCGCG	1340, 2672	10
771	24	ACACGGTCGC	1895, 4571	10

772	24	ACCCGATGGC	1234, 2717	10
773	24	ACGCTGCGGC	2876, 3341	10
774	24	AGATCCTGGC	4268, 4445	10
775	24	CATGCTCGAC	3499, 4161	10
776	24	CCGGGATCGA	2907, 3071	10
777	24	CGAAGACGCC	3142, 4127	10
778	24	CGATCACCAC	2645, 4025	10
779	24	CGCCGACGAC	2088, 2385	10
780	24	CGGCGTCGGT	1524, 2830	10
781	24	CTCGTCGACG	425, 755	10
782	24	CTGGTGGCCC	107, 3407	10
783	24	GACAACGTCG	287, 2981	10
784	24	GCGGCCGCGC	1430, 4384	10
785	24	GCTGGGCAAG	2992, 3205	10
786	24	GGCCCGGTGG	1463, 1553	10
787	24	GGCCGAGCTG	1116, 3969	10
788	24	GGCGATCGCC	1668, 2379	10
789	24	GGCGGCGGCC	3792, 4204	10
790	24	GGGCCCGCCC	1258, 1417	10
791	25	CCCGGCCGGGCGGC	2440, 4054	14
792	25	CTCAGCGCGCTCA	3967, 4432	13
793	25	GCCCCCCGGCCGG	2436, 2467	13
794	25	CCGGTGCTGCCG	1935, 2550	12
795	25	CGCGATGTCGTC	2105, 4342	12
796	25	CGCGGCGGTGGC	3246, 3258	12
797	25	GCCGACGCCGAT	1794, 4408	12
798	25	TCGTCAACGGCG	557, 593	12
799	25	TGCTGGCCAACC	1522, 2161	12
800	25	TGTCGGCGGCCA	3162, 4631	12
801	25	ACCGTTCCTGT	1721, 2372	11
802	25	CAATGTCGGCG	2030, 3159	11
803	25	CCCAGGTGCTG	1783, 2873	11
804	25	CCCGCGCCGCC	509, 4214	11
805	25	CCCGGTGCTGC	1934, 3981	11
806	25	CCGTCAGCATG	1951, 4256	11
807	25	CGGAGCAGATC	692, 4544	11
808	25	CGGCCGCGCTG	545, 4298	11
809	25	CTACCACGCCG	3585, 4248	11
810	25	GACCGCGCCGC	3282, 3745	11
811	25	GCGATCCGCGA	399, 3448	11
812	25	GGCGCCTGCAG	1190, 4568	11
813	25	GGTCGACCAGA	2904, 3084	11
814	25	TGATCGACACC	1330, 4454	11
815	25	AACGCCGATG	814, 1227	10
816	25	ACAGCGGACC	1714, 1754	10
817	25	ACATCGCGGC	4271, 4609	10
818	25	CACGATGGAC	1925, 3150	10
819	25	CATCGTCAAC	591, 4467	10
820	25	CCGCGATCGA	2858, 3038	10
821	25	CGACCCGGCA	857, 1119	10
822	25	CGACCTCGGC	2483, 3324	10
823	25	CGCCCGACGA	2663, 3848	10
824	25	CGGACTACCC	2359, 3875	10
825	25	CGGCCTGGCC	174, 1430	10
826	25	CGTGTTCTCC	237, 3813	10

827	25	CTGCGCATCA	349, 1458	10
828	25	GACCGCCGAC	2294, 2619	10
829	25	GATCAACCGG	648, 4560	10
830	25	GCCGGCACCG	195, 889	10
831	25	GCCGGCCACC	501, 1371	10
832	25	GCTCAACGCC	228, 678	10
833	25	GGTGACCACC	339, 2174	10
834	25	TCCCGCTGTC	1637, 1774	10
835	26	CGGGACCGGAACTGGG	4510, 4534	16
836	26	CCTGGGTGGCGGC	1459, 3581	13
837	26	CCACGATCGTCG	1484, 3164	12
838	26	CGAGCGCGGCGG	2707, 3670	12
839	26	GCCGCCGCCCCG	3436, 3720	12
840	26	GGCCGCGGCGGC	3363, 3818	12
841	26	GGGGACCGGCAC	4468, 4498	12
842	26	CCCCGCGGCCG	1427, 3108	11
843	26	CCGGGCCGGGC	3851, 4008	11
844	26	CCTGGCCGCGA	3659, 4249	11
845	26	CGCGGCGGGCA	1361, 4030	11
846	26	GCCGCCGCGGT	131, 3780	11
847	26	GGTGTCGAGCG	2016, 3513	11
848	26	CCAGTTCGGT	1250, 3467	10
849	26	CCCGACCGCG	1868, 4126	10
850	26	CCGCCGCCGC	2474, 3386	10
851	26	CGGTGCTGCT	381, 1105	10
852	26	CTGATCGTCG	1002, 2699	10
853	26	CTGCCCGAGT	365, 4268	10
854	26	GACCTGGGTG	1457, 3507	10
855	26	GAGCTGCTGC	59, 2864	10
856	26	GCACGCCGCG	484, 2951	10
857	26	GCCGTACCTG	995, 4159	10
858	26	GCGCCGAGGC	2464, 4197	10
859	26	GCGCCGGCGG	2452, 3488	10
860	26	GGTACGCCGT	474, 990	10
861	26	GTTGTCGGTG	1564, 2535	10
862	27	CGGCGAAGGCCTG	887, 1666	13
863	27	CGGTGACGCTGCA	2848, 4621	13
864	27	GCAGGGCGCCGCG	491, 4127	13
865	27	CGTCATCGTCGC	2777, 3521	12
866	27	GCGCCGCCCGCG	357, 551	12
867	27	ATCACCAGCGT	4038, 4413	11
868	27	CCATCACCAGC	133, 4411	11
869	27	CGGCCCCGGCG	199, 328	11
870	27	CGGTGGTGTTC	2569, 3910	11
871	27	CTTGCCGGCCT	1368, 4314	11
872	27	TCATCACGGTC	3571, 4021	11
873	27	TGGCCGGCATC	3670, 3859	11
874	27	AAAGCTCGGC	1756, 3733	10
875	27	AGCAGGGCGT	2376, 3295	10
876	27	CACGTCGGTG	314, 4616	10
877	27	CCGTCATCGT	3520, 4471	10
878	27	CCTGGGCCGC	2648, 3165	10
879	27	CGCCGGACTG	155, 3509	10
880	27	CGTCGCGCAG	983, 1109	10
881	27	CTGGCGCTGG	1213, 3836	10
882	27	CTGGGCGGCG	1661, 3717	10

883	27	GATCTCGCCG	3641, 4389	10
884	27	GCGATCTCGG	1390, 3903	10
885	27	GGCGATCAGC	741, 4670	10
886	27	GGCGATCTCG	3638, 3902	10
887	27	GTCGCGCCGC	354, 804	10
888	27	GTTGCCCTCG	861, 1431	10
889	27	TCCGGCGCGG	615, 3848	10
890	27	TCGCCGGTGT	1743, 4174	10
891	28	GCACAGCGCGCC	2162, 3450	12
892	28	GGTCGGCGAGCT	1062, 3128	12
893	28	CCGGCGCGGTC	336, 2951	11
894	28	CGACGTGCGCG	135, 1170	11
895	28	CGGCGGCGAAG	1289, 2180	11
896	28	GAGCAGGATGA	2868, 4461	11
897	28	GGCCCGGCGCG	333, 1132	11
898	28	GTCGTAGGGGC	778, 4119	11
899	28	TCCCGGCGCCG	2007, 2355	11
900	28	CCATGTCGTC	2999, 3302	10
901	28	CCGCCGGGCG	1198, 1902	10
902	28	CCGCGCCGGT	453, 2135	10
903	28	CGCTCGGCGT	872, 3619	10
904	28	CGGCGCTGAC	1993, 3962	10
905	28	CGGCGCTGGG	1358, 4498	10
906	28	CGGTCGCCGA	1346, 4351	10
907	28	GACCGGCGCG	372, 2949	10
908	28	GCAGGTCGAC	636, 4541	10
909	28	GCCCCGGTGC	796, 3887	10
910	28	GCCGGTGCTG	1971, 3017	10
911	28	GCGCCGTCGG	1986, 3739	10
912	28	GCGCGTAGGA	884, 4600	10
913	28	GCGTCGAGCA	2291, 4456	10
914	28	GGAGCCGACG	1074, 1165	10
915	28	GGCCGCTGGG	425, 1423	10
916	28	GGTGCCGTGG	2901, 3855	10
917	28	GTCGACGTGC	133, 1627	10
918	28	GTCGATCAGG	1747, 3435	10
919	28	GTCGGCGGCG	208, 1777	10
920	29	CGAGCAGCGCGGCC	609, 4257	14
921	29	ACCGCCGATCCG	3493, 4195	12
922	29	TGATGGCGCCGG	2075, 3126	12
923	29	ATCGTCGAGAT	3455, 3940	11
924	29	CCGGCAGCGCG	2513, 4050	11
925	29	CCTCGTCGTCG	264, 4398	11
926	29	CGACGAGCTCG	288, 812	11
927	29	GACGCCGCCGA	2948, 4684	11
928	29	GACGGCCCGGC	727, 2300	11
929	29	GCACCGCCGAT	11, 4193	11
930	29	GCGCCCTCGCG	1300, 1611	11
931	29	GGCCGTCGAGG	3298, 3675	11
932	29	TCGTCGCCGCG	143, 2982	11
933	29	TGACGCCGGCG	1868, 4125	11
934	29	ACATCAGGCC	2015, 2423	10
935	29	ACGGCGCCTC	156, 1559	10
936	29	CAGGCTGCCG	912, 4558	10
937	29	CCAGGCTGCC	594, 911	10

938	29	CCGCGGTCGT	441, 3992	10
939	29	CGATGTTGAA	3771, 3834	10
940	29	CGCCGCCTGG	709, 870	10
941	29	CGGTGCGGCC	1952, 4317	10
942	29	GACGACCTCC	834, 1635	10
943	29	GGTACTTCGT	3102, 4476	10
944	29	GGTAGCCGGG	1139, 3801	10
945	29	GTCCTCGTCG	262, 3386	10
946	29	TCCTCCGGGG	2311, 2797	10
947	30	GGCGCTGGCAACCGC	4190, 4506	15
948	30	ACACCTGGGCG	1466, 3493	11
949	30	AGATCAGCATG	362, 854	11
950	30	AGCCGCAGGAA	970, 3760	11
951	30	CAAGGTCGACC	2936, 3167	11
952	30	CACCGTCATCA	1302, 4091	11
953	30	CGCCGCCCACC	152, 2113	11
954	30	CTGCTCGACGA	873, 1161	11
955	30	GATGCCGCCGA	432, 591	11
956	30	GCCGGCCCGGT	520, 4489	11
957	30	GCGCCGACGTA	1183, 2598	11
958	30	GTCGGCGAGCG	246, 2290	11
959	30	ACGTCGTCGG	241, 3049	10
960	30	ATCAGCAGGC	637, 3076	10
961	30	ATCGTGGTCG	1477, 2386	10
962	30	CAGCCGCAGG	780, 969	10
963	30	CCTGCTCGAC	1160, 1917	10
964	30	CGATCAGCAG	557, 635	10
965	30	CGGCGTGATG	426, 3266	10
966	30	GACGAACGCC	1395, 2486	10
967	30	GATCGGCGCC	1242, 3188	10
968	30	GCGCGAGGAC	1226, 2900	10
969	30	GCGGACGTCG	237, 4567	10
970	30	GCGGCGAAGG	3858, 4582	10
971	30	GCGGCGATCG	1702, 2708	10
972	30	GGCGTCCTCG	1044, 2215	10
973	30	GTCGGCGACC	819, 1569	10
974	30	TCAAGACCGC	3235, 4559	10
975	30	TCCCGCTCGG	265, 1718	10
976	30	TGCAGCCCGG	1066, 4745	10
977	30	TGGCGCCCGA	1339, 2000	10
978	31	GCGCGCCCGCAA	840, 3851	12
979	31	GGGATCGTCGGG	681, 4601	12
980	31	AGCGATGCGAC	2433, 4269	11
981	31	CCAGTTCGACC	1975, 4738	11
982	31	CCGACCCGGTC	1602, 3798	11
983	31	CCGGTCGACGG	1866, 2667	11
984	31	CGACGACTTCG	2928, 4672	11
985	31	CGCGCAGTGGG	1469, 3024	11
986	31	GCGCCGCCACC	818, 2119	11
987	31	GGCCGCCTCGA	1491, 2096	11
988	31	TCGGGCGCGAA	907, 1036	11
989	31	ACGAGCGTCG	1246, 2851	10
990	31	AGGCGGCCGC	1646, 4567	10
991	31	CACGCCGCCG	877, 1281	10
992	31	CAGCCCGGTG	2270, 4456	10
993	31	CCAGGGCAGG	693, 2234	10

994	31	CCGCGCCCAT	1662, 3887	10
995	31	CCGGTGGCCG	1486, 3511	10
996	31	CGACCGGTTG	1764, 3657	10
997	31	CGCCACGACC	1857, 2364	10
998	31	CGCCGACAGG	381, 3701	10
999	31	CGCCGCCGCC	603, 2952	10
1000	31	CGCGGCCGGC	1379, 3297	10
1001	31	CGTCACCGCG	629, 1050	10
1002	31	CTCCTCCTCG	28, 3755	10
1003	31	GACTCGCGCA	164, 3915	10
1004	31	GCCCAGCGGC	957, 3565	10
1005	31	GCCCTCGACG	2893, 3234	10
1006	31	GCGCCCGGGT	54, 4613	10
1007	31	GCGGCCAGGG	134, 673	10
1008	31	GCGTTCAAGC	3196, 4466	10
1009	31	GCTGGGACTG	1580, 1841	10
1010	31	GGCCCGCGGG	2180, 3149	10
1011	31	GGCGCGCCCG	51, 839	10
1012	31	GGTCGGCCCT	1944, 3625	10
1013	31	TCGCTGATGC	3068, 4370	10
1014	31	TGCCCGCCGC	557, 599	10
1015	32	GCGGCGCCGGCCGGGCC	1700, 1885	17
1016	32	CCGCGCCGGCGGTC	1445, 1871	14
1017	32	AACGCCAGCACGC	2230, 2929	13
1018	32	GCACGGCGCGCCG	3138, 4144	13
1019	32	CAGCACGCGGTC	3246, 4205	12
1020	32	CCAGCACGACGA	1760, 3692	12
1021	32	CCGCCGGTCCCG	1670, 4545	12
1022	32	ACGCGCGGCCG	2078, 2340	11
1023	32	AGGACGGCGCC	897, 3924	11
1024	32	CCGAGGCGTAT	150, 1607	11
1025	32	CGCCACCGTCC	2010, 2952	11
1026	32	CGGCCCGCAGG	331, 850	11
1027	32	CGGGCGCGGAC	2414, 3125	11
1028	32	CGGTCGGTGAC	2785, 3969	11
1029	32	CGTGCACGGCG	4126, 4378	11
1030	32	GCCAGCCCGGC	3652, 3879	11
1031	32	GCGTCGTCGCG	1406, 3284	11
1032	32	GGCCGCCGCGA	3492, 3732	11
1033	32	GGCGACGCGGC	2697, 3333	11
1034	32	GTGACGACGTA	1255, 2713	11
1035	32	TCAAGCTCATC	507, 1053	11
1036	32	TGACCGCGATG	261, 4039	11
1037	32	CAGCGCCGCC	1170, 4085	10
1038	32	CCCGCGCCGA	3553, 3754	10
1039	32	CCGACCGCGG	1381, 1429	10
1040	32	CCGCACGCCC	247, 4424	10
1041	32	CCGCCAGCGC	1556, 2042	10
1042	32	CCGCCCGCGC	3681, 4573	10
1043	32	CCGCGCCAGC	2874, 3648	10
1044	32	CCGCGCCGCG	3158, 3712	10
1045	32	CCGCGCTGGG	555, 687	10
1046	32	CCGGCGGCGA	2579, 3517	10
1047	32	CCGGGCGGGC	666, 4465	10
1048	32	CGATCGGCCG	378, 3840	10

1049	32	CGATGGCGAA	1847, 3997	10
1050	32	CGCGCTCGGC	178, 2278	10
1051	32	CGGCGACGGC	1742, 1778	10
1052	32	CGTCGCCACC	4060, 4664	10
1053	32	GAACGACACC	1499, 1788	10
1054	32	GCGGCCGCTG	2166, 2292	10
1055	32	GGTGGCGGCC	2028, 2259	10
1056	33	CGTCGGCGGTGCCGA	65, 1172	15
1057	33	TCCCCGGCCGGCGG	270, 2225	14
1058	33	CGAGGCGGTGGAG	2975, 2993	13
1059	33	GGTGCGCACCGCG	1910, 2618	13
1060	33	GTTCGCGGCGGCG	195, 4275	13
1061	33	CTGGTGCCCTCG	373, 3354	12
1062	33	GCCCAGCAGGCC	2910, 3618	12
1063	33	GGCCTCGGCGGC	4209, 4660	12
1064	33	CCGCGGCCTCG	1996, 4205	11
1065	33	CGACGTCGGCC	1454, 4626	11
1066	33	CTCGGGCAGCC	852, 1191	11
1067	33	CTGGACCGCGC	3300, 4564	11
1068	33	GCCACGGTGTT	1406, 3543	11
1069	33	GCGCCAGGCCC	932, 2056	11
1070	33	GGCTCGCCGGC	3694, 4704	11
1071	33	GTCCTGCTGGG	2460, 4228	11
1072	33	GTTCGTCGGCG	1169, 1765	11
1073	33	TCAGCAGCCTG	2536, 2554	11
1074	33	TCGGTGATGAC	1794, 2442	11
1075	33	ACCCGCGTCG	1128, 1281	10
1076	33	ACCGTCAGCA	1312, 3468	10
1077	33	CCGCCGCGGC	1241, 1705	10
1078	33	CCTTCGCGGC	1382, 2107	10
1079	33	CGAGTCGGCG	3056, 4176	10
1080	33	CGCCCAGCAG	2656, 3617	10
1081	33	CGCGGGCCGC	286, 1960	10
1082	33	GCCATCGCGC	724, 2282	10
1083	33	GCTGCCGATG	2390, 4242	10
1084	33	GCTGGTCGTC	2334, 2453	10
1085	33	GTCGAGCACC	225, 870	10
1086	33	GTGCGGCGCG	2248, 4296	10
1087	33	TCACCGAGCT	3289, 4391	10
1088	33	TCCTCGCTGT	217, 1869	10
1089	33	TCGCCTCGTC	467, 1082	10
1090	33	TGGTCGCCCA	1040, 3613	10
1091	34	ACTTCGGGTCGAT	136, 4649	13
1092	34	GATCGTCGACACC	2409, 3322	13
1093	34	GCAGATGATGCAG	452, 494	13
1094	34	CCGCCGCCGATC	222, 2303	12
1095	34	CGGCGGCGGTGG	274, 4229	12
1096	34	GGGCGGGGCGAC	548, 1738	12
1097	34	CCCGGATTCGG	2785, 3089	11
1098	34	CCGCCGGCGCC	1901, 4355	11
1099	34	CGCGCTGCAGG	1460, 2343	11
1100	34	GGGTCAGGCCG	398, 4634	11
1101	34	GGTCATCGCGA	4155, 4263	11
1102	34	TGCGCGCCGCC	322, 4366	11
1103	34	ACGACGACGG	756, 1756	10
1104	34	ATCACCGCGG	3407, 3674	10

1105	34	CACCGACCAG	1604, 3508	10
1106	34	CCCGCCTGGC	1192, 3127	10
1107	34	CCGCTGGTGC	84, 3260	10
1108	34	CCGGATGGCG	2853, 4289	10
1109	34	CCGGCCGGTG	695, 714	10
1110	34	CCGGTCAGCG	663, 4717	10
1111	34	CCGGTGACGC	1114, 4117	10
1112	34	CGCCACCGAC	62, 1391	10
1113	34	CGCCGTGGTG	4410, 4485	10
1114	34	CGCGGTGGTC	4335, 4473	10
1115	34	CGGCCACGGC	1280, 1481	10
1116	34	CGGCGCGACC	1969, 3568	10
1117	34	CGGCGTCCTG	4251, 4503	10
1118	34	CGTCCGGATG	4527, 4596	10
1119	34	CTCACGGCCT	192, 3020	10
1120	34	CTCGCGCAGC	2490, 3481	10
1121	34	CTTCGCCACC	1388, 2040	10
1122	34	GCACGGCCAG	1562, 2102	10
1123	34	GCGGTCTCGG	573, 4168	10
1124	34	GCTGCGGCGC	1965, 3298	10
1125	34	GGCCGGCAAC	1920, 3229	10
1126	34	GGCGGCGCCG	254, 2175	10
1127	34	GGGCGCCCAG	338, 380	10
1128	34	TGCAGGCGAT	472, 1784	10
1129	34	TGCATCGAAA	39, 2437	10
1130	34	TGCGGCCGAT	1829, 2186	10
1131	34	TGTGCGGCGT	2159, 4499	10
1132	34	TTCTTCGAGG	1500, 3835	10
1133	35	CGACCGGCTGCTGG	3236, 4230	14
1134	35	GATCAGCGTCGTCA	2564, 3404	14
1135	35	CCGCCACCGGTCG	967, 991	13
1136	35	GGCGGCGGCCCTG	454, 3951	13
1137	35	CGCCACCGCCGC	1073, 1107	12
1138	35	CGCGATCGCCGC	22, 2106	12
1139	35	TCGCGCTGGTGT	222, 2272	12
1140	35	AGGTGATCAAC	1364, 1862	11
1141	35	CAACATCGCCA	717, 2055	11
1142	35	CCCCGCCGCCG	1031, 1085	11
1143	35	CCGCGGCGGCC	1397, 4667	11
1144	35	CCGCGGGGAAG	2888, 3664	11
1145	35	CCGGGCGCGCA	1990, 2046	11
1146	35	CGACGCCGACG	3984, 4512	11
1147	35	CGCGGTGATCG	765, 1464	11
1148	35	CGGCGTCGCCG	1674, 2414	11
1149	35	GACCGCGTCGA	4468, 4527	11
1150	35	GCCCTCGGTGG	576, 3641	11
1151	35	GCTGCCCGCCG	91, 3170	11
1152	35	GGTCACCGACG	3254, 4713	11
1153	35	GTCGCGGCGCT	1816, 3856	11
1154	35	GTTCGTCGCCG	653, 3969	11
1155	35	ACATCATGGG	827, 1697	10
1156	35	AGGGCGGCGG	1097, 1718	10
1157	35	ATCACCCAGG	182, 1051	10
1158	35	CAGGGCCGGG	3943, 4441	10
1159	35	CATCACGGTG	2996, 3032	10

1160	35	CCCCCGACGG	602, 3496	10
1161	35	CCGAGATCGG	2629, 3190	10
1162	35	CCGCGCACCA	1853, 3435	10
1163	35	CCGCGTTGAC	1949, 4196	10
1164	35	CCGGCGCAGC	2865, 3158	10
1165	35	CGACCGTTGG	2725, 4746	10
1166	35	CGCCCACCCC	3058, 3538	10
1167	35	CGCCGCGATC	19, 3527	10
1168	35	CGGCGCCGCG	16, 2243	10
1169	35	CGGTGGCGAC	327, 816	10
1170	35	CGTTGCGGCG	501, 4052	10
1171	35	CTACATCGGC	2237, 4143	10
1172	35	CTACGTCAGC	1803, 3716	10
1173	35	GAACCCGCGG	792, 1153	10
1174	35	GACGGCGTCG	2412, 4392	10
1175	35	GCCGCGGCCC	615, 921	10
1176	35	GCCGGTGGTG	316, 804	10
1177	35	GCGCTGGTGG	146, 2288	10
1178	35	GGACGCGGTG	1461, 2849	10
1179	35	GGCGCCGTCG	1581, 1618	10
1180	35	GGGATGGTGC	592, 4588	10
1181	35	GGGGTGGTGG	1192, 2077	10
1182	35	TCGCGGGCCT	1243, 2764	10
1183	35	TGGCGCCGCC	1019, 1635	10
1184	35	TGGTCACCGA	3184, 4712	10
1185	36	CGACGCCGCGCGC	657, 682	13
1186	36	GCTGGCCGCCGTC	103, 2959	13
1187	36	GCCGGACGTGAC	3082, 4495	12
1188	36	GGAGCGGCCGGC	1637, 2638	12
1189	36	GGAGGTCCTGCG	2713, 4158	12
1190	36	GGGCGGCGTGGG	4320, 4534	12
1191	36	AAGGACGGGGC	2300, 4223	11
1192	36	ACGCCGGTTCG	973, 3759	11
1193	36	ACGCGCGCGCC	1745, 2185	11
1194	36	AGCTGCTGGCC	60, 81	11
1195	36	CCCGGCGCCGC	3013, 4742	11
1196	36	CGCCGACCGGC	43, 1072	11
1197	36	CGGCGCGGGCG	3219, 4620	11
1198	36	CTCGACGACGA	26, 2684	11
1199	36	GCGGGCGGGCC	844, 4353	11
1200	36	GGCGCCACGGC	747, 4199	11
1201	36	GTTCATGGGCT	1370, 4417	11
1202	36	TCGCCGACCCG	3006, 3207	11
1203	36	TCGGCGCGGGC	525, 3218	11
1204	36	ACCGGTGACG	856, 2244	10
1205	36	AGATCGCGCA	639, 3623	10
1206	36	ATCGTCGAAG	2361, 3820	10
1207	36	CACGCCGACC	1070, 4018	10
1208	36	CATCGTCGAA	3658, 3819	10
1209	36	CCACCGCCGA	39, 243	10
1210	36	CCGCAGCGCG	2698, 3706	10
1211	36	CCGCCGCGAT	1760, 4586	10
1212	36	CCGCGTGGCC	3346, 3480	10
1213	36	CCGGCCCGGT	1324, 4557	10
1214	36	CGCAAGCTGC	77, 596	10
1215	36	CGCCGGCGCG	1826, 2021	10

1216	36	CGCTGGGCCG	1877, 4082	10
1217	36	CGGCCGGTTC	583, 775	10
1218	36	CGGTGGAAGG	183, 4702	10
1219	36	CGTCGATCCC	3871, 4610	10
1220	36	GATCGCCGAC	1992, 3205	10
1221	36	GCCGCCCGGG	1188, 1244	10
1222	36	GCCGGGGCGG	3280, 4284	10
1223	36	GCGGAAGACG	986, 2480	10
1224	36	GGCGCCGATG	1656, 4250	10
1225	36	GGCGCGCCGC	3294, 4580	10
1226	36	GGGCGACCAG	2003, 2395	10
1227	36	GTCGAACGGC	1256, 3406	10
1228	36	GTCGATGACC	3376, 3673	10
1229	36	TCGTCGCGCT	486, 4406	10
1230	37	CAAGGCGCTCGAC	3883, 4021	13
1231	37	GACCTGGACCCGG	1463, 3935	13
1232	37	CAGCTTCGGCGC	2341, 4219	12
1233	37	CCGGATCGTGGT	3577, 4306	12
1234	37	GGCGCCGAGCCG	440, 1748	12
1235	37	GCCCAGGGCGC	434, 3185	11
1236	37	GCTGGCCGCCG	1573, 2107	11
1237	37	GGCCCACGCCG	1219, 3433	11
1238	37	GGTGGCCGAAG	2086, 3244	11
1239	37	ATCATCGGCG	1364, 4364	10
1240	37	ATCGCCGAAG	614, 3812	10
1241	37	CCGGTGGCCG	2084, 4299	10
1242	37	CGAGCTCGAG	2692, 4633	10
1243	37	CGCAACGTCC	209, 650	10
1244	37	CGCACCGGCC	2786, 4535	10
1245	37	CGCGACCGGA	2261, 3572	10
1246	37	CGGCAAGGGT	2464, 4285	10
1247	37	CGGCGTCGGC	3271, 3400	10
1248	37	CTCGGCGGGC	1202, 4207	10
1249	37	CTGCACCTCG	1196, 3991	10
1250	37	GACACCGCGC	1163, 1526	10
1251	37	GCACGCCGGG	1146, 3505	10
1252	37	GCCCGCGAGG	1076, 2309	10
1253	37	GCCGACGTCG	1118, 2630	10
1254	37	GCCGCAGCCG	372, 1969	10
1255	37	GCGCACCGGC	2785, 3923	10
1256	37	GCGGCCGGCA	2459, 4553	10
1257	37	GCTGCTCAAC	409, 3556	10
1258	37	GGAGCGCCGG	751, 4747	10
1259	37	GGGCTGGCCG	2105, 3530	10
1260	37	TCACCGGCAA	1050, 3771	10
1261	37	TGCTCGACCG	1377, 2397	10
1262	38	GCCGCGGCGGCGG	1498, 3699	13
1263	38	GCCGGCGCCGATC	1539, 2507	13
1264	38	AGGGCGGCCGCC	1489, 2553	12
1265	38	GCGCCCGGCGGG	1896, 1916	12
1266	38	TTCGTTCGGCGC	1610, 4748	12
1267	38	ACGGCATCCAC	860, 4569	11
1268	38	CCTCGACGACG	3455, 3872	11
1269	38	CGACCGGCTGC	3812, 4322	11
1270	38	CGGCGCCGACT	1005, 1395	11

1271	38	GATGCCGCGCG	11, 4585	11
1272	38	GCGAGTCCGGC	2082, 4111	11
1273	38	GCTCACCGAGC	1347, 1368	11
1274	38	GCTCGAGGCGC	2128, 2524	11
1275	38	GGCCATCGCCG	41, 3154	11
1276	38	GTCGGTTCCGG	550, 1413	11
1277	38	CACCGATGCC	4217, 4581	10
1278	38	CATCGGCGGC	636, 987	10
1279	38	CCCAGCTCGA	2466, 2520	10
1280	38	CCGCCGCTCA	575, 952	10
1281	38	CCGGGAATTG	138, 849	10
1282	38	CGCGCGGCGC	1391, 3035	10
1283	38	CGGCGAGCGG	1158, 2008	10
1284	38	CGTCCACCCC	1097, 2660	10
1285	38	CGTGGTGGCC	2630, 3926	10
1286	38	GACGGCTCCG	1270, 3669	10
1287	38	GATGGCCGCG	3238, 3695	10
1288	38	GCCGACCGCA	628, 3215	10
1289	38	GCCGGAGCGG	3779, 4145	10
1290	38	GCTGGAGATC	465, 4187	10
1291	38	GGAGATGATG	2540, 3689	10
1292	38	GGTCGACATC	756, 3863	10
1293	38	GTTCCACCGC	1526, 3589	10
1294	38	TCCCGGCCGG	843, 4140	10
1295	38	TCGACAAGGC	76, 476	10
1296	38	TGCACTCCAA	3652, 3940	10
1297	38	TGCTGCTCGC	3066, 3279	10
1298	39	CCACGCCGCCGCCGA	406, 2266	15
1299	39	CACCACCACGGCG	1586, 2171	13
1300	39	CCGGCGGTCGATG	1688, 4447	13
1301	39	GCGGCGCCGCGC	1402, 2583	12
1302	39	GGGCGCCGTGGC	1445, 4485	12
1303	39	CAACCGCTGGA	3469, 3841	11
1304	39	CCGACGGTCGC	333, 2488	11
1305	39	CCGGCGTGCCG	2029, 4254	11
1306	39	CGAGCGCCGCG	583, 3235	11
1307	39	CGCCTGCACCG	605, 3535	11
1308	39	CGGTCGAGAAC	3396, 3486	11
1309	39	CGGTGGGTGCG	745, 3024	11
1310	39	CGTCGTCACCG	41, 4063	11
1311	39	GATCCGGCGCG	3675, 4531	11
1312	39	GCCCTGGTGAA	1491, 3707	11
1313	39	GCCGGCGCACC	1252, 1846	11
1314	39	GGCTGGCGACC	1927, 2782	11
1315	39	GTCGTCGCCGG	1283, 2990	11
1316	39	TGCGGCCGGTG	1531, 1558	11
1317	39	TTGCCTAGTGC	125, 141	11
1318	39	ACGACGAGGG	3558, 4551	10
1319	39	AGCCGCAGCA	1515, 4383	10
1320	39	AGCGCCGACT	1233, 1755	10
1321	39	CAGCGCTCGC	1637, 3746	10
1322	39	CATCGCGTTC	3856, 4689	10
1323	39	CCGACGTCGA	2058, 3459	10
1324	39	CCGAGCGCCT	3010, 3819	10
1325	39	CCGTTCGGCG	269, 2957	10
1326	39	CGACACCGCG	1358, 2702	10

1327	39	CGCCGACGTC	2056, 2662	10
1328	39	CTTGATGCCG	464, 4744	10
1329	39	GCCCAGCGCC	1751, 4735	10
1330	39	GCCGAGCGCC	581, 3009	10
1331	39	GCCGTGCGCA	1799, 2220	10
1332	39	GCGCGCCGGC	1296, 2561	10
1333	39	GCGGCATCGG	1006, 2685	10
1334	39	GCTGTAGGCG	2147, 4636	10
1335	39	GGATTGGTGC	995, 4727	10
1336	39	GGGGGCACCA	1890, 3602	10
1337	39	GTCACACCGC	2369, 4664	10
1338	39	GTCGATCAAG	1574, 3367	10
1339	39	TGCGGTCGAT	1345, 1570	10
1340	40	TTCTTGGCGGGCGCCTT	631, 676	17
1341	40	GACCCGCTGCGGG	1237, 4488	13
1342	40	GCCGGCGCCGTCG	113, 741	13
1343	40	TCCGCGCGACGCC	4375, 4530	13
1344	40	CGTGCGCCGACG	1299, 1940	12
1345	40	GCAACCCGGGCA	3973, 4210	12
1346	40	TCCGCGCGGCGC	2369, 2867	12
1347	40	CAGCGACGGCG	349, 4022	11
1348	40	CGCCTTGACGT	4647, 4662	11
1349	40	GGCGCCGCGCA	1905, 4518	11
1350	40	GGCGCGCCGCA	2614, 3613	11
1351	40	TCCGCGGAGGT	1058, 3167	11
1352	40	TGGGCGCCGGG	1553, 2315	11
1353	40	ACGCGGCGGA	10, 2465	10
1354	40	AGCTGGCGGA	237, 361	10
1355	40	CCCGCCGGCC	1525, 3625	10
1356	40	CCGCCTTGAG	2508, 3144	10
1357	40	CCTCAGCGAC	346, 2523	10
1358	40	CGCCCACCAG	88, 895	10
1359	40	CGCCGACCCG	1968, 3889	10
1360	40	CGCCGCCTTG	705, 2506	10
1361	40	CGCGATGCGG	2296, 2941	10
1362	40	CGCGCCGGCC	1132, 1995	10
1363	40	GCCGCCCCCG	967, 4002	10
1364	40	GCGCGCCGAG	1809, 2395	10
1365	40	GGCCAGCACC	1255, 4130	10
1366	40	GGCCGCCCGG	2143, 3944	10
1367	40	GGCCGGGAAG	1638, 3792	10
1368	40	GGCGAGCAGC	3225, 3839	10
1369	40	GGGCTCGCCG	1147, 1569	10
1370	40	TCCGCCTTGA	3143, 4660	10
1371	41	CCACCGCCGTCGG	2831, 2846	13
1372	41	ACCTGCTCGCGG	2065, 3923	12
1373	41	CGGCGGCGGGGC	1546, 4147	12
1374	41	GCGTCGGCGGCC	3110, 3290	12
1375	41	GGGGCGGGCGCG	1725, 4059	12
1376	41	TGGCGGACCGCG	1501, 2894	12
1377	41	CCCCAGCGGTC	1347, 4464	11
1378	41	CGGGCTGCCGG	1808, 3346	11
1379	41	GATGTGCGGTT	367, 2374	11
1380	41	GCGGCGCCGCT	2767, 4030	11
1381	41	GCGGCGGCCGA	477, 1099	11

1382	41	GTCGCGCGTCC	2130, 3424	11
1383	41	TGGCGGCCGCG	75, 2086	11
1384	41	TGTCGCCGCCG	159, 3801	11
1385	41	ACCAGCGCCG	3318, 3725	10
1386	41	AGCACGGCCA	594, 3989	10
1387	41	CAGCGGCGCG	2820, 3103	10
1388	41	CCAGCGGCGC	2819, 4027	10
1389	41	CCGCGGCGGC	1097, 1543	10
1390	41	CGCCCAGCCC	49, 3235	10
1391	41	CGCCCCCTCG	1122, 1452	10
1392	41	CGCCGCCGGC	1429, 1526	10
1393	41	CGCCGTGGTG	2718, 3088	10
1394	41	CGCGCCGCCG	1427, 1785	10
1395	41	CGCGGAACCG	96, 4436	10
1396	41	CGCGGGCGCC	903, 1322	10
1397	41	CGGCGCCCCC	1449, 2171	10
1398	41	CGGCGCCCTC	1271, 4424	10
1399	41	CGGTTCACCT	746, 4250	10
1400	41	CTACTGCTGG	2928, 3073	10
1401	41	GCATCGCCGC	1609, 2684	10
1402	41	GCCCGCGGCG	1541, 4481	10
1403	41	GCCCTCGCGG	1686, 4159	10
1404	41	GCCGCAGCAG	4553, 4670	10
1405	41	GCGGTGGGCC	1310, 3218	10
1406	41	GGGCGTGCTC	1706, 1751	10
1407	41	GTCCTGGTCG	3271, 4416	10
1408	41	GTGATTCCAC	653, 3151	10
1409	41	GTGCTGCTGG	1641, 2788	10
1410	41	GTGGTGGATC	340, 1952	10
1411	41	TCGCCGGGCA	2261, 3519	10
1412	42	GCCCGCGTTGCT	150, 4341	12
1413	42	GGCCGGCGGGGA	4085, 4473	12
1414	42	GGCTCGGCGGGC	2674, 3205	12
1415	42	ACGGCGCCGCG	575, 1831	11
1416	42	CCAGCTCGCCA	1571, 3084	11
1417	42	CGCCCGCCGGG	1268, 3879	11
1418	42	CGCGGCGTTGG	552, 3594	11
1419	42	CGGCGGGGGCG	2694, 2745	11
1420	42	CGGGCCGCCCG	989, 1501	11
1421	42	CTCGACAACCA	1788, 1890	11
1422	42	CTGCTGCAGCT	4642, 4657	11
1423	42	GCCGACGCCGC	841, 4240	11
1424	42	GCCGGGCCGCC	987, 4253	11
1425	42	GGGTGGCCGCG	764, 791	11
1426	42	GGTGCTGCGCA	1032, 3771	11
1427	42	GTCCGGCTCGG	2156, 3201	11
1428	42	TCCAGCAGCGC	119, 2418	11
1429	42	TGGCGTCGGGC	2446, 3704	11
1430	42	ACCGGTTCGC	80, 1261	10
1431	42	ACGACGACGC	1184, 1642	10
1432	42	ACGCGCTGCG	273, 4277	10
1433	42	ACGTCGAGCG	8, 1414	10
1434	42	AGGCCGAGGC	1996, 4403	10
1435	42	CATCGCGGCG	1689, 2567	10
1436	42	CCTGCTGCAG	42, 4656	10
1437	42	CGAGGCGGCG	462, 3957	10

1438	42	CGATGCCGAA	810, 2338	10
1439	42	CGCCGTCCGC	1086, 1286	10
1440	42	CGCGCCGCCG	253, 730	10
1441	42	CGCGGGAGGC	2766, 3731	10
1442	42	CGGGCGGTGC	2723, 4109	10
1443	42	CGGTCGCGGA	1654, 2011	10
1444	42	CGTGCCGGTG	1754, 3249	10
1445	42	CTCGACGACC	2387, 4528	10
1446	42	GATGTCGGGC	2952, 4737	10
1447	42	GCACTCCGAC	510, 2168	10
1448	42	GCGCCTGCGC	1775, 2897	10
1449	42	GCGTAGTCGG	104, 2466	10
1450	42	GCTGCTGGGG	2777, 2807	10
1451	42	GCTGCTGTCG	3786, 4230	10
1452	42	GGCCCAGCCC	2306, 3021	10
1453	42	GGCTGCTGTC	3785, 3931	10
1454	42	GGGCCGGCGG	2687, 4084	10
1455	42	TCGGCGGCAT	2858, 2924	10
1456	42	TCTTCGCCGA	674, 3686	10
1457	42	TTCGCCGTCC	1084, 4492	10
1458	43	CTGCACCACGTTCC	430, 1758	14
1459	43	TCCCGATCGAGGGG	4490, 4733	14
1460	43	CCACGCTCAACG	501, 4157	12
1461	43	CCGCCGACGCCG	240, 2710	12
1462	43	CGACGTGCTGGA	3798, 4008	12
1463	43	CGGGTGATCGAC	317, 4543	12
1464	43	GCCAGCATCCGG	1017, 1195	12
1465	43	GGGTGCGCACCG	3046, 4091	12
1466	43	GGTCGGCGACGA	3113, 3552	12
1467	43	CATCGCGCCGA	748, 1904	11
1468	43	CGACGCCGTCA	2888, 3627	11
1469	43	CGAGATCCTCG	91, 4059	11
1470	43	CGCCATTGCGG	801, 2984	11
1471	43	CGCCGCGCTGC	2906, 4182	11
1472	43	CGTTCCGGCCG	1244, 4389	11
1473	43	CTCGGTGCAGT	2849, 3453	11
1474	43	GCATCATCCGG	186, 4589	11
1475	43	GCGCGGGGTCG	3546, 4380	11
1476	43	GGCCGACGCCG	859, 3624	11
1477	43	GGTGCCGTTCG	598, 1312	11
1478	43	TCTTCGGCGCG	2020, 3088	11
1479	43	TGATCCACGTT	1237, 1876	11
1480	43	ACGCCCGCCG	236, 679	10
1481	43	AGATCGCCGC	2704, 2902	10
1482	43	ATCAGTTCGA	201, 3434	10
1483	43	CACCAGCGGC	260, 557	10
1484	43	CACCGACGTG	737, 3795	10
1485	43	CCGCGGCGGT	1125, 3332	10
1486	43	CCGGCCGGGC	2419, 4698	10
1487	43	CGAGGTGCCG	61, 304	10
1488	43	CGCCAGGCCG	2646, 2819	10
1489	43	CGCCGGTAGC	1838, 2154	10
1490	43	CGGCTCGCGC	899, 4564	10
1491	43	CGTCGAACTG	423, 1535	10
1492	43	GACGTCGACG	415, 2883	10

1493	43	GCGGCCCGGC	1820, 3887	10
1494	43	GGCCGGCAGC	157, 1977	10
1495	43	GGTCACCGAC	1259, 3792	10
1496	43	GTTCCCGAGG	584, 3937	10
1497	43	TGACCGCCAG	1096, 2464	10
1498	44	CAGCGCATCTGGACCAAGGACCACCCG	2666, 3437	27
1499	44	CCGATCGTCCCGGTGATCGTGTGGGG	3407, 4189	26
1500	44	ACCCCGAGGCCACCATCAGCCGCAG	2553, 4106	25
1501	44	CTGGGTGCCGCACCGG	2854, 3622	16
1502	44	GGTGCCGATCGTCCCG	2632, 4185	16
1503	44	GCGATCAACCACACC	2324, 3101	15
1504	44	AGGTGCCGATCGTC	2712, 4184	14
1505	44	TTCATGATCAAGGC	3176, 3958	14
1506	44	TCAAGGAGTTCAA	2586, 3357	13
1507	44	TCGATGTGCCCGA	423, 1567	13
1508	44	TTCTGGGTGCCGC	2852, 4402	13
1509	44	CCGGGCGCCCGC	4286, 4695	12
1510	44	GATCCCGGTGGA	3241, 4023	12
1511	44	GGCCGCGCGCAT	3667, 4158	12
1512	44	GGGCGGCGCGCC	2934, 4425	12
1513	44	AACGCGCGCAG	1060, 2811	11
1514	44	ACCGCCCGTCG	569, 3732	11
1515	44	AGGCCGCCGTC	1276, 2508	11
1516	44	CGGCGGCCCGA	141, 3693	11
1517	44	CGGTCAGGCAC	560, 764	11
1518	44	GATCGAGCCGA	400, 2743	11
1519	44	GCAGTTCGTCG	195, 4678	11
1520	44	GGCGCCATCCG	1132, 2158	11
1521	44	GGCGGCCAGCA	187, 1584	11
1522	44	GGGCGCCGCGC	2170, 3373	11
1523	44	GGGGGCGGCGC	3159, 4423	11
1524	44	GGTGAGCTGGT	2534, 3305	11
1525	44	TGCGGTTCATG	2400, 3171	11
1526	44	CATCTGGTCC	1302, 1320	10
1527	44	CCGTGCAGGT	598, 3495	10
1528	44	CTCCGGTGCG	894, 4669	10
1529	44	GACGGGCGCC	1128, 3370	10
1530	44	GCAGGTCAGG	1808, 3199	10
1531	44	GCCCGCTTGG	2022, 2240	10
1532	44	GCCGTCGGCG	738, 1680	10
1533	44	GCGGCGGTGC	2307, 4063	10
1534	44	GGGGCGCCGG	2960, 3255	10
1535	44	GTCCAGCGTG	693, 1944	10
1536	44	GTGGCCGTGC	1287, 3281	10
1537	44	TGCAGCAGGT	1552, 3195	10
1538	44	TGCAGGTGCG	1897, 3516	10
1539	44	TGGCGCGCGC	1499, 2252	10
1540	45	GGCACCCGCGGCGCCCGGCA	2505, 2526	20
1541	45	CCGGGGCTGGTCAAACC	3043, 3073	17
1542	45	GGGCTCGTCAGGCCGGG	3061, 3091	17
1543	45	CATCAGCTCGCCGGGC	176, 3261	16
1544	45	GCCGGCAGCGCACCGG	2443, 2965	16
1545	45	ACATGGCCGCCGA	301, 4332	13
1546	45	CGCCGCCCTGCA	1037, 2585	12
1547	45	GCGCCCGCGGCC	2431, 2572	12
1548	45	CAACACCGAGG	218, 4693	11

1549	45	CAGGCCGCCGC	543, 2691	11
1550	45	CCAGCCCGGGG	3038, 3128	11
1551	45	CGCCGGGCCGG	276, 2163	11
1552	45	CGGCATCGAGG	329, 3868	11
1553	45	GAACCCGCCGG	1192, 1374	11
1554	45	GCAGGCCGCCG	542, 4087	11
1555	45	GCCCAGGCCGG	2724, 3156	11
1556	45	GCGCCCGGGGT	2932, 2953	11
1557	45	ACCGCTACTT	4269, 4446	10
1558	45	CACCGGCCGC	953, 3605	10
1559	45	CCGCCACCGA	28, 2737	10
1560	45	CCGGCGCCCA	2110, 2650	10
1561	45	CGATCGCCTG	1010, 2639	10
1562	45	CGGCACCGGG	2492, 3965	10
1563	45	CGTCGAGCGG	128, 1844	10
1564	45	GATCGCGCCG	1032, 4706	10
1565	45	GCGCACGCTG	53, 4483	10
1566	45	GCGCCGGGGT	634, 3010	10
1567	45	GGACAGCGCG	2910, 3249	10
1568	45	GGACGGTGGT	1689, 1871	10
1569	45	GGCATCACCA	1675, 2614	10
1570	45	GGCGTCGTAG	864, 2995	10
1571	45	GGTGAGCCCG	1407, 2985	10
1572	45	GTCGACGGGT	2767, 4304	10
1573	45	GTGCCGCGCG	1906, 2149	10
1574	45	TGCCCGGGCA	1130, 1669	10
1575	46	AGTGGCGCCTGATGCGTGCTACGACGATGCAGAGCGAAG CGATGAGGAGG	182, 232	50
1576	46	CCTGATCAGCGGC	718, 4667	13
1577	46	ACGCCGAATACG	1227, 1401	12
1578	46	CCGACACCGAGG	702, 1884	12
1579	46	CCGTGCGCAACC	3832, 4204	12
1580	46	CTGGGCGATCGG	938, 2241	12
1581	46	CGACTACGGGC	2863, 4610	11
1582	46	CGCGAACACCC	902, 2969	11
1583	46	GCAGCCGGCCG	625, 3670	11
1584	46	GCCCGCCGGCG	4076, 4642	11
1585	46	GCCCGGCGCCG	1453, 4569	11
1586	46	GCGCATGCCAC	398, 2502	11
1587	46	GCTGATCGCCG	2366, 4322	11
1588	46	GCTGCCGGCGG	1762, 2561	11
1589	46	GCTGGCGCCCG	4601, 4739	11
1590	46	GCTGGGCGTGC	2606, 4547	11
1591	46	GGCGCCGCGCG	4023, 4345	11
1592	46	GGCGGCGCTGG	489, 2234	11
1593	46	GGTGCTGCTGG	3043, 4733	11
1594	46	GGTGGCCGGGC	2740, 2833	11
1595	46	TCGTGTTGCGC	516, 3820	11
1596	46	ACGTACCTGA	3371, 3912	10
1597	46	CAGCGCACTG	65, 1282	10
1598	46	CCGGCTGGCC	1846, 3694	10
1599	46	CCTGGCGGCG	2914, 4339	10
1600	46	CGACGTGCGC	349, 2944	10
1601	46	CGATCCTGCG	1080, 3498	10
1602	46	CGCCGCCGCC	2399, 2522	10

1603	46	CGCCGCGCCG	1216, 2676	10
1604	46	CGCCGGCAGC	2054, 3926	10
1605	46	CGCGCTGCCG	1711, 4100	10
1606	46	CGCGCTGGCG	4241, 4598	10
1607	46	CGCGGTGGTG	1753, 3406	10
1608	46	CGCTGTTCAT	1380, 3276	10
1609	46	CGGCGAGCAC	1423, 2291	10
1610	46	CGGCTGGTGG	1321, 1535	10
1611	46	CTGGTCGCCG	2220, 2762	10
1612	46	GACCAAGGCC	565, 1876	10
1613	46	GCACATGGCC	50, 1471	10
1614	46	GCACCGGCGC	1294, 3327	10
1615	46	GCATCGCCTG	1726, 3069	10
1616	46	GCCGCTGCCG	1372, 2558	10
1617	46	GCTGGCCTAC	3025, 4154	10
1618	46	GGACGACGCC	1396, 1492	10
1619	46	TCGGCGCGGT	861, 2329	10
1620	46	TGCTGGGCGC	4018, 4191	10
1621	46	TTCGCCACCT	452, 4122	10
1622	47	AAACGTCGGCGGAGCCGACC	4706, 4734	20
1623	47	CGTGGCCGTCGTC	9, 3549	13
1624	47	TCGGCGCTGCTGG	1799, 3080	13
1625	47	TGATCGCCGACGG	557, 4076	13
1626	47	GGGCATCTCGGT	1543, 2704	12
1627	47	ATCGTCGGCGC	1738, 3311	11
1628	47	CAACGGCTGGG	1495, 3262	11
1629	47	CCTACAACGCC	2079, 3884	11
1630	47	CGCCGTCGTCG	1711, 3617	11
1631	47	CGCTGAGCGGC	2691, 3860	11
1632	47	CTCGCGCCCGG	1354, 2834	11
1633	47	GATCCTGGCCG	1750, 2980	11
1634	47	GCACGCGGTGT	396, 2611	11
1635	47	GCCGGTGGCTA	1862, 4509	11
1636	47	GGCGACGACGC	148, 3140	11
1637	47	GGGCGACATCG	3304, 4005	11
1638	47	GTTCCTGTCCA	705, 2788	11
1639	47	TCTGGGTGTAC	1971, 3111	11
1640	47	ACGGCGCGAT	2880, 2973	10
1641	47	CATGGGCGAC	144, 4002	10
1642	47	CCCCGACGGC	33, 297	10
1643	47	CCCGGGCGGC	3247, 4209	10
1644	47	CGCCGGCTCG	1268, 2926	10
1645	47	CGCGGGCCCG	2212, 4036	10
1646	47	CGGCAACGGC	3016, 4026	10
1647	47	CGGCAAGGCC	1612, 2518	10
1648	47	CGGCGGCGCG	1435, 2728	10
1649	47	GACCTGGTGC	343, 4054	10
1650	47	GCAGTTCCCG	3183, 3750	10
1651	47	GCCGCCGTCG	2852, 3615	10
1652	47	GCGAATCCAC	478, 3432	10
1653	47	GCTGGCCGCC	2815, 2893	10
1654	47	GGCCGGCCCG	874, 2368	10
1655	47	GGGGGCCGCC	1282, 4338	10
1656	47	GTACCTGCAG	1378, 1672	10
1657	47	TCGCCGGCTC	1448, 2925	10
1658	47	TCGGGCTGGC	470, 2481	10

1659	47	TGGGCCGCGA	124, 1404	10
1660	48	GGCCGCGGCCGCC	3266, 3871	13
1661	48	GTACGCCGGCCGG	314, 786	13
1662	48	AGGCGCTGCGCA	1549, 2835	12
1663	48	GGTGGTGCTGGC	3364, 3982	12
1664	48	TCCTCGACGACT	385, 4236	12
1665	48	ACTTCGCCGGC	3408, 4383	11
1666	48	CACCGGCACCG	2713, 3211	11
1667	48	CCCGGCCGAGC	347, 2275	11
1668	48	CCCGGCGTCGA	977, 2602	11
1669	48	CCGAGGCCGAC	538, 2526	11
1670	48	CCGCATCGTCG	2206, 3256	11
1671	48	CGAGCTGCCGG	1253, 3733	11
1672	48	CGCGACGGCGA	159, 3041	11
1673	48	CGGCCTGGAGG	656, 4546	11
1674	48	GATCGCGCTGG	3775, 4297	11
1675	48	GGCGATGGCCG	2116, 4099	11
1676	48	TCAAGGCGATC	943, 3768	11
1677	48	TCGCGGTCGCC	2511, 2862	11
1678	48	ACCGTCGCCC	858, 2690	10
1679	48	ACGGCCGCTC	3093, 3966	10
1680	48	ATCACGGCGC	723, 4323	10
1681	48	ATCGACCCGC	2540, 3173	10
1682	48	ATCGCCGTGC	258, 1746	10
1683	48	CCGCCGACGA	2241, 4509	10
1684	48	CGACGCCATC	2377, 4612	10
1685	48	CGACGCCGCC	425, 4408	10
1686	48	CGACGGCATG	1403, 3439	10
1687	48	CGACGGCCGC	1493, 3091	10
1688	48	CGCCCGCCGC	1968, 3034	10
1689	48	CGCCGAGGCG	3628, 3703	10
1690	48	CGCCGAGGGC	1385, 4033	10
1691	48	CGCGTCGGCG	2444, 4372	10
1692	48	CGTGATCGCC	3064, 4420	10
1693	48	CTTGCCGCGC	3565, 3791	10
1694	48	GACATGACCG	1988, 2138	10
1695	48	GCGAGCCCGA	2220, 3588	10
1696	48	GCGAGGTCTT	847, 3558	10
1697	48	GCGCGGCGAG	2617, 3345	10
1698	48	GGCCGAGGCG	1058, 3241	10
1699	48	GGGCCGCATC	1466, 3253	10
1700	48	GGGCGACCGC	248, 674	10
1701	48	GTCGGTCGCG	2158, 2776	10
1702	48	TCGAGCACGC	880, 2586	10
1703	48	TCGAGTACTC	1522, 4500	10
1704	48	TGCTCGAGCT	2798, 4224	10
1705	49	CCGATCATCGGCA	2403, 2873	13
1706	49	CCTGCCGTCGAGC	854, 3241	13
1707	49	GCGCCGGCGCCCG	595, 1233	13
1708	49	GCTGACCGCCGAC	1163, 3754	13
1709	49	CCGGGCGGCATC	1473, 2163	12
1710	49	CCGGCGAGGAG	1978, 3117	11
1711	49	CGCGCAGCACG	3415, 4584	11
1712	49	CGGCGGCGAGC	41, 2372	11
1713	49	CGGTGCGCGAC	1066, 3060	11

1714	49	GATCCAGGACG	2908, 3037	11
1715	49	GGCGCCGGCGC	594, 747	11
1716	49	TCGTCACCGGC	1456, 3084	11
1717	49	AACATCCCGC	363, 3569	10
1718	49	ACGAGGTCGA	1129, 4244	10
1719	49	ACGATCACCG	1560, 3698	10
1720	49	ACGCGCTGGT	694, 2646	10
1721	49	AGCCCGGCGG	37, 2577	10
1722	49	AGCTCGACGA	1501, 2098	10
1723	49	AGCTGGCCGA	1603, 2538	10
1724	49	CACCGCGGCC	197, 1514	10
1725	49	CATCGACGAG	665, 2393	10
1726	49	CCCGGCACCC	441, 625	10
1727	49	CCGAGGACGT	1570, 4574	10
1728	49	CCGCAGGCCG	1248, 4486	10
1729	49	CCGCCGCCGA	1305, 3927	10
1730	49	CCGGTGGCGC	168, 2895	10
1731	49	CGACGGCCAC	2240, 3457	10
1732	49	CGAGACGCTG	821, 1094	10
1733	49	CGAGCACCAG	1148, 2177	10
1734	49	CGCCGGTGGG	4185, 4444	10
1735	49	CGCTGGCCAA	238, 2685	10
1736	49	CGGCAAGCCG	1856, 3259	10
1737	49	CGGCGAGACC	500, 890	10
1738	49	CGGCGGCGCC	1889, 3721	10
1739	49	GAAGCGGGCC	1925, 4285	10
1740	49	GACCGCGGTG	737, 1061	10
1741	49	GATCGACGGC	1643, 2752	10
1742	49	GCCGACGTGC	1938, 3680	10
1743	49	GCCGCGCAGC	3413, 3818	10
1744	49	GCGACTTCCT	3225, 3396	10
1745	49	GGGCTCGAGC	1782, 4702	10
1746	49	GTCGAGTCGA	261, 4535	10
1747	49	GTGACGCCGC	1417, 4480	10
1748	49	TGCCCAAGCT	1495, 1831	10
1749	49	TGCGCAAGTC	3708, 3987	10
1750	49	TGGTCTACGA	685, 1732	10
1751	50	CGTCCCCGGCGCG	244, 2811	13
1752	50	GCGCTCCAGCGCC	1005, 3719	13
1753	50	CGCGACCCGCAG	903, 2971	12
1754	50	CAGCGCCCCGC	1044, 1677	11
1755	50	CAGCTCGCCGA	2535, 3244	11
1756	50	CGACGAACTCG	1874, 3913	11
1757	50	CGCCACCAGCG	870, 2244	11
1758	50	CGGGTGGCGGT	2107, 4639	11
1759	50	GAGGGGCGGGG	3222, 4101	11
1760	50	GCCGCCGGTGC	2142, 2268	11
1761	50	GCCGGGCCGGA	3385, 3995	11
1762	50	GGAGCTCCCGG	199, 767	11
1763	50	TCGACGAGTTG	809, 3068	11
1764	50	TCGGCCAGATA	479, 1057	11
1765	50	TGCCGTCGGCG	1814, 2793	11
1766	50	ACCAGCGCCT	1090, 1606	10
1767	50	CACTCGCCGG	2185, 3094	10
1768	50	CAGCGCCCGC	2379, 3106	10
1769	50	CAGCTGGGCC	2163, 2938	10

1770	50	CCCTCGAGCC	108, 967	10
1771	50	CCGGCGACCA	1267, 4619	10
1772	50	CGCAGCGCCC	1042, 2377	10
1773	50	CGCCTCGGCC	1200, 3734	10
1774	50	CGCGCCTCGG	1198, 2365	10
1775	50	CGCGGCGGGT	1961, 2482	10
1776	50	CGCGGTGATC	1233, 1500	10
1777	50	CGGGCGTTCA	39, 3041	10
1778	50	CGGGTGCCGA	1906, 2996	10
1779	50	CGTAGCGCTG	2468, 2672	10
1780	50	GAACACCAGC	1602, 3440	10
1781	50	GCGGAATTCG	3551, 3572	10
1782	50	GCGGGTGCCG	2788, 2995	10
1783	50	GCGGGTGGCG	1113, 4638	10
1784	50	GGCCCGCACG	3277, 4083	10
1785	50	GGCGCGCCGC	1692, 1731	10
1786	50	TCGGCGTCGA	1027, 1867	10
1787	50	TGCTCGATGA	1138, 3966	10
1788	51	GAAACGCAGGTTGCGCCA	331, 3546	18
1789	51	AGCACGTCGTCGGCCA	878, 4108	16
1790	51	CCGGCACCCGCGGCGG	225, 3440	16
1791	51	GTCCATCAGCACCGGC	172, 3387	16
1792	51	GCCAGCACGAACAGC	1103, 4489	15
1793	51	GCACGATGCCGGCC	399, 957	14
1794	51	AGCACGAACCCGA	2273, 2966	13
1795	51	AGCCGTTGATGCC	663, 4463	13
1796	51	CAGGTTCGGGTCG	2542, 3483	13
1797	51	CGATGGTCGCCAC	2394, 2988	13
1798	51	GAACAGGCACAGC	1486, 4746	13
1799	51	CAGCAGCGGGGT	2944, 4260	12
1800	51	CCCGTAGTTGCC	823, 4440	12
1801	51	GCCCACGCCAGC	3532, 4483	12
1802	51	GCCGGCGGCGGA	505, 3702	12
1803	51	GGCCCGGTGCCG	2777, 4061	12
1804	51	ACCAGATCGCG	1083, 3692	11
1805	51	ACCAGCCGTTG	1200, 4460	11
1806	51	ATCAGCCGCCA	1415, 4675	11
1807	51	CAGGATGACCG	1669, 4728	11
1808	51	CAGGTCGGTGA	7, 2509	11
1809	51	CCGATCGCGGT	1601, 4657	11
1810	51	CGCCGTAGCTG	1179, 3794	11
1811	51	CGGCCAGCACG	1101, 4103	11
1812	51	CGGCGAACAGG	1338, 2067	11
1813	51	CGGCGCGCTCA	676, 3034	11
1814	51	CGGTGGTGTAG	597, 4178	11
1815	51	GCAGGCCGATC	4541, 4652	11
1816	51	GGCGGCCAGCA	1684, 4347	11
1817	51	GGGTGAACGTG	2307, 4634	11
1818	51	AGCGGCCCGT	818, 4036	10
1819	51	CAGCGAACCG	1528, 1900	10
1820	51	CAGCGCCGCG	1840, 3843	10
1821	51	CAGGTCCTCG	274, 3984	10
1822	51	CATGTAGCCG	847, 2191	10
1823	51	CCGGCCTTCG	1048, 2618	10
1824	51	CGACCACCAG	3716, 4253	10

1825	51	CGCGACCACC	1271, 4251	10
1826	51	CGCGGCGTCC	2755, 3108	10
1827	51	CGGCCAGCGG	612, 3599	10
1828	51	CTCGGCCAGC	3597, 4101	10
1829	51	GAGCCGTTGA	662, 3856	10
1830	51	GCCGGGCCGC	1427, 4687	10
1831	51	GCCGTTGACG	118, 2725	10
1832	51	GGTGCCGTCC	3615, 3672	10
1833	51	GTACCACGCC	559, 1582	10
1834	51	TGCCCGCCGA	2577, 3893	10
1835	51	TGCCGGCGGC	504, 1680	10
1836	51	TTGCGGTCGG	2699, 3292	10
1837	52	CGATGCGGCGGCTCG	2541, 4721	15
1838	52	ATGGCCACCGCGG	338, 4588	13
1839	52	CCGCGACCGCGGC	1548, 4604	13
1840	52	GTCCAGCGCGCGG	1399, 1929	13
1841	52	GTCGAGGTCGGCG	1716, 2848	13
1842	52	AGCGCCACGATG	326, 950	12
1843	52	CGAGGGCGATCA	411, 1973	12
1844	52	CTGCGGCGCCAG	301, 3576	12
1845	52	GCCAGCCCGACG	827, 3472	12
1846	52	GCGCCCACCGGC	2569, 4123	12
1847	52	GGGCGCGGTGAT	1015, 1429	12
1848	52	CAGCACCGCGA	7, 940	11
1849	52	CCGTCGCCGTG	1482, 1792	11
1850	52	CGACAGCGCCA	3639, 4527	11
1851	52	CGCCGAGGGCG	408, 4100	11
1852	52	CGCCGGCCGCC	1164, 3716	11
1853	52	CGTCGACGCCG	2590, 3428	11
1854	52	GACGACGACGT	1267, 2738	11
1855	52	GCCCGGCGGCG	230, 4736	11
1856	52	AGCGACGCGG	721, 1623	10
1857	52	AGGTCGGCGG	2284, 3217	10
1858	52	CACCACCACG	163, 4410	10
1859	52	CACGTCGGCG	2318, 3681	10
1860	52	CAGCGGCGCC	205, 3168	10
1861	52	CAGGGCGATC	367, 3561	10
1862	52	CCAGCACCGC	195, 939	10
1863	52	CCAGCCCGGC	2387, 2928	10
1864	52	CCGGGTCGGC	2488, 4040	10
1865	52	CGAGCCGCCG	357, 3699	10
1866	52	CGCCAGGGTC	1692, 4257	10
1867	52	CGCGCCCAGC	190, 4620	10
1868	52	CGTCGGTGGC	1814, 3731	10
1869	52	CGTTGGGCGT	2758, 4070	10
1870	52	CTCGCGCGGC	1917, 2672	10
1871	52	CTCGGCGCCG	1159, 3384	10
1872	52	GACGCGCCCA	188, 3673	10
1873	52	GACGCGGGGC	1647, 2621	10
1874	52	GAGCGACGCG	184, 720	10
1875	52	GGCCAGCCCG	2385, 3471, 4179	10
1876	52	GGCGATGGGG	886, 2645	10
1877	52	GTCTCGTCGA	2798, 3424	10
1878	52	GTGAAGACCG	485, 2703	10
1879	52	TCAGCAGCGC	273, 321	10
1880	52	TCCACCAGCA	383, 4162	10

1881	52	TCCAGCACCG	131, 938	10
1882	52	TCGCGCGCAC	1284, 2190	10
1883	53	AACCAGCCGGGCG	1570, 3142	13
1884	53	CAGCGCGCCGATG	364, 847	13
1885	53	GGCGGCCAGCACG	3980, 4205	13
1886	53	GGTCAGCACCAGC	943, 2898	13
1887	53	ACCGGCAGCAGC	392, 1511	12
1888	53	CCAGCAGCACGC	1536, 2333	12
1889	53	CGCACCACCAGC	1262, 2476	12
1890	53	CGCGGCCCGCAG	1947, 2877	12
1891	53	CGGCGAGCCGGG	1117, 3206	12
1892	53	GTGACCGCGGCC	224, 2971	12
1893	53	ACCGCGACGGC	3052, 3493	11
1894	53	AGCACCAGCGC	41, 3034	11
1895	53	CAGCACCAGCG	244, 3033	11
1896	53	CAGCGCCATCA	607, 2745	11
1897	53	CCACAGCGCCA	664, 2742	11
1898	53	CCACCGGGACG	1248, 2258	11
1899	53	CGCGCGGCTTG	2066, 2279	11
1900	53	CGGTCGCCGCG	1014, 2140	11
1901	53	CGGTGACCGCG	222, 3670	11
1902	53	CGTCGGAGGTG	786, 1866	11
1903	53	CTGACACGGGA	1399, 4457	11
1904	53	GCCGCCGCGCG	533, 3118	11
1905	53	GTCTGGTCGCG	191, 2059	11
1906	53	TGTCGACGGTC	1008, 3700	11
1907	53	ACGCGATCGC	381, 2225	10
1908	53	AGGCCGGCCA	3388, 3452	10
1909	53	ATCACCACGG	2050, 2650	10
1910	53	ATCTGGCCGA	3529, 3903	10
1911	53	ATGCCCAGGG	449, 3289	10
1912	53	ATGGCGACCA	308, 2503	10
1913	53	CAACAGCACG	2919, 3938	10
1914	53	CACCACCGCG	863, 3489	10
1915	53	CAGCCCGGCG	1384, 1552	10
1916	53	CAGCGCACCG	256, 2445	10
1917	53	CAGCGCCGCC	3114, 3513	10
1918	53	CAGCTCGTCG	1276, 1905	10
1919	53	CAGCTGGGCG	1489, 3336	10
1920	53	CAGGGCGCCG	319, 1752	10
1921	53	CGCCAGCGTG	2385, 2964	10
1922	53	GAAGCGGCGG	61, 880	10
1923	53	GACCGCCAGG	502, 2673	10
1924	53	GATGGCCAGC	1698, 2439	10
1925	53	GCCGATCACC	2646, 2829	10
1926	53	GCTCCACCGA	423, 4135	10
1927	53	GCTGACACGG	1604, 4456	10
1928	53	GGACACGAAC	3315, 3772	10
1929	53	GGCCAGCGTC	184, 2292	10
1930	53	GGCGGCGTCG	1037, 4175	10
1931	53	GGGGCCGACG	142, 1713	10
1932	53	GGTCAGCCCG	1150, 1381	10
1933	53	GGTCAGCGCG	361, 3420	10
1934	53	GGTGTAGTTG	1827, 3965	10
1935	53	GTCCATCGCG	4558, 4690	10

1936	54	CCACTAGGTATCGATGGTGGCGACCCGCTTCGCCCGGCTC CGCCGCGCTCGCGATCGCCACT	4485, 4599	62
1937	54	GTTCCAGATGATC	3807, 4152	13
1938	54	CAATTGGTCCAG	197, 662	12
1939	54	CGCCAGCGCCGG	2301, 3936	12
1940	54	CGCCGTCGGCGA	355, 1705	12
1941	54	GCCAACTCGCGC	2662, 3433	12
1942	54	GCGATCAACTCG	1536, 3160	12
1943	54	TCGGCGTCGTCG	459, 3385	12
1944	54	CCAGGCCGCCG	754, 1391	11
1945	54	CCGGCCGCCCG	818, 831	11
1946	54	CGATGCGGGAC	992, 3761	11
1947	54	CGGGCAGCGTC	1508, 1741	11
1948	54	GAAACGCCAGG	1160, 3515	11
1949	54	GCGCCGGCGCG	1442, 2711	11
1950	54	GCGGCCCGGCC	234, 1771	11
1951	54	GGTGATGGTGC	479, 1290	11
1952	54	GTTGATCAGGT	1275, 4143	11
1953	54	TCGGCGCTGAT	708, 2146	11
1954	54	AGAGCCCGAA	634, 1367	10
1955	54	ATCCGGGCTG	1030, 2805	10
1956	54	CAGCCGGGTG	698, 923	10
1957	54	CAGGTCGCGG	2559, 4362	10
1958	54	CCCGGCAGCA	615, 2701	10
1959	54	CCGCAGCCGG	1925, 2738	10
1960	54	CCGCCGGGCA	1494, 1737	10
1961	54	CGACGGCCGG	2267, 2595	10
1962	54	CGCCGACCGC	2118, 2732	10
1963	54	CGGCCCCGAC	3102, 3307	10
1964	54	CGGGACCGCC	1489, 2295	10
1965	54	CGGGCGATCA	804, 3157	10
1966	54	CTCGGCCAGC	2748, 2979	10
1967	54	CTGCACGACG	1080, 3840	10
1968	54	GAACAGCAGC	1353, 3852	10
1969	54	GATCGCCCAC	1137, 1867	10
1970	54	GATGTCGCAG	572, 2511	10
1971	54	GCGCGCGGTC	1628, 2930	10
1972	54	GCGGGCGATC	803, 1023	10
1973	54	GCTGCTTGAT	4085, 4301	10
1974	54	GGTAGTGCAG	1151, 3734	10
1975	54	TTCGCTGCCG	302, 2216	10
1976	55	GCCGCCCGCCTTGCC	1490, 1642	15
1977	55	GCGACGACGGTGTCG	781, 4351	15
1978	55	GACGCCGCCGATG	2077, 2935	13
1979	55	ACGCGGCCAGCG	3408, 3921	12
1980	55	CACCGTGGTCG	904, 2620	11
1981	55	CAGCAGGGTGG	477, 2764	11
1982	55	CCAGGCCGCGG	380, 3756	11
1983	55	CGGCGACGGCG	2439, 3369	11
1984	55	CGTCGTCGTCG	515, 4538	11
1985	55	GCAACAGGATC	1063, 3105	11
1986	55	GCGGTCGCGGT	3289, 3859	11
1987	55	GGCCAGCGGCC	2899, 3827	11
1988	55	TGCGCAATTCG	350, 1788	11
1989	55	TGTCCCGCAAC	637, 1438	11
1990	55	ATCCTGCGGG	501, 3839	10

1991	55	ATCGCCCCGG	2344, 4061	10
1992	55	CAGCCACACC	528, 3511	10
1993	55	CCACCGCGCC	1039, 2829	10
1994	55	CGCACGCCAG	470, 3135	10
1995	55	CGCCGAGGTC	1282, 2356	10
1996	55	CGGGAAGTCG	2977, 4710	10
1997	55	CGGGCCCCGG	2498, 2702	10
1998	55	GAACAGCCCC	1747, 3019	10
1999	55	GAACAGCGCG	291, 1723	10
2000	55	GATCACACCG	899, 3226	10
2001	55	GATGCTGGTG	1082, 1891	10
2002	55	GCCTTCTTCA	1017, 1512	10
2003	55	GCGCCGCCAT	2192, 4424	10
2004	55	GGACTGCATC	1196, 4276	10
2005	55	GGCCCACATG	3772, 4242	10
2006	55	GGCCGGAACG	194, 4168	10
2007	55	GGCGTCGTGC	2156, 4486	10
2008	55	GTTGCGGGTA	2110, 2400	10
2009	55	TCGCCGAACA	2573, 2813	10
2010	56	CGGCCGCCAGCCCG	2085, 3253	14
2011	56	CTCGTCGGTGCGCA	1855, 2389	14
2012	56	GTGATGTCGGCGCC	1097, 2507	14
2013	56	CGGCGCTGTCGAT	3063, 4303	13
2014	56	CATGCCGATGAT	952, 1471	12
2015	56	CGGCGTCGGCGT	2745, 4646	12
2016	56	CGTCGGCCACCA	792, 1992	12
2017	56	GGCGATCACCGC	2668, 2734	12
2018	56	TCCTTGCCGGTG	3777, 4705	12
2019	56	ACGACCTCGCC	1568, 3089	11
2020	56	CCGCACCGATC	1824, 4330	11
2021	56	CCTTGACGCCG	936, 4568	11
2022	56	CGATCGCCTTG	2322, 3619	11
2023	56	CGGTGGCGTGG	3667, 4616	11
2024	56	CGTCGGCGCTG	2658, 3060	11
2025	56	GCCTCGGCGTC	2807, 4642	11
2026	56	GGCCGCGGGCC	2625, 3927	11
2027	56	GGCCGCGTCGA	1306, 4692	11
2028	56	GTCGTCGGCGC	3058, 3905	11
2029	56	TGAGCACCACG	1260, 4204	11
2030	56	ACCACACCGT	1275, 2651	10
2031	56	CACACCGGAC	1324, 2066	10
2032	56	CCCGCAGCGT	873, 1917	10
2033	56	CCGCCGATCG	155, 3615	10
2034	56	CCGCCGGACT	1046, 4411	10
2035	56	CCTCGATCGC	2319, 2724	10
2036	56	CGCGGCCAGC	2269, 4629	10
2037	56	CGGCGGCCTG	46, 1515	10
2038	56	CGTCCTCGAT	2172, 2721	10
2039	56	CGTGGTGGCG	121, 1685	10
2040	56	CTCGTCGGCC	1909, 1990	10
2041	56	CTTCTCGGCG	3701, 4680	10
2042	56	GACCATTCCG	65, 1421	10
2043	56	GATCCGCTCG	1849, 4142	10
2044	56	GATCTCGGCG	2110, 3503	10
2045	56	GATGACCCGC	889, 3464	10

2046	56	GCCCGGTGGC	855, 3664	10
2047	56	GCCGTGGCCG	3148, 4184	10
2048	56	GCCTCGTCGG	1907, 2387	10
2049	56	GCGATCGTGT	167, 3180	10
2050	56	GCGGCGCCCT	77, 1937	10
2051	56	GGCCGTCACC	544, 817	10
2052	56	GGCGGCGATC	3176, 4599	10
2053	56	GTCAGCTTCG	476, 2940	10
2054	56	GTCGAGGATC	1027, 3743	10
2055	56	GTGCGGCGCG	237, 3320	10
2056	56	TTCGGCGACG	1630, 2344	10
2057	57	CGTCGTCGTGGT	3552, 3594	12
2058	57	TCGAGCTCGGAA	1623, 4375	12
2059	57	TCGCGGGCGAAA	1594, 1727	12
2060	57	ACGCGGCCGCG	217, 901	11
2061	57	AGCGGCCGAAT	2868, 3281	11
2062	57	AGGTCGACGCC	101, 922	11
2063	57	CCAGCGCCGCC	2571, 3698	11
2064	57	CGATGGCCCGC	321, 2625	11
2065	57	CGGGCTGGGCG	803, 3609	11
2066	57	CTCGCCGCCCT	1556, 3186	11
2067	57	GCCAGCGCCGC	2570, 4663	11
2068	57	GCGACATGGTC	434, 4450	11
2069	57	GCGCCAGGTGG	752, 4134	11
2070	57	GGCGAGGTCCA	274, 1682	11
2071	57	GTCGGCGTCCA	340, 1448	11
2072	57	TCGGTGCGGGC	1709, 1958	11
2073	57	TCGGTGCTCGG	3418, 3472	11
2074	57	ACGGTCGTCG	2396, 3454	10
2075	57	CCGGCGCGGT	1080, 3569	10
2076	57	CGAGAACTTG	52, 1433	10
2077	57	CGCAGGCCCG	2096, 3947	10
2078	57	CGCCCTTGCC	1279, 1411	10
2079	57	CGCGTCGCCC	4521, 4596	10
2080	57	CGGTGACCGT	3434, 3497	10
2081	57	CGTCGCGCAG	653, 1911	10
2082	57	CTTGCCGGGC	406, 2713	10
2083	57	GAGGTAGAAG	1244, 4584	10
2084	57	GATCAGGTCG	97, 2062	10
2085	57	GATCGCCGCG	2341, 2638	10
2086	57	GATCGTGCAC	1333, 4179	10
2087	57	GGCCGCGGCG	764, 2473	10
2088	57	GGTGCCGGGG	3536, 4721	10
2089	57	GTCCGCCGAA	3347, 3359	10
2090	58	AACGCCGAGATC	1356, 1563	12
2091	58	AGCTGGTCAACG	1075, 1348	12
2092	58	GGTCGCCGGCCG	1701, 2227	12
2093	58	TGGCGATCGTCT	3169, 4480	12
2094	58	ATCGACAACGG	3041, 4371	11
2095	58	CCGGATGCTCG	3632, 3863	11
2096	58	CGACGCCGGCG	357, 911	11
2097	58	CGCGCAGCTGG	958, 1070	11
2098	58	GACCCCGACCG	1287, 3464	11
2099	58	GGTGGCGGCGG	556, 3389	11
2100	58	TCGTCACCGGC	3589, 4237	11
2101	58	ACGACCGCGA	1446, 4012	10

2102	58	ATCGTCACCG	3588, 3654	10
2103	58	CAACGGATGG	3999, 4574	10
2104	58	CACGGAGTCC	115, 379	10
2105	58	CCCTGCCCGG	77, 4732	10
2106	58	CCGAGCCGAA	3286, 3564	10
2107	58	CCGCTACGGC	96, 2255	10
2108	58	CCGGTGGCGG	554, 4675	10
2109	58	CGCGGCGCCG	433, 3557	10
2110	58	CGCGGCGCGG	3077, 4250	10
2111	58	CTGGCGGCCG	939, 3672	10
2112	58	GCGCACGCCC	1400, 3956	10
2113	58	GCGGGCACGG	1669, 1960	10
2114	58	GCGGGCGCTG	2767, 3710	10
2115	58	GCGGTGACCG	1972, 3195	10
2116	58	GCGTACGCGA	465, 3802	10
2117	58	TCCAACATCG	2999, 3609	10
2118	58	TCCAGCGGGC	56, 1665	10
2119	58	TCGTCGTCCT	2571, 3160	10
2120	58	TGACCGCCGG	1481, 2703	10
2121	59	CCGCGCCGACGTCG	693, 1444	14
2122	59	CGCGTTCCATGACG	3739, 3856	14
2123	59	CGTGCCGGCGGCG	178, 2496	13
2124	59	CGAGGTCGCCG	865, 1177	11
2125	59	CGGCGGCGATG	66, 3140	11
2126	59	CGGGCTCGGCC	1150, 3041	11
2127	59	CTCGACGTCGC	2108, 3476	11
2128	59	CTTGCCGGCGA	3207, 3770	11
2129	59	GATCGACGGCG	1234, 2888	11
2130	59	GCCCGCGCAGC	2091, 2684	11
2131	59	GGGTCGGCCAC	3289, 3630	11
2132	59	TCCAGGACGAC	567, 2725	11
2133	59	TCCGCGACAGC	2513, 2947	11
2134	59	AGGGCCGTCT	378, 1676	10
2135	59	CACCGAGGCG	1273, 1417	10
2136	59	CCACAACGTC	1666, 2016	10
2137	59	CCGGAACGGG	3281, 4311	10
2138	59	CCGTGCCGGC	2495, 3577	10
2139	59	CGACCGGGTC	1786, 2717	10
2140	59	CGACGGCAAC	1708, 1990	10
2141	59	CGAGCAGCGC	3062, 3526	10
2142	59	CGATCCACCG	2709, 3658	10
2143	59	CGCCCGCCGC	2639, 3999	10
2144	59	CGCCGAAATG	1285, 1594	10
2145	59	CGCCTCGGCG	2802, 3812	10
2146	59	CGCGACGGCA	1988, 2209	10
2147	59	CGCGGCGGCG	905, 2445	10
2148	59	CGCTGGGCCA	4022, 4432	10
2149	59	CGGATCCGCG	2761, 4587	10
2150	59	CGGCGATCTC	3158, 4241	10
2151	59	CGGGCCCCGG	3275, 3409	10
2152	59	CGTCGATCAC	2396, 3302	10
2153	59	GCACGTCGGG	3224, 4679	10
2154	59	GCCCGCGGCG	283, 2913	10
2155	59	GTATGGCCGG	2484, 2624	10
2156	59	GTCCTTGCCG	154, 3767	10

2157	59	TCGGTGAGCC	1658, 4159	10
2158	60	TCACGTCGGTGAG	722, 989	13
2159	60	ACGCCGTCGGCC	4072, 4328	12
2160	60	CGCGGGTGGTGA	27, 3314	12
2161	60	AGGGCATGCCG	1573, 2071	11
2162	60	CCCGGCCGGTC	1280, 2631	11
2163	60	CCCGGGCGCCG	2197, 2696	11
2164	60	CGCGGTCGACG	3510, 3549	11
2165	60	GCCGAAGCCCT	256, 1904	11
2166	60	GGCCGCGGCGG	1981, 2864	11
2167	60	TGCCGGTGTCG	168, 2089	11
2168	60	ATCAGCGTGT	748, 1036	10
2169	60	CACGATCGGG	196, 4149	10
2170	60	CAGCGGCTGC	3778, 4447	10
2171	60	CCAACTCGAG	3897, 4239	10
2172	60	CCAGCCAGCG	1861, 2326	10
2173	60	CCCGTCGAGC	1083, 3442	10
2174	60	CCGCGCCGAA	252, 1223	10
2175	60	CGCGAGCGCC	2933, 3567	10
2176	60	CGGCCCGAAA	771, 2707	10
2177	60	CGTCGAGGCC	1816, 2902	10
2178	60	GGAACGCCAG	1855, 1972	10
2179	60	GGGCTGACCG	3379, 3813	10
2180	60	GTTCAGCCAC	3600, 4251	10
2181	60	TCCGGTTGGG	3021, 3090	10
2182	60	TGATCAACGT	234, 3617	10
2183	60	TGCCCGAGGA	3467, 4418	10
2184	60	TGGTGCGCCG	4043, 4223	10
2185	60	TTCCGGTGGC	3987, 4136	10
2186	61	TCGACGACGGCGGC	1229, 2583	14
2187	61	GCTGCTGTTCGGG	3578, 3593	13
2188	61	CCGCGCTGCTGG	2939, 3652	12
2189	61	CTGGCCGCCGAC	2018, 2615	12
2190	61	CTGTTCGTGGTG	2857, 3525	12
2191	61	GCGACGCCGCCG	93, 2996	12
2192	61	GCGGCTCGGGAT	1792, 4277	12
2193	61	TGGTGCTCGACG	1334, 3946	12
2194	61	CACGCGGATCG	2790, 3030	11
2195	61	CCGCGCCCGCG	1025, 4668	11
2196	61	CGCATCCTCGA	2890, 3423	11
2197	61	CGCGGCCGCCA	2817, 4202	11
2198	61	CGTCGACGACG	2581, 4610	11
2199	61	CTGCGGCTCGG	1790, 2168	11
2200	61	GATCATCGCCG	34, 783	11
2201	61	GTGCTGGCCGC	2612, 2627	11
2202	61	AACTTCGTCG	1216, 3351	10
2203	61	CACCGGCCGC	3509, 3995	10
2204	61	CACGCCCGAG	109, 2353	10
2205	61	CACGGTGAGC	4535, 4601	10
2206	61	CATCATCGCG	3210, 4523	10
2207	61	CCCGGGCGAT	999, 3715	10
2208	61	CCGGCCGGGC	1162, 1206	10
2209	61	CCGTCGCTGG	2845, 3618	10
2210	61	CGCCGCGCTG	2545, 2937	10
2211	61	CGGGCGACGC	90, 495	10
2212	61	CGGTTCCGGT	1866, 3062	10

2213	61	GAGGTGCCCA	1640, 3759	10
2214	61	GCCGCTGGGC	2422, 2766	10
2215	61	GCGAACGCGT	1072, 4309	10
2216	61	GCGGTGTCGG	1432, 1480	10
2217	61	GGTGCTCGAG	1921, 2958	10
2218	61	GTTCTCCGCG	2056, 4708	10
2219	61	TCGACGCCGC	2541, 3706	10
2220	62	GGAGCCGGGCGCAGCGGGTCGCC	1479, 1511	23
2221	62	TGCGCACGCCGACG	3120, 3464	14
2222	62	TCGCCGGCATCGG	601, 2312	13
2223	62	GCCGTCGGCGGC	689, 3274	12
2224	62	GCGGCCGCCGAG	1073, 1731	12
2225	62	GGCCCGCGCCGT	1164, 1661	12
2226	62	GTGATCAGCGCC	1635, 2756	12
2227	62	TCGGTCGGTGAT	1455, 1544	12
2228	62	AGTGCGCGGCG	1934, 1960	11
2229	62	CGACGACCTCG	505, 1270	11
2230	62	CGCCGCCGTGG	752, 3885	11
2231	62	CGCGGCCGCCG	1730, 2556	11
2232	62	CGCGGGTGCGC	1811, 3114	11
2233	62	TCGACCGCGGG	99, 3109	11
2234	62	ATGCCGACGA	1380, 3574	10
2235	62	CAGCTTCGCC	2619, 3510	10
2236	62	CATCGCCCGC	137, 1402	10
2237	62	CGAGGCGGTC	1339, 3988	10
2238	62	CGCCACCGCA	1201, 3800	10
2239	62	CGCCGCAGGC	2492, 3631	10
2240	62	CGCCGCGACG	500, 1592	10
2241	62	CGCGGTCGTG	1006, 1628	10
2242	62	CGGCGCCCCG	404, 2137	10
2243	62	CGGGCGGCTG	1838, 4342	10
2244	62	CGTCGCGGAA	181, 4018	10
2245	62	CGTCGGCGAC	2547, 3833	10
2246	62	CTGCGCAGCG	1100, 3588	10
2247	62	CTGGTCAGGG	903, 915	10
2248	62	GAGGCCCTGG	1683, 3723	10
2249	62	GCCGGCGGCG	641, 2680	10
2250	62	GGCGATCATC	2706, 3761	10
2251	62	GGCGCGGTCG	1626, 1875	10
2252	62	TGCCCGCCAT	457, 592	10
2253	62	TGCGACGGCG	4352, 4365	10
2254	62	TGGCGGCCGC	277, 1071	10
2255	63	TAGCCCGGGTACCCG	3724, 3751	15
2256	63	CCCTGCGGATT	424, 3811	11
2257	63	CGGCGATCGTT	1574, 1975	11
2258	63	CGGGCAGATGG	1454, 1900	11
2259	63	GACCCGCGCGG	2743, 2888	11
2260	63	GGGGTCGCCGA	2666, 3255	11
2261	63	GGGTGTGCGGC	579, 4304	11
2262	63	TGTCGACCCGC	1753, 2739	11
2263	63	CCGCCGGCTT	610, 860	10
2264	63	CCGGCGGTGG	3646, 3791	10
2265	63	CGCGCCCCGG	3315, 4562	10
2266	63	CGCGGAGGCC	3083, 4646	10
2267	63	CGCTGACCGG	4086, 4712	10

2268	63	CGTCGACCTC	548, 4619	10
2269	63	GCGCGTCGGC	177, 1556	10
2270	63	GCGGCGATCG	1573, 4737	10
2271	63	GCGGCGGCGA	1570, 1699	10
2272	63	TGTCGCTGAT	941, 2392	10
2273	64	GCGAGGCGCAGAACT	3116, 4382	15
2274	64	CGGCCGCATCCA	927, 2734	12
2275	64	GCGTGCGCGACC	749, 1321	12
2276	64	GGCCGGCAGCAC	3798, 3999	12
2277	64	TCGCCCGGGCTG	1143, 3649	12
2278	64	ACGTGGCGCGC	2664, 4301	11
2279	64	CAAGGTGCTGC	489, 3210	11
2280	64	CCGCCCGGTCA	4194, 4617	11
2281	64	CGGCATCCAAG	1448, 3278	11
2282	64	CTCGACCACCT	4275, 4324	11
2283	64	GCGGGGCGACC	1694, 3686	11
2284	64	GCTCGGCGGCC	1547, 3584	11
2285	64	GGCCACGTCGA	1290, 4242	11
2286	64	GGCTGCGCAAC	1831, 2254	11
2287	64	TGCTCGAGCGG	830, 2687	11
2288	64	TTCGACGCCGT	1242, 2046	11
2289	64	ACCGATCAAC	2901, 3007	10
2290	64	ACCTGGTGAA	161, 764	10
2291	64	ACGTCGGCGG	2753, 3535	10
2292	64	AGGTGCGCGA	875, 3110	10
2293	64	CACCGAGCTG	69, 2024	10
2294	64	CCCGAGATCG	91, 2830	10
2295	64	CCGCGGCCGG	981, 2586	10
2296	64	CGCAGGGCCG	1180, 4610	10
2297	64	CGCCGACGAA	969, 4440	10
2298	64	CTGATCTCGG	1569, 3100	10
2299	64	CTGCTCGAGC	2686, 3249	10
2300	64	GCCCGGCGTC	1093, 4146	10
2301	64	GCCGCGGCCG	2585, 4649	10
2302	64	GCGTGGCGAG	1368, 3557	10
2303	64	GCTGGCGGGC	1487, 3225	10
2304	64	GGCACCACGC	151, 3412	10
2305	64	TACCTGGACG	898, 4057	10
2306	64	TACGGCGGAT	2992, 4156	10
2307	64	TCGACCTCGA	383, 1513	10
2308	64	TTCGCCTCGC	2314, 4315	10
2309	65	ACCCGCTGCGCCCGGCTACGGCCTATAGCGGCG	2684, 2717	33
2310	65	TGGCCGCCGCGCCG	595, 1483	14
2311	65	CGCGCGCAACAAG	734, 2318	13
2312	65	CGCGACCTCGCC	275, 2391	12
2313	65	CAGACGCTGCT	1176, 4675	11
2314	65	CAGGTCGCCGA	338, 3565	11
2315	65	CCCGCAGCGAC	1376, 3822	11
2316	65	CCGGCGCGCGT	2420, 4056	11
2317	65	CGACGGCGTCG	1049, 3927	11
2318	65	CGCATCTGCCC	23, 405	11
2319	65	CGCTGGGCGAG	967, 2284	11
2320	65	CTCAACGACCT	95, 1923	11
2321	65	GCACATCACGG	995, 3109	11
2322	65	GGCCGGCGCGC	1010, 4054	11
2323	65	GGTCGACGCCG	1406, 2600	11

2324	65	GGTGGCCGCCG	541, 1481	11
2325	65	TCGCGGCGGCC	2191, 4047	11
2326	65	TGGCCAACGCC	2173, 3348	11
2327	65	AACCCGATCA	3158, 3476	10
2328	65	CACCGCCCGC	2063, 3817	10
2329	65	CAGCGCCGCC	3319, 4420	10
2330	65	CCCCGACCAG	2465, 3546	10
2331	65	CCCGGTGTCG	3835, 4219	10
2332	65	CCGCAGCGCC	3784, 4417, 4459	10
2333	65	CCGCGGCCTG	220, 463	10
2334	65	CCTCGACCTC	1856, 2949	10
2335	65	CGACGTGATC	641, 3006	10
2336	65	CGCCCCGACC	2498, 3544	10
2337	65	CGCCGAGACC	3663, 4033	10
2338	65	CGCGCAACAC	160, 1249	10
2339	65	CTGGGGCCGC	1641, 1937	10
2340	65	GACGACGCCG	1356, 2983	10
2341	65	GAGCGGGCCG	932, 1215	10
2342	65	GATGGCGTCG	115, 3616	10
2343	65	GCCACGACGA	1699, 1990	10
2344	65	GCCAGGCCGC	3948, 4641	10
2345	65	GCTGCGGGTG	569, 1316	10
2346	65	GGCCATGTCC	1286, 4075	10
2347	65	GGCCGCGCTT	312, 2789	10
2348	65	GGCCTACGCG	37, 2312	10
2349	65	GTTCGGCGCC	632, 3333	10
2350	65	TCGACGACCC	174, 1561	10
2351	65	TCGACGGCGT	3585, 3926	10
2352	65	TGCACATCAC	910, 3108	10
2353	65	TGCGCGCCCG	1720, 4100	10
2354	65	TGCGGTGCGC	766, 2848	10
2355	65	TGGTCGCCGA	1417, 1516	10
2356	66	CGCGGCGCGGAACCGC	2856, 3417	16
2357	66	TCCAGGTTCGGC	690, 1616	12
2358	66	TCGCCGTTCTTG	1748, 3729	12
2359	66	TGGTCAGCAGCG	2292, 2798	12
2360	66	AGCGCCGCGCC	420, 3310	11
2361	66	AGGCCTTGGCG	2178, 2933	11
2362	66	CCCGCGCCGCC	330, 1057	11
2363	66	CCCGCGGCCGC	932, 1134	11
2364	66	CCGCGTCGGCG	655, 2921	11
2365	66	GCAGCGCCGCG	418, 901	11
2366	66	GCCGATGTCGG	2775, 3279	11
2367	66	GCTGCTGCTCG	2361, 3601	11
2368	66	GGGGCTTGTTG	7, 2538	11
2369	66	GGTCGACGTCG	511, 4373	11
2370	66	GGTCGGCCGAG	58, 3299	11
2371	66	GGTGATGCGGC	356, 404	11
2372	66	GTGGTGATGCG	111, 402	11
2373	66	GTTCGTAGAGG	754, 2636	11
2374	66	TGGTGCCGCCG	307, 726	11
2375	66	TGTAGCCGATG	628, 2771	11
2376	66	ACCAGGTCGA	507, 4521	10
2377	66	CAGGTAGGCG	1247, 2395	10
2378	66	CAGTTCGTAG	1361, 2634	10

2379	66	CCAGGCCGGC	3134, 4447	10
2380	66	CCGATGTCGA	1012, 1571	10
2381	66	CCGTCGAGAT	3079, 3823	10
2382	66	CGCGCGCGCC	1073, 3754	10
2383	66	CGGCGCGCCC	3861, 4262	10
2384	66	CGTCGACGAT	2025, 3715	10
2385	66	CGTTCGCGCA	672, 4074	10
2386	66	CTCGAGCACC	1097, 3147	10
2387	66	CTCGATCGCC	887, 2946	10
2388	66	CTCGATGAAC	98, 569	10
2389	66	CTCGGTCAGG	2667, 3261	10
2390	66	GACAGGAACC	1173, 3591	10
2391	66	GCCCAGGCCG	4445, 4695	10
2392	66	GCCGATCACG	4004, 4226	10
2393	66	GCGCACCGCG	221, 4676	10
2394	66	GCTCGGGCCC	1112, 4204	10
2395	66	GGCCCCGCCG	1876, 3456	10
2396	66	GGCGATCTCG	707, 3707	10
2397	66	GTAGATGTCG	1669, 1930	10
2398	66	GTCCGCCAGG	785, 3569	10
2399	66	GTGCCACAGC	2386, 3836	10
2400	66	TCCAGCGTTT	468, 3948	10
2401	66	TCGGCGGCCG	987, 3582	10
2402	67	CGGCCAGCTCGCG	877, 2033	13
2403	67	GTCGGCGCGGATC	2100, 4231	13
2404	67	ACCGCCGCGAGC	2119, 3249	12
2405	67	CTGCCGAACCTG	2892, 2931	12
2406	67	GCGGCTGCCGAA	2888, 3527	12
2407	67	TCGACGTCGGCG	106, 1924	12
2408	67	TCGGCCTGCTCG	3148, 3971	12
2409	67	TGATCACCATCG	92, 2908	12
2410	67	ATACCTCGGCC	1522, 1693	11
2411	67	CAGCAGCTCCT	2529, 4099	11
2412	67	CCGGCGATCAC	1751, 3508	11
2413	67	CGCCGAGGCGC	583, 1790	11
2414	67	CGTCGTCACCG	2447, 4174	11
2415	67	GGCCGCCGAGG	1192, 1787	11
2416	67	TCGGTGGTGAT	917, 4735	11
2417	67	TGGCGGCCGCG	1814, 4574	11
2418	67	TTCGCCGGCGC	356, 1151	11
2419	67	ACGCCACCAG	1964, 4367	10
2420	67	AGCAGCCGAT	753, 4298	10
2421	67	CAGCGCCCGA	467, 1283	10
2422	67	CCGCGATGAG	2075, 2462	10
2423	67	CCGGCCCGCG	1663, 2070	10
2424	67	CGACGCCCGC	547, 1111	10
2425	67	CGCCCGCGCC	9, 1898	10
2426	67	CGCGTCGTCG	736, 2442	10
2427	67	CGGAGCCGAG	1509, 4622	10
2428	67	CGGCCGCCGA	1786, 2809	10
2429	67	CGGCGGCTGC	2223, 3525	10
2430	67	CGGTGTCGGC	2249, 3966	10
2431	67	CGTCGCCCGC	622, 1895	10
2432	67	CGTCGGCGGC	1384, 2571	10
2433	67	CGTGCCGATC	1630, 2058	10
2434	67	CTGCGCCAGG	2421, 2490	10

2435	67	CTGCGGCCGT	2823, 4152	10
2436	67	CTTCGAGGAG	2318, 4050	10
2437	67	GCCGAAGAGC	830, 3075	10
2438	67	GCCGAGCGCG	2189, 4719	10
2439	67	GCCGCGACCT	136, 4540	10
2440	67	GGACGCGGCC	2804, 4418	10
2441	67	GGCGTCGGCG	2097, 3841	10
2442	67	GGTCGCCCGG	3880, 4695	10
2443	67	GTCGCGCGAA	711, 1490	10
2444	67	GTCGTCGGCG	183, 2569	10
2445	67	GTTGATCACC	90, 1069	10
2446	67	TCGACCAGCT	434, 1218	10
2447	67	TCTCGCGGCC	823, 1425	10
2448	67	TGCTCGCCGA	579, 894	10
2449	67	TGGTCGCCCG	2748, 3879	10
2450	67	TTCCGTGGCG	340, 2091	10
2451	68	CCGCTGCGCCGCGC	2681, 3703	14
2452	68	CGTCGGCGCTGG	1675, 4059	12
2453	68	GTCGTCGAGGTC	489, 4418	12
2454	68	CCCTGGGCGGC	239, 4520	11
2455	68	CGTCGTCGAGG	776, 4417	11
2456	68	GATCGCCGACG	1415, 2490	11
2457	68	GCGTTCGCCGC	1019, 2098	11
2458	68	GCGTTCGCGGT	2363, 2911	11
2459	68	GGTTCGCCATC	2400, 3396	11
2460	68	GTCGGGGTCGC	88, 989	11
2461	68	TGTCGACCTCG	1006, 4585	11
2462	68	CAACGCCGGC	2248, 4243	10
2463	68	CACCGACGCG	3025, 4660	10
2464	68	CCGACCGGCG	2651, 2930	10
2465	68	CCGACGACCC	613, 3483	10
2466	68	CGACGACGAG	752, 2329	10
2467	68	CGACGGCGTC	3433, 4411	10
2468	68	CGCGACCGAC	2925, 3937	10
2469	68	CGGCGAACAA	826, 1913	10
2470	68	CGGCGCGCCG	1134, 3658	10
2471	68	CGGCGGGATC	2484, 3573	10
2472	68	CGTTCGGCGC	3266, 3654	10
2473	68	CTGCTCGAGC	3302, 3818	10
2474	68	GAACGCGCTG	3829, 4343	10
2475	68	GATCGCGATG	1601, 3853	10
2476	68	GATGTCGACC	175, 1004	10
2477	68	GCAGCTCGAT	368, 581	10
2478	68	GCGGGCGGGC	2068, 2735	10
2479	68	GTCGGCGACG	103, 1517	10
2480	68	GTGACGCCGA	745, 1695	10
2481	68	GTGATGCCCC	532, 1458	10
2482	68	TCGCCGCCTT	1810, 4106	10
2483	68	TCGGCGGCGC	867, 3690	10
2484	68	TCGGGAAGGG	1162, 1971	10
2485	68	TGATCAGCCC	934, 2385	10
2486	68	TGCTCGCGCA	4308, 4431	10
2487	69	TCTTGACCCCATACACCGAGTAATT	3653, 3678	25
2488	69	GCTGCTCATCGCGG	1791, 3735	14
2489	69	ATCGTGGCCGAGA	3352, 4284	13

2490	69	GTCGGCGATCAGC	2468, 3724	13
2491	69	ACCGCCTTGAGG	2442, 2868	12
2492	69	CGCGACCCGACC	3187, 4579	12
2493	69	GCGCTCCCAGGG	2284, 2300	12
2494	69	GGCGTTGACCGC	3144, 4718	12
2495	69	CGACCCGCCCG	1332, 1932	11
2496	69	CGCAGTTCAGC	1547, 3958	11
2497	69	CGCTGGCCGCG	4390, 4699	11
2498	69	GCCGATGGCCG	564, 861	11
2499	69	TCCGCGGCGCC	923, 2592	11
2500	69	TCGGCGCCGGC	607, 2562	11
2501	69	CCGACGCCGA	814, 1886	10
2502	69	CGCGGCCCGA	3778, 4597	10
2503	69	CGCGGCGCGC	892, 4484	10
2504	69	CGTGGAGGAG	1734, 1911	10
2505	69	CTGCCCGACG	172, 1168	10
2506	69	CTGGGCGCCG	1879, 3561	10
2507	69	GAAGGGCCAG	23, 1455	10
2508	69	GATGAGCTCG	3123, 3838	10
2509	69	GCGCGGCCCG	1007, 3777	10
2510	69	GGACCCGTTT	1587, 2581	10
2511	69	GGCGGGCGTT	2683, 2766	10
2512	69	GGCGTTCCTG	984, 3041	10
2513	69	GGGTCGGCTG	2957, 4451	10
2514	69	GGTCGCGGTC	1500, 2617	10
2515	69	GTGGCCTGGA	202, 4350	10
2516	69	TCGAACTCCG	3904, 4114	10
2517	69	TCGACATCGG	620, 2364	10
2518	70	CCCGACGCCGTCA	3369, 4428	13
2519	70	AGCGACCCGATC	2051, 2271	12
2520	70	ATCGCGTCGCTG	3627, 4108	12
2521	70	ACCGGACCATG	3448, 3862	11
2522	70	CACAGCCGACG	1161, 1326	11
2523	70	CGAAGAGCGTG	3659, 4071	11
2524	70	CGACGGTGTCG	2768, 3493	11
2525	70	CGCCGACGCGC	712, 3029	11
2526	70	CGTCGCCGGCG	2340, 2867	11
2527	70	TGCGCCGCGCG	287, 3322	11
2528	70	TGGGCTACCCG	3244, 3808	11
2529	70	ACCGGTGAGC	583, 4392	10
2530	70	CACCACGGTG	108, 1473	10
2531	70	CACCCGTGGC	342, 3099	10
2532	70	CACGCCATCG	2185, 4102	10
2533	70	CCGGCGTCGC	1969, 3169	10
2534	70	CGCGACGGTG	232, 3491	10
2535	70	CGTCGCGGAA	1622, 2595	10
2536	70	CTGGCCCTGA	1180, 1393	10
2537	70	GCCCTGACCG	481, 2516	10
2538	70	GGCCGAGGCC	1653, 3647	10
2539	70	GGCCGCAGCG	1614, 1820	10
2540	70	GTCGTCGCCG	2338, 2587	10
2541	70	TCGAACGCGA	227, 2363	10
2542	70	TTGGACAGGA	750, 3908	10
2543	71	CACGGTGCCGGGGA	301, 1032	14
2544	71	CTACCACGTCGAC	561, 2004	13
2545	71	GGGCACCGAGGAC	1326, 1689	13

2546	71	AGCGACGACGTC	1594, 2319	12
2547	71	CGCCGCGAGGTG	3579, 4474	12
2548	71	ACCGGCGGGTG	250, 1420	11
2549	71	AGGGCGTGCAC	3448, 3826	11
2550	71	ATCGCCCAAAA	2736, 4628	11
2551	71	CCGCACCGTCG	2193, 3614	11
2552	71	CCGCCGACCCC	665, 1610	11
2553	71	CCGCGGCGGAT	800, 1970	11
2554	71	CGCAGGGATGC	2755, 4602	11
2555	71	GCGGCTGCCCG	789, 2768	11
2556	71	GCTACACCGAT	1640, 3763	11
2557	71	ACCGGATCAT	1454, 2650	10
2558	71	ACGGCATTCC	1552, 2933	10
2559	71	ACGGCCAGCC	1394, 1993	10
2560	71	CAACGCCGAC	2846, 3332	10
2561	71	CCGCCGATCC	617, 932	10
2562	71	CCGCGCCGGA	3105, 4384	10
2563	71	CCGCGGGCAC	656, 1853	10
2564	71	CCTCGTCGGC	1710, 2060	10
2565	71	CGACCATGAT	4505, 4658	10
2566	71	CGACGGCGCC	1248, 1293	10
2567	71	CGCCAACCTG	3290, 3848	10
2568	71	CGCCGGGCGG	266, 482	10
2569	71	CGGATCAGCG	532, 3571	10
2570	71	GACACCTACC	130, 1906	10
2571	71	GATCAGCATG	4092, 4530	10
2572	71	GCCAACGCCG	466, 3330	10
2573	71	GCCCCGATCT	2889, 4237	10
2574	71	GCCGACGCCA	1468, 1651	10
2575	71	GCGGCTGCGG	783, 3971	10
2576	71	GCTCAACGGC	1389, 1547	10
2577	71	GGACACCTAC	129, 3776	10
2578	71	GGCGCCAACC	901, 3846	10
2579	71	GTCATGGCCC	4056, 4698	10
2580	71	TCTTCGGCGC	884, 3733	10
2581	71	TGCGCGGCTG	2870, 3958	10
2582	71	TGGTGGCCAA	461, 973	10
2583	71	TGTTCCAGGA	3046, 4084	10
2584	71	TTCAGCGACG	1243, 4713	10
2585	72	AGCTCGTCGACGTCG	4569, 4629	15
2586	72	TCACCGACGACGG	68, 3134	13
2587	72	CACCGGCGCGAT	2713, 4661	12
2588	72	CCGGCAGGTCCG	934, 4270	12
2589	72	CCGGCATCGCG	3868, 4686	11
2590	72	CGAAGTCCGAG	191, 1183	11
2591	72	CGAGCTCGACG	1015, 4195	11
2592	72	CGAGGTCCTCG	2190, 4147	11
2593	72	CGCCCGATCAA	521, 2400	11
2594	72	CGCCCGGCGGC	1621, 3742	11
2595	72	CGCGCCCGTCG	2800, 2959	11
2596	72	CGGCGGCATCG	279, 1586	11
2597	72	CGTCGCCGAGG	1949, 4495	11
2598	72	CTGGCCGAGCA	599, 1028	11
2599	72	GCCGCCGCTGG	1427, 3712	11
2600	72	GGGCGGCCAGG	697, 1408	11

2601	72	GTCCGCGGCGG	274, 620	11
2602	72	GTGGCCGAGGT	1635, 3887	11
2603	72	ACGGCCGGCC	5, 1867	10
2604	72	CAGCGCGGTG	551, 974	10
2605	72	CCCGGCCACC	539, 590	10
2606	72	CCCGGCCGCC	2600, 3310	10
2607	72	CCGAAGATCG	4239, 4443	10
2608	72	CCGACGAGCT	3405, 4674	10
2609	72	CCGCCGAGGT	481, 2186	10
2610	72	CCGGACGGCC	2030, 4226	10
2611	72	CCTGAACGGC	323, 333	10
2612	72	CGACGGCAAG	48, 3385	10
2613	72	CGATGCCGGG	1885, 3823	10
2614	72	CGCTCGCGGT	3702, 4485	10
2615	72	CGGCGACGGC	45, 1174	10
2616	72	CGGCTCGCCG	1895, 2768	10
2617	72	CGGTCCGCGA	2856, 3684	10
2618	72	GATGGCCGGC	1580, 3863	10
2619	72	GCCCACCGGC	4304, 4658	10
2620	72	GCCGACCCCG	18, 3566	10
2621	72	GCTGACGCCG	3613, 4430	10
2622	72	GGCCGTCGCC	171, 1946	10
2623	72	GGCGCCCTGC	1046, 1664	10
2624	72	GGTGACGATC	117, 4751	10
2625	72	GGTGGTCACC	3956, 4529	10
2626	72	GTCAGCGGGA	1610, 3798	10
2627	72	GTGTCCCGCG	1262, 4115	10
2628	72	TCGGCGGCCT	1365, 4347	10
2629	73	AAGCCCGAGCCGAAGCCCGA	4029, 4053	20
2630	73	GCCGCGCTTGCGATCGCCAC	1336, 1393	20
2631	73	CGACCCGCTGCGCCCGGCT	1315, 1373	19
2632	73	CCAAAGCCCGAGCCGAAG	4050, 4074	18
2633	73	CCGGCGCCCGCCC	1557, 4122	13
2634	73	GCCGACGTTGCCG	18, 123	13
2635	73	GGCCTCGTCGGC	413, 431	12
2636	73	GGCGGCGGCGGC	531, 2156	12
2637	73	CAACCAGCTGC	1583, 2315	11
2638	73	CCGGATCTGCG	2640, 3344	11
2639	73	CGCCGGCGCAG	3121, 4426	11
2640	73	CGCGCGGCTGC	2911, 3854	11
2641	73	CGCTGGCCAAG	2530, 4324	11
2642	73	GCCAGCACGTG	693, 4704	11
2643	73	GGCGTTGATCG	3332, 3521	11
2644	73	GGTGCGCAGCA	767, 4193	11
2645	73	TGCTGGAGCGG	3637, 3721	11
2646	73	TGGCCGGCGCC	3454, 4261	11
2647	73	ACCCTGACCC	2112, 3765	10
2648	73	ACCGTCCGGC	628, 2803	10
2649	73	ACCTTCGCCG	2214, 2814	10
2650	73	CACCACCGAG	3245, 4244	10
2651	73	CAGGCCGTCC	3669, 4644	10
2652	73	CCACCGCGGG	3175, 3958	10
2653	73	CCCGGCTCGG	3748, 3945	10
2654	73	CGACACCCGC	1634, 3086	10
2655	73	CGACCTGATC	1934, 3653	10
2656	73	CGACGCGGTC	2651, 3377	10

2657	73	CGCGACGGCG	1617, 3818	10
2658	73	CGCGCTGCTG	2678, 3893	10
2659	73	CGGCAGGTCG	2133, 4667	10
2660	73	CTACGGCGGC	1646, 1781	10
2661	73	CTTCGGCCTC	400, 427	10
2662	73	GAGCCTGCCC	1595, 4470	10
2663	73	GCCGCAGCGG	4185, 4660	10
2664	73	GCGCCGCATC	224, 4610	10
2665	73	GCGCGACAAG	805, 4217	10
2666	73	GGTTGTTCTG	2080, 4726	10
2667	73	GTCCGCGGCT	276, 4013	10
2668	73	GTGCGCCTGC	216, 2358	10
2669	73	TCGCCCCGCG	3417, 4680	10
2670	73	TCGCCGAGGC	4366, 4522	10
2671	73	TCGCGCTGGC	2404, 2527	10
2672	74	GAGCGGCGGCGCCG	2622, 3406	14
2673	74	GGCGCGGAACAGGT	2123, 2450	14
2674	74	GGCGATCAGCGCG	2003, 3110	13
2675	74	GGTGCTGGCCAA	3064, 4464	12
2676	74	ACGAACGGTGC	2049, 4526	11
2677	74	ACGACGTCGAC	230, 4736	11
2678	74	AGCAGCCGGTC	559, 2523	11
2679	74	CATGCCGGCGA	2354, 3760	11
2680	74	CGGTGTCCCAG	1756, 3609	11
2681	74	CGTAGTGGTGG	101, 716	11
2682	74	GCTGACCGCGG	42, 4455	11
2683	74	GGCCGCCGTCG	2722, 3453	11
2684	74	TGAAGACCACC	1930, 2976	11
2685	74	CACCATGCCG	528, 3757	10
2686	74	CAGCCGCAGC	541, 579	10
2687	74	CCAGGTCCGC	422, 2335	10
2688	74	CCCGACACGT	961, 3847	10
2689	74	CCGGGCCGGG	1876, 2952	10
2690	74	CCTGCGCCTG	617, 3393	10
2691	74	CGATCGGTGA	208, 895	10
2692	74	CGCACCCGCG	1707, 4615	10
2693	74	CGCCGGGCCG	511, 2950	10
2694	74	CGCTGACCGC	41, 2254	10
2695	74	CGGCATCGGG	641, 4383	10
2696	74	CGGCCGGTGG	1108, 3689	10
2697	74	CGGGCCGCCG	3451, 4274	10
2698	74	CGGTCAGCCC	347, 2404	10
2699	74	CGTCAAGGGC	3004, 4109	10
2700	74	CTCGACCAGA	63, 3241	10
2701	74	CTGCGCCGAT	1677, 4542	10
2702	74	GACGTGCAGG	2861, 4597	10
2703	74	GCCCGTTGCG	1603, 2218	10
2704	74	GCCGGCCTTC	570, 3183	10
2705	74	GCCGGTCAGC	177, 2402	10
2706	74	GCTCGATCCG	2071, 4659	10
2707	74	GGCGCGGGCG	972, 2993	10
2708	74	GGTGGCGCCG	486, 1014	10
2709	74	GTCGGCGCTG	1922, 3995	10
2710	74	TCGCGTCGGC	635, 3165	10
2711	74	TCGCTGACCG	40, 1869	10

2712	74	TCGTCGGCGC	1920, 4195	10
2713	74	TGCGGCGACC	2327, 2710	10
2714	75	ACACCGGACCCGATGGGCGCCGACCCGCTTCGCCCGGCT CCGCCGCGCTCGCGATCG	3887, 3944	57
2715	75	CCGATGGGTGCCGACCCGCTTCGCCCGGCTCCGCCGCGCT CGCGATCGACACCGGA	3782, 3839	56
2716	75	TCTGATCAGGCGCGAG	3654, 3670	16
2717	75	GCGGCCGACGTCATC	2035, 2341	15
2718	75	GCGGGCTGGGCCG	1889, 2708	13
2719	75	GGCAGCTCACCGA	684, 1967	13
2720	75	CTCAACCCCGGC	2831, 3326	12
2721	75	GAACGCGGTGTA	1362, 4593	12
2722	75	GACGAACGCGGC	557, 4110	12
2723	75	TCGCCGACATCG	2907, 3009	12
2724	75	AGTTCGCCGAC	126, 2904	11
2725	75	CCAGCGCGGCC	2234, 2336	11
2726	75	CCGGCGGCCAC	1495, 1999	11
2727	75	CGCCCAGCGCG	2499, 4097	11
2728	75	CGGAGAACGCG	4271, 4589	11
2729	75	CGTCGAATCCG	790, 4082	11
2730	75	GACCTCGCCGC	1950, 4302	11
2731	75	GACGCCGACGG	846, 2792	11
2732	75	GTGCTGGCCTC	3176, 3296	11
2733	75	TCCGACCCGCA	444, 3632	11
2734	75	ACGTCGTCGC	967, 2053	10
2735	75	AGCTGGCCCG	3054, 3195	10
2736	75	AGGGCCAGGC	2527, 4372	10
2737	75	CACCGCGCCC	830, 1809	10
2738	75	CCCCGGCGGC	1598, 1997	10
2739	75	CGACACGGCG	284, 2611	10
2740	75	CGACCTTCAT	3561, 4541	10
2741	75	CGGCGACGTC	962, 3208	10
2742	75	CGGGCCAGGC	735, 2092	10
2743	75	GACCGCCGCC	1740, 2875	10
2744	75	GACGGCCGCG	1043, 1731	10
2745	75	GAGACCTCGC	3470, 4300	10
2746	75	GCAGCAGGTC	652, 709	10
2747	75	GCATCGGCAG	670, 1832	10
2748	75	GCGCGCCGCT	629, 950	10
2749	75	GCGCTGGCCG	155, 3380	10
2750	75	GGCCGCACGC	241, 2945	10
2751	75	GGGTGGCGCT	3375, 4624	10
2752	75	GTGCACGGTC	2864, 3043	10
2753	76	TCGGCATCCCCGAC	2043, 3204	14
2754	76	CCACCGCGGTGGC	1701, 2193	13
2755	76	CCGCCGGCGCC	3378, 3622	11
2756	76	CGCAGGCCCGC	1503, 3012	11
2757	76	CGCGGCGCTGC	423, 1720	11
2758	76	CGCTGCACGTC	1054, 3842	11
2759	76	CGGCCGGGGTG	2754, 3875	11
2760	76	CGTCGTCGTCG	1339, 1951	11
2761	76	CGTGCCGCTGG	2806, 3118	11
2762	76	GACCGCAGCGA	1108, 2015	11
2763	76	GCCGACCCCAG	1778, 2675	11
2764	76	GCTGGTGCAGG	2728, 3046	11
2765	76	ACCGTCGTCG	1949, 2036	10

2766	76	CACCCGCTGC	1050, 3913	10
2767	76	CCAGGGCGTC	1642, 3804	10
2768	76	CGACAGCTTC	3789, 4194	10
2769	76	CGACGACCCG	3867, 4425	10
2770	76	CGACGTGCCG	2803, 3355	10
2771	76	CGAGTGGCCG	4479, 4615	10
2772	76	CGGATCCGGC	1286, 3407	10
2773	76	CGGCGCGCCC	472, 3331	10
2774	76	CTCGTCGGCC	2779, 3398	10
2775	76	CTTCGCCCGC	2833, 4678	10
2776	76	GACGTCGTCG	544, 1337	10
2777	76	GAGTTCCGCG	2633, 3546	10
2778	76	GATGGTGACG	538, 3996	10
2779	76	GCACGACGAG	2923, 4473	10
2780	76	GCTCGACGAG	1606, 2560	10
2781	76	GGCCGACCCC	2674, 3924	10
2782	76	GGCCGCGGCG	1682, 1717	10
2783	76	GGTCGCGATG	379, 1474	10
2784	76	GGTGGTGCAG	3966, 4464	10
2785	76	GTGTCGAGCT	909, 2954	10
2786	77	CCCGGTGGTCCCGG	879, 909	14
2787	77	GGGCGGCCCGGGCG	286, 986	14
2788	77	CGGCGGCCCCGGT	902, 953	13
2789	77	GCCGGGCCCGCCG	1663, 1783	13
2790	77	GCGGCTGGTCGAC	3206, 3379	13
2791	77	ATCGTGTTCATC	134, 714	12
2792	77	CCCGGGCGGCCC	803, 983	12
2793	77	CGCACCCGTCGG	1265, 4330	12
2794	77	CGGCGCCGACCC	2060, 2423	12
2795	77	GACGCCGCGGCC	1543, 3054	12
2796	77	CCAACTCGCCG	100, 2644	11
2797	77	CCCCGCCGCCG	614, 3874	11
2798	77	CCCGGTCCCGG	873, 945	11
2799	77	CCGCACCGCAG	2208, 4062	11
2800	77	CCGCGCCGCCA	637, 4720	11
2801	77	CCGCGGCGGCC	184, 399	11
2802	77	CCGCGGTGGTG	3004, 4010	11
2803	77	CCGGCCGTCGA	3508, 3527	11
2804	77	CCGGCGTCCGG	4248, 4584	11
2805	77	CGCCGGGCCCG	1782, 1995	11
2806	77	CGGCCACGTCG	3913, 4108	11
2807	77	CGGCCTGGGCC	2402, 4179	11
2808	77	CTCCAGCCGGC	2379, 2684	11
2809	77	CTTCACCGGCT	160, 2669	11
2810	77	CTTCGACGGCG	1647, 4474	11
2811	77	GACGCGGACGC	3355, 3367	11
2812	77	GGCCGCCGGCC	2320, 4048	11
2813	77	GGCGGGCACCG	1233, 1698	11
2814	77	GTCAGCCCGGC	1056, 1769	11
2815	77	TGGGGACCCGG	834, 867	11
2816	77	ACGCCGGGCC	1781, 2566	10
2817	77	AGCGTCCCGG	2982, 3906	10
2818	77	ATCACGGCCA	195, 2637	10
2819	77	CACGCAGGTG	1191, 1632	10
2820	77	CATCGCGGCC	3098, 3161	10

2821	77	CCACTGCCAC	773, 2186	10
2822	77	CCCGCACCCG	4095, 4328	10
2823	77	CCCGGTGGCG	2295, 3845	10
2824	77	CCTGCCCGCG	2339, 3928	10
2825	77	CGATGACCCG	2753, 3035	10
2826	77	CGGCGGCACC	274, 4309	10
2827	77	CTGGACAACG	1618, 3896	10
2828	77	GCCGGCACCT	3824, 4631	10
2829	77	GCCGGCGCTT	1123, 4352	10
2830	77	GCGGACCCAT	1334, 2728	10
2831	77	GCGGCGATGA	2523, 2749	10
2832	77	GCTGGGCGGC	2584, 2657	10
2833	77	GGACATCGCG	548, 3095	10
2834	77	GGCCCCTGGG	861, 1378	10
2835	77	GGTGCTCGAC	2243, 4677	10
2836	77	GTTCCGGTCA	1592, 3953	10
2837	77	TCGGGCTGCG	3112, 4694	10
2838	77	TGCTGCAGGC	1751, 3744	10
2839	77	TGTGCCGGCA	3408, 4628	10
2840	78	CGCCGCGACGGCGG	2666, 3134	14
2841	78	GGCGTCCAGGACGT	3224, 4647	14
2842	78	TTCGACGTCGACGC	910, 1270	14
2843	78	CCTGCGCGCGCG	771, 873	12
2844	78	CGACGTCGCAGG	246, 2423	12
2845	78	CGGCCCGCGCGG	2171, 3523	12
2846	78	CGTGCGGCGGGA	2758, 4580	12
2847	78	GCCTGCTCGCGG	1617, 3324	12
2848	78	GCTCGGCGACCG	138, 3968	12
2849	78	GGTGATCGACGC	1640, 2084	12
2850	78	AGCTCGGCGAC	3204, 3967	11
2851	78	CCGACCCGCGC	351, 748	11
2852	78	CGCCTGGCCGA	1823, 2382	11
2853	78	CGCGGCCAGCG	1348, 3257	11
2854	78	CGGTGCGCTCG	132, 1368	11
2855	78	GAGCGGCTGGT	1174, 4725	11
2856	78	GATGCTGGCGG	280, 2189	11
2857	78	TCGCCGCGCTG	1103, 1744	11
2858	78	ACCGCCGGCG	527, 1542	10
2859	78	CACCGGCTCG	1787, 3399	10
2860	78	CACCTGACCG	1009, 1998	10
2861	78	CACGCCTTGG	1800, 4089	10
2862	78	CATCTCGGCC	2900, 3441	10
2863	78	CATGGCCGGC	1589, 4671	10
2864	78	CCCAGCGCCA	2463, 4198	10
2865	78	CCGCGGCGCG	2854, 2959	10
2866	78	CCTGGTCAAG	1158, 3098	10
2867	78	CGACGGTGGC	2272, 3034	10
2868	78	CGATGACCCC	543, 3545	10
2869	78	CGCGGCCCTC	2992, 3536	10
2870	78	CGCGGCGGCC	343, 3453	10
2871	78	CGGCTGCTGC	856, 1914	10
2872	78	CTCGGCCTCG	4515, 4542	10
2873	78	CTGGGCGGCC	2394, 3182	10
2874	78	GACCGAGGTG	1416, 4047	10
2875	78	GACCGCCGGC	1541, 1908	10
2876	78	GACGGTGTGG	406, 446	10

2877	78	GCACGCCCGC	1578, 1661	10
2878	78	GCCGCGGCCA	943, 3255	10
2879	78	GCCGGCCTTG	2696, 4503	10
2880	78	GCCGGGAGCT	2062, 2203	10
2881	78	GCGCAGCATC	3435, 3777	10
2882	78	GCTGCGCAGC	106, 3774	10
2883	78	GCTGGCCGAC	1556, 2417	10
2884	78	GGCCGCCGCG	939, 3131	10
2885	78	GGCGCTGGGG	2102, 2336	10
2886	78	GGCGGCGCCG	2237, 4497	10
2887	78	GGGGTCGACG	1056, 3576	10
2888	78	TCCGCCCAGA	1114, 4156	10
2889	78	TGGTGGCCGC	123, 3127	10
2890	79	GAGAATGTCGACG	673, 1836	13
2891	79	GCCGCTCGGCGAG	2174, 3276	13
2892	79	GGCCGTCGTCGTC	899, 2972	13
2893	79	GACCAGCACGCG	276, 985	12
2894	79	GGCCACGGCGGC	2922, 4381	12
2895	79	ACCGCGGCGGC	3013, 3195	11
2896	79	CCGAAACCGGG	2278, 2455	11
2897	79	CCGAACCCGAA	2314, 2443	11
2898	79	CCGGGACCGAA	2266, 2308	11
2899	79	CCGGGGTCCGC	1939, 3520	11
2900	79	CGCGCCCGCGG	2960, 4040	11
2901	79	GCACGTCACCG	780, 2207	11
2902	79	GCCGACGGCCG	586, 1911	11
2903	79	GCCGGGCGGGT	164, 3996	11
2904	79	GCGACGCCGCC	692, 3468	11
2905	79	GCGACGGTGCC	2829, 3268	11
2906	79	GGCGATCTGCT	1401, 2139	11
2907	79	GGCGCCGCGCC	2250, 2399, 4035	11
2908	79	GTTCTGGGCCG	1611, 3678	11
2909	79	ACGTGGTGCG	69, 1541	10
2910	79	CACCGGCAAT	1050, 1491	10
2911	79	CCGCGGATTC	1442, 2039	10
2912	79	CCTGGCGCCG	441, 2396	10
2913	79	CGAGACGACG	486, 1563	10
2914	79	CGGTGTGCGC	53, 1452	10
2915	79	CGTCGTCGGA	3690, 4224	10
2916	79	GGCCCGGGCC	1383, 2228	10
2917	79	GGCGGAAGCG	1866, 3669	10
2918	79	TCGAGCACGA	335, 371	10
2919	79	TGGCCCGCCA	320, 1250	10
2920	79	TGGTCGCCGA	1556, 1663	10
2921	80	CAGCAGCACCACCAGCG	678, 3043	17
2922	80	CAGCAGCGCCTCGCG	2615, 3445	15
2923	80	CGCGGCCAGCAGCAG	591, 888	15
2924	80	GAAAGCCGTTGCCG	1614, 3978	14
2925	80	GCGCCGGGGCGCGG	3692, 3784	14
2926	80	CCAGCTGCAGCGC	1912, 2455	13
2927	80	CGGCCGGCGGCCA	553, 2007	13
2928	80	CACCGCCGCGGC	534, 4223	12
2929	80	CGCGGCCGCGCG	1402, 2908	12
2930	80	CGGCGACCAGCA	1978, 3036	12
2931	80	GCGTCGTACACC	526, 2385	12

2932	80	GGGCAGCAGGCC	636, 1158	12
2933	80	ACCTCCACGCC	454, 3431	11
2934	80	CCGCGGCCAGC	590, 927	11
2935	80	CCGGCGTCGCG	3623, 4030	11
2936	80	CTCGGCGCGCA	2600, 4596	11
2937	80	CTCGTCGGCGA	1973, 2321	11
2938	80	GCTGGGCGGCC	1419, 4181	11
2939	80	GGATCAGCGCG	3285, 3555	11
2940	80	GGGCGGTCGCC	1885, 3167	11
2941	80	GTCGTCGGGGG	1823, 2756	11
2942	80	TCGAGCGCACG	1085, 2720	11
2943	80	TCGCTCGGCCG	510, 4495	11
2944	80	ACCCTCGACG	2956, 3130	10
2945	80	ACCGAGCCGG	1503, 3362	10
2946	80	AGGGCGGTCG	655, 1884	10
2947	80	ATCGGCTTGT	2660, 4414	10
2948	80	CACCAGCAGG	1599, 3745	10
2949	80	CAGCGTGCAA	3207, 3388	10
2950	80	CCACCAGCAG	1598, 3477	10
2951	80	CCAGCGCCAC	1592, 3766	10
2952	80	CCGGCCCCGC	41, 2026	10
2953	80	CGACCAGTCC	915, 1667	10
2954	80	CGATCCAGCT	2148, 3072	10
2955	80	CGCGGTGCCG	364, 4023	10
2956	80	CGGCAGCAGC	2872, 3442	10
2957	80	CGGTCAGGTA	3528, 3708	10
2958	80	GACGGCCAGC	1587, 3736	10
2959	80	GCGAGCGCGG	415, 883	10
2960	80	GCGCCGCGCG	1059, 4248	10
2961	80	GCGGCAGCAC	626, 1226	10
2962	80	GCGTCGACGA	445, 778	10
2963	80	GCTGATCGCC	1322, 1364	10
2964	80	GGCCGCCGCC	4068, 4131	10
2965	80	GGCCGGGGCG	3012, 3876	10
2966	80	GGCGCGCCGC	1056, 3570	10
2967	80	GGTGGCCGTG	89, 104	10
2968	80	GTCGAGCACG	996, 1730	10
2969	80	GTCGCCCGGC	29, 3863	10
2970	80	TGTCGCCGTG	2890, 4305	10
2971	81	GGCGGGCCGTATTGAGG	845, 863	17
2972	81	CCGTAGCCCGGCGGC	716, 752	15
2973	81	AGGCCGCCACCG	1481, 1870	12
2974	81	CCGACGAGGAGG	449, 1232	12
2975	81	CGACGGCCGGTT	1895, 3566	12
2976	81	CGCGCGCACGAC	2310, 4723	12
2977	81	GGACAAGCTGCG	1146, 3282	12
2978	81	ACGCCGAGGCC	940, 1337	11
2979	81	CAACGAGCACG	1329, 1937	11
2980	81	CACCCAGAACG	1299, 3720	11
2981	81	CCGACCGGTTC	1556, 4465	11
2982	81	CCGCGGCGATG	1127, 3580	11
2983	81	CGAGCCCGCCG	3186, 3702	11
2984	81	CGCGGCGCTGC	232, 3105	11
2985	81	CTCGCCGGTGG	517, 2238	11
2986	81	GACCTCTACAC	1809, 2957	11
2987	81	GCCCGACGATG	399, 1831	11

2988	81	GCCGCCCGCGG	339, 2885	11
2989	81	GCCGGGTGATC	2251, 2754	11
2990	81	GCGCGGCGCTG	2796, 3104	11
2991	81	GGCCGACGGCG	1509, 3066	11
2992	81	GGCGGCGGCGC	920, 2901	11
2993	81	TGCCGACCCCG	2365, 4022	11
2994	81	ACACCGCCCG	390, 2739	10
2995	81	ACCGGCATTG	1031, 1041	10
2996	81	AGCACCACGG	158, 218	10
2997	81	AGGTGGCCGA	1391, 1424	10
2998	81	CAGCGGGATG	205, 688	10
2999	81	CGAATCCGCC	1281, 4007	10
3000	81	CGACGCCGTC	472, 3303	10
3001	81	CGGCCCGGTG	4269, 4403	10
3002	81	CGGGCGGCGG	894, 2899	10
3003	81	CGGGTAGACC	832, 1803	10
3004	81	GAACTGATCG	2591, 4342	10
3005	81	GAAGACGTCG	2412, 4303	10
3006	81	GACCGCGGCG	1125, 4642	10
3007	81	GACGTCCCGG	2097, 4281	10
3008	81	GCAGTGGTTG	4240, 4293	10
3009	81	GCCGGGTGGG	775, 793	10
3010	81	GGAAGCCGGA	2483, 4062	10
3011	81	GGCGCCCAGG	484, 3454	10
3012	82	CGGGTTCGATCG	466, 511	12
3013	82	GCGGCGGCCGGC	1785, 3564	12
3014	82	ACTCGTCGAAC	289, 2344	11
3015	82	AGCCGCGGCCG	494, 3956	11
3016	82	CAGCGTCAGCG	1958, 4649	11
3017	82	CCAGGCCGATC	2593, 4633	11
3018	82	CGAGCGGTGCA	131, 386	11
3019	82	CGATGTCGGCG	1162, 3869	11
3020	82	CGGCGCGCAGC	568, 1195	11
3021	82	CGTCGACGATG	4076, 4365	11
3022	82	GCCAGGTCCCC	1173, 2097	11
3023	82	GCCCGGCAAAC	850, 3810	11
3024	82	GGCGGTGACGT	3388, 4357	11
3025	82	TCGCCGTGGCG	2544, 4001	11
3026	82	ACCATCGCGA	1296, 4238	10
3027	82	ACCGCGACCA	2121, 2307	10
3028	82	ACGCGCTCCA	555, 4400	10
3029	82	AGCCGCTCGG	3510, 4572	10
3030	82	AGCGCGGCCT	3606, 4448	10
3031	82	ATGGTCGATG	807, 4095	10
3032	82	CACCATCGCG	1295, 3133, 4125	10
3033	82	CAGATCGGCG	239, 3382	10
3034	82	CAGCACCGCG	1889, 2117	10
3035	82	CAGCGAGCGG	383, 4321	10
3036	82	CCACCGCCAC	301, 2386	10
3037	82	CCATCAGGCG	331, 3794	10
3038	82	CCCGTCGGTG	1451, 3238	10
3039	82	CCGAACAGCG	144, 3600	10
3040	82	CCGGTGAGCA	1584, 3471	10
3041	82	CGACGACGAT	1819, 4106	10
3042	82	CGAGATGCCC	2533, 3632	10

3043	82	CGCGCGGAAC	1755, 4384	10
3044	82	CGCGGTCAGC	230, 2174	10
3045	82	CGCGTCGACG	191, 4712	10
3046	82	CGGTGTCCAG	1360, 1639	10
3047	82	GCAGCACCGC	1717, 1888	10
3048	82	GCGGGCGATT	1406, 3984	10
3049	82	TCGTAGCGCA	1902, 4415	10
3050	83	CGTCCAGCACCCG	260, 4409	13
3051	83	GACGCTGCGCAC	1716, 4143	12
3052	83	GGTGACCGTGAT	2939, 4347	12
3053	83	TGGCGTTGCTGG	1583, 4270	12
3054	83	ATGCCGACGAC	346, 613	11
3055	83	CGAAGACGGCG	1929, 4034	11
3056	83	GACGGTGGGGT	1455, 1647	11
3057	83	GCCGCGGCGTG	1895, 3078	11
3058	83	GGCGGCGTCCG	2574, 3525	11
3059	83	TGCCGGCTGGT	3693, 4339	11
3060	83	AGCACCGGCG	142, 3486	10
3061	83	CAACTCCGCG	1756, 1837	10
3062	83	CACCGGGTTC	1081, 1377	10
3063	83	CCCAGGACAA	831, 2806	10
3064	83	CCTCCTGCTG	170, 3203	10
3065	83	CGACGTCGAA	654, 2385	10
3066	83	CGAGGTCGTC	2020, 4208	10
3067	83	CGCCACCACG	2305, 3609	10
3068	83	CGCCTCCTGC	168, 4362	10
3069	83	CGGCGGTGGA	1011, 2285	10
3070	83	CGGTGAGCAG	506, 4466	10
3071	83	CGTCGCGGAG	326, 973	10
3072	83	GACCACCACT	21, 2762	10
3073	83	GATGCGGCGG	528, 2281	10
3074	83	GCCCCGGGCA	2150, 4372	10
3075	83	GCGCGCCGCC	2333, 4386	10
3076	83	GCGCTGACCG	1181, 1600	10
3077	83	GCGTTCGGAC	387, 3792	10
3078	83	GGCCGGCACC	2852, 3677	10
3079	83	GTCGGCCGCG	1551, 4428	10
3080	83	TCGAGCACCT	439, 2051	10
3081	83	TCGTCGCCGC	2504, 3436	10
3082	83	TGCAGCACCG	139, 2995	10
3083	83	TGGTCATCGA	996, 3907	10
3084	83	TGTATCCGAC	1161, 2789	10
3085	83	TTTCCGGGTC	3598, 4230	10
3086	84	TGCGGTCGACCAC	370, 2961	13
3087	84	CAGGCCCGCCAC	1624, 2512	12
3088	84	CGGTCCAGCACC	1135, 1490	12
3089	84	GATCTCGCGCAG	2026, 4126	12
3090	84	GGCGTTGATGCC	3388, 3994	12
3091	84	AGCGGCTCGCC	51, 1712	11
3092	84	CACCTCGCCCT	2416, 4462	11
3093	84	CCGGGACGGCC	355, 1851	11
3094	84	CGCGATGGTGT	1561, 4417	11
3095	84	CGCGGCGGCGA	332, 1609	11
3096	84	CGGCGGCGGCG	2209, 2326	11
3097	84	GCGCCCGGGCC	115, 4700	11
3098	84	GCTGGCCGGGC	1549, 1947	11

3099	84	GGGCACCTCGA	3373, 4384	11
3100	84	GTCGTCACCGC	2936, 4448	11
3101	84	ACGCTCGAAC	98, 2084	10
3102	84	AGCGGTCCAG	1383, 1488	10
3103	84	CACCGCGACG	839, 1358	10
3104	84	CAGCGTCAGC	1258, 4108	10
3105	84	CAGGGCCGCG	863, 3412	10
3106	84	CCAACGCGTC	2862, 3255	10
3107	84	CCCGCCGCGC	2825, 4240	10
3108	84	CCGCCGTGGG	786, 1439	10
3109	84	CCGGCGCCCC	302, 2310	10
3110	84	CGCCCGATCG	549, 3754	10
3111	84	CGCGCCCCAC	266, 1351	10
3112	84	CGGCACCGGC	2103, 2305	10
3113	84	CGGCGCTGCG	1090, 4397	10
3114	84	CGTCGACGTC	1036, 3501	10
3115	84	CGTCGCGGTC	457, 1230	10
3116	84	GCCCGCCAGC	3955, 4273	10
3117	84	GCCCTTGGCC	3328, 3352	10
3118	84	GCCGAACGAG	4033, 4048	10
3119	84	GCCGCGGGGC	1541, 3778	10
3120	84	GCCGGCGACG	2077, 4537	10
3121	84	GGGGTTGGTG	2704, 3913	10
3122	84	GTCGGAGGCG	1477, 3988	10
3123	84	TCCGGGACGG	354, 600	10
3124	84	TCGGCGTAGG	468, 3155	10
3125	84	TGCCCTTGGC	3351, 4428	10
3126	84	TGGGTGAGGT	1655, 3243	10
3127	84	TGGTCGGCGA	2456, 4316	10
3128	84	TTGACGAAGT	2441, 3095	10
3129	85	CGCGCTTCGCGCGCGCTTACCTCGCTCGG	4331, 4385	29
3130	85	GCCCGGCGAGCG	887, 4242	12
3131	85	GCGCGGCCCGCG	2400, 3572	12
3132	85	CAGCACGTCGC	2336, 2933	11
3133	85	CCCGGCCGGGC	2754, 4628	11
3134	85	CTGCTCGGCGA	3322, 4710	11
3135	85	GCCCGCAGCGC	625, 4744	11
3136	85	TGGCGGTCTGC	734, 2128	11
3137	85	CAGGAATGCG	426, 2444	10
3138	85	CCAGCCCGGC	884, 1936	10
3139	85	CCAGCCGCTT	1211, 1541	10
3140	85	CGAGCTGGTC	3590, 3782	10
3141	85	CGCCAGATCG	1985, 4134	10
3142	85	CGCCGCGCTG	3644, 4001	10
3143	85	CGGCCGGTAG	205, 842	10
3144	85	CGGCGGCGCG	247, 1592	10
3145	85	CGTCGACGTC	1960, 4157	10
3146	85	CTGACCGCCG	2483, 3891	10
3147	85	GCCGCCAGCG	1483, 2604	10
3148	85	GCCGCGGTGG	1615, 4470	10
3149	85	GCCGGCCAGC	2697, 3088	10
3150	85	GCGCACCGGC	3725, 4065	10
3151	85	GGTCTCGGTC	1880, 3506	10
3152	85	GTCGATCGCC	828, 2462	10
3153	85	GTCGGAGATC	1784, 4431	10

3154	85	GTCGGCCAGG	1677, 4656	10
3155	85	GTTGCGCAAT	81, 2548	10
3156	85	TCACCGTCTC	2308, 4502	10
3157	85	TCGGCGGTCG	2496, 3364	10
3158	85	TGCAGCAGCT	2145, 4609	10
3159	86	TGGTGGTGGCGCTG	30, 1728	14
3160	86	CGCCCGACCGGGT	1192, 3871	13
3161	86	CCCGAGCTGGCC	3582, 3759	12
3162	86	CGGCGACCGATC	137, 1794	12
3163	86	GGGGGTGTTGCT	3299, 3323	12
3164	86	GTCGACGTCGCG	1967, 3981	12
3165	86	CCATTTCGCGC	1380, 4750	11
3166	86	CCGCGACGCCG	1828, 2580	11
3167	86	CGCCCCTGCGG	2381, 4499	11
3168	86	CGGCCCGCCCG	2622, 2637	11
3169	86	GCCCGCGATGG	2050, 4480	11
3170	86	GCGGTCCGGGG	3263, 3597	11
3171	86	GGCCGGCCCGG	3012, 4389	11
3172	86	GGCGCTGGTCG	938, 3147	11
3173	86	TGCGCCGCCGA	1946, 2036	11
3174	86	ACCCCGGCCA	774, 4107	10
3175	86	CACCACGCCG	1041, 2692	10
3176	86	CCACCGACGA	2735, 3116	10
3177	86	CCCGGCGCCG	2498, 4163	10
3178	86	CCGCCCTGCG	2202, 2759	10
3179	86	CCGCCGACGA	1276, 4312	10
3180	86	CGACGCCGGC	905, 1112	10
3181	86	CGCCTTCATC	2952, 3236	10
3182	86	CGCTGGCACA	998, 4595	10
3183	86	CGGCCGCCGG	1509, 3000	10
3184	86	CGTCAGCAGG	106, 2796	10
3185	86	CGTCGACGAC	2475, 4280	10
3186	86	CGTGCTGGCG	2520, 3224	10
3187	86	GACCGCGCCG	259, 1165	10
3188	86	GCCCGCGACG	2227, 2578	10
3189	86	GCCGCGCCGC	1609, 3360	10
3190	86	GCGCGGCGTG	535, 1927	10
3191	86	GGCCACCGCC	695, 2754	10
3192	86	GGTGCGCGCC	2025, 2946	10
3193	86	GGTGTTGGAG	3707, 3842	10
3194	86	GTCAACGAGG	2908, 3627	10
3195	86	TCCGGCCCGC	2608, 2620	10
3196	86	TCGACACCGC	6, 657	10
3197	87	ACGGCGAGCGCCGCATGGCGCGAGTGAGGAGCCGGGCAA TCGGGCCTAGCCCGGCG	4068, 4124	56
3198	87	CGGCGTGGCCGACCA	235, 2369	15
3199	87	GGTCGTCGACGATC	2255, 2663	14
3200	87	CAGCTCCTCGCC	2649, 3930	12
3201	87	GCGCCGACCACC	768, 2341	12
3202	87	TCGCCGGCGGCG	609, 4544	12
3203	87	ACGCCGCCGCC	1422, 4391	11
3204	87	CCAGGTGTCGA	824, 2778	11
3205	87	CGCCGTCGGCG	229, 458	11
3206	87	CGCGCAGGTCG	352, 2249	11
3207	87	CGGCGTCGCGG	478, 3917	11
3208	87	CGTCGATGCGG	556, 2438	11

3209	87	CTGGGGGGCGG	1451, 1792	11
3210	87	GATCAGCGGCA	1708, 3591	11
3211	87	GCCGACACCGT	4435, 4452	11
3212	87	GTCAACGGCGA	1475, 4735	11
3213	87	TCAGGTCGCCG	1009, 1573	11
3214	87	TCGGCGAATCG	2515, 3955	11
3215	87	AGGCCAGCCG	1196, 3899	10
3216	87	CAGCGTGGAG	128, 977	10
3217	87	CAGGTAGCCG	2604, 3174	10
3218	87	CCGAATCCGG	725, 4282	10
3219	87	CGTAGCCGCG	316, 346	10
3220	87	CGTCGCGGGT	40, 931	10
3221	87	GCCCAGCAGG	213, 749	10
3222	87	GCCCGGCCGT	283, 4238	10
3223	87	GCCGAATCCG	425, 4281	10
3224	87	GCCGCACCAC	1761, 3356	10
3225	87	GCCGCGCAGC	389, 2568	10
3226	87	GGCCGACACC	179, 4434	10
3227	87	GGGCTTGCCG	3207, 3432	10
3228	87	GTCGGACCAG	3831, 4467	10
3229	87	GTCGTCGCGG	38, 1145	10
3230	87	TCGACGACGT	501, 3295	10
3231	87	TCGCCGAACC	2705, 3805	10
3232	87	TTGTTCGGCG	3249, 3951	10
3233	88	CGTCGTGACGGTG	43, 3307	13
3234	88	GTGCTGGGCATCG	2248, 2981	13
3235	88	ACGCCGCGCTGG	2444, 3543	12
3236	88	CCTACGCCGACC	1790, 3721	12
3237	88	CGGCGGTGTGCT	1095, 3430	12
3238	88	CTGGGTGCGCTG	3371, 3885	12
3239	88	GCCGCCGCGATC	3852, 4592	12
3240	88	TCATCGCGATCC	3066, 4024	12
3241	88	ATGCGTGCGAT	1012, 4691	11
3242	88	CCTGCTGCCCG	2734, 3743	11
3243	88	CGACTACGTGC	3262, 4103	11
3244	88	CGCGGTGCGCG	4088, 4507	11
3245	88	CGCGGTGGCCA	2223, 4193	11
3246	88	CGGTCATCGCG	4021, 4186	11
3247	88	CGTCGCCGTCG	3674, 4549	11
3248	88	GCGGCGGGGTC	711, 789	11
3249	88	GGCCAGGCCGC	616, 4499	11
3250	88	GGCGGGCGAAT	1183, 4622	11
3251	88	TGCTGATCTAC	2712, 3501	11
3252	88	TTCACCGCCCG	972, 2050	11
3253	88	ATCGTGACCG	3395, 3485	10
3254	88	CATGAGCGCG	1608, 4441	10
3255	88	CCCGGATATC	731, 1717	10
3256	88	CCGCGGCCGA	525, 2468	10
3257	88	CCGGCGGGCG	4620, 4635	10
3258	88	CCGGTCGTCG	574, 1048	10
3259	88	CCTGGACACC	1779, 1914	10
3260	88	CGACCGCCGA	2456, 4678	10
3261	88	CGACTTCAGG	985, 1545	10
3262	88	CGCCACCGCC	2325, 3277	10
3263	88	CGCGATCGCC	2178, 2758	10

3264	88	CGCGCCGGAT	2338, 2667	10
3265	88	CGTCGAGGGC	909, 4423	10
3266	88	CGTGGTGCGG	3217, 4074	10
3267	88	CGTTCATCGT	2832, 3667	10
3268	88	GCACGCCGAC	1463, 3256	10
3269	88	GCCCTGGCCA	122, 4356	10
3270	88	GCGGTCGCCG	1438, 2789	10
3271	88	GCTGATCGCG	3001, 3659	10
3272	88	GGAGCGCCGC	814, 2269	10
3273	88	GGCCGCCATC	1983, 3974	10
3274	88	TCAGCCGGCC	115, 879	10
3275	89	CTGGCGATCGCCG	639, 2054	13
3276	89	GGATTGACGACGC	742, 2723	13
3277	89	CCGTCCTCGACG	1491, 2894	12
3278	89	GCGCTCGAGGGC	1586, 2689	12
3279	89	CCGAACTGGGC	280, 1117	11
3280	89	CCGCGCCGGCG	445, 2568	11
3281	89	CTGTCCGGCGG	138, 975	11
3282	89	GCCCGCAACAC	409, 2301	11
3283	89	GCGCGCGGTCG	472, 4750	11
3284	89	GCGCTGGCCGG	321, 4390	11
3285	89	GCGGTCGCGCA	1796, 2641	11
3286	89	GGCCGCGGCCG	1666, 3934	11
3287	89	GGCCTCGGCGA	2220, 3034	11
3288	89	GTCCGTCGTCG	344, 3725	11
3289	89	CAACCCGCGG	1940, 2710	10
3290	89	CAGCGTGCTG	3262, 4471	10
3291	89	CCGCCGCCGA	1452, 4305	10
3292	89	CCGTCGTCGG	942, 1608	10
3293	89	CCTGCCCCGC	962, 4573	10
3294	89	CGAGCGCTGG	1011, 4045	10
3295	89	CGCCCGGGTC	1245, 2365	10
3296	89	GACGAGGCGG	504, 1047	10
3297	89	GCCAGCGTGC	154, 3260	10
3298	89	GCCGCCGCCG	2521, 4304	10
3299	89	GCCGGTGCAC	3112, 4516	10
3300	89	GCGACGCGGC	1956, 3966	10
3301	89	GCGCCCGCAT	2998, 3135	10
3302	89	GCGCGCGGTG	65, 1858	10
3303	89	GCGCTGCGGG	1466, 4406	10
3304	89	GCGGAGCCCG	404, 3896	10
3305	89	GCGGCGCAGC	578, 2545	10
3306	89	GCGGCTCACC	269, 4420	10
3307	89	GCGTTGCGGG	27, 3709	10
3308	89	GGCAGCGCGC	61, 895	10
3309	89	GGCCGCCCTG	4171, 4603	10
3310	89	GGCCGCCGTG	3307, 4120	10
3311	89	GGCGGCCGCG	3931, 4030	10
3312	89	GGGCGCGTGG	1999, 3686	10
3313	89	GGGCGGCCGC	4029, 4116	10
3314	89	GGGTGGCGGC	3927, 4342	10
3315	90	CGGTGGCCGCCGCGG	609, 3139	15
3316	90	ACCGGCGCGGGCA	338, 767	13
3317	90	CCGCGCGGGCGG	148, 3593	12
3318	90	CCGGCCGGCGGC	510, 1197	12
3319	90	CGCCCGGCTGGC	736, 3104	12

3320	90	CGGCGTTCGGCG	2527, 2873	12
3321	90	CGGTGCGCCAAC	896, 930	12
3322	90	CGGTGGTGGCCG	246, 2702	12
3323	90	CTGGCCGCCCTG	1972, 3418	12
3324	90	GGCGCTGGGCGC	1102, 2430	12
3325	90	GGCGGCGCGGGC	1046, 1737	12
3326	90	GGTGCTGCCGCC	137, 598	12
3327	90	TTGCTGGCCGCG	788, 2173	12
3328	90	ACATCATCGGC	726, 2936	11
3329	90	CCGCGGCGACG	1402, 3119	11
3330	90	CCGGCGGGCGA	3611, 4699	11
3331	90	CGACGGCCCGG	2261, 4383	11
3332	90	CGCGGTGCGGC	472, 580	11
3333	90	CGGCAACGGTT	427, 1909	11
3334	90	CGGCGCGGTCG	1536, 1707	11
3335	90	CGGCGTCGCCG	2562, 3354	11
3336	90	CGGTGACGTGC	2721, 3731	11
3337	90	CGTGGCCGCCG	703, 1686	11
3338	90	CTCGACGTGCT	1630, 2311	11
3339	90	CTGCGGGCCGG	1651, 2332	11
3340	90	GACCTGCTGGC	1013, 2422	11
3341	90	GCCGCCGGCCG	506, 814	11
3342	90	GCTCGTCGTCG	3020, 3659	11
3343	90	GCTGCGGCGCG	1306, 3433	11
3344	90	GCTGGCCGCCG	502, 1776	11
3345	90	GGACGACGCGC	523, 1291	11
3346	90	GGATCCGTTGG	2286, 3980	11
3347	90	GGGTCGACGAC	417, 3503	11
3348	90	TGCGCGCGACG	1415, 4094	11
3349	90	ACTCGGCGAG	56, 1210	10
3350	90	AGCGGCTGGC	210, 492	10
3351	90	CAGCCCCGCG	3642, 3690	10
3352	90	CAGGTGCTGC	1166, 4147	10
3353	90	CCCGCCGGCG	3165, 3607	10
3354	90	CCCGGTGCGC	928, 1668	10
3355	90	CCGCCGAATT	849, 3799	10
3356	90	CGCCACGACG	2590, 4596	10
3357	90	CGCGCGGCCG	2481, 2800	10
3358	90	CGCGCGGTGC	892, 3945	10
3359	90	CGCGCTGCTG	1359, 2508	10
3360	90	CGGCCGCCGC	2354, 2615	10
3361	90	CGGTGGCGGC	112, 1733	10
3362	90	CGTGCACGCC	1060, 4400	10
3363	90	CTGGGCATCG	2689, 3006	10
3364	90	GACCGCATCG	971, 2113	10
3365	90	GCGCGCGACC	1593, 1953	10
3366	90	GCGGTGGCCG	3138, 3406	10
3367	90	GCTCGCGCTG	1374, 1824	10
3368	90	GCTGCGGCCG	646, 1154	10
3369	90	GGCGCTCGCG	1371, 2253	10
3370	90	GGGTGGCGTG	1466, 2129	10
3371	90	GGTCGCCACC	3209, 4314	10
3372	90	GTCGAACGCG	69, 1181	10
3373	90	GTCGACGGTC	2835, 3526	10
3374	90	GTGGGAGCGG	1815, 4684	10

3375	90	TCGCCCGCGC	3488, 4043	10
3376	90	TCGCCGCGGC	1399, 3271	10
3377	90	TGCCGGCGCG	2234, 2978	10
3378	90	TGCTGGCGGC	28, 1834	10
3379	90	TGGGTCGACG	416, 539	10
3380	91	CCGCGCCGCCCGGA	167, 1081	14
3381	91	GCCGCCGCGCTGGC	3074, 3223	14
3382	91	CCGCCCAGCGGGC	1748, 2797	13
3383	91	CGCAGGATCGAC	1768, 2411	12
3384	91	CTCCCGCGCGAA	567, 1821	12
3385	91	GCGGTCGCAGCT	258, 2378	12
3386	91	TCACCGTCGGCG	1613, 3009	12
3387	91	CGACGTCGGTG	707, 872	11
3388	91	CGCGCCCTGGC	1071, 3061	11
3389	91	CGGTGCCGGTG	539, 2051	11
3390	91	GCAGCAGCCCG	281, 842	11
3391	91	GCCACCGGACG	3511, 4468	11
3392	91	GCGCGACCGCA	1359, 4335	11
3393	91	GGCACCCGCGG	77, 3389	11
3394	91	GGTCCTCGGCG	593, 1250	11
3395	91	GGTGGTGGCCA	390, 1194	11
3396	91	TCGCCGCGGTC	253, 1910	11
3397	91	AGCGCGACCG	4334, 4540	10
3398	91	CCAGCGCGGT	1238, 1334	10
3399	91	CCAGGGCGCG	1226, 2118	10
3400	91	CCCGAGGGTG	3284, 3570	10
3401	91	CCCGCGGTCG	2133, 2960	10
3402	91	CCGGCGACTC	2467, 3765	10
3403	91	CGACCCGCAG	1931, 4404	10
3404	91	CGCGCCGGCC	15, 4613	10
3405	91	CGCGGCGCGG	1350, 3203	10
3406	91	CGGTGCCCGG	696, 1124	10
3407	91	GACGTCGACG	4018, 4638	10
3408	91	GCCGCCGCAG	836, 3426	10
3409	91	GCGGCGGCAA	2263, 3746	10
3410	91	GGCGATGTCG	1164, 1305	10
3411	91	GGCGCCCAGC	1329, 4570	10
3412	91	GTGTCCAGCG	628, 1033	10
3413	91	GTTCCGGCGA	2251, 2464	10
3414	91	TTGTCGAGCG	25, 3542	10
3415	92	GGCCAGCTGGGTGG	1834, 2305	14
3416	92	CGCCCAGCGCGGT	2628, 2892	13
3417	92	ACAGGTCGCGCA	2427, 2454	12
3418	92	GCCGAGGCCGAG	4299, 4335	12
3419	92	GCTGGCCACCGA	1405, 4187	12
3420	92	GGTGGCCGCGGC	2881, 4289	12
3421	92	GTCGCGGCGCTG	3079, 4590	12
3422	92	CCGATGATGTC	3260, 3519	11
3423	92	CCGCCAAGAAG	1698, 1740	11
3424	92	CCGCGACCGGC	1530, 2832	11
3425	92	CGCCGTCGACG	3979, 4694	11
3426	92	CGGGCGGCGGC	2126, 3383	11
3427	92	CGGTGGCCGCG	2880, 3685	11
3428	92	GCCGCCCGCAA	23, 1709	11
3429	92	GCCGCCGCCGT	3353, 3974	11
3430	92	GCGCGCCGATG	2031, 3255	11

3431	92	GCGGGCCAACC	985, 4559	11
3432	92	GGCGATCAACG	13, 4670	11
3433	92	GGCTGCGGGCG	3030, 3750	11
3434	92	ACCGCCGTGG	2221, 2741	10
3435	92	ACCGGGTCGC	1235, 1994	10
3436	92	ACCTGGCGAC	1165, 4504	10
3437	92	ACGAGCTCGA	708, 3156	10
3438	92	ATCGCCATCA	2726, 4029	10
3439	92	CAAGGACGGC	1114, 1555	10
3440	92	CACCACGCCG	2962, 3127	10
3441	92	CACGAACAGC	2332, 3247	10
3442	92	CCAGCTCGGT	1965, 2367	10
3443	92	CCGCGCTGCA	2540, 3709	10
3444	92	CGACGGCGAG	964, 1585	10
3445	92	CGAGGCGATC	121, 4085	10
3446	92	CGCCGCCGCC	3581, 4271	10
3447	92	CGCCGCGAAC	2923, 4538	10
3448	92	CGCTCGAGGA	519, 1275	10
3449	92	CGGCGAGGAG	52, 1336	10
3450	92	CGGCGGCGAG	382, 772	10
3451	92	CGGGCCGTAC	931, 1570	10
3452	92	CGGGTGCTGG	1070, 4098	10
3453	92	GACCACCTCG	1870, 2278	10
3454	92	GCCACGCCAC	468, 1052	10
3455	92	GCCCCGCCCG	2141, 4611	10
3456	92	GCGCGGCACC	250, 1384	10
3457	92	GCGGCCGCGC	1855, 3705	10
3458	92	GCTCGCTGGC	1401, 2499	10
3459	92	GGTAGCCGAT	2865, 3478	10
3460	92	GTCGGCGATC	10, 2584	10
3461	92	TCGGCAGGTC	3186, 3420	10
3462	93	CCGCGCGCCGGGCG	2244, 3737	14
3463	93	CCAGCAGCGGCGC	624, 4381	13
3464	93	CGCCAGCGCCGCG	1258, 1852	13
3465	93	GGTGGCCGGCGAG	2374, 4580	13
3466	93	ACGTGGCCGTCG	723, 1748	12
3467	93	CAGCGCCACCAG	1660, 3965	12
3468	93	CGTCATCGACAG	298, 1183	12
3469	93	GCCAGCGACGCC	659, 1640	12
3470	93	TTCGGCGACGCG	791, 3312	12
3471	93	AACGCCAGCGC	185, 1850	11
3472	93	ACCCGACGCGA	169, 4061	11
3473	93	CAACGCCAGCG	184, 1636	11
3474	93	CACCAGCAGGA	1462, 4595	11
3475	93	CAGCGCGCGCA	1624, 4307	11
3476	93	CCAGCACCACG	864, 4663	11
3477	93	CCGCGGCCAGC	654, 4252	11
3478	93	CGACCGCGCCG	3457, 4267	11
3479	93	CGCGCCGCCCA	1033, 2725	11
3480	93	CGGCGACCGCG	1353, 3454	11
3481	93	CGGTCACCGCG	774, 1431	11
3482	93	GACGACGTGGT	2579, 3789	11
3483	93	GATGCCGGCCA	3076, 4745	11
3484	93	GCAGCGCGGCG	1347, 4108	11
3485	93	GCCGGCGGCGG	973, 2618	11

3486	93	GGTCAACGTGA	2209, 4397	11
3487	93	GTGCCGCCCGG	30, 4545	11
3488	93	GTTTTCGTCGA	2570, 3330	11
3489	93	AACGGGATCA	1373, 1583	10
3490	93	ACCAGCAGCG	3158, 4380	10
3491	93	AGGTGGCCGG	482, 2373	10
3492	93	ATGCCGCCGG	1734, 2361	10
3493	93	CAACCGCGCG	1276, 3471	10
3494	93	CACGGCGTAG	964, 3067	10
3495	93	CAGCCGCGCG	2914, 4562	10
3496	93	CAGCGGCCCG	370, 3995	10
3497	93	CAGGCGCGCG	238, 388	10
3498	93	CCAGCCCAGC	4085, 4658	10
3499	93	CCGCACGGTG	208, 2830	10
3500	93	CCGCCAGCGC	1026, 1302	10
3501	93	CCGCCAGCTG	706, 1869	10
3502	93	CGATCGCGCC	1162, 4615	10
3503	93	CGCGAGCAGG	1723, 1783	10
3504	93	CGCGTCCGGC	2023, 3949	10
3505	93	CGGCGTCGAA	2650, 3623	10
3506	93	GACGAGCACC	1420, 2123	10
3507	93	GAGGCCGGCG	1094, 2480	10
3508	93	GCACCAGCAG	1461, 4378	10
3509	93	GCCAGCCCCG	807, 1133	10
3510	93	GCCAGGGGCC	1119, 4234	10
3511	93	GCCGTCGGCG	3618, 3762	10
3512	93	GCTGCTGGCG	742, 4193	10
3513	93	GGTGAGCGCC	1252, 3728	10
3514	93	GTGCAGCGCG	1621, 4106	10
3515	93	TCAGCAGCAC	3183, 4459	10
3516	93	TCGGCCCCGG	435, 3646	10
3517	93	TGGTGCCCGC	3, 1716	10
3518	94	CGGGCTAGTGGCGATCGCAAGCGCGGCGAAGC	1616, 1671	32
3519	94	GGGCGCGGCGGGTCGCCACCATCG	1649, 1704	24
3520	94	CCTGCTGCTGGTG	645, 4126	13
3521	94	GGGTCACCGGCGC	1316, 4023	13
3522	94	CATCCGCCGCCG	862, 4531	12
3523	94	CCGAGAAGCCGC	287, 3993	12
3524	94	CGTCATCGCCGT	765, 4147	12
3525	94	GATCGTCATCGC	762, 1350	12
3526	94	CCAGGGCCCCG	2771, 3530	11
3527	94	CGCGCTGGCCG	1479, 3584	11
3528	94	CGCTGACCAAC	248, 3568	11
3529	94	CGGCGCCGTCC	3139, 4042	11
3530	94	CGGCGCGCTGG	876, 1476	11
3531	94	CGGCTCGCCGA	897, 4328	11
3532	94	CGGGCGCGGCG	1703, 3343	11
3533	94	CTCGTGCTGGG	604, 4448	11
3534	94	GAGCCCCAGCG	4418, 4739	11
3535	94	GCGCGGCCGAC	1788, 3292	11
3536	94	GGCCGCCGCCG	2696, 3422	11
3537	94	GGCGGCGCCGC	240, 2816	11
3538	94	GGCGTTCGTCG	2390, 4642	11
3539	94	GGGTGCTCACC	3595, 3619	11
3540	94	GTGGTGGTGGC	1072, 2406	11
3541	94	ACGCGCTGGC	3583, 3679	10

3542	94	CCACGGTCAA	2488, 3040	10
3543	94	CCGATCGCCG	3474, 4505	10
3544	94	CCGGCGCGGC	503, 3289	10
3545	94	CGATCCCGCC	2467, 3113	10
3546	94	CGCCCACGGT	618, 2485	10
3547	94	CGCGGGCAGC	2447, 2753	10
3548	94	CGGGTGCTCA	3618, 3707	10
3549	94	GACGCCGGCG	3065, 3718	10
3550	94	GCCGACCACG	3035, 4478	10
3551	94	GCCGCCAGGT	316, 3825	10
3552	94	GCCGTCCTCG	661, 2060	10
3553	94	GCGCGCACTG	1460, 4591	10
3554	94	GCTGCGGGCC	3449, 3548	10
3555	94	GGACGCCGGC	3064, 3077	10
3556	94	GGCGCTGGGC	1572, 4273	10
3557	94	GGGGCGCGGC	1648, 3150	10
3558	94	GGTCGCGGGC	308, 2750	10
3559	94	GTGGGCGCCG	2550, 2790	10
3560	94	TCATCGGGGC	425, 1562	10
3561	94	TCGACGGGGT	1310, 3406	10
3562	94	TCGCGGGTGG	211, 3900	10
3563	94	TGGTCGCGGG	2749, 3897	10
3564	95	GGGGGTCCGGCGTGCG	393, 460	16
3565	95	CGGTGCTGCTCGG	333, 372	13
3566	95	GCCGCCCGGGCGC	197, 674	13
3567	95	GCCGCCGCGACGG	906, 2339	13
3568	95	CAACGCCGCCGC	3206, 4305	12
3569	95	CGCCGGGATGCG	415, 2683	12
3570	95	CGGTGATCGCGG	4480, 4663	12
3571	95	GCCCGCGCGATC	1228, 3399	12
3572	95	GCTGCTGCGCAA	1401, 1452	12
3573	95	AGCACCGCGAC	2661, 3489	11
3574	95	CCAGTCGCAGC	2830, 3388	11
3575	95	CCTGCTGACGG	1080, 2163	11
3576	95	CGATCGCGGCC	3259, 4735	11
3577	95	CGCGGCGTCGG	1757, 1993	11
3578	95	GCCCGGTTCCC	2265, 2487	11
3579	95	GCGGTGGCGGC	116, 4608	11
3580	95	GGCCCGGATTC	92, 3301	11
3581	95	GTTGGCGACGA	4251, 4700	11
3582	95	ACCGCGACGC	1145, 1986	10
3583	95	ACCGCGGTGG	113, 143	10
3584	95	CACGGCCGGA	4043, 4574	10
3585	95	CCGAAGCCGC	192, 276	10
3586	95	CCGCCATCCG	1659, 2442	10
3587	95	CGGCGATCGC	1619, 4732	10
3588	95	CGGGTGTTGC	2626, 2698	10
3589	95	GCTCGGCCGA	177, 3334	10
3590	95	GCTGGGCCGG	385, 3557	10
3591	95	GGCCCGCGCG	286, 1227	10
3592	95	GGCGTCGCCG	605, 1951	10
3593	95	GGGCCTGACC	502, 1383	10
3594	95	GGGTCGTCCG	872, 3731	10
3595	95	GGTCGCCGCC	1654, 4415	10
3596	95	GGTGCTGATG	2110, 3473	10

3597	95	TCGAGGCGTT	30, 2394	10
3598	95	TGTTGCGGCG	229, 2757	10
3599	95	TTCGGGGCCG	4388, 4400	10
3600	96	GCCCGGCGCCGTCG	1573, 4711	14
3601	96	CAGCGCCGCCACC	2120, 2297	13
3602	96	CTCGCCGGCCCGG	1661, 3507	13
3603	96	GCGGACAACAGCA	3059, 3131	13
3604	96	CGCGGCGCTGAT	1744, 1871	12
3605	96	GGCCTGCTCGCG	3885, 3969	12
3606	96	GGTGCCCGCCGC	597, 714	12
3607	96	TCGGCGGCCACC	1830, 1902	12
3608	96	TGCCACCGCCGC	657, 3261	12
3609	96	CACCTCGCCGG	2515, 4679	11
3610	96	CAGCGCGCCGG	4056, 4296	11
3611	96	CCGGCGGCGCC	85, 3829	11
3612	96	CGGCGAGGTCC	1178, 1205	11
3613	96	GATCGGCGACC	1936, 4361	11
3614	96	GCACGTCGTCG	2715, 3399	11
3615	96	GCAGCGAGGTG	3147, 4043	11
3616	96	GCCGCCAACCG	1515, 2310	11
3617	96	GCGTCGGCGGC	49, 1827	11
3618	96	GGCCGCCGCGG	126, 4437	11
3619	96	AGCCGGCGGC	1023, 1707	10
3620	96	CCAATCCGTT	2096, 4645	10
3621	96	CCCGCCGGCG	1173, 1443	10
3622	96	CCCGGCCGCG	2696, 4325	10
3623	96	CCGCGGATCT	1328, 1845	10
3624	96	CCTCGGCGGC	1900, 2215	10
3625	96	CGATCGGCGG	551, 581	10
3626	96	CGCGGCGTCG	736, 1823	10
3627	96	CGGCATCGAT	575, 1537	10
3628	96	CGGCGGGATC	264, 2377	10
3629	96	CGTAGCCGCC	4010, 4478	10
3630	96	GCCACCAGCA	1494, 2325	10
3631	96	GCCCAGCCGG	1703, 4410	10
3632	96	GCGGCATCGA	574, 1953	10
3633	96	GGGTGCCCGG	2691, 3162	10
3634	96	GTAGGGGCTG	841, 923	10
3635	96	GTCGACGTGC	3250, 3474	10
3636	96	TCCCGATCGG	2256, 2913	10
3637	96	TCCCGGTCGG	1633, 2046	10
3638	96	TCGCCGGCCA	238, 1602	10
3639	96	TCGCGCAGGG	443, 4153	10
3640	96	TTTCGACGCC	372, 2874	10
3641	97	AGCTGGCGATCGCCGCGGAGCAGATGGTGGCGACCCGCT GCGCCCCGGCGCTGCCGG	1373, 1430	57
3642	97	CACCATGTTCGACGA	3405, 4219	15
3643	97	AAGATCGCGCG	3091, 4367	11
3644	97	CAGCAGGGCCA	3814, 3871	11
3645	97	CCGGCGAGCTG	3323, 3464	11
3646	97	CGCCGACGTCG	560, 2058	11
3647	97	CGCGGTGGCGG	2214, 2940	11
3648	97	CGGCGGGCACG	2400, 2931	11
3649	97	CGGTCCGCGAG	152, 4086	11
3650	97	GAGGATTCGAT	1047, 4013	11
3651	97	GGGTGCGCCGC	759, 3624	11

3652	97	GTGGTCGAGGC	1293, 4481	11
3653	97	TGGTCGGGCTG	973, 1946	11
3654	97	AAAGCCGTGG	735, 4708	10
3655	97	AAGGCGGCCA	2849, 3526	10
3656	97	AGGTCGCCCG	586, 3218	10
3657	97	AGTGAGCCTG	1315, 4620	10
3658	97	ATCCGCCGGC	3734, 3986	10
3659	97	CCAGCAGGGC	3834, 3870	10
3660	97	CCGCGCGGTG	2211, 4463	10
3661	97	CCGGTGCGGT	987, 1087	10
3662	97	CCTGTCGAAG	1154, 4360	10
3663	97	CGACCGGCAG	2544, 3954	10
3664	97	CGAGGCCGCG	3258, 4078	10
3665	97	CGGCGCCGAC	557, 2676, 4743	10
3666	97	CGGCGTTCAA	335, 3518	10
3667	97	CGGCTCACCA	3400, 4418	10
3668	97	CGGGATCGGC	32, 329	10
3669	97	GACATCGAGC	2884, 3073	10
3670	97	GAGATGTTCG	2272, 4175	10
3671	97	GCAGCAACCG	3702, 3768	10
3672	97	GCAGCCCTAT	3756, 3798	10
3673	97	GCCCGACATC	2502, 2880	10
3674	97	GCCGAGGCGC	1014, 4592	10
3675	97	GCGCGGTGTC	3062, 4513	10
3676	97	GCTGGGCGCC	1249, 2142	10
3677	97	GGACAGCCCG	1353, 3925	10
3678	97	GGCCTGGGCG	2392, 2868	10
3679	97	GGGCTGGCCA	256, 2647	10
3680	97	GTCATCACCA	1807, 2740	10
3681	97	TCGAGGCCGC	1006, 3257	10
3682	97	TGGCCGCCGC	3496, 3688	10
3683	97	TTCGACCAGG	2314, 4061	10
3684	98	CGCCCCCGCCGCC	3423, 3471	13
3685	98	GCTGGCCCGGGCG	1359, 4477	13
3686	98	CCGCAGGCCCCG	1002, 3038	12
3687	98	CTGGCCGAACTG	1606, 2603	12
3688	98	GGCGACGCCGCC	3958, 4547	12
3689	98	GGCGCTGTGGGC	1823, 2392	12
3690	98	ACCCGAACCCG	3025, 4736	11
3691	98	CCCGGGACGAA	3243, 3255	11
3692	98	CCGACGACCTG	838, 2595	11
3693	98	CCGCCGCCACC	829, 2888	11
3694	98	CCGGCGCCGCC	3416, 3552	11
3695	98	CGAGTTGCGCG	719, 2575	11
3696	98	CGCCGACGACC	1326, 2593	11
3697	98	CGCCGGCGCCG	3550, 3979	11
3698	98	CGCCGGCGCGC	3197, 3463	11
3699	98	CGCTGGCCCGG	871, 4476	11
3700	98	CGTCGCCGCCG	3644, 3973	11
3701	98	GACCTCGCGCA	2215, 2666	11
3702	98	GACGGCCGCGG	1703, 3109	11
3703	98	GATCGCCGACG	1323, 1853	11
3704	98	GGCGGCCGACG	884, 4228	11
3705	98	GGTGGTGGTGC	986, 1040	11
3706	98	GTTGCTGGCCG	514, 1602	11

3707	98	TCGCCGCGCTG	588, 2077	11
3708	98	TGGCCGGGGTT	1067, 4584	11
3709	98	TGGCGCCGCTG	207, 4116	11
3710	98	ACCTGACGCT	1487, 1652	10
3711	98	AGATCGCCGA	940, 1852	10
3712	98	CCGAGACGAA	3232, 3268	10
3713	98	CCGCCGCCGG	2978, 3494	10
3714	98	CCGCGCTCGG	2682, 3849	10
3715	98	CCGCTGGCCC	1504, 4475	10
3716	98	CGACGAGACG	673, 2833	10
3717	98	CGAGGACGCC	3544, 4303	10
3718	98	CGCCGACGTC	127, 1028	10
3719	98	CGCCGAGGAC	2021, 4300	10
3720	98	CGCGGGCAAC	741, 4342	10
3721	98	CGTTGCTGGC	513, 1798	10
3722	98	CTGGCTGGCC	1356, 1539	10
3723	98	GACGTCACCG	798, 4750	10
3724	98	GCCGACGCCC	3167, 4370	10
3725	98	GCCGCAGGCC	1001, 2280	10
3726	98	GCGCGCTGGC	868, 2059	10
3727	98	GGCCGCCGCC	827, 2015	10
3728	98	GGCGTCGGCC	821, 1931	10
3729	98	GGGCTGGCTG	689, 1353	10
3730	98	GTCGCCGAGG	308, 4298	10
3731	98	GTCGTCGACG	792, 1222	10
3732	98	GTGCGCGCCA	629, 1842	10
3733	98	GTGCTCGCCG	1246, 4364	10
3734	98	GTGGTCGCCG	305, 2073	10
3735	98	TGACCTCGCG	1469, 2214	10
3736	99	CGGCGGCGCGGCCGCGC	613, 2773	17
3737	99	CGGCGGCCGCCGGCG	3471, 3950	15
3738	99	CCACAACTACAT	4524, 4749	12
3739	99	CCACGCTGCAGG	2385, 2409	12
3740	99	TCGGCCGCGCTG	575, 1785	12
3741	99	TGACCATCGACG	941, 1420	12
3742	99	ACGTCGACCTG	2475, 2640	11
3743	99	CGACGGCCTGG	505, 1892	11
3744	99	CGGCGCCCGCC	711, 1017	11
3745	99	CGTCGTCGGCG	2326, 2869	11
3746	99	GCGCACCACGG	277, 605	11
3747	99	GGTGACCATCG	939, 993	11
3748	99	GGTGCTCGACG	646, 3499	11
3749	99	TCAGGGCCGGA	815, 4272	11
3750	99	TGGACGCGGCG	630, 962	11
3751	99	ATCGTCTTCG	233, 1177	10
3752	99	CATCGAAATG	781, 3109	10
3753	99	CCGGAACGGG	3552, 4040	10
3754	99	CCGGCGCCCG	710, 4127	10
3755	99	CCGGTCGCCG	2243, 3937	10
3756	99	CGCGGCGGCC	1772, 3469	10
3757	99	CGTCATCGAC	1670, 4085	10
3758	99	CTGCCGGTCG	2300, 2588	10
3759	99	GAAGGCGTCG	221, 4576	10
3760	99	GACGACGTCG	1941, 2321	10
3761	99	GCCCGGACCG	292, 387	10
3762	99	GCCGGCGCCC	590, 709	10

3763	99	GGCCGACGTC	2470, 4079	10
3764	99	GGTCGCCGAC	448, 3028	10
3765	99	GTCGTGCAGC	1362, 3434	10
3766	99	TGGCCACCGG	1957, 3402	10
3767	100	CGCCACCGGGTTG	3072, 3667	13
3768	100	GACGAGCTGCACA	551, 1226	13
3769	100	TGGCCGCGCAGGT	48, 2695	13
3770	100	CGCGGTGGGCGA	3840, 4198	12
3771	100	CGCTGGTCGCGG	453, 825	12
3772	100	CGGCAGATGTCC	2551, 4616	12
3773	100	CTTGGCGACCTG	2292, 3102	12
3774	100	GCGATCGACGCC	593, 1004	12
3775	100	GGCCGTCGTCGA	361, 3089	12
3776	100	ACGACCGGTTC	864, 1449	11
3777	100	AGCTCACCGAG	1185, 1518	11
3778	100	CCGGCGTCGGC	345, 3872	11
3779	100	CCTGGCCGCGC	4267, 4726	11
3780	100	CGCACCACCGG	795, 4337	11
3781	100	GGCGCCCGGGT	3178, 4214	11
3782	100	GGCGGGCACCG	148, 3245	11
3783	100	GTTCGACGAGA	961, 1561	11
3784	100	TCGAGAAGGCG	1023, 1572	11
3785	100	TGATCTTCACC	1677, 3900	11
3786	100	AGATCGCCGA	972, 1140	10
3787	100	AGCTGACCCG	81, 1854	10
3788	100	AGGACATGGC	1926, 4311	10
3789	100	ATCCAGCGCG	1895, 2021	10
3790	100	CAAGGCGATC	883, 1279	10
3791	100	CCCGCCACCG	2476, 3070	10
3792	100	CCCGCGGCGA	525, 630	10
3793	100	CCGATCAGCA	2827, 4160	10
3794	100	CCGCAAGCCG	1537, 2161	10
3795	100	CGACCGCTAC	781, 850	10
3796	100	CGACGAGCTG	550, 1786	10
3797	100	CGACGTCGAC	2098, 4141	10
3798	100	CGAGGACGCG	2131, 2592	10
3799	100	CGAGGCGGGC	145, 904	10
3800	100	CGATGGCCGG	3683, 3702	10
3801	100	CGCGCGCCGG	982, 3124	10
3802	100	CGGCGCACCA	308, 2012	10
3803	100	CGTTGGTGCT	195, 4026	10
3804	100	CTGCTGGGCC	17, 3438	10
3805	100	GAGCCGACGG	737, 2929	10
3806	100	GATCCTCAAG	610, 763	10
3807	100	GCCAGCCTGC	1034, 3604	10
3808	100	GCCGCCGAGG	140, 581	10
3809	100	GGAGCTGACC	79, 1939	10
3810	100	GGCCGACCGC	847, 3588	10
3811	100	GTCACGGTCG	2090, 2317	10
3812	100	TCCAGCCGGT	717, 2309	10
3813	101	CGTCGTCGGGTAGC	354, 2721	14
3814	101	GCCGGCTGACCTC	2460, 3053	13
3815	101	GGCGCGCGCGGTG	289, 505	13
3816	101	GTCGTCGTGATCG	571, 1743	13
3817	101	CTCGGACAGCCG	1060, 3760	12

3818	101	GGCCCGCGACAG	3039, 3111	12
3819	101	GGCGCTGGCCGC	667, 2586	12
3820	101	ATCGCGGGGCG	282, 1546	11
3821	101	CAGCGCGACCG	325, 3871	11
3822	101	CCGCCGGGCAG	1023, 1128	11
3823	101	CGAATAGCGGG	1519, 1531	11
3824	101	CGAGCAGCTCG	2762, 2888	11
3825	101	CGGCCAGCGCG	2669, 3855	11
3826	101	GACGACGTCGT	565, 2940	11
3827	101	GCACGATCAGC	2855, 3446	11
3828	101	GCCCCAGGCGC	643, 1862	11
3829	101	GTCGGTCAGCC	94, 3904	11
3830	101	TCGGCCAGCGC	696, 3854	11
3831	101	AGGGTGACGA	1984, 3016	10
3832	101	CACATCCAGC	109, 154	10
3833	101	CACCGAGATC	1697, 3195	10
3834	101	CACCGCGGTC	1039, 1147	10
3835	101	CACGTCGGCG	376, 1502	10
3836	101	CACGTGGCGC	2535, 3144	10
3837	101	CCACCGCGGT	1038, 2239	10
3838	101	CCAGGCCGTC	45, 3485	10
3839	101	CCGCGGCGCT	663, 3614	10
3840	101	CGATTCGTCG	962, 3258	10
3841	101	CGCCGATGCC	2446, 2555	10
3842	101	CGCGTCCAGC	2784, 3583	10
3843	101	CGGCCAGGCC	417, 3804	10
3844	101	CGGTGGACAG	3326, 3927	10
3845	101	GACGGCGCGC	401, 502	10
3846	101	GCAGATCGGC	2633, 3849	10
3847	101	GCCGCCACCG	1764, 1964	10
3848	101	GCCGCCGAGC	1076, 2883	10
3849	101	GCCGCGGTGC	1953, 2121	10
3850	101	GCGACCACGT	1497, 3139	10
3851	101	GCGCCGTCCA	2187, 3343	10
3852	101	GCGGGTCGCG	391, 1303	10
3853	101	GGCGATGTCG	2871, 3625	10
3854	101	GGCGCCGCCG	1907, 2441, 3607	10
3855	101	GGCGCGCGGC	1482, 3750	10
3856	101	GTCGGTGCCG	184, 2925	10
3857	101	TCGCCGACGG	684, 1431	10
3858	101	TCGCCGGCCA	5, 3800	10
3859	102	ACGTGCTGGTGCACG	2680, 4843	15
3860	102	CGGCGGCGCTGGCCG	4657, 4822	15
3861	102	CGCCGCCGGCGACG	433, 4331	14
3862	102	ACGCCGCCGGCG	432, 4015	12
3863	102	ACGGCGCGGCGC	24, 849	12
3864	102	CGCCGATGAGCG	76, 3054	12
3865	102	CGGCGACGTGGG	3187, 3241	12
3866	102	CGGCGATGGCCC	328, 4231	12
3867	102	CGTCGGCGGCGC	217, 4819	12
3868	102	GTTCGCCGACGC	3151, 4622	12
3869	102	AAGGCCGCCAA	1699, 4155	11
3870	102	CCGCGCCGAGG	3403, 4685	11
3871	102	CGCCGGCGGGC	1256, 2612	11
3872	102	CGGGAACCGGC	124, 3376	11
3873	102	CGTCGACGTGC	3097, 4838	11

3874	102	CTGCTGCAGCG	2892, 4437	11
3875	102	GCAGGGCCTGC	61, 3707	11
3876	102	GCCTTGGCCGC	1180, 2649	11
3877	102	GCGCCGACGTC	403, 1294	11
3878	102	GCGGGCCACCG	2597, 3947	11
3879	102	GCTGCGCCACG	3307, 4733	11
3880	102	GGACGCGGTGA	3491, 4529	11
3881	102	GGCCGCCGCGG	389, 3842	11
3882	102	GGTGACGTGCC	347, 941	11
3883	102	TGGCCGCCGCG	388, 2668	11
3884	102	ACGTCGGCGG	2962, 4818	10
3885	102	CCGACGGCGA	46, 3183	10
3886	102	CCGCATCACG	1901, 4299	10
3887	102	CCTCGACGAG	2326, 4787	10
3888	102	CGACGCCCTG	2741, 3911	10
3889	102	CGATCCGCGC	3271, 4750	10
3890	102	CGGCGCCACC	1212, 3695	10
3891	102	CGGGGTGGTC	1329, 2819	10
3892	102	CGGGTCGGCG	164, 4584	10
3893	102	CGTCCTGGCC	4177, 4418	10
3894	102	CTACCTGGGC	4247, 4472	10
3895	102	GATCGAAACC	1930, 3995	10
3896	102	GCCGGCCACG	1539, 2754	10
3897	102	GCGGCCACCT	3876, 4113	10
3898	102	GCGGTGCCGG	117, 4650	10
3899	102	GCGTGGTCGA	2527, 2734	10
3900	102	GCTGATCGCC	1281, 3770	10
3901	102	GGCGATCCGC	2991, 4748	10
3902	102	TCCTCGCCGG	618, 2587	10
3903	102	TCCTGCACCG	1477, 4564	10
3904	102	TTCGACATCC	3750, 4263	10
3905	103	AGCACCGCCACCG	1999, 2167	13
3906	103	CCGCGCCGACGCC	1491, 1977	13
3907	103	CGAACCCGGCCAG	2513, 4675	13
3908	103	TCGACGTCGTGCT	1229, 2068	13
3909	103	ACCGCGGCGGCC	1145, 4506	12
3910	103	ATGGCCGGCAAT	118, 967	12
3911	103	CCGCGCTGCTGG	596, 1070	12
3912	103	CCGGCAGGCCGT	3752, 4468	12
3913	103	CGCGCCGCCACA	2090, 3233	12
3914	103	GGGCCGCGGCCC	216, 746	12
3915	103	CCAGCACCGCC	2165, 3005	11
3916	103	CGACGTTGGCC	2234, 3773	11
3917	103	CGCCGCGGCGC	136, 2454	11
3918	103	CGGCCAGCGCC	3116, 3503	11
3919	103	CGGCCGCGATC	2249, 3947	11
3920	103	CGGCGACCGCG	374, 4501	11
3921	103	CGGCGCCACCC	2484, 3933	11
3922	103	CTGCGCGCCGG	358, 3804	11
3923	103	GAACGCCTCGG	990, 4394	11
3924	103	GCGCGATCGCC	2945, 4594	11
3925	103	GCTGCGCGCCG	2439, 3803	11
3926	103	GGATCGCGGCC	810, 3110	11
3927	103	GGTCAGGTTGG	2127, 2730	11
3928	103	GGTGCACACCC	2028, 4739	11

3929	103	AACCGCACGC	4582, 4780	10
3930	103	AAGGCGGGCA	154, 2275	10
3931	103	ACCGCGCCGA	1490, 2650	10
3932	103	AGGGCCGCGG	215, 1585	10
3933	103	AGGTCACCGA	48, 4043	10
3934	103	CACCGCCGAC	58, 2304	10
3935	103	CACGGCGGCG	2823, 2856	10
3936	103	CAGCGCGACG	2529, 3183	10
3937	103	CAGGTCGGCG	3039, 4862	10
3938	103	CCCGCCCAGC	2328, 4891	10
3939	103	CCCGCGCCGC	437, 4721	10
3940	103	CCGCACCGCC	4196, 4490	10
3941	103	CGACCCGACG	1272, 2928	10
3942	103	CGACGAGCCG	4002, 4483	10
3943	103	CGACGCGGGC	2016, 3917	10
3944	103	CGAGGTCACC	46, 4283	10
3945	103	CGCCGACGAG	7, 3999	10
3946	103	CGCCGGCGGC	840, 2957	10
3947	103	CGGCGATCGC	2261, 4309	10
3948	103	CGGTCGGCGA	3817, 4386	10
3949	103	CGTCGACCGC	1785, 3725	10
3950	103	GCCCACCGCG	1141, 3402	10
3951	103	GCCGGCGACC	372, 1321	10
3952	103	GGGCATCGCG	453, 519	10
3953	103	GGGGTCAGCA	1210, 2842	10
3954	103	GTCCATCCCG	957, 2322	10
3955	103	TACCGCGCCG	1489, 4334	10
3956	103	TCGCGCCGCC	286, 3232	10
3957	103	TCGGGCGGCC	187, 4424	10
3958	104	GTTGCGGACCCGCA	515, 3659	14
3959	104	ACAGCACCCGGTC	1778, 2360	13
3960	104	CAGCGCCGACACC	1869, 2633	13
3961	104	CCCCGACGATCTG	1002, 1203	13
3962	104	GCCGAACGCCAGG	1288, 2373	13
3963	104	CGGCCACCTCGG	894, 2545	12
3964	104	CGGTCAGCAGCG	1218, 4829	12
3965	104	GCGCGGCCGCCA	1524, 4240	12
3966	104	TCGCGGTCCAGC	406, 2158	12
3967	104	ACCGGCACGCC	851, 1073	11
3968	104	CCACACGTCGG	3227, 4671	11
3969	104	CGGCCGCCGGC	963, 4171	11
3970	104	CGGCGATGGCG	2839, 3109	11
3971	104	GCAGCGCGGCC	1521, 2473	11
3972	104	GCTCGTTGCGG	2977, 3655	11
3973	104	GGCGGCGGCGA	1087, 2813	11
3974	104	GGGCGCCGCGC	2387, 4446	11
3975	104	GGTCGATCAGC	1862, 3941	11
3976	104	TCGGCGGCGGA	54, 342	11
3977	104	TGCTCGCGACG	831, 3604	11
3978	104	ACCACCCCGA	1031, 1301	10
3979	104	CACGATCCCG	877, 1054	10
3980	104	CACGATCTCG	218, 3545	10
3981	104	CAGCGCCCGC	125, 2104	10
3982	104	CCAGCAGCGC	993, 1518	10
3983	104	CCAGCGCCAG	454, 1170	10
3984	104	CCATCAGCCG	1626, 4399	10

3985	104	CCGGCCACCA	1505, 1654	10
3986	104	CGCCGGCGCC	785, 3962	10
3987	104	CGCGCCGGCG	783, 3490	10
3988	104	CGCGCGACGC	2570, 3213	10
3989	104	CGCGGCGAAC	1045, 2717	10
3990	104	CGGCGACGGC	586, 3202	10
3991	104	CGTTGCCAGC	1138, 2505	10
3992	104	CTGGGCGATG	44, 1956	10
3993	104	GCCGATCAGC	1375, 4581	10
3994	104	GCCGCCGCCG	929, 3077	10
3995	104	GCGAGCCGGT	1613, 2916	10
3996	104	GCGATGACGA	1151, 3886	10
3997	104	GCGGGCGTGG	630, 3852	10
3998	104	GCGTCGGCGA	3105, 4795	10
3999	104	GGCCTGGCGC	308, 3027	10
4000	104	GGCGATGACG	1150, 4163	10
4001	104	GGCGATGTTG	104, 2171	10
4002	104	GGCGGCCACC	1024, 2936	10
4003	104	GGTGCCGATC	1746, 2414	10
4004	104	GTCACCGAAC	3467, 4497	10
4005	104	GTCGTAGAAG	293, 2963	10
4006	104	TCGTTCTCGA	2703, 2997	10
4007	104	TGCGCTCGCC	1646, 4111	10
4008	105	CGCGACCACCGCG	906, 4823	13
4009	105	GCGGCGGCGACCA	3295, 3532	13
4010	105	GGCCGCGGCGGCG	2781, 3528	13
4011	105	AGGCGCTGGCCG	956, 2594	12
4012	105	CCGAGATCGCCG	1721, 1742	12
4013	105	CGCGCTCGGCGA	3233, 3714	12
4014	105	GCCGACGGCGAC	1444, 2653	12
4015	105	GCTGCTGATCGA	1107, 1854	12
4016	105	TGCTCGCCGCCG	3485, 4777	12
4017	105	CACCGACCCCG	936, 2154	11
4018	105	CCGACACCAGC	64, 4840	11
4019	105	CCGCCGACGGC	1091, 1442	11
4020	105	CCGCGCGCGCC	665, 2954	11
4021	105	CGACCTCGACG	1022, 3585	11
4022	105	CGAGCCGCTGC	1143, 3561	11
4023	105	CGCCGAGCAGG	1398, 2448	11
4024	105	CGCCGCCGACG	1062, 1440	11
4025	105	CGTCACCGACC	933, 3579	11
4026	105	CGTCCTGGCCG	1257, 3161	11
4027	105	CGTGGCGGCCG	761, 2736	11
4028	105	GCGATCCGCGA	128, 727	11
4029	105	GCGCGTCGAGG	1233, 4567	11
4030	105	GGCCCGCTCGG	3516, 4192	11
4031	105	GTCGGTCTTGG	23, 4418	11
4032	105	ACCGCCACGT	114, 255	10
4033	105	ACCGCCGTCG	1303, 2908	10
4034	105	ACCGGCGACT	2248, 4300	10
4035	105	ACCTGTGCGC	854, 1373	10
4036	105	AGATCGGCCG	2776, 3886	10
4037	105	CCGAGTTCGG	234, 2890	10
4038	105	CCGGCCGCTG	1170, 2229	10
4039	105	CCGGCGCGCT	2492, 3229	10

4040	105	CCGGCGCGGG	2024, 3972	10
4041	105	CCGGCTGGCC	780, 4053	10
4042	105	CGAAGGCGAC	996, 4107	10
4043	105	CGACGGCGCG	1014, 3999	10
4044	105	CGCGCCGGCG	3968, 4670	10
4045	105	CGCGGCCGCG	1473, 3063	10
4046	105	CGGAGGAATC	818, 3453	10
4047	105	CGGCAAGCCG	1338, 3741	10
4048	105	CGGCGTCGCG	804, 3105	10
4049	105	CGGCGTGCGC	1668, 4561	10
4050	105	CGTCGCCGAG	1737, 2445	10
4051	105	CTCGAGACCG	1297, 4294	10
4052	105	CTGCTGACCG	2086, 2728	10
4053	105	GCGATGGCCA	177, 2371	10
4054	105	GCGGTCGGTC	20, 3909	10
4055	105	GCTTCGCCGG	977, 3941	10
4056	105	GGAGGCGGCC	873, 3603	10
4057	105	GGCCCAAGCC	1814, 4273	10
4058	105	GGCGGCTGGC	2077, 2333	10
4059	105	GGTGATCGGC	1905, 4137	10
4060	105	GGTGGGCGGC	1916, 2529	10
4061	105	TCCGCCCCGG	1198, 4171	10
4062	105	TGCCGCGCGC	663, 2927	10
4063	105	TGGCCGTCGG	4554, 4692	10
4064	105	TTCTCCCGGC	775, 1165	10
4065	106	GACGGGTCGCTGG	3241, 4607	13
4066	106	GCACGTCGAGCA	208, 2028	12
4067	106	CACCGAGGGCG	2955, 3479	11
4068	106	CACTTCTACCG	26, 3846	11
4069	106	CCGACGGGGCC	739, 3922	11
4070	106	CCGCGCCCGTC	1937, 2223	11
4071	106	CCGGCGGCTGG	283, 1479	11
4072	106	CGCCGACGACG	2457, 4040	11
4073	106	CGCGGCACCGG	1825, 1871	11
4074	106	CGCGGCGAACG	323, 2367	11
4075	106	CGGCCAGGGCG	832, 2777	11
4076	106	CTCGCCGTCGA	254, 2707	11
4077	106	GCACCGCGGTC	2855, 4225	11
4078	106	GCGCGCCACCG	878, 2244	11
4079	106	GGCCCAGGAGG	1244, 1569	11
4080	106	AACGGCGCGT	180, 1669	10
4081	106	ACCGCCGGGT	3667, 3748	10
4082	106	ACTGGCACCT	1541, 4572	10
4083	106	AGATCCGGGA	1328, 3601	10
4084	106	CACCGCCGGG	1911, 3747	10
4085	106	CACCGGCATC	4333, 4750	10
4086	106	CATCGAGGCC	3404, 4468	10
4087	106	CCATGTCGGC	4090, 4278	10
4088	106	CGACGGCGGC	1650, 4651	10
4089	106	CGAGCGGTTC	2691, 3072	10
4090	106	CGATTCCAGC	338, 512	10
4091	106	CGGCGAGCTG	854, 2648	10
4092	106	CGTGCTGCTG	2619, 3198	10
4093	106	CTTGGCCAGC	197, 476	10
4094	106	GCCCGGCGAG	929, 3536	10
4095	106	GGCGTTCCTG	1713, 3015	10

4096	106	GGGCACCGCG	983, 2853	10
4097	106	GTGCTCGCCG	2683, 2704	10
4098	106	TCGCCCTGGA	2870, 3736	10
4099	106	TGGCGCGACG	2338, 4787	10
4100	106	TTCGCTGGGC	2841, 3386	10
4101	107	GCGGCGATCGCGGC	1498, 1831	14
4102	107	GTTCGACGTCGAGG	1796, 4182	14
4103	107	TCGGCGGCGCCGA	2430, 3417	13
4104	107	CCGCCGAGCCGA	107, 3937	12
4105	107	CGCGCCGATCGC	2492, 2666	12
4106	107	GACCGCGCACAC	144, 2606	12
4107	107	GCGGCCAGCAGG	2451, 3204	12
4108	107	GCGTTCGGCGGC	1989, 2426	12
4109	107	AGCAGCCAGCC	1327, 4694	11
4110	107	CACGGCGCTGG	387, 3920	11
4111	107	CACGTAGAACG	1101, 2019	11
4112	107	CAGCGCCTCGG	2228, 3410	11
4113	107	CGATGGCGATG	1022, 3304	11
4114	107	CGCGGCGACGG	532, 2972	11
4115	107	CTCGGCGGCCA	2210, 2447	11
4116	107	GCCGCGGCGGC	1903, 2253	11
4117	107	GGGCCAGGCGC	1124, 2527	11
4118	107	TCGACGTGCTC	794, 4428	11
4119	107	ACCGCGCGGC	1300, 3199	10
4120	107	ACGTCGGCGA	2619, 3318	10
4121	107	ACGTCGGCGT	1090, 3609	10
4122	107	AGCCCGTGGT	1444, 1769	10
4123	107	AGCCGCCCGA	353, 1757	10
4124	107	CACCCGGCCG	14, 3359	10
4125	107	CACGTCGGCG	3317, 3608	10
4126	107	CAGCCGCCAG	1338, 2888	10
4127	107	CAGCTCCTGC	2102, 2789	10
4128	107	CCACGGGCCG	915, 4126	10
4129	107	CCGCGGCCAG	1241, 4149	10
4130	107	CCGCGGCGAC	531, 2311	10
4131	107	CCGCTCGGCG	3689, 4648	10
4132	107	CGAGCAGAAC	270, 1053	10
4133	107	CGATCGGCCG	2368, 4545	10
4134	107	CGCCGCGGCG	1902, 2309	10
4135	107	CGCGGCGCCG	100, 2864	10
4136	107	CGCGGCGTCG	2705, 3950	10
4137	107	CGGTGTCGCC	3895, 4524	10
4138	107	CGTAGGCGCG	884, 3463	10
4139	107	CGTCGCGGCG	2702, 3048	10
4140	107	GAAGCGGGTC	1617, 2573	10
4141	107	GACGTCGGCG	1089, 2618	10
4142	107	GCACCAGCGC	2659, 3761	10
4143	107	GCCCGCCAGC	2693, 4445	10
4144	107	GCGCGCACCG	1915, 2934	10
4145	107	GCTCGGCGGC	2446, 2716	10
4146	107	GGCCGCCACC	724, 1233	10
4147	107	GGTCTGCAGC	3185, 4273	10
4148	107	GGTGCCCAGC	1857, 2753	10
4149	107	GTCGTGTTCC	2165, 3152	10
4150	107	TCCACCGCGC	740, 1297	10

4151	107	TCGGCGGTGG	949, 1210	10
4152	107	TGCCCTCGGC	2206, 2680	10
4153	107	TGGTGCCGGC	2554, 4077	10
4154	108	CGATGGACGTCG	2738, 2819	12
4155	108	CGCCAAGGCCGG	1989, 3503	12
4156	108	CTGCTCGACCGG	3633, 4536	12
4157	108	GTCAACAACGCC	3291, 3444	12
4158	108	TCGACGTCGACC	4278, 4549	12
4159	108	AGCCGGGCCAG	483, 2753	11
4160	108	CAGCGGCTGTT	2878, 4160	11
4161	108	CCGCGGCGGTG	1504, 1678	11
4162	108	CCGGGCGTGGA	2425, 2691	11
4163	108	CCTGGGCTGGC	2010, 3461	11
4164	108	CGAACAGCACC	101, 1459	11
4165	108	CGAGGCCAACC	2505, 4060	11
4166	108	CGAGGCCTTCG	1950, 3656	11
4167	108	CGATGTCACCG	595, 2196	11
4168	108	CGGTGGCCGCC	640, 901	11
4169	108	CTGACCGGCGA	2455, 3735	11
4170	108	TCCAGTCAGCA	3759, 4698	11
4171	108	TCCCGATCTCG	4270, 4792	11
4172	108	ACACGGCGGC	2028, 3428	10
4173	108	ACGGCGACCA	2282, 4876	10
4174	108	CAACGCGTCG	302, 1709	10
4175	108	CAGCGCCGCC	1403, 1640	10
4176	108	CCGCAGCGCG	1031, 1391	10
4177	108	CCGCGCCGAC	2085, 2898	10
4178	108	CCGCGTCGAG	1312, 3194	10
4179	108	CGACCACCGC	2264, 3256	10
4180	108	CGCCGCCGGC	1327, 3062	10
4181	108	CGCGGCCGCC	53, 3497	10
4182	108	CGGCATCGAT	622, 1363	10
4183	108	CGGCGCCGGC	1540, 2235	10
4184	108	CGGTGATGCG	559, 1006	10
4185	108	GATCCAGCGC	1744, 2931	10
4186	108	GCCGACAGCG	1197, 3932	10
4187	108	GCGTCGATGC	723, 4591	10
4188	108	GCTACTTCCG	3409, 4260	10
4189	108	GGTGCGCTGG	4327, 4515	10
4190	108	GTCGGCGAGC	2521, 4815	10
4191	108	GTTGTCGTAT	147, 2394	10
4192	108	TCGCCGAGCG	2948, 3891	10
4193	108	TCTCGCAGTT	258, 4891	10
4194	108	TGCCGTTGGC	814, 1171	10
4195	109	GGTCGGGCCCGCCG	3479, 4750	14
4196	109	ACCGCCGCGGCG	1276, 1659	12
4197	109	ATGCCCTCGTCC	1539, 4608	12
4198	109	CGATCGCGCCGT	1139, 1960	12
4199	109	GGAGAAGGTCAA	511, 3909	12
4200	109	CGACGGCGCTC	875, 1442	11
4201	109	CGCGTTCGCGG	82, 3119	11
4202	109	CGGGGCGATCG	532, 2014	11
4203	109	GGGCGAGCAGC	749, 1861	11
4204	109	GTCGCCGGCAT	1308, 2987	11
4205	109	GTCGGCATCGA	1802, 3555	11
4206	109	TGCCCAGCCCG	3106, 4540	11

4207	109	TGCCCGCCACC	2257, 2793	11
4208	109	AACGGCGGGG	527, 4677	10
4209	109	AGGGCGGCCG	219, 2673	10
4210	109	CACCGGCGGC	610, 2653	10
4211	109	CACGCCGAAC	3020, 3681	10
4212	109	CCAGCCCTCG	3825, 4124	10
4213	109	CCAGGACGAC	1058, 1122	10
4214	109	CCGATGCCGA	1833, 4848	10
4215	109	CCGCGTCGGC	1798, 3001	10
4216	109	CCGGTGCGGC	2525, 4189	10
4217	109	CCTTGCCGGC	1222, 4723	10
4218	109	CGAACTGCCC	893, 4535	10
4219	109	CGACCCGGTC	358, 2980	10
4220	109	CGACGACATC	430, 2041	10
4221	109	CGATCAGCGT	2053, 4456	10
4222	109	CGCACCGGCG	608, 2475	10
4223	109	CGCCCCGGCC	3146, 4637	10
4224	109	CGCCGAGGTT	3503, 4405	10
4225	109	CGGCGACGAC	869, 1155	10
4226	109	CGGCGGCCAT	234, 4336	10
4227	109	CGGCGTCCAG	2743, 4429	10
4228	109	CGGTGCGCAG	2512, 4792	10
4229	109	CTTGGCCGCG	2441, 2861	10
4230	109	GATGCCGCCC	738, 2178	10
4231	109	GCAGCCGGGT	1082, 3371	10
4232	109	GCCCCGACGG	1902, 3934	10
4233	109	GGCGACTGCG	1413, 4005	10
4234	109	GGCGTTGCCC	3660, 4388	10
4235	109	TCGAGCGATC	1134, 1614	10
4236	109	TCGCGGCTCG	1, 1503	10
4237	109	TTGGCGAACC	3532, 4656	10
4238	110	AGGTCGATGTCCC	4213, 4702	13
4239	110	CGACGCAGAATC	455, 1435	12
4240	110	CGTCGTCGCCGT	2001, 3733	12
4241	110	GGCGGCGGTGAA	294, 4680	12
4242	110	GGGATCGCCCTA	1000, 1117	12
4243	110	TCGCGGGAGATG	4495, 4645	12
4244	110	AACAACGCCGG	1106, 1939	11
4245	110	ACCTTGGCCGT	1346, 4252	11
4246	110	ACGACGCGGGC	2845, 4715	11
4247	110	CATCAGCGGCG	1546, 2699	11
4248	110	CCAGCCGCGCG	684, 1234	11
4249	110	CGACCAGGCCG	434, 4835	11
4250	110	CGATGGCGCCG	4115, 4808	11
4251	110	GATCATCAACA	1252, 2100	11
4252	110	GCAGCGCCGCC	1793, 2935	11
4253	110	GCCAAGGCGGG	1316, 2164	11
4254	110	GCGTCGCGGTG	49, 2887	11
4255	110	GGATGACCCGG	170, 2647	11
4256	110	GGGGCCTCGAT	1086, 4549	11
4257	110	GTCGCGCGCGA	198, 2741	11
4258	110	TCGCGCAGGTA	2874, 3356	11
4259	110	ACGACATCGG	1497, 1751	10
4260	110	ACGGGGGCCT	1083, 3425	10
4261	110	CACCCGATCG	3133, 3343	10

4262	110	CACCGTCGCG	1066, 2869	10
4263	110	CAGCGCGGTG	546, 4062	10
4264	110	CGCGCCGAGC	357, 2963	10
4265	110	CGCGGCGATG	2178, 3929	10
4266	110	CGTCACCGGC	904, 2379	10
4267	110	CTCGCGCAGG	3355, 4479	10
4268	110	CTCGTGCACC	249, 4155	10
4269	110	GATCCCGCGG	233, 2473	10
4270	110	GCCGCGTCGT	1661, 2047	10
4271	110	GCCGGCCGCG	651, 3866	10
4272	110	GCGGCCCTCG	606, 4581	10
4273	110	GGCCTGCAGG	2125, 3229	10
4274	110	GGCCTGGACG	1765, 1921	10
4275	110	GGTGGCGGCG	276, 291	10
4276	110	GTCGGGCAGG	2140, 4695	10
4277	110	GTTGAAGCCG	597, 4620	10
4278	110	TCGCCGAGGG	2423, 2618	10
4279	110	TCGGTGCGCT	562, 4042	10
4280	110	TCGTCACCGG	903, 1673	10
4281	111	CTGCTCAACTGGC	3545, 3704	13
4282	111	TCGACCACGCCGA	3483, 4667	13
4283	111	CCTGGACGCCGA	3249, 4362	12
4284	111	TCGACGAGGCCG	3962, 4097	12
4285	111	ACCCTGCAGCA	2954, 4468	11
4286	111	CCGAGCCGATC	489, 4511	11
4287	111	CGACGAGTACA	2174, 3100	11
4288	111	CGTCGACCACG	2011, 4665	11
4289	111	GCCGGTGCCCG	1772, 4482	11
4290	111	GCTCGCCGAGG	2390, 3646	11
4291	111	AGCAGGACCG	1924, 2763	10
4292	111	CACCGCCGAC	2093, 3694	10
4293	111	CATCGGCGCC	1471, 2273	10
4294	111	CCGACGACGA	1101, 2473	10
4295	111	CCGCACAGCT	1400, 2383	10
4296	111	CCGCGCCCGA	1242, 1554	10
4297	111	CGAGCAGGTC	841, 4170	10
4298	111	CGCCGACTAC	2321, 3151	10
4299	111	CGGACACCGC	1236, 3690	10
4300	111	CGGCGACGAG	3097, 4272	10
4301	111	CGGCTGTCGG	2034, 2451	10
4302	111	CGTCATGCTC	682, 3391	10
4303	111	CTGTTCGACG	83, 4093	10
4304	111	GACCTGACCC	2610, 2675	10
4305	111	GATGTCGCCG	2199, 4282	10
4306	111	GCGCAACGTC	676, 4659	10
4307	111	GCGGCGTCGA	2134, 3596	10
4308	111	GCTCGGCGAG	3508, 4227, 4647	10
4309	111	GGCGGCGTGA	1755, 3758	10
4310	111	GGGCGGCGTG	1955, 3757	10
4311	111	GGTCGCCACC	1817, 4122	10
4312	111	GTCGCCGACG	1695, 3587	10
4313	111	GTGACGGGGT	1795, 3465	10
4314	111	GTGGCGGCTG	2447, 3355	10
4315	111	GTGTGGCGGC	2445, 4424	10
4316	111	TCGGCGTCGG	2053, 2826	10
4317	111	TGGGTTTCGG	1879, 2859	10

4318	112	AGTCACGCTCGA	1508, 2429	12
4319	112	CCGACGGGGCG	957, 1263	11
4320	112	CCGAGCGTGAC	1492, 1522	11
4321	112	CGACGACGCTG	2582, 4834	11
4322	112	GCCGAGCCCGA	3076, 3288	11
4323	112	GCGCGCCGACC	4096, 4516	11
4324	112	GCGGCAGCGGC	2779, 3089	11
4325	112	GGCGCTCGCCG	571, 2484	11
4326	112	GGCGGCGGCGC	536, 1033	11
4327	112	TGCGGTCGCGG	1232, 1713	11
4328	112	AACGCCGGCG	677, 4421	10
4329	112	ACGGCGGCGG	534, 701	10
4330	112	CACGCGCGTC	1657, 2076	10
4331	112	CGCCGTTGCG	2327, 2965	10
4332	112	CGGCGGTGTC	2687, 4879	10
4333	112	CGTCGTCGCG	604, 3544	10
4334	112	CTTCGGCGCG	811, 4696	10
4335	112	GCCGAGCCGC	309, 920	10
4336	112	GGGCACCTCG	1378, 3102	10
4337	112	GTCGGTCAGC	1118, 4391	10
4338	112	TACGCCTGGG	129, 2294	10
4339	113	TCAACATCCCGGTCA	4169, 4280	15
4340	113	CACCGAGGACGAC	622, 4865	13
4341	113	CGACGCGGTGCTG	802, 850	13
4342	113	GCGCCGCCGCCGC	3062, 4060	13
4343	113	TCGCCGACCTCGG	1521, 4822	13
4344	113	CCAGGCGCTGGT	1867, 4266	12
4345	113	CGCCAGCACCTG	1433, 3405	12
4346	113	GACATCGACGTC	353, 782	12
4347	113	GACGGCAAGGTG	4402, 4686	12
4348	113	GTAGAACACGTT	184, 3510	12
4349	113	AGGTCGGCTTC	579, 2621	11
4350	113	AGTTCACCAAG	1611, 2111	11
4351	113	CGACATCGACG	781, 4554	11
4352	113	CGCATCGTCGT	3453, 4414	11
4353	113	GAGGTCGTCGC	827, 2830	11
4354	113	GCGACACCATC	714, 1713	11
4355	113	GCTGGCCGCCC	2484, 4775	11
4356	113	GCTTCCAGCTG	3845, 4313	11
4357	113	GTTCGACGACC	1837, 3903	11
4358	113	ACAGATCGCC	3921, 4817	10
4359	113	ACGCGATCGC	1755, 3569	10
4360	113	AGCTGATCAA	384, 2246	10
4361	113	AGGGCGTGGA	1263, 1632	10
4362	113	CAAAGAGGCC	2274, 2331	10
4363	113	CCCGGGTGCA	3261, 3782	10
4364	113	CGACCGCGAC	1747, 3696	10
4365	113	CGACGAGTTC	985, 1606	10
4366	113	CGACGTGGTG	934, 1777	10
4367	113	CGAGGGCGTG	1261, 2565	10
4368	113	CGCCGACCTG	598, 4763	10
4369	113	CGGCATCGAC	1198, 2793	10
4370	113	CTGATCGACA	3019, 3333	10
4371	113	CTTCGGCCTG	2409, 3185	10
4372	113	CTTCTTGATG	2292, 2448	10

4373	113	GACCGCCGCG	1948, 3218	10
4374	113	GACGACGTCG	157, 4803	10
4375	113	GAGGTCGAGG	572, 1892	10
4376	113	GCCGCCGAAG	58, 2821	10
4377	113	GCGCCACCAG	2103, 4638	10
4378	113	GCTGCCAGTG	2519, 3239	10
4379	113	GGCAGCGGGT	399, 4499	10
4380	113	GGCCCGGGTC	223, 4716	10
4381	113	GGCGCCAAGC	1652, 2689	10
4382	113	GGCGGCCAGG	1862, 3001	10
4383	113	GTGCCCGTCA	2164, 3883	10
4384	113	TCGTGGCCGC	2138, 3170	10
4385	113	TGACCGCGAC	1995, 3211	10
4386	113	TGCACCGAGG	620, 2380	10
4387	113	TTGCTCGACG	503, 4680	10
4388	114	AATCCATCCGCGCC	1772, 1786	14
4389	114	GACCGGGTGCGCGC	1969, 3162	14
4390	114	AGTGCTGCTCG	666, 2353	11
4391	114	CAGCAAGATGA	603, 714	11
4392	114	CGCGGACGTCG	798, 3142	11
4393	114	GCCGATCGGGT	4433, 4737	11
4394	114	GGCACCAGCTT	77, 3201	11
4395	114	GTACGGCACCA	382, 3197	11
4396	114	TCGTGGTCGTG	2260, 3111	11
4397	114	ACGCCCTCGG	2619, 4239	10
4398	114	ACGCGGACGT	797, 2089	10
4399	114	CACCGGCGCC	1383, 4061	10
4400	114	CAGTTGATCA	2736, 2787	10
4401	114	CCACCAGCTT	3355, 4201	10
4402	114	CCGCGCACCA	887, 3647	10
4403	114	CGACGCCGAC	690, 1209	10
4404	114	CGAGACGCTG	343, 1104	10
4405	114	CGATCTTGGT	4339, 4793	10
4406	114	CGCTGGCCGA	159, 1640	10
4407	114	GATGAGGATG	3017, 4274	10
4408	114	GCCGCCGTAG	2765, 4629	10
4409	114	GCCGGGATCG	3461, 4008	10
4410	114	GCCTCGGCGA	464, 4708	10
4411	114	GCGCGCAACG	2018, 3567	10
4412	114	GCTGCTGTCC	256, 759	10
4413	114	GCTGGGCGCC	121, 873	10
4414	114	GGAGCGGTTC	1434, 1677	10
4415	114	GGCCGCCGCG	3136, 4163	10
4416	114	GGGCGTCGGC	593, 4578	10
4417	114	GGGGCGCGCA	3564, 3951	10
4418	114	GTACTGCGAG	2894, 4617	10
4419	114	GTCGCCGCCC	3221, 4677	10
4420	114	GTCGCTCAGT	1888, 1950	10
4421	114	GTCGTCCACG	3153, 3876	10
4422	114	TCGGTGACGT	2976, 3700	10
4423	114	TGCTGCGGCA	204, 1073	10
4424	114	TGGCCTCGGC	462, 2162	10
4425	114	TGGTCGCCGC	3964, 4675	10
4426	115	GCGGCCGCGTCGC	57, 4606	13
4427	115	GGCCGCCGAGATC	3540, 3864	13
4428	115	AGTCGAAGGCCA	1419, 2853	12

4429	115	CATCTCGACGTG	1666, 4209	12
4430	115	CGCGGTGTGCAC	593, 4821	12
4431	115	GGAACGGGATGT	2106, 2175	12
4432	115	GGCGCCCACCGG	437, 2494	12
4433	115	TGGGCGCCGAGG	3509, 3758	12
4434	115	CCCGCGCACCC	2776, 4332	11
4435	115	CCGGTCGCCGG	1264, 2252	11
4436	115	CGATGGCACCG	358, 4422	11
4437	115	CGCATCCACCG	2563, 4803	11
4438	115	CGCGCCGTCGG	212, 4467	11
4439	115	CGGCGCCGTCG	1293, 2592	11
4440	115	CTACACGCCGC	3126, 3576	11
4441	115	GATGTGTTGTG	952, 4881	11
4442	115	GCCCAGCGACG	1192, 4257	11
4443	115	GTCGGCCATGA	638, 4521	11
4444	115	GTCGGCGCCGT	2263, 2590	11
4445	115	AGGTGGTCGA	3293, 3815	10
4446	115	CCCGGCCATC	1489, 2763	10
4447	115	CCGGCAGGTG	3366, 4586	10
4448	115	CCTCGTAGAG	1641, 1806	10
4449	115	CGAGCGCGCA	3435, 4126	10
4450	115	CGATGTCGGT	1779, 2481	10
4451	115	CGCAGCGCCG	123, 3703	10
4452	115	CGCGCCCAGC	1189, 2311	10
4453	115	CGGCACGTGC	2752, 3259	10
4454	115	GACGACGATG	2991, 4115	10
4455	115	GATCGCCGAG	1714, 4289	10
4456	115	GCGCCGCAGC	1324, 3642	10
4457	115	GGATCAGCGG	607, 2577	10
4458	115	GGGCGCGCAG	1093, 3027	10
4459	115	GTGCCGTCGG	1856, 4369	10
4460	115	TCCGACTGCT	495, 2432	10
4461	115	TCGCCGCGGC	1287, 3062	10
4462	115	TGACCGCGAC	349, 876	10
4463	115	TGGGCGAAGC	1817, 1886	10
4464	116	CCATCCTGGTGAACG	675, 3393	15
4465	116	ACGAGTACGACCTG	3780, 4041	14
4466	116	ACGAGGTCAAGC	4272, 4701	12
4467	116	CGAGATCGTCAT	721, 3352	12
4468	116	CTGGCGCACCAG	2605, 4019	12
4469	116	GAGCGCGTCGAT	1760, 2791	12
4470	116	GCAGCGGATGTC	703, 2914	12
4471	116	GCGGCGACGAGA	756, 3231	12
4472	116	GGCGAGAAGATC	959, 4481	12
4473	116	GTCCTCGGACAG	1868, 2338	12
4474	116	TCACGTTCGGCG	1337, 2256	12
4475	116	ACACCGAATTC	612, 927	11
4476	116	ACAGCGGCGGC	4212, 4473	11
4477	116	CGAGGACGGCA	3550, 4261	11
4478	116	CGCCGAGCGTG	1287, 2206	11
4479	116	CTTGGCCTCGG	2539, 2653	11
4480	116	GCCGCTGCTGG	1252, 4000	11
4481	116	GGCCGACGACG	1165, 4771	11
4482	116	TCAAGGAGAAG	357, 4104	11
4483	116	TCCACGAGCGC	3041, 3474	11

4484	116	ACCGACTTCG	3749, 4841	10
4485	116	ACGCCGAGCG	1241, 2205	10
4486	116	CAAGGACGAG	3775, 4342	10
4487	116	CCTCGAGCTG	1108, 4117	10
4488	116	CGACGAGGTC	3928, 4699	10
4489	116	CGAGGGCGAG	955, 3424	10
4490	116	CGATGCCCCG	1687, 1999	10
4491	116	CGGCGTGCGC	4291, 4384	10
4492	116	CGTCAAGGAC	2187, 3772	10
4493	116	CGTCGGAAAC	570, 4161	10
4494	116	CGTGCTGCCC	490, 3508	10
4495	116	CTACCTGCTC	3289, 3661	10
4496	116	CTATCGCTGG	130, 4735	10
4497	116	CTGGCGATGA	1368, 2151	10
4498	116	CTGTTCTCCA	233, 668	10
4499	116	GACATCTACC	3656, 3722	10
4500	116	GCCATCCTGG	203, 3392	10
4501	116	GCCCGCGCAG	2875, 3639	10
4502	116	GCGGTGATGG	587, 1481	10
4503	116	GCTGCGACCG	1189, 1773	10
4504	116	GGCGGCACCA	3677, 4178	10
4505	116	GGCGGTGATG	586, 2551	10
4506	116	TCAGCAGCAG	1609, 2571	10
4507	116	TCCGCCGATG	3814, 3838	10
4508	117	CCTCGGCGGCGTCG	3362, 4613	14
4509	117	CGCGGCGCCGGGA	2271, 3842	13
4510	117	CCGGATCGCCGC	2210, 4202	12
4511	117	CGACGGCGGCGT	24, 1598	12
4512	117	CGCCGGCGCCGC	156, 3744	12
4513	117	CTGGAATCCGAC	1893, 2775	12
4514	117	GCCACCGGCGCG	1713, 2223	12
4515	117	GCTGCTCGCCCA	624, 2645	12
4516	117	GTCGGCGGCGAC	2166, 3971	12
4517	117	ACCCCGGCACG	1627, 2395	11
4518	117	AGTTCGCCGAC	3031, 4672	11
4519	117	CCGGCCCGGCG	3003, 4652	11
4520	117	CGACGCCGTCG	348, 1973	11
4521	117	CGCCGGTGTCG	2374, 4703	11
4522	117	CGTCACCACCG	2843, 3014	11
4523	117	CGTCGACGCCG	345, 3158	11
4524	117	GACCACGCCAC	1857, 3670	11
4525	117	GAGTTCGGTGC	124, 4272	11
4526	117	GCGCCTGGCGC	908, 3404	11
4527	117	GCGCGGCGCCG	2270, 4484	11
4528	117	GCGTCGGCGGC	2568, 3969	11
4529	117	GGCACCCAGCC	3615, 4048	11
4530	117	GGTGGCGCCGG	2495, 2510	11
4531	117	ACTCGCCGCC	138, 306	10
4532	117	ATCTCGACGC	536, 2016	10
4533	117	CACCTCGGCG	1347, 3360	10
4534	117	CCACGCCGCC	672, 4787	10
4535	117	CCCAGCGAAA	3697, 4533	10
4536	117	CCCGCCGGCC	396, 4593	10
4537	117	CCCGCGGCGG	712, 2724	10
4538	117	CCGCGTCGGC	2566, 3527	10
4539	117	CCGGCTGGCG	584, 2297	10

4540	117	CGACCCCCGA	104, 830	10
4541	117	CGATCGCCGC	1113, 2743	10
4542	117	CGCGCCGCGC	685, 1819	10
4543	117	CGCTGCGACC	242, 575	10
4544	117	CGGATGCGCG	1677, 3566	10
4545	117	CGGCCTGGCC	1796, 2192	10
4546	117	CGGCGCGGCG	2459, 4482	10
4547	117	CGGGCGATCG	2607, 3721	10
4548	117	CTACATCGAC	1227, 1338	10
4549	117	GAACGGGCGA	1945, 2604	10
4550	117	GACCGGCGCA	2549, 3860	10
4551	117	GCAACACCCG	762, 4466	10
4552	117	GCGCGCCGCG	1818, 3047	10
4553	117	GCGGCCAGCC	1992, 3310	10
4554	117	GCGGCGGGGG	3990, 4017	10
4555	117	GCTGGCCCCG	288, 2111	10
4556	117	GCTGTTCGGC	519, 642	10
4557	117	GGTCGGCAGC	3114, 3381	10
4558	117	GGTGCTGTGC	2237, 2765	10
4559	117	GTACACCGCG	4431, 4881	10
4560	117	GTGGTGCCGT	1081, 3090	10
4561	117	TCGACGCCCT	1906, 4216	10
4562	117	TGCCCGCATC	529, 1736	10
4563	118	GATGTCGCGCTG	497, 1965	12
4564	118	ACCGTCGCCGC	1702, 2074	11
4565	118	ATCAGGTTGGT	2425, 3037	11
4566	118	CACCGCCCAGC	158, 852	11
4567	118	CAGCGCCTCGC	509, 3012	11
4568	118	CCAGCTCGCGC	816, 1800	11
4569	118	CCCGACGCTGC	1306, 4852	11
4570	118	CCGCGGCGATC	658, 2744	11
4571	118	CCTCGGTGCCG	1281, 2780	11
4572	118	CGCCGATCAGG	2420, 3439	11
4573	118	CGCCGTGCCGG	1381, 2973	11
4574	118	GACGCCGACGC	919, 1721	11
4575	118	GGCGCTCGGCC	3639, 4107	11
4576	118	GGCGGGCGACA	2163, 3756	11
4577	118	AAGCGCTGCG	237, 1203	10
4578	118	ACGCGTCGGC	1416, 2156	10
4579	118	ACGGCGCCGC	4058, 4364	10
4580	118	ATGCTGTTCA	3865, 4575	10
4581	118	ATTCCCGCCG	561, 3434	10
4582	118	CCCGCGGCGA	2743, 3498	10
4583	118	CCGCGTTGGA	178, 3692	10
4584	118	CCGGCACGCC	1557, 2837	10
4585	118	CCGGCGCCGG	1472, 2701	10
4586	118	CGCCGCCACC	152, 3945	10
4587	118	CGCCGCGCTG	988, 3487	10
4588	118	CGCCGTGCAC	930, 3906	10
4589	118	CGCGGTGGTC	2520, 3597	10
4590	118	CGGTGCGCGG	381, 896	10
4591	118	CGTCGATCTC	733, 1002	10
4592	118	CGTCGGCGGC	972, 1359	10
4593	118	GCCGAAACCG	1432, 1696	10
4594	118	GCGGACGTCG	86, 2445	10

4595	118	GCTCGACCCG	2030, 3424	10
4596	118	GCTGCAGCTC	1575, 3159	10
4597	118	GGAGGCCGGC	4020, 4200	10
4598	118	GGAGTCGGCG	413, 461	10
4599	118	GGGCCGAGGC	225, 3632	10
4600	118	GGTTGAGCTG	2249, 2378	10
4601	118	GTGAAGATCA	321, 2866	10
4602	118	GTGTGCGCCA	950, 4126	10
4603	118	TCGGCGAGCG	2885, 4444	10
4604	118	TCGTCGGTGA	777, 2860	10
4605	118	TTGCCGTGGC	273, 3749	10
4606	119	CGAGGAGATCGACGC	159, 1006	15
4607	119	GCCGGCGCCACCGC	112, 968	14
4608	119	GGCCACCATCGGC	955, 2189	13
4609	119	CGACGATCAACA	2134, 3042	12
4610	119	ACATCGCGGGT	1753, 3378	11
4611	119	CATCGCGCTGG	943, 4807	11
4612	119	CCGCAAGGCCG	2372, 3505	11
4613	119	CCTCGTCGGTG	1852, 2974	11
4614	119	CGCCGCCCGCT	1132, 3446	11
4615	119	CGTGCTGGCCG	1114, 3199	11
4616	119	GAACGTCTACG	2081, 4619	11
4617	119	GCCTCCGGCGG	821, 2457	11
4618	119	TCAACGGCTTG	254, 338	11
4619	119	TGATCGCCGGC	933, 1071	11
4620	119	ACACGTTGGC	2221, 4096	10
4621	119	ACGACGCCAC	597, 4195	10
4622	119	ATGTGCTGTC	4504, 4842	10
4623	119	CAACACCATC	2893, 3139	10
4624	119	CAACCTCAAC	3271, 4514	10
4625	119	CACCGAGCTG	2576, 3490	10
4626	119	CCAGCTGCTG	3163, 3427	10
4627	119	CCAGTCGCTG	1886, 3980	10
4628	119	CCGCTGCTCG	2358, 4644	10
4629	119	CCTCGGCGCT	3225, 3568	10
4630	119	CGCCTTCGCG	1556, 2627	10
4631	119	CGCGATCCGC	3723, 3992	10
4632	119	CGGCGACCAG	1679, 3974	10
4633	119	CGTCGCCGAC	1813, 4454	10
4634	119	CGTGATCAAC	519, 3262	10
4635	119	CGTGGACCAG	2153, 3328	10
4636	119	CTACGCCACC	4568, 4795	10
4637	119	CTGATCGCCG	740, 1070	10
4638	119	GAGCGCGTTC	1943, 3214	10
4639	119	GCCGAAGTCA	382, 4748	10
4640	119	GCCGAGCAGG	2235, 4329	10
4641	119	GCCGGTGGCG	3635, 4044	10
4642	119	GGCGCTGCTG	243, 393	10
4643	119	GGCGGTGCAC	1033, 1745	10
4644	119	GGCGTCGGCA	466, 1313	10
4645	119	GGCTGGTGGT	206, 2742	10
4646	119	GGGCATCCAG	1640, 4418	10
4647	119	GGTCGACGAC	646, 4349	10
4648	119	TCAACAAGCT	4339, 4489	10
4649	119	TCGACAAGCG	2919, 3320	10
4650	119	TCGGCAAGGT	1651, 3898	10

4651	119	TCGGCATCGA	185, 870	10
4652	119	TTCCTGCAGG	307, 1232	10
4653	120	CCCCGAGCTCGCCCG	2799, 2980	15
4654	120	CTACGACACCCCG	1014, 3676	13
4655	120	GCCAACGTCACCT	2067, 2627	13
4656	120	ACATCGACCTGG	1639, 2784	12
4657	120	CCCGCCGCCGCC	3838, 3970	12
4658	120	CCGCCGCTGATG	958, 3458	12
4659	120	CTGCGCCGGCTC	934, 2280	12
4660	120	ACCCGCCGCCG	3426, 3969	11
4661	120	CCGCCGGCGCC	3944, 4113	11
4662	120	CCGCCGGTGCC	3665, 3902	11
4663	120	CCGCGGCCGGG	863, 1228	11
4664	120	CGACGAGAGCG	702, 4566	11
4665	120	CGCCGGCCACG	1094, 1397	11
4666	120	CGTCACCGTCG	1487, 4761	11
4667	120	CGTCCCGCCGG	1134, 3733	11
4668	120	CTTCGCCGCCG	2108, 4108	11
4669	120	GCCCCCGCCGG	3781, 3940	11
4670	120	GCCGCCGCCGC	3454, 3862	11
4671	120	TCAACCCGCCG	2353, 3966	11
4672	120	TGGCGATCTTC	2490, 2532	11
4673	120	TGTCGGTGCGC	1781, 4751	11
4674	120	ACCTCAACGA	674, 3417	10
4675	120	CAACGACAAC	2153, 3850	10
4676	120	CAACTCGACC	645, 1592	10
4677	120	CACCTTCGAC	366, 2264	10
4678	120	CCAGCACCGC	3219, 4464	10
4679	120	CCCAGATCCG	3090, 3751	10
4680	120	CCCCAACTTC	2210, 3244	10
4681	120	CCGACGAGAC	1804, 3480	10
4682	120	CCGCACCGCG	240, 4375	10
4683	120	CCTGGTGGCG	156, 4386	10
4684	120	CGCCCAGACG	1086, 1610	10
4685	120	CGGCGCCAGC	219, 4366	10
4686	120	CGTGTTGCCG	3331, 3706	10
4687	120	CGTTGCCGGC	1502, 2599	10
4688	120	GAACGCCAAC	2623, 3031	10
4689	120	GAAGCCGTCG	4314, 4684	10
4690	120	GACCCTCAAC	597, 2147	10
4691	120	GATCACGGCG	987, 2506	10
4692	120	GCGGGCCGGC	1827, 4038	10
4693	120	GCTGCTCGTC	1128, 3322	10
4694	120	GGCGCGCGAC	1952, 4491	10
4695	120	TCCGGCGCCG	3811, 4133	10
4696	120	TCGTCGACAC	656, 4508	10
4697	120	TGACCAACGG	2826, 3690	10
4698	120	TGCGCGAGCT	404, 3492	10
4699	120	TGGCCAACCT	2383, 3273	10
4700	121	TTGTCCGCGGCGG	53, 4463	13
4701	121	ACGGCGCGGTGC	668, 4328	12
4702	121	ACGTCGTGGGCG	306, 1212	12
4703	121	ACGCCGCGACG	2704, 4298	11
4704	121	CCTCGGCGAGC	1171, 1678	11
4705	121	CCTGGCGGCGC	1228, 3234	11

4706	121	CGCCAACACCC	250, 3066	11
4707	121	CGCCCCGCGCG	1928, 2185	11
4708	121	CGGGGGTAGCG	3992, 4107	11
4709	121	CGTGGCGTTCG	37, 2306	11
4710	121	GCCGTGCTGCC	119, 2514	11
4711	121	GCGACGTCGTC	2337, 3533	11
4712	121	GCGCCAACACC	249, 1115	11
4713	121	GCGCGCCGCCG	1384, 1984	11
4714	121	TGTCGGCGGCG	195, 773	11
4715	121	ACGAACGAGG	3010, 4267	10
4716	121	CACCGGCGGG	2075, 4101	10
4717	121	CAGCTCCACC	1892, 2069	10
4718	121	CCCTCGACGA	2031, 3098	10
4719	121	CGATGGCGTC	1291, 3749	10
4720	121	CGCCGCGCCG	2449, 2886	10
4721	121	CGCGGCCCGG	2959, 4831	10
4722	121	CGCTGCGGCA	1399, 3815	10
4723	121	GACGACGACG	2231, 2564	10
4724	121	GATCACCGGC	4098, 4785	10
4725	121	GATCGGGTCG	83, 840	10
4726	121	GCCCGGGCAG	684, 3312	10
4727	121	GCGGTCCGGT	542, 3544	10
4728	121	GCTGCTCATC	7, 3759	10
4729	121	TCGCGAAGAC	830, 2897	10
4730	122	CCCGACGGCCGCC	2280, 3073	13
4731	122	GCCGCCCAGCGC	2235, 2791	12
4732	122	GGTTGGCCAGCT	1898, 4121	12
4733	122	ACCGAACCGCC	3635, 3662	11
4734	122	AGCGCGCCGAG	2302, 4646	11
4735	122	CCGCCCGAGCG	2377, 3695	11
4736	122	CGACACCACCC	343, 4794	11
4737	122	CGACGACGGCG	769, 3335	11
4738	122	CGCCGCCGTCG	127, 854	11
4739	122	CGGCTCACGCC	3623, 3650	11
4740	122	CGTGGTGGTGC	897, 939	11
4741	122	GGGGCGTTGGC	2978, 3010	11
4742	122	GGTCAGGAAGT	4299, 4506	11
4743	122	TCGCGGTCGCG	1153, 2564	11
4744	122	ACGCGATCGA	1121, 2865	10
4745	122	AGCCCGGCGG	2850, 3465	10
4746	122	CAACGCGGCG	307, 4524	10
4747	122	CACCGCCGAC	199, 4245	10
4748	122	CACCGCCGTC	2961, 4587	10
4749	122	CGACGCCACC	1323, 1736	10
4750	122	CGCCCGCCGG	2513, 3212	10
4751	122	CGGCGGCGGC	2946, 3939	10
4752	122	CGGCTGGGTG	281, 3850	10
4753	122	CGGGCCGCGC	326, 4228	10
4754	122	CTGCGGGCCG	323, 4839	10
4755	122	GATGTTGGTG	1527, 3177	10
4756	122	GGAATCGAGA	991, 2333	10
4757	122	GGCCAGGTTG	1083, 1773	10
4758	122	GGCGCCGGTT	2268, 4449	10
4759	122	GGGCACCGAG	790, 3909	10
4760	122	GGGCGCCGCC	124, 2372	10
4761	122	GGTCTCGAGC	1866, 2640	10

4762	122	GTAGTAGTAG	912, 3231	10
4763	122	TCGCTGGCGC	545, 1668	10
4764	123	CGCAGGGTGGCCAGAACGGTCCGCCGGCGCA	3128, 3236	31
4765	123	AATGGTCCGCCGGCGCAGGGTGGCCAG	3196, 3223	27
4766	123	CCCGCCGCAGGGCGGGCAGA	3336, 3435	20
4767	123	GGCCAGAACGGCCCGCCGGC	3271, 3298	20
4768	123	CGTGCTGGTGGCC	1664, 3801	13
4769	123	AACCGGCGAACG	4240, 4810	12
4770	123	CGCCGCGCGGGG	811, 3557	12
4771	123	CGCTGCCGCGGT	799, 2904	12
4772	123	GAACACCCCGCC	3117, 3405	12
4773	123	GCGGCCACGGCG	849, 3089	12
4774	123	GCGGCCAGGTAG	4173, 4338	12
4775	123	AAACCGCTGCG	215, 1693	11
4776	123	ACCCGCCGCAG	3386, 3434	11
4777	123	ATCGGCCGCGA	1164, 4070	11
4778	123	CCCGCCGCCGC	3648, 3702	11
4779	123	CCGGCGGCCAC	54, 3086	11
4780	123	CGACGCCGGAT	1522, 4357	11
4781	123	CGACGGTGACG	2096, 2213	11
4782	123	GCGGAACCGGC	1544, 2346	11
4783	123	GCGGCGCCGCC	567, 1704	11
4784	123	GCGTATTGGTC	175, 4089	11
4785	123	GGCCAGCGAGC	257, 1901	11
4786	123	GGCGGCCCGGG	362, 3067	11
4787	123	CCCGGCGGCC	35, 3085	10
4788	123	CCGCCTGGTG	90, 4375	10
4789	123	CCTGCGCGGC	844, 1327	10
4790	123	CGCGGTGGTG	745, 4016	10
4791	123	CGGCCAGAAC	2520, 3297	10
4792	123	CGGGGGCTGG	1641, 3735	10
4793	123	CGTCGGCGAC	373, 4648	10
4794	123	CTCGGCCAGC	1898, 4505	10
4795	123	CTGGTGATCG	2416, 2506	10
4796	123	GAACCGGCCG	1533, 3771	10
4797	123	GAGCGCGGTC	1021, 3373	10
4798	123	GCCCCGGCGG	33, 4281	10
4799	123	GCCGGGGCCG	2722, 4407	10
4800	123	GCCGTGCTGG	1493, 1662	10
4801	123	GCGGGTTCAC	415, 972	10
4802	123	GCTCGTCGGC	4399, 4645	10
4803	123	TGCCCGGCAC	1510, 1596	10
4804	123	TTCGCCGGCG	902, 2481, 4115	10
4805	124	CAAGGCGCCGCCC	1951, 4045	13
4806	124	CGGCCCCGGCACG	284, 4263	13
4807	124	GCGGTCGCCGCCG	269, 2264	13
4808	124	CCGCGTCGTCA	456, 3400	11
4809	124	CCGTCCTCGGC	1868, 1898	11
4810	124	CGATCGATCCG	146, 4223	11
4811	124	CGGACGGCATC	1306, 3482	11
4812	124	CGGTCAGCTTG	1818, 2545	11
4813	124	GGCCTGGGCCT	4513, 4683	11
4814	124	AACGGCCGCG	2203, 3792	10
4815	124	AGCGGCCCGT	1802, 2042	10
4816	124	ATCCTGCGGC	2838, 3328	10

4817	124	CAACGACCGC	3507, 4153	10
4818	124	CACCCGGGCG	2019, 4875	10
4819	124	CCTCGACGTG	2642, 4143	10
4820	124	CGAACTGCTC	193, 2534	10
4821	124	CGACGCGGTC	655, 4395	10
4822	124	CGCGGCGTGG	937, 3144	10
4823	124	CGGATCGAGA	899, 4081	10
4824	124	CGGGCGCGCG	2610, 4489	10
4825	124	CGGTTGCGGG	2688, 2826	10
4826	124	CGTCGTCGAG	2082, 2124, 3071	10
4827	124	GACCACGGCG	1182, 4572	10
4828	124	GCAGGGTGGC	160, 1719	10
4829	124	GCAGGTGCGG	1482, 4778	10
4830	124	GCCGAAGTCG	3778, 4737	10
4831	124	GCCGTCGTCC	172, 3748	10
4832	124	GGCGCTGGCC	3390, 4188	10
4833	124	GGCGGTCAGC	1816, 1966	10
4834	124	GGGGCGACGA	1162, 2776	10
4835	124	GGGGGCGACG	2775, 3057	10
4836	124	TCAGCCTCGA	2803, 3228	10
4837	124	TGACCACCAT	1686, 4010	10
4838	124	TGCGCGGCGT	3142, 3467	10
4839	125	CGATCTCGCCCT	2387, 3829	12
4840	125	CTCACTCCTCCT	160, 4235	12
4841	125	GCGCCCTGCAGC	1112, 1298	12
4842	125	TCGTCGGCGGAG	3498, 4029	12
4843	125	TGGCGTGGCCCT	2815, 3889	12
4844	125	CATGCCGGCCA	1177, 1968	11
4845	125	CCAGCACCTCG	3382, 4844	11
4846	125	CGAAGAAGTCG	2264, 3811	11
4847	125	CGACCTCGAAC	1425, 3229	11
4848	125	CGACGCCGACG	1329, 2489	11
4849	125	CGGCCATGCCG	1173, 1311	11
4850	125	GCGGGTTGCCG	954, 2092	11
4851	125	GGAAGTAGTCG	2785, 4448	11
4852	125	GGGAGCTCGAC	325, 690	11
4853	125	GGTGCCGTCGG	425, 2346	11
4854	125	GTCTTCGTCGG	4025, 4537	11
4855	125	TGCACGACCAT	1472, 2925	11
4856	125	TGCGCTTGCGG	1832, 2887	11
4857	125	CACCGCGGCG	1561, 4731	10
4858	125	CAGGTGATGG	351, 1777	10
4859	125	CCCCGCGGCG	2054, 4393	10
4860	125	CCGCCCAGCA	1571, 1733	10
4861	125	CCGCTGGCGC	4659, 4704	10
4862	125	CCTCGAGCTC	2630, 3370, 4583	10
4863	125	CGCACCGAAC	258, 3065	10
4864	125	CGGGTCGATC	1258, 1444	10
4865	125	CTCGATCAAC	2283, 3419	10
4866	125	CTTCGCCGAG	2135, 4310	10
4867	125	GAGCTTGCCG	2229, 4809	10
4868	125	GATCTCGTCG	314, 2481	10
4869	125	GCCCGCAGGA	1635, 4441	10
4870	125	GCTTCGGTCA	588, 2108	10
4871	125	GGGTCGGTGC	3699, 4155	10
4872	125	GGTGACCTCG	1165, 3491	10

4873	125	TCAAACCGAT	3766, 4144	10
4874	125	TCAGGTCGCC	2210, 4694	10
4875	125	TCATCTGCTC	3901, 4228	10
4876	126	CGAGCTGGGCGGC	3061, 4282	13
4877	126	GGACGGTCATCGC	1426, 4826	13
4878	126	CGTCGCGGCCGC	3220, 4734	12
4879	126	GCGGACCTGGTC	935, 4421	12
4880	126	GGTGTCGGCGCG	2096, 2942	12
4881	126	GTCGCCGACATC	3872, 4373	12
4882	126	TGGCCGACGGCG	3024, 3921	12
4883	126	CATCACCCCGT	914, 2752	11
4884	126	CGACGACGCCG	3094, 3505	11
4885	126	CGCGATCACCA	710, 3166	11
4886	126	GCCGTCGGCGT	44, 2732	11
4887	126	GGGCTGGGCAA	3428, 4597	11
4888	126	ACGCCGAGGC	3834, 3885	10
4889	126	ACGTCGGTGA	888, 1803	10
4890	126	CAAGGTCGGC	3787, 4243	10
4891	126	CCATCGCGGA	1618, 2562	10
4892	126	CGACCGGGCG	3006, 3574	10
4893	126	CGACGGCGAC	3000, 3496	10
4894	126	CGAGGCGTCG	2683, 4459	10
4895	126	CGCCGACGTC	788, 4728	10
4896	126	CGGACACGGT	544, 1588	10
4897	126	CGGCGCTGAC	1720, 3562	10
4898	126	CGGCGGCGTG	2302, 3628	10
4899	126	CGGCGTTGGC	283, 673	10
4900	126	CGTCGCCGAC	4372, 4725	10
4901	126	CTCGGCGATC	1847, 3334	10
4902	126	GATGGCCGAC	3022, 3985	10
4903	126	GCGCGCGAGG	3452, 3848	10
4904	126	GGCGATCTCG	1073, 1517	10
4905	126	GGCGGCATCG	3815, 4013	10
4906	126	GGGCGGCATC	3238, 3814	10
4907	126	GGTGGTGGTC	692, 3865	10
4908	126	GGTGTCGTAG	521, 575	10
4909	126	GTCGACGGCG	2998, 4493	10
4910	126	GTCGCCGTCG	503, 761	10
4911	126	TCACCGGGTC	2991, 3801	10
4912	126	TCATAGCCGC	303, 4517	10
4913	126	TCTTCATCGA	2319, 3402	10
4914	127	CACGCCGGAACCG	3657, 4320	13
4915	127	CGGTCCCGAGGTG	2789, 3149	13
4916	127	GTCGTCGACGGCG	3174, 3960	13
4917	127	TAGCCGCCGTCGA	3910, 4705	13
4918	127	GCTTCGGCGATC	1540, 3442	12
4919	127	GTGCGCCGCGAG	2356, 4740	12
4920	127	CAGCGGCATGT	2040, 4506	11
4921	127	CGATCAGCGCG	4451, 4646	11
4922	127	CGCCGAGGCGG	236, 2196	11
4923	127	CGCCGGCACCG	2386, 2687	11
4924	127	CGCCTCGCCGC	3036, 4827	11
4925	127	GGACGCCGCCC	2867, 2972	11
4926	127	GTGGCCGCGTG	3703, 4861	11
4927	127	TCCTCGGGTGG	3608, 3696	11

4928	127	ACATCGTCGA	7, 3325	10
4929	127	ACGAGGTCTA	561, 3298	10
4930	127	CATCGTCGGC	3314, 3626	10
4931	127	CCGAGGCCGA	1420, 3637	10
4932	127	CCTCGGGGTG	491, 2810	10
4933	127	CGAAATCGTC	2945, 3686	10
4934	127	CGACAAGCGC	670, 2463	10
4935	127	CGAGCTGCGC	529, 3542	10
4936	127	CGATGTAGCG	1577, 1907	10
4937	127	CGCCTCGAGT	4791, 4851	10
4938	127	CGCGGATCGC	3068, 3889	10
4939	127	CGCGGCGGGC	1808, 3509	10
4940	127	CGGCGATGCG	3005, 4106	10
4941	127	GAGCCGCCGC	1173, 1741	10
4942	127	GATCTGCAGG	255, 4572	10
4943	127	GCGCCGACCT	2770, 3733	10
4944	127	GCGCGGTCGA	843, 4444	10
4945	127	GGCGCCGACC	75, 3732	10
4946	127	GGTGCTGCTG	2172, 3125	10
4947	127	TCAACGGCGA	753, 1397	10
4948	127	TCCACGCCGG	139, 1553	10
4949	127	TCGTGCGCCG	2191, 2354	10
4950	127	TCTCGTGCGC	1253, 2189	10
4951	127	TGTTCGGGCC	4232, 4495	10
4952	127	TTCGCCAAAG	1965, 2898	10
4953	127	TTGCCGCTGA	1375, 1774	10
4954	128	GATCGCCGCGGTGCG	921, 4231	15
4955	128	GTCGACACCGGCG	1402, 2937	13
4956	128	CCGCGTCGACCG	400, 2333	12
4957	128	CGCTGGCCGGTC	2755, 3369	12
4958	128	GCCGCCGAGCTG	1669, 4025	12
4959	128	GCGGCGGCGCAG	610, 2155	12
4960	128	TGGGTGCCGGCC	1390, 2701	12
4961	128	CGTTACGCCCG	595, 3046	11
4962	128	CTGTCGCCGCA	778, 2564	11
4963	128	GACGGCGACGC	2898, 3710	11
4964	128	GGCGGCCCTGG	4384, 4456	11
4965	128	GGGCGCGGCCG	1332, 4426	11
4966	128	TCGGCGAAACC	30, 91	11
4967	128	ATCCGGCGGT	3443, 4167	10
4968	128	CCGCAACGCG	2397, 2826	10
4969	128	CCGCCCAGGC	2924, 3558	10
4970	128	CGACGGCGAC	1115, 2897	10
4971	128	CGAGGTGGCG	1818, 4450	10
4972	128	CGCCACGACG	485, 3332	10
4973	128	CGCCGAGGGC	1469, 1683	10
4974	128	CGCCGCCACG	1289, 3038	10
4975	128	CGCCGCCCGC	1178, 2391	10
4976	128	CGGCGCCGAG	1466, 1484	10
4977	128	CGGCGCGGCG	1502, 2351	10
4978	128	CGGGCAGGTG	3790, 4797	10
4979	128	CGTCGACGGC	1112, 1148	10
4980	128	CGTCGATGGC	3861, 4017	10
4981	128	CTCGAAGAGC	50, 134	10
4982	128	GCCGCGAGGC	4257, 4616	10
4983	128	GCCGTTGCGG	107, 2035	10

4984	128	GCCGTTGGCG	1740, 2664	10
4985	128	GCGATCGCCG	3260, 4789	10
4986	128	GCGCGGTCGA	1793, 2102	10
4987	128	GCGGGCCGGA	1728, 1868	10
4988	128	GCGGGCCGTT	1553, 2660	10
4989	128	GCTGGCGGAC	1893, 4402	10
4990	128	GGCACCTGGG	60, 1384	10
4991	128	GTGCGCGGCG	2376, 2418	10
4992	128	TGATGGCCGG	3531, 3885	10
4993	128	TGCCGTTGGA	349, 4371	10
4994	129	CGACGTCACGCTG	551, 1087	13
4995	129	ACGTCTTCATCG	3491, 4172	12
4996	129	CCGGTTCGCCGA	877, 2923	12
4997	129	CGGTCAGCGATC	1747, 4373	12
4998	129	CTGCGCGACCGC	1256, 3038	12
4999	129	GACCGCGACGGC	1454, 3134	12
5000	129	ACCCGCCGACC	1939, 3764	11
5001	129	CACCGCGACGA	2099, 4411	11
5002	129	CCGGTCGAATG	798, 2813	11
5003	129	CTGTGGCGCGT	1427, 4501	11
5004	129	GCCTGGCTGGC	1547, 2205	11
5005	129	GCGCGAGCAAC	2005, 4692	11
5006	129	GGCGACCGGCA	925, 2869	11
5007	129	GGCGCGGGTGC	1165, 3991	11
5008	129	GGTCCGCGCTG	1248, 1951	11
5009	129	GTGGTGGGCGG	1978, 4300	11
5010	129	TATCGCCCGGA	659, 4717	11
5011	129	ACCTCACCCT	513, 4034	10
5012	129	ATCGCGTCCA	252, 3719	10
5013	129	CAACGCGGTC	401, 2039	10
5014	129	CACCGACCGC	2617, 3130	10
5015	129	CACCGATCCG	4227, 4860	10
5016	129	CCGCGGTGTC	2161, 2465	10
5017	129	CCGTGGCCGC	1371, 3343	10
5018	129	CCTGGGCGCC	713, 3855	10
5019	129	CGACGAGATC	3160, 3621	10
5020	129	CGACTTCGTC	2638, 4662	10
5021	129	CGCCACCCGC	542, 1935	10
5022	129	CGCCGTGGCC	1534, 2266	10
5023	129	CGTGATCGCC	1318, 3540	10
5024	129	GAGCTGTATC	2576, 3961	10
5025	129	GAGGCCTACG	3359, 4165	10
5026	129	GATCCCGACG	1082, 3748	10
5027	129	GCCGACGAGA	3158, 4123	10
5028	129	GCTGCGCGAG	3516, 4689	10
5029	129	GGCGCACCGC	535, 1237	10
5030	129	GTGGGTGGCG	1159, 1381	10
5031	129	TCGGTGCCGT	775, 1176	10
5032	129	TTCACCCCGG	2942, 3170	10
5033	130	CGATTGAACTGATTGGTCAGGATCA	925, 2683	25
5034	130	TCGATGAAGGTGCGTTGCGCGACG	2083, 3841	24
5035	130	CGGCAGCAGCATGCCGCCGCC	1845, 3603	21
5036	130	GTTGATCAGGAAGATCCA	1686, 3444	18
5037	130	CGCTTGGTGCCGAACCG	1969, 3727	17
5038	130	CTGGAAGTAGCTCGG	1320, 3078	15

5039	130	ACCAGCATCGTCAC	1372, 3130	14
5040	130	CCACCAGCACGAC	746, 2498	13
5041	130	CCGGGGGAGAGCA	1576, 3334	13
5042	130	CGATCAATGCCAG	1475, 3233	13
5043	130	TTCGCTTGCCGGC	863, 2448	13
5044	130	CCGAGCCGGGCG	666, 3556	12
5045	130	CGCGCACAGCAG	1917, 3675	12
5046	130	CTGCATCGGCGT	1293, 3051	12
5047	130	ACGACCGCCTG	2056, 3814	11
5048	130	ATCGGGATCAC	2005, 3763	11
5049	130	CCGATCGGCAG	1267, 1675	11
5050	130	CCGCCGATCGG	1234, 3505	11
5051	130	CCGGCCTCACG	1813, 3571	11
5052	130	CGCCACCAGCA	744, 2862	11
5053	130	CGCCGACGGCG	614, 3353	11
5054	130	GCGCCGGTGGC	4075, 4396	11
5055	130	TCCGGCGATCC	407, 2157	11
5056	130	ACGTCGAGGC	388, 4267	10
5057	130	AGCGCCGCGG	1048, 4450	10
5058	130	AGCGCCTGCA	1036, 2794	10
5059	130	AGGACGCGAA	2419, 3827	10
5060	130	AGGCGTGCGC	782, 3212	10
5061	130	CATGATCGCG	1092, 1644	10
5062	130	CATGGCGCCG	2271, 3012	10
5063	130	CGATCAGCCA	1721, 3479	10
5064	130	CGGCCCCGAT	962, 1256	10
5065	130	CGTCATCGTC	825, 3798	10
5066	130	GAACAGCCGC	1413, 3720	10
5067	130	GACGACGGTG	732, 2109	10
5068	130	GCAAGATGTT	1898, 3656	10
5069	130	GCCATCAGGG	907, 1174	10
5070	130	GCCGGCGCCG	3102, 4071	10
5071	130	GCGGCCCCGA	961, 1360	10
5072	130	GGATTGCGGA	2594, 3163	10
5073	130	GGGTGTTCTC	2261, 4694	10
5074	130	TGCCGCGACG	2209, 2661	10
5075	131	GGCCGCGCCGCCG	1690, 4251	13
5076	131	CGCCGCGCGGCG	665, 3612	12
5077	131	ACGTCGACGGC	836, 3754	11
5078	131	CGCCGTCGCCG	2007, 2817	11
5079	131	CGCGCTGGTCG	525, 2460	11
5080	131	CGGCCGCGCCG	4250, 4586	11
5081	131	CGGCGGTCTGT	228, 2157	11
5082	131	GATCAGCCGGG	3370, 4455	11
5083	131	GCACGGTCAGC	2088, 2960	11
5084	131	GGCACGCTCGA	1048, 3421	11
5085	131	GGCGGTCGGCA	4368, 4554	11
5086	131	GGTGATCGTGA	726, 3160	11
5087	131	GTGATGCCCAA	3173, 4660	11
5088	131	GTGGACGCCGC	3436, 3607	11
5089	131	GTGGCAGTTGC	2736, 4859	11
5090	131	ACAAGCTGCC	1295, 2402	10
5091	131	ACGGCCGCGT	3944, 4837	10
5092	131	CCCGGTTTCG	1030, 2633	10
5093	131	CCGCCGGGGC	1210, 4355	10
5094	131	CCGCGCTCGC	2585, 4798	10

5095	131	CGACTCGGCG	2688, 3307	10
5096	131	CGATCCGGGT	1069, 4436	10
5097	131	CGCCGGGGTG	1927, 4726	10
5098	131	CGCGCGCTGG	1648, 1863	10
5099	131	CGGCCCGCGG	1412, 2777	10
5100	131	CGGGGGCGAT	1830, 3566	10
5101	131	CGGTGCTGGA	644, 4034	10
5102	131	CGGTTGCCGG	77, 1009	10
5103	131	CGTCGGCGCG	3217, 3462	10
5104	131	CTGACCGGGC	1159, 3994	10
5105	131	CTGGTCGCCG	130, 2437	10
5106	131	GACGGCCGCG	3943, 4248	10
5107	131	GACGTGGTCG	2230, 4881	10
5108	131	GATCGCGGCC	154, 2793	10
5109	131	GCAGGCGTTC	210, 1224	10
5110	131	GCATCGGGGG	746, 1826	10
5111	131	GCCGCCGGGG	1925, 4354	10
5112	131	GCCTGCGGCA	4071, 4690	10
5113	131	GGCCCGCGCT	521, 2582	10
5114	131	GGCGAGATCG	2788, 4779	10
5115	131	GGCGGGGGCG	1627, 3564	10
5116	131	GGGCGCGCCG	2140, 3048	10
5117	131	GGGTGCTGAT	167, 2240	10
5118	131	TCGAGGAGCT	2645, 3851	10
5119	131	TCGGTGCTGG	1781, 4033	10
5120	132	AGCCAGCCGTCC	1137, 4247	12
5121	132	CGCGCCGATTCC	2003, 4843	12
5122	132	GATCAACACGTC	1232, 2984	12
5123	132	GCAGGTTGGGCG	487, 790	12
5124	132	ACCGTGACACC	24, 4454	11
5125	132	ATCGGAAACCG	17, 636	11
5126	132	CGCGCCGCGCA	1904, 4264	11
5127	132	CGCGGTGACGT	737, 2885	11
5128	132	CGCTGAGCCCG	811, 4488	11
5129	132	CGGTGAGCCCG	58, 4761	11
5130	132	CTTGCGGGCCA	878, 2834	11
5131	132	GCCATCGTCCA	1808, 4399	11
5132	132	GCCGCCGGCGC	394, 600, 4615	11
5133	132	GCGGTCGACGG	2942, 3811	11
5134	132	GTGCGTCAGCC	2679, 2927	11
5135	132	TCGCCGGCGAC	2277, 3164	11
5136	132	TCGGCGAAGTG	174, 2919	11
5137	132	TCGTCGGTGAG	54, 2256	11
5138	132	AACGGTTCCA	375, 4520	10
5139	132	CAGCTCGCTG	3769, 4390	10
5140	132	CAGGGCGGGA	3274, 4717	10
5141	132	CCAGCACCAC	1063, 2452	10
5142	132	CCATGCCGTT	523, 2136	10
5143	132	CCCGCACCGC	1422, 1978	10
5144	132	CCCGGTGCGG	1927, 3360	10
5145	132	CCGACGCGAC	103, 561	10
5146	132	CCGTCGGCCA	114, 159	10
5147	132	CGCCCTGCCG	554, 2173	10
5148	132	CGCGCGGTGA	735, 4757	10
5149	132	CGGCGTAGAG	3951, 4239	10

5150	132	GACCGACGCG	101, 3562	10
5151	132	GATGTGGCGC	3198, 3640	10
5152	132	GCCGGCGGCG	2726, 4234	10
5153	132	GCCGTCGCGG	3031, 3065	10
5154	132	GCGCCGCAGC	3589, 4438	10
5155	132	GGCCGCTTCG	3456, 4054	10
5156	132	GGGGCGGTCG	1190, 2939	10
5157	132	GGTGATCCCG	1828, 2375	10
5158	132	GTCGCCGGCG	3163, 4231	10
5159	132	TCGCCGTCGC	435, 3063	10
5160	132	TCGTGCGCGG	2997, 3928	10
5161	132	TGACGTCGCC	2215, 4227	10
5162	132	TGCCCCGGGT	895, 2644	10
5163	132	TGGTCGCCGA	1653, 3843	10
5164	133	GCTGTCGGCGATCCAC	1256, 2734	16
5165	133	GGCGCGGGCGCGT	189, 1893	13
5166	133	ATGACGACTTCG	1980, 3818	12
5167	133	GACTTCGGCGGC	1461, 4625	12
5168	133	CATGTCCAGGG	3241, 4082	11
5169	133	CCGCGGCCTCG	3747, 4693	11
5170	133	CGTCGGTGTCC	1702, 2040	11
5171	133	GATCGTGATGA	1097, 1490	11
5172	133	GCATGTCCAGG	3240, 3520	11
5173	133	TCCAGCCCGCC	3191, 3875	11
5174	133	ACCGCGAACG	3533, 4152	10
5175	133	AGGTCGTCGT	3854, 4371	10
5176	133	ATCCGGCCGC	1001, 3296	10
5177	133	CACTCGCCCC	139, 3761	10
5178	133	CAGGTCGCGC	3325, 4580	10
5179	133	CATCAGCCGG	2983, 4682	10
5180	133	CCGATCGACC	1163, 2837	10
5181	133	CCGGCGGCCA	3419, 4362	10
5182	133	CGACGGCCGG	1048, 3939	10
5183	133	GCCGCCGCGG	3226, 4834	10
5184	133	GCGCGGCCAC	1555, 3666	10
5185	133	GCGCGGCGAA	4142, 4510	10
5186	133	GCGGCAGGCT	348, 1760	10
5187	133	GGATCCGCCG	4616, 4787	10
5188	133	GTCGGGATCG	1619, 1958	10
5189	133	TGCCCGATCG	214, 2834	10
5190	133	TGCGGCGGCC	5, 1241	10
5191	134	CGGGCTCGCCGA	2065, 3310	12
5192	134	CTGTTCGGTGAC	4526, 4739	12
5193	134	TCGGTGCCGTCG	4629, 4878	12
5194	134	CCGTCCGGGTT	2212, 4866	11
5195	134	CGGTGTCCGCG	1090, 3593	11
5196	134	CTGGGTGATGA	1175, 3852	11
5197	134	GCCGACGGGTC	1197, 3434	11
5198	134	TGGTGCGGCCG	2434, 4717	11
5199	134	ACGGATGCCT	3535, 3921	10
5200	134	ACGGGACCGC	3194, 4180	10
5201	134	CCCGAGCTGC	689, 3636	10
5202	134	CGCATCGGCG	1, 2105	10
5203	134	CGCCGCCGCC	3993, 4376	10
5204	134	CGCCGCGACC	841, 1589	10
5205	134	CGGCGACGGG	1918, 4175	10

5206	134	CGGCGCGCCG	444, 1232	10
5207	134	CGGGTCGCCG	1481, 4496	10
5208	134	CGTCACCGGG	2059, 3376	10
5209	134	CGTGATGCCA	988, 3020	10
5210	134	GCCGTCGATG	2366, 3684	10
5211	134	GCGCCCGGCG	665, 4215	10
5212	134	GGCCGTCGAC	1513, 3815	10
5213	134	GGCCGTCGAT	2365, 3243	10
5214	134	GGGCCGTCGA	3242, 3814	10
5215	134	GGTCGGCATC	93, 3112	10
5216	134	GTCGGCGAAG	403, 800	10
5217	134	GTTGCGCAGC	2632, 4514	10
5218	134	TCCATGTTGG	461, 3753	10
5219	134	TCGCCGCCGC	3655, 3992	10
5220	135	ACCCTAGCGTGGCGACGGTGAGCGCCGGAAGCGGCGCGA GCAAGGAGCGCGCAATCG	434, 491	57
5221	135	CGGCATCGGCGGC	4604, 4799	13
5222	135	GCGGCGCTGGCCG	2735, 3719	13
5223	135	GATCGCGTCGAG	1940, 2048	12
5224	135	ACACCGAACTG	722, 4243	11
5225	135	ACCGTCGAGCA	3080, 4131	11
5226	135	ACCTGCGCTAC	1637, 4309	11
5227	135	CAAGGTGCACC	1623, 4301	11
5228	135	CAGGTCCCCGG	133, 2273	11
5229	135	CGCGGGGCCGG	1608, 2491	11
5230	135	CGGATCGACGA	1036, 1743	11
5231	135	CGGTGCGGGCG	776, 989	11
5232	135	CTCGGCCCGGA	112, 3239	11
5233	135	GATGGGCGCGC	1839, 4511	11
5234	135	GCCAGCGCCGG	2359, 3629	11
5235	135	GCTCGAGCCGT	918, 3175	11
5236	135	TCGAGGATCGC	2463, 3480	11
5237	135	TCGCGCTGGCC	1676, 3669	11
5238	135	TGACCGTCGAG	3078, 3474	11
5239	135	ACAACTCGGC	2419, 3813	10
5240	135	CAGGGCGCGG	1360, 4464	10
5241	135	CATCGACGCG	654, 3277	10
5242	135	CCGCCGCGGC	1664, 2179	10
5243	135	CGACGGCCGC	1653, 2216	10
5244	135	CGCCGCGGGC	376, 561	10
5245	135	CGCCGGCAAC	4676, 4754	10
5246	135	CGCGTTGTCG	2683, 3877	10
5247	135	CGCTGCTGGC	1343, 1523	10
5248	135	CGGGATCGCG	1014, 1275	10
5249	135	CGGGCGGGCG	2168, 2575	10
5250	135	CGGTGATGTG	3936, 4264	10
5251	135	CTCGGCGACG	2285, 2333	10
5252	135	GCCTCGGCGA	2040, 2331	10
5253	135	GCGTCGGTGT	2855, 4705	10
5254	135	GGCGATGCAC	1815, 1872	10
5255	135	GGCTGGACGG	410, 4792	10
5256	135	TCGGCGAGAC	1145, 4321	10
5257	135	TGCCGGTGAT	2641, 3933	10
5258	135	TGCGCGCACC	1770, 4109	10
5259	135	TGGGGCGTCG	1138, 4225	10

5260	135	TTCGGCGGGA	196, 807	10
5261	136	AGCGCCGGCATC	1486, 2738	12
5262	136	GACCGGGTCGAC	889, 2860	12
5263	136	GGCACCGAGCCG	977, 3454	12
5264	136	TGCTGGCCACCG	270, 3642	12
5265	136	ACGGTGGCCGC	1731, 3315	11
5266	136	CACCGTGCTGG	565, 1988	11
5267	136	CGAGGTGACCG	289, 2884	11
5268	136	CGCCCTGGCCG	694, 1207	11
5269	136	CGCGCTGAGCG	2836, 2983	11
5270	136	CGCGGCTGCCG	441, 1582	11
5271	136	CGTCGTCGAGG	1195, 2760	11
5272	136	CTCCGAGTCGG	1276, 1453	11
5273	136	TGCTGGCCCAC	2065, 4361	11
5274	136	ACCGCCCAAG	1078, 3898	10
5275	136	ACCGTGGTCG	335, 3014	10
5276	136	AGGGCCTGAT	4298, 4862	10
5277	136	CCACCCCGGT	735, 912	10
5278	136	CCGGCCTGGG	969, 1618	10
5279	136	CCGGCGACGA	462, 4327	10
5280	136	CCTGGGCGCG	2434, 4350	10
5281	136	CGCGGCCGGC	3094, 4218	10
5282	136	CGGCCGCGCT	2832, 4547	10
5283	136	CGGCGGCCGT	3477, 4817	10
5284	136	CGGTTGGCTG	2012, 2024	10
5285	136	CTGGACCGCG	1094, 4516	10
5286	136	CTGGCTGCTG	1808, 3634	10
5287	136	CTGGGTGGTG	62, 4527	10
5288	136	GAGTCGGGCA	4804, 4840	10
5289	136	GCGGCGCTGG	1866, 4399	10
5290	136	GCTGCGGATC	310, 3538	10
5291	136	GCTGCGTCTT	3691, 4664	10
5292	136	GGCCGCCAGC	2590, 2710	10
5293	136	GGCGGCGCTG	1604, 4398	10
5294	136	GTGTTGGCCG	2106, 3413	10
5295	136	GTTCGATGTG	1335, 3742	10
5296	136	TCCGCGCCGG	1797, 2564	10
5297	136	TCGCCGGCCC	1573, 3573	10
5298	136	TCGGCGGCGA	1719, 3032	10
5299	136	TGTCGCCGAG	2412, 2700	10
5300	137	TCGCCGTCGTGGCC	1537, 4863	14
5301	137	CACCCCGGCCTG	1050, 2574	12
5302	137	CGCCATCGTCAG	977, 4167	12
5303	137	GCGCGTTGCTCG	1021, 3091	12
5304	137	TCGTCGACGACT	88, 3234	12
5305	137	AGCCGTGGCCG	2515, 3598	11
5306	137	CATCGGTGTCG	1097, 2498	11
5307	137	CGCCGTCGAGG	155, 2684	11
5308	137	CGCGGCCTCCG	341, 2178	11
5309	137	CGGCACCTGGA	1436, 1590	11
5310	137	CGGCGCCGTCG	152, 1788	11
5311	137	CTCGGCGGGCA	2208, 2709	11
5312	137	CTGGGATTCGC	4211, 4694	11
5313	137	GCCCCCGAGCT	2892, 4365	11
5314	137	GCCGCCAGGAT	3809, 4786	11
5315	137	GGTGCGCGGGC	3554, 4628	11

5316	137	GGTTGTGCACC	367, 2387	11
5317	137	TCGCGGCCTCC	340, 796	11
5318	137	TCGGCCGCACC	949, 1264	11
5319	137	AAGCCGCTGG	696, 1849	10
5320	137	ATCGCCGTCG	942, 1536	10
5321	137	CCGAGTGTGC	3195, 3210	10
5322	137	CCGCGCGTTG	1565, 3089	10
5323	137	CCGGATCTGC	3505, 4578	10
5324	137	CGAGGCGGCC	2279, 3534	10
5325	137	CGGACAGCTC	1833, 2702	10
5326	137	CTGCTGGCCG	3748, 4844	10
5327	137	CTGGCGGTCG	828, 3018	10
5328	137	GACCACGATC	3066, 4835	10
5329	137	GCCGCCGTCG	4487, 4820	10
5330	137	GCGACTACCG	775, 4013	10
5331	137	GCGCGCCACG	612, 2860	10
5332	137	GGAAGAGCTG	1280, 4316	10
5333	137	GGCCAGATCG	2787, 4520	10
5334	137	GGCGCCCTCG	2445, 2964	10
5335	137	GTCACCGGCG	1365, 2604	10
5336	137	TGCTCGAGGC	274, 1719	10
5337	137	TGGGAGCGGT	3055, 3270	10
5338	137	TGGGCGTCGG	1522, 2037	10
5339	138	CGCTCCTCCCCATCGCTGCGCTCTGGATCGTCGCCGGCGG GGGTCATGTGCGCCGCTCCTCCCCATCGCTGCGCTCTGGA TCGTCGCCGGCG	202, 308	92
5340	138	ACGCACGTCGACGAGGT	1200, 2152	17
5341	138	CCTGGCCATCGCCG	757, 2869	14
5342	138	GCCGCCGCGGCGG	2066, 4224	13
5343	138	GCCCGGCCCGCG	4548, 4874	12
5344	138	TCAGCACGCCGG	3108, 4372	12
5345	138	TGCGGCCGCCGT	1275, 1352	12
5346	138	ACGCCGACCAG	2, 1847	11
5347	138	ACGTTGATGGC	1105, 1222	11
5348	138	ACTCGTCCGAC	712, 4578	11
5349	138	CCCGGATGCTG	1256, 2768	11
5350	138	GACAGTGCGCG	630, 1321	11
5351	138	GACGACGCCGG	1623, 4837	11
5352	138	GACGTCGGAGC	1674, 2968	11
5353	138	GCACCACGGGG	496, 2667	11
5354	138	GGCCGCCGCGG	2699, 4223	11
5355	138	GGCCGGGACGG	3073, 4003	11
5356	138	GGCTGCGTGGC	2171, 4311	11
5357	138	TCCAGGTCGAC	804, 2572	11
5358	138	CACCGCCCGG	148, 4303	10
5359	138	CCGTCGATGT	2500, 4452	10
5360	138	CCTTGCGCAC	3585, 3705	10
5361	138	CGACGGCGTT	604, 3477	10
5362	138	CGCGAAGGCG	2288, 3040	10
5363	138	CGCGCCACCC	743, 2860	10
5364	138	CGCGCCCTCG	46, 2983	10
5365	138	CGGCAAGGTG	2194, 4258	10
5366	138	CGGCGGCGAA	1866, 1980	10
5367	138	GACGCGGTGA	3181, 3251	10
5368	138	GACGGCGCCG	2023, 3970	10

5369	138	GATCGCGCCG	1563, 3646	10
5370	138	GATGACGTCG	1236, 2965	10
5371	138	GCGTCACGAC	401, 598	10
5372	138	GGAGATGCCG	1467, 3857	10
5373	138	GGGCGCGACG	1969, 2998	10
5374	138	GTCGGCGGCG	2457, 2730	10
5375	138	GTTGGCGGTG	61, 4105	10
5376	138	TCCGGCCCGG	2749, 3098	10
5377	138	TCGATGATGA	2960, 3347	10
5378	138	TCGTCGGGGG	1189, 3146	10
5379	138	TGCGCGAACT	4727, 4790	10
5380	138	TTCGGCGGCA	2885, 4436	10
5381	139	GTGATCGCCGCGATC	2183, 3898	15
5382	139	CATCTACGGCGCC	2473, 4191	13
5383	139	CGCTGGCGCTGG	2706, 3524	12
5384	139	CGGCGGCGGCAC	2590, 3384	12
5385	139	GGCGACCGAGGA	3564, 4716	12
5386	139	ACGACGCCGAG	259, 1848	11
5387	139	CCGGCGGCGGC	2589, 3275	11
5388	139	CGCGCTGGGCG	2374, 2806	11
5389	139	CGGGCGCGTCG	24, 4152	11
5390	139	CTCACTGCGCG	185, 4356	11
5391	139	GCGCGACCGCG	127, 2236	11
5392	139	GGTGTGCGCCG	3324, 4219	11
5393	139	GTCGCTGTTCG	2341, 2869	11
5394	139	TGCTGATCATC	2169, 2829	11
5395	139	AACGCCATCA	1064, 1712	10
5396	139	ACCACCGACA	1463, 2279	10
5397	139	AGGTCAAGGA	1950, 4385	10
5398	139	ATCGTCACCG	2645, 3682	10
5399	139	CACCCGCGGC	688, 1669	10
5400	139	CAGGGCGGCG	889, 1538	10
5401	139	CATCGTCACC	3681, 4578	10
5402	139	CCGACACCGC	202, 571	10
5403	139	CCGGGGCGGC	772, 4657	10
5404	139	CGACGACACC	2085, 4677	10
5405	139	CGGCATCGTC	2614, 3678	10
5406	139	CGGTGACCGT	2325, 3194	10
5407	139	CGGTTGGACC	3985, 4396	10
5408	139	CGTGCTGCTC	4170, 4479	10
5409	139	CTCGGGCGCG	2041, 2210	10
5410	139	GCAGGGCGGC	311, 888	10
5411	139	GCCGCGGCGC	974, 2410	10
5412	139	GCGCCGCGGC	2408, 2913	10
5413	139	GGCGCCGCTG	3168, 3729	10
5414	139	GTCGGGCGCC	1138, 2944	10
5415	139	TCGGCGAGGG	1548, 1737	10
5416	139	TGCGCAATCG	3756, 4319	10
5417	140	CGGGGAATTCGGGCT	2360, 2375	15
5418	140	CCGCTCGGCGAGG	679, 961	13
5419	140	CGAGCCGCTCGG	469, 675	12
5420	140	CGTGGTGACCTG	975, 1428	12
5421	140	CAGCGTGTCCG	1341, 2746	11
5422	140	CGGCCAGCAGC	2123, 3217	11
5423	140	CGGCGATCATC	3772, 4334	11
5424	140	CGTTCTGGTCG	1085, 1175	11

5425	140	CGTTGGTGACG	599, 3988	11
5426	140	GCGGCGGTTCG	2303, 2579	11
5427	140	GGTGGTCGCGG	900, 3797	11
5428	140	TCGTCGGGGGC	859, 3843	11
5429	140	TGCGTGGTGGC	928, 2757	11
5430	140	CCGCGGCGGC	1158, 3526	10
5431	140	CGCGATCTCG	4434, 4824	10
5432	140	CGGGCGATCT	204, 3459	10
5433	140	CGTCGAGGTG	3562, 4298	10
5434	140	CTTGCCGGTG	1755, 4122	10
5435	140	GCGGATGTCG	1992, 3179	10
5436	140	GCGTCGTAGA	1735, 3402	10
5437	140	GCTCGACGGC	2534, 4103	10
5438	140	GTCGACGGCG	1593, 2169	10
5439	140	TCCGGCGTCG	1000, 3557	10
5440	140	TCGTCGCCGT	1168, 1966	10
5441	140	TGCAGCTCGA	2293, 4099	10
5442	141	ACCGCGCGCACG	739, 814	12
5443	141	GAAGGTCGGCGG	2813, 4851	12
5444	141	GCAGACGTCGTC	723, 4243	12
5445	141	GGCATCGGCCAG	2715, 3746	12
5446	141	CGTCGCCGCGG	2456, 4309	11
5447	141	CGTCGGCCGCC	2872, 3391	11
5448	141	CGTGCTCGGCG	2492, 2648	11
5449	141	CGTGGTCGTGC	2256, 2316	11
5450	141	GCGGGGTCGTC	683, 1342	11
5451	141	GCGGTCATCGG	3009, 4364	11
5452	141	TCCACCTTCTT	1287, 1479	11
5453	141	ACCGTCGTCT	2860, 3818	10
5454	141	ACGTGGTCGT	2255, 2533	10
5455	141	CCACGTTGCG	3296, 4568	10
5456	141	CCAGCGGGCG	1963, 3535	10
5457	141	CCCGTCGTCG	2221, 2451	10
5458	141	CCGAGCCGAC	3442, 3888	10
5459	141	CCGCTGGTGC	1989, 3587	10
5460	141	CCGGCCTGGT	2186, 2752	10
5461	141	CCTAAGCTGA	960, 4541	10
5462	141	CGAACGGTTT	1511, 4281	10
5463	141	CGAGTTGCCC	437, 1706	10
5464	141	CGGGGACCCC	2036, 2474	10
5465	141	CGTCCGCCTG	3619, 4834	10
5466	141	CGTGCCGATG	2199, 2304	10
5467	141	CTGCACGCGG	2703, 4271	10
5468	141	GACCGGCGCC	1064, 1376	10
5469	141	GCCGATCCGG	313, 522	10
5470	141	GGCGTGGGCG	2155, 3192	10
5471	141	TCCCAGTCCT	21, 2959	10
5472	141	TGTCACGGCG	881, 4738	10
5473	142	CAACGTCTTAC	1222, 4347	11
5474	142	CGACGAGTTCG	536, 4831	11
5475	142	GCGGCGACTTC	616, 3451	11
5476	142	GCTTGAGCCAC	1190, 3158	11
5477	142	TAGCGGAGAGG	629, 941	11
5478	142	TCGGATGCCGC	1146, 4800	11
5479	142	CGATGTCGCC	515, 581	10

5480	142	CGCCGGCATC	657, 4187	10
5481	142	CGCCTCGATC	3616, 4031	10
5482	142	CGCGGCCAAG	956, 2516	10
5483	142	CGGGAAGTCC	4308, 4449	10
5484	142	CGGGTTGATG	690, 1564	10
5485	142	CGTAGCGGAG	182, 627	10
5486	142	CGTCGCGATC	1004, 1957	10
5487	142	GAATTCGGCC	701, 3676	10
5488	142	GCGGCCGGAT	2844, 2995	10
5489	142	GCGGTGACAG	4, 142	10
5490	142	GCGGTGGCCA	1712, 4758	10
5491	142	GCTCGCGGCC	1936, 2991	10
5492	142	GTAGCGCGCC	1113, 1283	10
5493	142	TCAGTAGGTG	2670, 4885	10
5494	142	TGTCATCGCC	3635, 4025	10
5495	142	TTGAGGTCGA	508, 2009	10
5496	143	GCGAGCTGGCTGA	2405, 2570	13
5497	143	CGACGAAGACGG	3471, 4224	12
5498	143	AATTGCGACAC	3086, 3725	11
5499	143	CAACAGTACTA	424, 4588	11
5500	143	CGATGGTGGCG	2694, 3588	11
5501	143	GTGATTCGACG	701, 3153	11
5502	143	ACACCGGTGG	2375, 3435	10
5503	143	AGTGTGCCGA	932, 1868	10
5504	143	ATGTCGGCGG	914, 2450	10
5505	143	CCGTGCGCAG	1710, 2874	10
5506	143	CGCCGTAGGC	111, 4801	10
5507	143	CGCTACGTTG	837, 4559	10
5508	143	CGGCATCGGC	2097, 2976	10
5509	143	GCCCGTCACG	592, 1431	10
5510	143	GGCGCTGGAT	2019, 2287	10
5511	143	TCGTCCAGGT	3110, 4885	10
5512	144	CGGCGAAGGTGCG	2883, 4174	13
5513	144	CTGCGGCCCTGGC	3363, 4668	13
5514	144	CGGGCGGTCGAC	3488, 3842	12
5515	144	GATTCCCAGTCG	1270, 2804	12
5516	144	TGATCACCAATC	288, 3764	12
5517	144	TCCCGAACCGC	99, 264	11
5518	144	AATCGGCCGG	2384, 3098	10
5519	144	CACCACGCTG	603, 771	10
5520	144	CCGGCGTTGG	1495, 4416	10
5521	144	CGAAATCGGC	137, 3095	10
5522	144	CGAGCCGCCC	4277, 4683	10
5523	144	CGATCGAAGT	1350, 1848	10
5524	144	CGGCCCCGCC	3128, 3148	10
5525	144	CTGCCGCCGA	3171, 3222	10
5526	144	GCCCTCGGCG	3250, 3537	10
5527	144	GCCGAGTCGG	2945, 3061	10
5528	144	TCCAGAGGTG	115, 2664	10
5529	145	GTTGGCCAGCGCC	3082, 3184	13
5530	145	CGGGCGCCAGGC	2718, 2763	12
5531	145	CGTGTTCGGGCG	2310, 2757	12
5532	145	ACCTCGACGAC	1585, 3458	11
5533	145	ATCGCCGCGGG	4010, 4373	11
5534	145	CCGGCTGGCAC	1889, 1932	11
5535	145	CGACGTTCCTG	1717, 2197	11

5536	145	CGATCTTCGAG	1252, 4092	11
5537	145	CGCCGCGCGGG	4070, 4799	11
5538	145	GGGTGCGGCCA	1961, 4734	11
5539	145	GGTGCAGGTCC	1772, 3612	11
5540	145	GGTGGCCTCGA	533, 3250	11
5541	145	TCGGTGCGCCG	2873, 3901	11
5542	145	TGCGCGAGCAC	2029, 2621	11
5543	145	ACGCGCTGCG	3799, 4209	10
5544	145	ACTTCACCGA	1000, 4467	10
5545	145	AGCTGCGCGA	2618, 4515	10
5546	145	CCCTGGGTGA	75, 3113	10
5547	145	CCGGTGACGA	716, 4085	10
5548	145	CCGTCGCGCG	3024, 4562	10
5549	145	CGACGTCGCG	817, 1013	10
5550	145	CGCCGGCCCC	3226, 3584	10
5551	145	CGCCGGCCTC	466, 4330	10
5552	145	CGCGGTCTCG	203, 383	10
5553	145	CGGCGACGGC	1620, 2940	10
5554	145	CGGCGCGGCG	2646, 4442	10
5555	145	GAGGTGCAGG	1116, 3610	10
5556	145	GCCGGCTTCG	4235, 4812	10
5557	145	GCCGGGCGCC	1526, 2716	10
5558	145	GCGATCGCCG	1452, 4007	10
5559	145	GCGCTCGGCG	224, 3744	10
5560	145	GGCGCGCCGC	586, 2073	10
5561	145	GGTGAGCACC	2926, 3154	10
5562	145	GGTGGCCGCG	494, 3403	10
5563	145	GTCACCGCCG	303, 4388	10
5564	145	GTCGTCCTCG	347, 2824	10
5565	145	GTGCCGGTCG	2575, 2794	10
5566	145	GTGGCCGTCA	1095, 2173	10
5567	145	GTTCATCGCC	320, 1391	10
5568	145	GTTCCCGACG	1473, 4839	10
5569	145	TCGGGGTTCC	567, 1468	10
5570	146	TTCGGCACCGCCGA	823, 2423	14
5571	146	CGGCCGCATGACG	113, 2968	13
5572	146	CCATCCCGCACG	1325, 4110	12
5573	146	CTCGCCGACGGC	2927, 4780	12
5574	146	GCTCGCCGCCGC	49, 1393	12
5575	146	GGCATCCCGCTG	2285, 2612	12
5576	146	TCCGCGACGCCG	971, 2883	12
5577	146	ACCGGGCCAGT	560, 660	11
5578	146	ATGGGCCGGCG	1296, 2307	11
5579	146	CCGCCGCGGTG	1576, 2020	11
5580	146	CGCTGGCGTTG	1885, 3297	11
5581	146	CGGGACCATCC	2728, 4105	11
5582	146	CGTGGCCAGTG	1221, 3340	11
5583	146	GACGGCGGCGT	1502, 3063	11
5584	146	GCGAATGGTGG	423, 1175	11
5585	146	GCGATCGGTGA	2162, 4819	11
5586	146	TGGCCGACTTC	1762, 2586	11
5587	146	ATCTCGGTCG	2861, 4088	10
5588	146	CCAGGTCAAG	127, 2202	10
5589	146	CCCTGCGTGA	923, 3909	10
5590	146	CCGACGCGAC	354, 3393	10

5591	146	CCGATGCCGG	4723, 4862	10
5592	146	CCGGTGCGCA	2871, 4023	10
5593	146	CCTGGCCGAC	280, 2584	10
5594	146	CGATGTCGTC	870, 3586	10
5595	146	CGGCGGCGAT	2104, 2658	10
5596	146	CGTCGCCCGC	1050, 3898	10
5597	146	CTACCTGCTG	639, 2458	10
5598	146	CTGATCGCCG	531, 3488	10
5599	146	GAGGCGCTGA	2322, 3383	10
5600	146	GCACGTCGCC	900, 1969	10
5601	146	GCGCCGCCGA	2832, 4068	10
5602	146	GTCGGTGGCG	1871, 2488	10
5603	147	TTTCGCGCAACAAGTCGACGTT	1371, 2011	22
5604	147	AATCGCCGATTTCGC	2002, 2517	15
5605	147	CCGAGCGTCGAGT	1337, 2493	13
5606	147	CGAGCTGGTCGC	245, 1639	12
5607	147	CTACTCGTCGGC	781, 2185	12
5608	147	GATCATGTTGTC	3117, 3948	12
5609	147	GGTGGTTTCGAG	1073, 3870	12
5610	147	GTGGGCGAATTC	1652, 1817	12
5611	147	CACAGCAGGTC	3070, 4753	11
5612	147	CACCGACGACG	2164, 2413	11
5613	147	CGCCGGCGGCG	433, 2750	11
5614	147	CGCGACGAGGT	2216, 4868	11
5615	147	GATGTCGATGC	2263, 2838	11
5616	147	TCGAGGAACGG	1800, 4674	11
5617	147	CACCATGCTG	361, 385	10
5618	147	CAGTTGCCGG	2351, 4330	10
5619	147	CCCTCTTCGA	4246, 4300	10
5620	147	CGCCTGCTCG	96, 2658	10
5621	147	CGGCGGCGGG	2202, 3894	10
5622	147	CGTCGGCGCC	457, 2744	10
5623	147	CGTGGCGCAG	2855, 3740	10
5624	147	GCGCGGCCGG	139, 1155	10
5625	147	GGAACTGCCG	1330, 1951	10
5626	147	TCGCCGCGGA	292, 1053	10
5627	147	TGCGGTCGAG	2984, 4427	10
5628	148	GTAGGAGTGATCGCGAACGCGGGCGAAGCCCGGGTGAAG CGGGTCACGA	3104, 3161	49
5629	148	CGCGCACGCCGTCG	6, 1672	14
5630	148	CGGCGAGGCGATC	753, 957	13
5631	148	CGAACAGCTCGA	2888, 4106	12
5632	148	GCCAACGTCGCG	499, 3960	12
5633	148	GCCAAGGAGTTC	2094, 3708	12
5634	148	GGTCGGCGTCGC	710, 1654	12
5635	148	CAAGCAGATCG	1255, 1720	11
5636	148	CCACCGCCCGC	2428, 3373	11
5637	148	CGAGGAGCTGG	2243, 2822	11
5638	148	CGTCGGGCACG	2726, 2963	11
5639	148	GCCGACGATCT	1190, 1382	11
5640	148	GGAGACGGGCG	306, 660	11
5641	148	GGCCGACCCGG	81, 3785	11
5642	148	TGGCCGACCCG	3625, 3784	11
5643	148	TGGCCGACTGG	2137, 2497	11
5644	148	AGATGATCAA	845, 2611	10
5645	148	AGCCGGGCGC	2392, 3030	10

5646	148	CCTGCGCCGC	100, 1966	10
5647	148	CGCGGCCAAG	2534, 3980	10
5648	148	CGCGGCGCGC	1460, 1667	10
5649	148	CGGCATCGGC	1927, 2300	10
5650	148	CGTCGACGAG	1819, 2237	10
5651	148	CTCGCTGGAC	186, 2924	10
5652	148	GCCGACGGCG	3261, 3660	10
5653	148	GCCTGCTGCT	2509, 3697	10
5654	148	GCGCGCCACC	1882, 4473	10
5655	148	GCTGTCGGCC	3917, 4000	10
5656	148	TACGGCAAGC	1715, 2772	10
5657	149	TCGCGGCGATGTGCG	1690, 1855	15
5658	149	GAGCAGCGCGACG	2008, 4307	13
5659	149	CCCTGGTCGGCG	2117, 3823	12
5660	149	CGCCTCGGCGAC	3860, 4367	12
5661	149	CGCGACACCAGC	846, 1401	12
5662	149	CGGCGGCGGGAT	1838, 1871	12
5663	149	GCCGCGGCGTTG	1503, 4795	12
5664	149	GTCGAGGCGATC	1611, 3628	12
5665	149	GTGGCGGTCAAC	389, 2488	12
5666	149	CATGATCATCG	1926, 4716	11
5667	149	CCTCGTCGAGG	1995, 3624	11
5668	149	CGTCATCGACA	607, 4884	11
5669	149	CGTCGCGGCGA	1781, 1853	11
5670	149	GACCGCGCGCG	1948, 3979	11
5671	149	GCCGACAGTGT	2967, 4491	11
5672	149	GCCGACCGGCT	2773, 3718	11
5673	149	GCGCCGCCGCG	1829, 3501	11
5674	149	GGCGATGGATC	3663, 4823	11
5675	149	TCACCGCGGGC	982, 1468	11
5676	149	TCGACCCGGCG	904, 1582	11
5677	149	TGGCCACCATC	1549, 1705	11
5678	149	ACCCTGTTCG	572, 3319	10
5679	149	ACGGTGATCG	1893, 4592	10
5680	149	ACGTCGCCGC	1125, 3557	10
5681	149	ATCGCCGCCA	1311, 2575	10
5682	149	CCCGCGCCCG	2041, 2660	10
5683	149	CGACGACAGT	343, 4397	10
5684	149	CGCCGCCGTC	1241, 2391	10
5685	149	CGCCGCGCAC	745, 2789	10
5686	149	CGTCGCGGCC	4096, 4782	10
5687	149	GATCGCCGCC	2388, 2574	10
5688	149	GCGCACCACG	870, 4409	10
5689	149	GCGCGGCGGG	1030, 1973	10
5690	149	GCGCTGCCGA	2962, 3266	10
5691	149	GCGCTGGCCA	1545, 3286	10
5692	149	GCGTCATCGA	606, 2612	10
5693	149	GCGTCGCGGC	1852, 4781	10
5694	149	GCTCGACGAC	337, 4394	10
5695	149	GGCCGAGACC	120, 2544	10
5696	149	GGCGATCGGC	2031, 2328	10
5697	149	GGGGCAGTCG	3309, 4470	10
5698	149	GTCATCGCCG	1095, 3217	10
5699	149	GTCGACGACG	918, 2100	10
5700	149	TCGGCACGGC	820, 3116	10

5701	149	TCGGTCGACG	1380, 3541	10
5702	149	TGGCCGCGCT	1423, 1634	10
5703	149	TGGGGCAGTC	2369, 3308	10
5704	149	TGGTGGGGGC	2321, 3791	10
5705	150	GGCGATCCCGGCGG	1483, 4653	14
5706	150	GCAACGCGTCGG	700, 4167	12
5707	150	ACGATGACCGC	318, 4732	11
5708	150	CCCGTCGGCCA	1174, 3118	11
5709	150	CGGCCGCCCGT	2621, 3989	11
5710	150	CGGCGCCAAAA	1256, 2751	11
5711	150	CTCCGGCCGAT	2089, 4018	11
5712	150	GGTGCCCATCG	1699, 2971	11
5713	150	TCGGGCCGGCG	1250, 1748	11
5714	150	CAGCCCGTCG	1171, 3556	10
5715	150	CATGGCCATG	53, 3544	10
5716	150	CGAAAACCAT	2184, 2817	10
5717	150	CGACGCGCCC	4288, 4854	10
5718	150	CGCAGGTCGG	1148, 1814	10
5719	150	CGCCCGCGCC	2844, 4599	10
5720	150	CGCGCCGCGC	94, 2635	10
5721	150	CGCGTCGCGC	1717, 3669	10
5722	150	CGCTGGAAGC	2075, 2459	10
5723	150	CGGCGCGCAA	619, 694	10
5724	150	CGGCGTCGGG	761, 1383	10
5725	150	GCATCGAGAT	589, 1209	10
5726	150	GCCCCAATGA	1504, 1930	10
5727	150	GCTCGCCCGG	2861, 4619	10
5728	150	GCTGCAAGAC	149, 377	10
5729	150	GGCGATGAGC	435, 4767	10
5730	150	GGGGCCGCGC	3601, 3971	10
5731	150	GTCGTCACCC	3061, 4576	10
5732	150	TCCACCGACG	24, 4283	10
5733	150	TGCTGCGCGG	225, 3414	10
5734	151	GCTGCTGGCCGGC	1530, 2043	13
5735	151	AGGTCGGCGCCG	1811, 4685	12
5736	151	CTCAAGCTGATC	4000, 4333	12
5737	151	GCGCCGCCGTCG	669, 3288	12
5738	151	GCGCGGTGGTGG	1283, 4505	12
5739	151	GGCGCGCGCTTC	1073, 2575	12
5740	151	TCCTCGACGCGG	1750, 3117	12
5741	151	CCGGCGCGCGC	1071, 2276	11
5742	151	CGACGTCCTCG	1212, 1745	11
5743	151	CGAGACCGACG	1206, 1896	11
5744	151	CGCCGAGGCCT	460, 3846	11
5745	151	CTGCTCGACGA	2473, 4705	11
5746	151	CTGCTGCACGG	89, 437	11
5747	151	CTGGTGGTCGA	1765, 3871	11
5748	151	GACGCCGACGG	1603, 4483	11
5749	151	GCGGCTGGGTG	651, 4548	11
5750	151	ACCCCGACGT	684, 2633	10
5751	151	AGGTGATGCC	1086, 3003	10
5752	151	ATCCGCGCGG	1279, 4669	10
5753	151	ATTCCTGGAG	3541, 4077	10
5754	151	CAACCCGGCG	3520, 4644	10
5755	151	CACCCCGACC	68, 1960	10
5756	151	CCACCCCGAC	682, 1959	10

5757	151	CCGACGCTGG	995, 3374	10
5758	151	CCGGCGGCTC	720, 4151	10
5759	151	CGACGACGAG	1341, 4212	10
5760	151	CGAGCTGCTG	85, 1527	10
5761	151	CGCAAGCTGG	2556, 4201	10
5762	151	CGCCGGCGAC	3094, 3493	10
5763	151	CGCCTGCATC	1142, 2739	10
5764	151	CGCGATCGGG	3241, 4386	10
5765	151	CGGCGGCGGC	1629, 3918	10
5766	151	CGGCTGGCGC	4417, 4593	10
5767	151	GACCTGCTCG	2287, 4702	10
5768	151	GACCTGGTCG	508, 857	10
5769	151	GATTTCGATC	2942, 3778	10
5770	151	GCAGGGCTAC	361, 2088	10
5771	151	GCCGCGTGAT	1715, 2243	10
5772	151	GCTGCGCACC	601, 1353	10
5773	151	GGACGCCAAG	1123, 1431	10
5774	151	GGGTTCGACC	3900, 4576	10
5775	151	TCCAGCCGCT	3177, 4232	10
5776	151	TCGTCGATGC	1517, 3468	10
5777	151	TGTGCTGGAA	2334, 2510	10
5778	151	TGTTCGGCGA	3195, 4367	10
5779	152	TTCGGCATCATGCAC	830, 1394	15
5780	152	CCCAAGCCGCTG	1100, 2594	12
5781	152	CCGTTGATCGTC	2216, 4865	12
5782	152	ACCGACGAGCA	393, 1343	11
5783	152	ATCGGCGTGCC	174, 4380	11
5784	152	CCGACGACGAG	651, 4240	11
5785	152	CCGCCGACAAG	699, 981	11
5786	152	CGAACGCATCG	880, 4373	11
5787	152	CGCTTCAACGA	213, 3088	11
5788	152	GCCGCGGCCAG	791, 4308	11
5789	152	GCGCAGCGGGC	1874, 4834	11
5790	152	GCGCCACGCCG	2475, 4333	11
5791	152	TCTACGAATTC	618, 3665	11
5792	152	ACGTCGTCGG	40, 2052	10
5793	152	ATGCCGGCGC	490, 1079	10
5794	152	ATGGCGGAGA	1841, 2987	10
5795	152	CAACCCGAAC	305, 1291	10
5796	152	CAACGGCTAC	1237, 2425	10
5797	152	CCGCCGGGCG	205, 1975	10
5798	152	CGACCCCGCC	976, 4226	10
5799	152	CGGGCAGGGC	1369, 2569	10
5800	152	CGTGGGCGCC	642, 2710	10
5801	152	GAATCGGGTG	123, 4770	10
5802	152	GACCGGGGTG	1417, 3020	10
5803	152	GACGCGCTGT	2168, 3139	10
5804	152	GAGAACGGCG	1262, 4666	10
5805	152	GCAGGCGGCG	1867, 2629	10
5806	152	GCCATCGGCG	1181, 1307	10
5807	152	GGCCGCGGCG	1505, 1799	10
5808	152	GGGCCGCTGG	2805, 4813	10
5809	152	GGTGCGTCAG	2023, 2923	10
5810	152	GTGGTCAACC	854, 4527	10
5811	152	TCATCACCGA	10, 2846	10

5812	153	CCGCGGCGGCGA	2388, 4609	12
5813	153	CCGCTTCGGCGA	1447, 4219	12
5814	153	CCGTCCGCGGCG	205, 3726	12
5815	153	CGCGGCGCGGGC	2359, 4208	12
5816	153	CCGCCGTGCGG	4242, 4789	11
5817	153	CCTCGGCGACC	2739, 4474	11
5818	153	CGGCCAGCGCG	320, 4750	11
5819	153	CGGCCGTGGCC	2373, 4138	11
5820	153	GCAATGCCCGC	149, 2456	11
5821	153	GCAGGGCGTCG	254, 3517	11
5822	153	GCGCGGTCGGC	2573, 4638	11
5823	153	GCTCGATCAGG	3742, 4777	11
5824	153	GTCGTTGCGCA	888, 1290	11
5825	153	TCGTCGAGGAC	688, 2207	11
5826	153	TGTTGACGATG	1696, 1846	11
5827	153	ACGCCGTCGG	673, 3465	10
5828	153	CATGCCCTGC	2302, 3615	10
5829	153	CCCTCACCGG	2750, 3114	10
5830	153	CCGCCGCGTC	4498, 4879	10
5831	153	CCGCGTCGCG	3421, 4516	10
5832	153	CGAACCGGGT	2151, 2277	10
5833	153	CGATCAACCC	2841, 4852	10
5834	153	CGCCGTCAGC	1176, 3149	10
5835	153	CGCCTCGAGG	2632, 3401	10
5836	153	CGCGCCGCGC	1760, 3944	10
5837	153	CGCGGCGAAC	2272, 3239	10
5838	153	CGCGGCGGTC	1359, 4714	10
5839	153	CGGCCTCGGC	1106, 2736	10
5840	153	CGGGACGGCT	2791, 4563	10
5841	153	GAAGGCGGCC	1676, 4601	10
5842	153	GACGCCGTCG	672, 3864	10
5843	153	GCCCGCGGCC	1203, 2617	10
5844	153	GCCGTGGGCC	502, 4685	10
5845	153	GCGCCCGGAC	2421, 3496	10
5846	153	GCGCGGCGGC	3553, 4660	10
5847	153	GCTGCACGCC	1170, 2610	10
5848	153	GGCCAGCAGC	3215, 3437	10
5849	153	GGCCGCCGCG	3626, 4877	10
5850	153	GGTGCGGGTG	1544, 1610	10
5851	153	GTCATCGAAT	307, 2444	10
5852	153	GTGGCCAGCA	142, 3213	10
5853	153	GTGGCGGTGA	58, 1072	10
5854	153	TCGAGCCGGT	934, 1402	10
5855	153	TGCTCGGTGA	2778, 2810	10
5856	153	TTCGACGGTG	72, 3059	10
5857	154	CCATCGCCACCGG	2440, 4393	13
5858	154	CGCGCCGGTCAGG	175, 4646	13
5859	154	CATCGCTTCGGC	3722, 3908	12
5860	154	CCGCCGAGCGTC	317, 3002	12
5861	154	CCAGGGCGTCG	591, 4337	11
5862	154	CCGCCACGATG	231, 3243	11
5863	154	CGACGTCGAGA	1134, 2374	11
5864	154	CGCCGCGCATT	835, 2045	11
5865	154	CGCCGCGGCGA	2899, 4604, 4780	11
5866	154	CGTCACCGGCG	1056, 3817	11
5867	154	CTCCGCGCCGG	172, 2354	11

5868	154	GATGCCACCGA	3169, 3866	11
5869	154	GCGCGGGCGTC	293, 806	11
5870	154	GGTCGGCGCGC	1224, 4101	11
5871	154	TCGCCGCGGCG	2413, 2898	11
5872	154	ACAGCGACAC	4615, 4884	10
5873	154	ACGTCACCGG	1754, 3816	10
5874	154	CAACGCCACG	361, 3307	10
5875	154	CACCGTCGCG	372, 2732	10
5876	154	CAGCGCCGGC	2483, 2767	10
5877	154	CATGGCGGCG	1414, 3806	10
5878	154	CCGCCGCAGG	100, 2594	10
5879	154	CCGCTGCGGC	3033, 3964	10
5880	154	CGCCGAGGCC	1713, 4385	10
5881	154	CGGCGCGGGC	804, 2198	10
5882	154	CGGGCCGTCG	2669, 2837	10
5883	154	CGTCGCCGCG	505, 3135	10
5884	154	GACCGAATCC	277, 3025	10
5885	154	GATGACGTCG	1705, 4430	10
5886	154	GCACGATGCG	2211, 4772	10
5887	154	GCGCCACGCG	705, 4726	10
5888	154	GCGCGACCAC	2536, 2802	10
5889	154	GCGTCGCCGG	938, 3068	10
5890	154	GCTCGGCGGC	6, 2395	10
5891	154	GGCGGCATCG	1164, 4515	10
5892	154	GGCGGTCACC	3574, 3713	10
5893	154	GGGCCCCGCC	2008, 4454	10
5894	154	GGGCCGCGGC	2863, 4117	10
5895	154	GTCATGGCGG	1372, 3569	10
5896	154	GTCGCCCCGA	3835, 4028	10
5897	154	GTGCTGGTGG	1991, 2568	10
5898	154	TCGAGCCCCC	1297, 4674	10
5899	155	CGAGGCCGCCGGCC	1297, 4019	14
5900	155	GGCAACAACGCCG	1313, 4676	13
5901	155	GGCGATGACCGA	1558, 4636	12
5902	155	ACGCCGAGCAC	1254, 4237	11
5903	155	AGGACGCCGAG	2576, 4234	11
5904	155	CAACATCATCG	1582, 3968	11
5905	155	CCAACGCGGTG	2912, 3733	11
5906	155	CCGGCGCCGCC	1437, 3655	11
5907	155	GCCGACGACGA	402, 4769	11
5908	155	GGGTGATGTGG	598, 4384	11
5909	155	CCCGCCGCCG	485, 2148	10
5910	155	CCGCGGGGCC	427, 1036	10
5911	155	CCGGCCGCTG	2181, 4277	10
5912	155	CCTGCTGCGC	569, 3195	10
5913	155	CCTGGCGCGC	1773, 4594	10
5914	155	CCTGGTGCGC	1357, 1486	10
5915	155	CGACGAGGGC	3959, 4873	10
5916	155	CGCAGCTGGG	56, 2777	10
5917	155	CGCCGAGCGG	1240, 2733	10
5918	155	GACCAGGCCG	1988, 2866	10
5919	155	GAGGGCATCA	1634, 3019	10
5920	155	GCACCGCGGA	1381, 3549	10
5921	155	GCAGATCGTG	614, 3177	10
5922	155	GCCCCGGTCG	3, 2346	10

5923	155	GCGACGCCGG	881, 4722	10
5924	155	GGCCAGCAGT	231, 3151	10
5925	155	GTTCCGGCGG	2388, 2418	10
5926	155	TCGCTCGCCG	414, 3495	10
5927	156	TTCGAGATCATGA	529, 2752	13
5928	156	AGGGCTACTGCT	446, 2429	12
5929	156	CGCCACCGCGGC	570, 1843	12
5930	156	CGGCGCGGCCGA	1183, 1726	12
5931	156	GGACGCACCTGG	466, 1616	12
5932	156	AGAACCTCTAC	1122, 4870	11
5933	156	AGGTCCTGGTG	803, 2180	11
5934	156	CAACGGCGCCA	2337, 3944	11
5935	156	CCCTATGACCC	850, 4647	11
5936	156	CCGGCGACGAC	2201, 3163	11
5937	156	CCGGGCCGCGA	591, 4109	11
5938	156	CGAGGGCGTGT	339, 4739	11
5939	156	CGCCGCGCGCC	2121, 3328	11
5940	156	CGCGCCCGGCG	3158, 4069	11
5941	156	CGGCCGGCAAG	964, 1629	11
5942	156	GCACTTCTACC	636, 2448	11
5943	156	GCGGACCTGGT	2224, 2470	11
5944	156	GGCCGAGGGCG	336, 402	11
5945	156	GGGATGGTGCT	739, 1874	11
5946	156	GGGCGGGACCC	484, 4204	11
5947	156	AGCGCGGCCA	124, 3540	10
5948	156	ATCGACGGCG	1178, 1274	10
5949	156	ATCGTCGGGC	478, 3453	10
5950	156	CAACGCGCCG	2106, 3929	10
5951	156	CACCGGCGGC	780, 3342	10
5952	156	CAGCATGCTC	294, 2790	10
5953	156	CCGCATTCCC	1251, 3920	10
5954	156	CCTCGGCCGG	585, 961	10
5955	156	CGCCGAGCTG	6, 3242	10
5956	156	CGCGCCGGCA	2155, 4836	10
5957	156	CGGCCGACGA	2027, 3693	10
5958	156	CGGCGCGCCG	994, 1514	10
5959	156	GATCGCCGCG	1327, 2118	10
5960	156	GATGGCCGCG	931, 1741	10
5961	156	GCACCCGGCC	1020, 3314	10
5962	156	GCCAACGGCG	607, 3942	10
5963	156	GCGACGGCCG	3689, 4271	10
5964	156	GCGGGAGTCC	1339, 1580	10
5965	156	GCTGGACCTG	3596, 4382	10
5966	156	GGACCTCGGC	582, 3737	10
5967	156	GGTGCCCAGG	2296, 2655	10
5968	156	TCACCGAGGA	815, 4312	10
5969	156	TCGGGCTGGA	3385, 4483	10
5970	156	TGTGGTGGTG	1045, 2546	10
5971	157	CCGACGACACCGT	1655, 2473	13
5972	157	TGTTGACGTTCG	4459, 4663	12
5973	157	ACGCCGACGTG	4168, 4651	11
5974	157	ACGTCGACTTC	2779, 4786	11
5975	157	CATCGGCCGCG	475, 580	11
5976	157	CGATGATGTCG	2708, 2915	11
5977	157	CTGATCGACGA	143, 502	11
5978	157	GACCGGCCGCG	541, 4245	11

5979	157	GATCCGGGCGC	3552, 4683	11
5980	157	GATGTCGATCA	1810, 2628	11
5981	157	GCCGCGACGAG	183, 1115	11
5982	157	GGCGTTCCGCG	274, 4181	11
5983	157	GTCTCGGCGAG	1643, 2833	11
5984	157	TCGGCGGCCTG	678, 1334	11
5985	157	TGATGTCGATC	2261, 2627	11
5986	157	TGCCGCGCTGG	727, 3926	11
5987	157	AAAGTCGGTG	3520, 3763	10
5988	157	ACCGAGCTGG	257, 2536	10
5989	157	CACGCCGACG	760, 4650	10
5990	157	CATGCGCGAC	1129, 3471	10
5991	157	CATGGCGGCC	1207, 3376	10
5992	157	CCGGCGTTGG	1694, 2695	10
5993	157	CCGGTGACCA	2111, 2980	10
5994	157	CGCGCCGCTG	385, 4085	10
5995	157	CGGTGTCATC	469, 997	10
5996	157	CTGACCGGCC	539, 1220	10
5997	157	CTGCCCGGCA	914, 4323	10
5998	157	GATCCTGCGG	2799, 4505	10
5999	157	GATCTGATCG	140, 3042	10
6000	157	GCCGGCCGAG	2720, 4826	10
6001	157	GCCGTGGCCA	32, 2081	10
6002	157	GCGCCGCACG	1037, 3828	10
6003	157	GCTCGACAGC	10, 3861	10
6004	157	GGCCGCGACG	1114, 2490	10
6005	157	TCTTCGCTGG	2653, 3890	10
6006	158	CCGCGCGCAGCACC	3368, 4157	14
6007	158	CGTCATCGCGCGGG	771, 2834	14
6008	158	GCGAACGGCAGCG	1346, 1877	13
6009	158	GCGACGAGACCG	476, 3093	12
6010	158	TCGATGAGCTCG	3340, 4285	12
6011	158	ACGCCGGCCTC	668, 3478	11
6012	158	GCTGGATCTCG	1667, 3151	11
6013	158	TCGCCTCGGCG	1059, 3575	11
6014	158	TGCCGACCGCG	3461, 4151	11
6015	158	ATCGCCGGCG	356, 3445	10
6016	158	ATCGCCTTGC	1255, 4048	10
6017	158	ATTGCGCTGG	1662, 4738	10
6018	158	CAGCAGCCGC	1570, 2391	10
6019	158	CCACCAGCGT	1080, 3404	10
6020	158	CCCTCGATCC	4027, 4333	10
6021	158	CCTCGAGCAT	1479, 2459	10
6022	158	CCTCGTCGTC	1026, 3638	10
6023	158	CGATCATGGT	2438, 4839	10
6024	158	CGCCGCTGTC	4367, 4454	10
6025	158	CGCGATCACG	496, 4584	10
6026	158	CGGCCAGCCG	2486, 2565	10
6027	158	CGGCCGAGCA	2068, 3138	10
6028	158	CGGCGGGCGA	566, 1648	10
6029	158	CGGTCAGCGC	975, 1506	10
6030	158	CTTGTCGATG	1815, 3336	10
6031	158	GCAGGTCGTC	1964, 4553	10
6032	158	GCCAAGGGCG	239, 1210	10
6033	158	GCCGCGCGCG	146, 1699	10

6034	158	GCGACGCTGG	86, 131	10
6035	158	GCGAGTGGTC	2316, 2642	10
6036	158	GCGCCCCCGG	1364, 3321	10
6037	158	GCGCGGTGCG	682, 2216	10
6038	158	GGCCCGGGTG	2653, 4828	10
6039	158	GGTGATGCCG	2160, 2196	10
6040	158	GGTGCCGACC	1192, 3459	10
6041	158	TGCCGTGCGG	965, 3434	10
6042	159	CGAACGCGTCGA	458, 1324	12
6043	159	CGCTGCGCAACA	263, 2995	12
6044	159	CTTCTTCCTTGG	404, 1381	12
6045	159	GTGCGGGCCGGC	345, 1909	12
6046	159	CATGAACGCCG	2324, 3686	11
6047	159	CCGGTGCGGGG	922, 3826	11
6048	159	CGCCGCCGAGG	2528, 4190	11
6049	159	CGGCCGGCCGA	2234, 4265	11
6050	159	GACACCGTGAT	2196, 2661	11
6051	159	GCGCTGCGCAA	2994, 3213	11
6052	159	GGCGGCTGGCT	1074, 3582	11
6053	159	AAGATCGCGC	2820, 4530	10
6054	159	ACCGAACAAC	252, 1797	10
6055	159	ATGATCCTGA	2061, 4554	10
6056	159	CACCCGCAGG	203, 1442	10
6057	159	CACCGCCGCG	1352, 4436	10
6058	159	CAGCGTCGAC	1547, 2072	10
6059	159	CATCGACAAC	3014, 4091	10
6060	159	CGACGAGATC	2276, 3119	10
6061	159	CGCGACGGCG	3870, 4706	10
6062	159	CGGCCACGGC	3620, 4736	10
6063	159	CTGGTGCAGG	426, 2424	10
6064	159	GCCGGTCGGC	1857, 4609	10
6065	159	GCCGGTTGAC	935, 4623	10
6066	159	GGCCGGGTGG	1532, 4212	10
6067	159	GTTCCGCGAC	2189, 2945	10
6068	159	TGATGAAGAT	605, 4525	10
6069	160	CGCAGGCCCTGGC	2699, 2885	13
6070	160	GCTGGCCGCCGGC	357, 817	13
6071	160	ACCCGCTGGGCG	1590, 1620	12
6072	160	GAGGCGTGCGCG	277, 3310	12
6073	160	GCCTGACCGACG	17, 4510	12
6074	160	ATCGATGCCGA	51, 265	11
6075	160	CCACCGCGCCC	2170, 3090	11
6076	160	CCGGATGAACC	1582, 4077	11
6077	160	CGACCGACATC	2274, 4319	11
6078	160	CGACCTGTTCG	2131, 2721	11
6079	160	CGACGGCTCGA	3035, 3864	11
6080	160	CGAGCTCATCG	3207, 3414	11
6081	160	CTCGGCGCGCG	979, 3058	11
6082	160	GATTCGGCGTG	3011, 4648	11
6083	160	GCGGCTGCTCG	3942, 4302	11
6084	160	GCTGCGCCAGC	3243, 4889	11
6085	160	GCTTCGCCGGC	2543, 3620	11
6086	160	GGTGCCCGCCA	2997, 4089	11
6087	160	GTGGCCGTCGG	82, 2973	11
6088	160	ACCGCGACGA	2325, 3431	10
6089	160	ACGACGTCAC	3281, 3404	10

6090	160	ACGCCGAGAC	2442, 3332	10
6091	160	CAGGCGGCCG	4147, 4722	10
6092	160	CCCACACCAA	386, 3482	10
6093	160	CCCGACGTCC	2614, 3982	10
6094	160	CCGCGACCGA	1458, 2271	10
6095	160	CCGCGGGTGC	2740, 2912	10
6096	160	CGATCGCGCT	374, 1605	10
6097	160	CGCCGACCTG	682, 3651	10
6098	160	CGCCTTCGTC	330, 2368	10
6099	160	CGGCACCGCG	2261, 3427	10
6100	160	CGGGAGCCGT	520, 1635	10
6101	160	CTCCCCGAGG	2225, 2420	10
6102	160	CTCGACGAGT	1103, 3577	10
6103	160	CTGGCGCGGG	2453, 4270	10
6104	160	GACGTCGGTG	1502, 2673	10
6105	160	GGACACCGCG	483, 4766	10
6106	160	GTGCAGGCGG	902, 4144	10
6107	161	GACGAATCCGACC	2358, 3284	13
6108	161	CCGCCGTCCAGG	2143, 4416	12
6109	161	CCGCCTTCGCCG	810, 2952	12
6110	161	CTCCGGGGTGAC	113, 2591	12
6111	161	GCGCGCCCAGTT	2243, 3538	12
6112	161	AGCAATCCGCC	2137, 2946	11
6113	161	CCACGTCGGCG	2198, 4519	11
6114	161	CCTCGTAGCGC	337, 616	11
6115	161	CGATCCAGCCC	2511, 4849	11
6116	161	CGCCGCCGTGG	2633, 4038	11
6117	161	CGGCCACGTCC	1664, 4510	11
6118	161	CGGCGTCGGCG	2216, 4678	11
6119	161	CGGGCCCGACG	1898, 3244	11
6120	161	CTCGGCGATGC	3125, 3478	11
6121	161	CTTCGTCATCG	236, 3207	11
6122	161	GTCACGTTCGG	3236, 3266	11
6123	161	TTGTTGAGCAG	1170, 2667	11
6124	161	AAGCTGGCCG	4361, 4388	10
6125	161	ACACCGGCCA	1017, 2499	10
6126	161	ACCGCGGCGT	708, 2026	10
6127	161	ACCTCGATGA	1119, 1696	10
6128	161	ACGACGTCGA	1071, 2038	10
6129	161	CACCGCCGGC	1956, 2773	10
6130	161	CCGCCGCGAC	1643, 1724	10
6131	161	CCGGCCGCGA	1996, 2308	10
6132	161	CGACCGCCCC	637, 2854	10
6133	161	CGACGGCCTC	2758, 4315	10
6134	161	CGCCAGGTCG	1259, 3871	10
6135	161	CTCGATGTCG	467, 782	10
6136	161	GACGATGACG	4114, 4694	10
6137	161	GCGCCGCGCG	3755, 3827	10
6138	161	GGCGCGCCCA	3094, 3537	10
6139	161	GGCGGTGGTG	2091, 4027	10
6140	161	GTGTCGATCC	975, 2111	10
6141	161	TCGGCGTTGG	483, 4824	10
6142	161	TGGCCGCGGC	514, 3019	10
6143	162	CGCGACGGCGAGC	163, 1345	13
6144	162	CGTCGGCGCCGGC	1672, 4332	13

6145	162	GGCGAGCGGGCCG	3724, 4093	13
6146	162	ACCGGCTGGCCG	2597, 3251	12
6147	162	CGAGGTGCTCGG	2317, 3906	12
6148	162	GGCACACCCTCG	2449, 2567	12
6149	162	ACAAGGCGGCG	2828, 4407	11
6150	162	ATCGCGCCGGG	299, 2976	11
6151	162	CGACGTCGCCG	2718, 4422	11
6152	162	CGATCACGTAC	699, 3023	11
6153	162	CGTCGACGTCG	3612, 4419	11
6154	162	CTACCTCGACG	3333, 3570	11
6155	162	GCCACCGGCGA	2482, 4219	11
6156	162	GGACGTCGGCG	1669, 3972	11
6157	162	GGCGATCGCCG	355, 1306	11
6158	162	ACGGTCAGCG	380, 2201	10
6159	162	CAGGGCGGTG	259, 2021	10
6160	162	CCCGAGGTCC	890, 2470	10
6161	162	CGAGGGTCGC	759, 3192	10
6162	162	CGCCATCCAC	4570, 4619	10
6163	162	CGCCGGACCC	2924, 4114	10
6164	162	CGGCGCCGGA	543, 4111	10
6165	162	CGGCGGGATC	630, 2493	10
6166	162	GATGGGCGAC	418, 3561	10
6167	162	GCCGCGCGCC	1601, 4436	10
6168	162	GCGCGGGCGG	825, 3241	10
6169	162	GCGGTCGACC	1730, 2686	10
6170	162	GGCCGGACGT	482, 2295	10
6171	163	TTCCAGGATCGCG	2612, 4728	13
6172	163	CGGCCCAGCGCT	86, 4547	12
6173	163	GCGTCCTCGGGC	2706, 3662	12
6174	163	GTCGGAGCCGGC	21, 4743	12
6175	163	GTTGCGGCCGCC	228, 4456	12
6176	163	TCTCGCGCACCG	1832, 4562	12
6177	163	ACGGGGCAGTC	590, 1096	11
6178	163	CCCCGCGCCCG	917, 2338	11
6179	163	CGCCGCCGCGA	2244, 2800	11
6180	163	GAAGGTGCCGT	3071, 3728	11
6181	163	GACCGCGGTGG	2192, 3548	11
6182	163	GACGGCCGCGG	1530, 4498	11
6183	163	GCGCCGCCGCG	2243, 2284	11
6184	163	GCGCGGTCTCG	1622, 3640	11
6185	163	GCGTTGCGGCC	226, 1691	11
6186	163	GGCCCGCAGAA	69, 450	11
6187	163	GGGCGTCGTAG	4202, 4859	11
6188	163	TCCAGCGAGAA	680, 3176	11
6189	163	ACCGGCCGCC	996, 3373	10
6190	163	AGGGGCGCTC	332, 785	10
6191	163	ATGCCGGTGC	2235, 3015	10
6192	163	CCTCCAGCGA	2761, 3174	10
6193	163	CGCTTCGGGA	2473, 4695	10
6194	163	CGGAACGCCG	1251, 1381	10
6195	163	CGGCCAGGTC	14, 1967	10
6196	163	CGGTCTTGTC	59, 4103	10
6197	163	CGTTCTCGCG	1052, 1829	10
6198	163	CTCCTCGATG	2969, 4584	10
6199	163	CTCGGGGTAG	3125, 4113	10
6200	163	GAGCACCCGG	2011, 3897	10

6201	163	GATAGCCGCC	1943, 3313	10
6202	163	GCAGCAGCCA	260, 1739	10
6203	163	GCCATCGCCG	646, 1009	10
6204	163	GGACAGCAGC	120, 1995	10
6205	163	GGCCTCCACC	351, 1899	10
6206	163	GGCGCGGTCT	2678, 3639	10
6207	163	GGCGGCCAGG	1965, 4332	10
6208	163	GGTCGGCCGG	1244, 1449	10
6209	163	GTGGTTGCGG	970, 2035	10
6210	163	TCGACGCCGG	1760, 2853	10
6211	163	TGGCCTGCCA	2064, 4655	10
6212	163	TGGTGGTTGC	2033, 3862	10
6213	164	CAGGATCGCGCG	1766, 4064	12
6214	164	GTCGACCGCGGC	726, 2492	12
6215	164	TGCCGGTCCGGC	970, 1078	12
6216	164	CGAATTCGCCG	2008, 4695	11
6217	164	CGCGCCACCGG	4158, 4733	11
6218	164	TCGTCGCGCCC	1801, 4280	11
6219	164	TGTGCGCGGCG	701, 941	11
6220	164	ACACCCCGGG	2227, 3676	10
6221	164	ATCACGCCCG	1836, 2193	10
6222	164	ATCCGCATCG	2127, 3485	10
6223	164	CGATCCGCGT	1481, 4051	10
6224	164	CGCCGCGCGC	1497, 4153	10
6225	164	CGGCGCGATC	2263, 4129	10
6226	164	GAACCGCTCG	561, 888	10
6227	164	GAGCCCGGCC	1850, 3689	10
6228	164	GATCAGCGGT	1233, 3299	10
6229	164	GCCGGCCGAG	1972, 3416	10
6230	164	GCTCGCGTCG	1224, 3856	10
6231	164	GGCCGGCCGA	478, 1971	10
6232	164	GGCCGGTGGC	1732, 2206	10
6233	164	GGCGGCGCCG	525, 3635	10
6234	164	GGTAGTTCCA	2934, 3817	10
6235	164	GTCGATGATC	3167, 4792	10
6236	164	GTCTTTCTCG	152, 4510	10
6237	164	TCGGCGAGCA	2109, 3258	10
6238	164	TGCAGCGCGC	952, 4625	10
6239	164	TGCGATCGCC	3074, 4443	10
6240	164	TGCGCGGCAG	1759, 2656	10
6241	165	CGAGGAGATCGAG	570, 3751	13
6242	165	CATCTGCAGCGA	2289, 4207	12
6243	165	CTCGAGGAGATC	568, 4144	12
6244	165	GCTGCTCGTCAC	894, 1877	12
6245	165	CACCTTGATCG	1595, 4189	11
6246	165	CCAAGGAGCAC	554, 2613	11
6247	165	CGGGTGTCGCG	473, 515	11
6248	165	CGGTCGCCGAT	249, 3974	11
6249	165	GACGGGGTCGC	299, 826	11
6250	165	GCGCAGCGGCT	412, 1063	11
6251	165	GGGGCCGACGA	2663, 3868	11
6252	165	TCCTGCGCGGC	25, 1099	11
6253	165	TGCCCCGTCGG	1397, 4446	11
6254	165	AATCCGCGGT	243, 2120	10
6255	165	ATGGTCGATT	3200, 4321	10

6256	165	CAATTCGCCG	2106, 3161	10
6257	165	CATGGTTGCC	129, 1658	10
6258	165	CGATGGTCGA	3198, 3270	10
6259	165	CGCCGAAGGC	2860, 3469	10
6260	165	CGGATGGTGC	1390, 4420	10
6261	165	GAGGACCGCA	3131, 4798	10
6262	165	GCTTCCGTGA	2279, 2456	10
6263	165	GGCGAGGCGA	4027, 4690	10
6264	165	TCGACCCGAA	3510, 4568	10
6265	166	AGCGTGAAGCCACGG	3602, 3622	15
6266	166	TGCGCGATGCCGTC	1020, 1354	14
6267	166	CGCCGACGGGCT	1156, 3285	12
6268	166	GAGGCGGCGCGG	638, 1296	12
6269	166	CAAGGCCGCCG	727, 2003	11
6270	166	CATCGCCGGCG	452, 3393	11
6271	166	CCGCGCGGTGC	2721, 4454	11
6272	166	CGCCGTTCGCT	251, 1109	11
6273	166	CGCGACCCTGA	616, 4575	11
6274	166	GCCATGTGCTC	1675, 3568	11
6275	166	TCGGCGCCTGC	972, 1906	11
6276	166	TTTGCGGACCG	3055, 4340	11
6277	166	ATCGTTCTCG	1500, 4030	10
6278	166	CAACGGCTGG	36, 2448	10
6279	166	CACCGCCCGT	1835, 3183	10
6280	166	CAGCAGTTCG	1959, 2509	10
6281	166	CAGCCACCGG	2470, 3640	10
6282	166	CAGTTCGCGC	1560, 2205	10
6283	166	CCACGAGCGC	1856, 3027	10
6284	166	CCCGTCCTGC	1785, 2641	10
6285	166	CCGCCGACGG	783, 1155	10
6286	166	CGACCGCCCG	570, 4247	10
6287	166	CGACGTGCGG	2952, 3799	10
6288	166	CGGCCCGCCG	779, 1759	10
6289	166	CTTCACCGAC	592, 2946	10
6290	166	GACGGCGATC	1686, 2734	10
6291	166	GAGCGGCCCG	183, 4431	10
6292	166	GATCGGCACC	3911, 4514	10
6293	166	GCGCAGCGTG	1394, 3598	10
6294	166	TCCAGGTGAT	109, 747	10
6295	166	TGAGCGTGAA	3620, 3893	10
6296	166	TGCTGGGCGG	909, 2242	10
6297	166	TGGCCAAGGC	723, 2110	10
6298	166	TGGTGGCCAA	720, 3005	10
6299	167	TCCTCGGCGGCCTT	1365, 1380, 1395	14
6300	167	GTCGATCACCCGC	989, 4257	13
6301	167	CGTCGGTGTCGG	1312, 1348	12
6302	167	GCCCTGGCATCG	1536, 3744	12
6303	167	TCCGTCCAGGCC	2156, 3396	12
6304	167	CCGCGACGAAG	1066, 1138	11
6305	167	CGACGTCGTCG	1324, 3517	11
6306	167	CGGCGTTGACC	4046, 4322	11
6307	167	CGGCTTCCTCG	1519, 3887	11
6308	167	CGTCGGCGGTG	683, 4403	11
6309	167	TCTTCGGTGAT	3429, 3586	11
6310	167	TGGGGCACTTG	642, 3409	11
6311	167	ATCCGCTGGC	1083, 1129	10

6312	167	CACCAGCACG	201, 270	10
6313	167	CATTGACGAC	497, 3511	10
6314	167	CCGGGTGCCG	2582, 2628	10
6315	167	CGCCGGAGCC	357, 1446	10
6316	167	CGGCCGGGCG	2207, 2283	10
6317	167	CGGCGGGCAC	3052, 3653	10
6318	167	CGTCGACGAA	3076, 3817	10
6319	167	CTCGGGTGTG	2966, 3930	10
6320	167	CTCGTCGGCG	681, 1292	10
6321	167	CTGCTCGACG	211, 920	10
6322	167	GACCATGTCG	2906, 4203	10
6323	167	GCCGCCCTCG	657, 3095	10
6324	167	GCCGCGGATT	95, 2872	10
6325	167	GCCGTCGATG	2831, 3687	10
6326	167	GCCGTTGACC	180, 4101	10
6327	167	GCCTCGTCGG	1290, 1308	10
6328	167	GGCGCGGATC	2882, 4356	10
6329	167	GTCGTGGAAC	965, 2513	10
6330	167	TCCGCCACCG	1672, 4509	10
6331	167	TGACGATGCG	114, 4178	10
6332	167	TGATCGACCA	259, 1917	10
6333	168	GCAAGACGTTCGC	51, 1419	13
6334	168	GGCGGGCGCGGTG	1237, 1524	13
6335	168	GTCGCGCTGACCA	650, 2116	13
6336	168	ATCGGCGGCACG	2940, 4085	12
6337	168	CCGCTGGTCACC	551, 3063	12
6338	168	CCTGCAAGCCGT	2454, 4537	12
6339	168	CGTCGCCGCCGG	40, 1039	12
6340	168	GGGCGCTGGCCG	1269, 3987	12
6341	168	TCGATCTACGGC	1223, 4694	12
6342	168	TGCTCGCGGTGA	1167, 4386	12
6343	168	ATCGGCATCGT	320, 3550	11
6344	168	CGCCGGGATGC	1941, 4378	11
6345	168	CGGCCAAGTAG	3839, 3850	11
6346	168	GACCGAGGTCG	700, 2873	11
6347	168	GGCCGGCGCCG	2652, 3381	11
6348	168	GGCGCTGACCG	4183, 4555	11
6349	168	GGTCGGCGCCA	271, 442	11
6350	168	GTCGAGCACGC	230, 1567	11
6351	168	ACCGGGCGCT	219, 1266	10
6352	168	ACTTCGCCAA	804, 933	10
6353	168	AGCGGCTCGA	1074, 2141	10
6354	168	CATCGGCATC	3549, 3822	10
6355	168	CATGGTGACC	838, 3360	10
6356	168	CCCCGCCCGG	1964, 2156	10
6357	168	CGACGGCCGC	28, 2214	10
6358	168	CGCCATCGGC	2936, 3819	10
6359	168	CGCCGGCGTG	604, 4270	10
6360	168	CGCGGCGGCG	139, 1861	10
6361	168	CGCGTTCGAC	154, 1180	10
6362	168	CGGCGACCCG	952, 1584	10
6363	168	GACGTCGACG	671, 1795	10
6364	168	GATCAACGAA	991, 4303	10
6365	168	GCAGCGCCTG	3477, 4805	10
6366	168	GCCGCGAGAT	1377, 4026	10

6367	168	GCGCTTTTCG	2928, 3673	10
6368	168	GCGGCAAGCG	1068, 1667	10
6369	168	GCGGTGCGCA	1202, 4640	10
6370	168	GGGCGCCGGT	2435, 2959	10
6371	168	GGGCGGCAAG	766, 1066	10
6372	168	GGGGGCGGGC	1453, 4063	10
6373	168	GGTGGCTACG	1882, 3562	10
6374	168	TCTGGTCAGC	3261, 4357	10
6375	168	TTCAAGACGT	2810, 3587	10
6376	168	TTCGGCGCCG	1331, 4265	10
6377	169	GCCGACATCGGC	685, 994	12
6378	169	GGCAGCGGCACG	2489, 4697	12
6379	169	CCATCGCCGGT	3042, 3167	11
6380	169	CGCGGCGCTGA	2602, 4002	11
6381	169	CGGCAGCGGCA	2488, 3858	11
6382	169	CGGCGCCACCA	2030, 4622	11
6383	169	GTCGGCACGGT	1308, 2339	11
6384	169	TCAACACGGTG	353, 1726	11
6385	169	TCGTCGACATC	227, 3872	11
6386	169	ACGACGTCGT	1662, 4882	10
6387	169	CCGGGCGCGG	4582, 4788	10
6388	169	CGACGCTGGA	2637, 2934	10
6389	169	CGACGTCACG	2752, 4098	10
6390	169	CGCCTCGAAC	600, 3561	10
6391	169	CGGCGACGGG	923, 4744	10
6392	169	CGTCGGCCGG	3753, 3837	10
6393	169	CTGCTTGCCG	1881, 3334	10
6394	169	GACGCGGCGC	574, 4000	10
6395	169	GATCGTCGAC	630, 2851	10
6396	169	GCAGCTGTTG	1032, 1062	10
6397	169	GCCGGGGGCC	2291, 4808	10
6398	169	GTCGCCTCGA	136, 3559	10
6399	169	TCAACGACGT	1252, 4879	10
6400	169	TCGCTGGCGG	1869, 2999	10
6401	169	TGGCGCCGAC	749, 2928	10
6402	170	ACGCCACCGCCG	1028, 2564	12
6403	170	CGTCGACCGAGC	213, 706	12
6404	170	CTGCTCGGCATC	1172, 4677	12
6405	170	CATCCCGGCTT	1827, 2515	11
6406	170	CCGCGGTGGGT	1278, 1377	11
6407	170	CGCCGACGTCG	31, 637	11
6408	170	CGCGCAGCTTC	1734, 4724	11
6409	170	CGCGCGACGCC	1629, 4006	11
6410	170	CGGTGCCCGCA	2161, 2583	11
6411	170	AACGCCACCG	1027, 3981	10
6412	170	AAGCATCCCG	1522, 1824	10
6413	170	ACCGTCGGCT	2048, 3195	10
6414	170	ACGTCGTCGC	3963, 4816	10
6415	170	AGCGCGACGT	54, 444	10
6416	170	ATCGCGGTTC	2150, 3805	10
6417	170	CCGGTGCCCG	830, 2582	10
6418	170	CGCTGAGCCG	189, 4184	10
6419	170	CGCTGGATCA	528, 2607	10
6420	170	CTCGCGGCGA	2394, 4543	10
6421	170	CTGGGCTCAC	1595, 3505	10
6422	170	GCACCGCTGG	2603, 2919	10

6423	170	GCCGTCGTCG	2467, 4161	10
6424	170	GGCCGCTACG	1969, 4809	10
6425	170	GGCGCCACCA	176, 3728	10
6426	170	GGCGCCGATG	997, 2488	10
6427	170	TCGATCGAGC	1919, 4399	10
6428	170	TCGGCCCGGT	2352, 3475	10
6429	171	CGACGGCGTCGGCG	1079, 3815	14
6430	171	ACGGCCGCCTTCG	66, 3207	13
6431	171	GGTTGCCGCGTT	240, 1513	12
6432	171	GTTCCTGATCGG	1816, 2625	12
6433	171	CGTGGGTGGCG	1121, 3738	11
6434	171	CTGGTCGGCGC	2102, 3749	11
6435	171	GGTGCGGAACT	1041, 4566	11
6436	171	GTGCGGTGATC	1299, 2117	11
6437	171	GTGGTGGCGGC	265, 1913	11
6438	171	TCGGCGTCATC	2372, 3364	11
6439	171	TGTTCCTGATC	563, 2624	11
6440	171	AACTCGTCGA	3430, 4678	10
6441	171	CGATCCGGCC	767, 1531	10
6442	171	CGGCGCTGAC	1758, 3564	10
6443	171	CGTGCTGTTC	1216, 2987	10
6444	171	CGTTGATTTC	3464, 3923	10
6445	171	GACGCCGATC	1648, 4448	10
6446	171	GATCCTGCTC	1408, 2658	10
6447	171	GCAGGGCGGC	3388, 4298	10
6448	171	GCCGCCTCGA	760, 973	10
6449	171	GCGCGCCGTG	353, 2951	10
6450	171	GCGGGCCAGG	3984, 4173	10
6451	171	GCTCAGCGCG	738, 2490	10
6452	171	GCTGCTCAAC	1771, 2514	10
6453	171	GGCGTCGAGC	1146, 4384	10
6454	171	TCGCGCTCGG	1006, 3358	10
6455	171	TGATGCCGAC	1073, 4502	10
6456	172	AATCGAGCCCCGGTCGGCGACGATGCAGAGCGCGCAGCG CGATGAGGAGGAGCTGACC	4120, 4178	58
6457	172	ACGATGCAGAGCGCGCAGCGCGATGAGGAGGAGCCGGCC AATCGAGCCCCGGTCGGCG	4023, 4081	58
6458	172	GTCAGCGGCACCG	2644, 4666	13
6459	172	AAGGCGGGGCTG	514, 4846	12
6460	172	GAGGTCTTCCGC	577, 3743	12
6461	172	GGCGGCGACGGC	1862, 2596	12
6462	172	TTGAGCGCGGCG	1905, 4273	12
6463	172	CCCGCCGCCGC	2079, 2689	11
6464	172	CGACCCGGTCG	3229, 3778	11
6465	172	CGAGGTCGTCG	1334, 3016	11
6466	172	CGATCGTCGAG	3339, 3690	11
6467	172	CGTTCTTCGAG	3123, 3819	11
6468	172	GCGCGACGATG	4, 4877	11
6469	172	TCTCCGGCGCC	2046, 2495	11
6470	172	AGCCGGCGAC	2531, 4518	10
6471	172	CCGAGGAAGC	596, 4511	10
6472	172	CGCGCCATCC	3110, 3392	10
6473	172	CGGCATCGAC	3832, 4389	10
6474	172	CTGGGCGCCC	2072, 4419	10
6475	172	GCCGCGGTGA	252, 484	10

6476	172	GGAGTGGGCG	3367, 4767	10
6477	172	GGCATGCACT	2361, 3506	10
6478	172	GGTCGCGATC	3685, 4410	10
6479	172	GTCGTCGACG	2512, 4309	10
6480	172	GTCGTCGAGC	3482, 4345	10
6481	172	GTTCCCGAGC	1722, 3769	10
6482	172	TGCCTTGCTC	1745, 2349	10
6483	172	TGCGGATCTG	138, 4782	10
6484	173	GCTGCTGGCGGACC	3664, 3867	14
6485	173	ACACCGTGCGGC	1967, 2062	12
6486	173	CCGGCGGCGACG	2219, 4834	12
6487	173	GGCTGGTGCCGC	660, 2690	12
6488	173	GGGCCGCCGACG	60, 2956	12
6489	173	CGCGACCGACG	4423, 4719	11
6490	173	CGGCGGTGACC	127, 2417	11
6491	173	CGTCCCCGATC	814, 1952	11
6492	173	GACCAGCAGGT	1638, 2607	11
6493	173	GGACGGGCCGC	2952, 4146	11
6494	173	GGCGTCGACCA	1465, 4768	11
6495	173	TCCCCGACGAA	583, 1254	11
6496	173	TCGAGCTGCTG	966, 3660	11
6497	173	TCGGTCGCGGC	2653, 3259	11
6498	173	TTTCGACGACG	443, 1216	11
6499	173	ACCGAGGCCG	222, 1445	10
6500	173	ACCTCGACGA	370, 1675	10
6501	173	ATCGCGTCGG	797, 1115	10
6502	173	CACCGCCGGC	281, 2457	10
6503	173	CAGGTGGTCG	1761, 3536	10
6504	173	CATCGTCGGC	467, 494	10
6505	173	CCGGGGCCGC	57, 3847	10
6506	173	CGACGGCGGC	1991, 4449	10
6507	173	CGACGGTGTA	1856, 3242	10
6508	173	GACGCCGAAT	1570, 3033	10
6509	173	GACGCGGTCC	2242, 4740	10
6510	173	GATGATGTGG	3147, 4389	10
6511	173	GCAGGACGGG	3607, 4143	10
6512	173	GCCCTGCGCG	2937, 3746	10
6513	173	GCGCGGCGCC	1943, 2285	10
6514	173	GGAGCGGGCA	1319, 1499	10
6515	173	GGATGCGCGA	1984, 4607	10
6516	173	GGCGATCGAC	398, 1162	10
6517	173	GGCGCGCACG	4158, 4659	10
6518	173	TCGTCGAGCT	963, 1726	10
6519	174	TCGGCCTGGTCGACGAGCTC	2472, 4141	20
6520	174	CATCACCGTCGAACCG	354, 868	16
6521	174	CCGTCATGGTCGTCG	629, 2168	15
6522	174	TCATGGAGGCGCT	83, 597	13
6523	174	CCGACGGCATCG	3625, 4525	12
6524	174	CGCGGGCCCGCA	912, 3219	12
6525	174	CGGCGCTGTCGT	1181, 1235	12
6526	174	GGCGATGGCCTC	1878, 2022	12
6527	174	TCAACGGCGGCG	3423, 4504	12
6528	174	ACACGGCCAGC	802, 4405	11
6529	174	CACGCTCGAGG	2248, 3013	11
6530	174	CATCAACGGCG	3914, 4502	11
6531	174	CCAGCGGCACG	1532, 1760	11

6532	174	CCGCGCTGGAG	939, 1484	11
6533	174	CCTGGGCAAGC	3025, 3890	11
6534	174	CGACGACGTCG	2866, 3076	11
6535	174	CGAGGCGTTCG	3355, 3836	11
6536	174	CGCCGCGGGCC	3216, 4354	11
6537	174	CGCGGCGCTGT	518, 1179	11
6538	174	CGGCCAAGGCC	4123, 4606	11
6539	174	GCAGAAGGCCG	1119, 2899	11
6540	174	GCGCAGCCAGC	2108, 2890	11
6541	174	GCTCAACGAGG	2440, 3349	11
6542	174	GGCGCGCAGGT	1308, 4386	11
6543	174	GGTGATCACCA	1584, 3755	11
6544	174	GGTGCTGGCCG	2593, 2713	11
6545	174	TCGCCACCATC	2841, 3561	11
6546	174	TGCGCCTCCGG	580, 2648	11
6547	174	AAGCCGGCCA	1525, 4119	10
6548	174	ATCGCCACCA	1792, 2840	10
6549	174	ATCTCGGCGA	1615, 2125	10
6550	174	CAAGACCACC	186, 4712	10
6551	174	CCCGAGACCA	2612, 2786	10
6552	174	CGCCGACCTG	2830, 4772	10
6553	174	CGCCGCCGAC	1376, 3974	10
6554	174	CGCGCGGGCC	910, 3311	10
6555	174	CGCGCTGGCC	3950, 4283	10
6556	174	CGGCCGGCCC	168, 1091	10
6557	174	CGTCGAGTCG	2728, 3851	10
6558	174	CTGGGCGGCG	1956, 3930	10
6559	174	CTGGGTCTGC	252, 3999	10
6560	174	CTGGTCACCG	120, 4443	10
6561	174	CTGGTGCTGG	2711, 3170	10
6562	174	GATCACCGGC	3475, 3743	10
6563	174	GCCCGGCGGC	1271, 4259	10
6564	174	GCGCCCCGAC	3001, 4520	10
6565	174	GGCCGCGCTG	1482, 1638	10
6566	174	GGGCCTGCTG	2298, 4022	10
6567	174	GGTCATCATG	592, 3262	10
6568	174	GTCGAACACC	1554, 4886	10
6569	174	GTCGGCGTCC	103, 1725	10
6570	174	TACCACTGGG	749, 3098	10
6571	174	TCGTGATCGA	570, 4795	10
6572	175	CCGGCATCGCCGA	2564, 2815	13
6573	175	TGGCCCGCGCCCG	1266, 3431	13
6574	175	CCGCGGCGCGGC	1940, 2016	12
6575	175	GGCGAGCGGTTC	800, 3954	12
6576	175	GTCGACAAGATG	74, 434	12
6577	175	GTGGCCCGCGCC	767, 3430	12
6578	175	AAGTCGACGGC	1235, 3544	11
6579	175	CCACCCGCGCG	3032, 4174	11
6580	175	CGCTGATCGAC	3940, 4081	11
6581	175	GCCGTCGGCGC	2724, 3219	11
6582	175	GGACCTGGTGC	3914, 4049	11
6583	175	GGCCCTGGCCC	127, 1874	11
6584	175	GGCCGCGGCGC	2014, 3981	11
6585	175	GGCGGCGGCGA	1554, 4872	11
6586	175	GTCGCTCATGT	1141, 4511	11

6587	175	TGACGTCGAAG	3236, 3599	11
6588	175	ACCCGGCGCC	306, 1993	10
6589	175	AGCTGGGTGA	1211, 2866	10
6590	175	CAGGCCGGCT	296, 3171	10
6591	175	CGACGGCAAG	4772, 4790	10
6592	175	CGAGGGCGTG	256, 631	10
6593	175	CGCCGGCCCG	2796, 3107	10
6594	175	CGCGGCCACC	367, 4529	10
6595	175	CGCGGGCGGC	394, 3332	10
6596	175	CGCTGGCGAT	153, 240	10
6597	175	CGGCGGCGGC	4658, 4871	10
6598	175	CGTAGTCGGG	2486, 2996	10
6599	175	CGTCGCGCAT	3365, 4540	10
6600	175	CGTGATCGGC	214, 1625	10
6601	175	CTCGCCGGTC	67, 899	10
6602	175	CTGCAGGACA	3846, 4839	10
6603	175	GACGCCGACG	3084, 4154	10
6604	175	GCCTGCAGGA	528, 3844	10
6605	175	GGCGTGGTCG	1024, 4505	10
6606	175	GTGGCCGCGA	2053, 4608	10
6607	175	GTTGTCGACG	1165, 4073	10
6608	175	TCCGGTGGCC	3474, 4632	10
6609	175	TGCAGTTCGT	2689, 4219	10
6610	176	AGGCCGCCTACGA	348, 3584	13
6611	176	ACATGGCCGAGA	3446, 3671	12
6612	176	CCTCGCCCGCGC	2007, 3359	12
6613	176	CGAAGGCCGCCT	2655, 3581	12
6614	176	GCGCCCAGCTCG	4367, 4379	12
6615	176	GCGGGCTGATCG	1910, 3770	12
6616	176	GGCGAGGATCGC	604, 691	12
6617	176	CGCCGCCGTCG	1810, 4146	11
6618	176	CGGCGCCGAGG	441, 3654	11
6619	176	GCCACCGCGGC	3037, 4232	11
6620	176	GGAGAACACGT	820, 4645	11
6621	176	TCGGCCAGGAA	2696, 4067	11
6622	176	TTGCGCTCACC	1176, 4880	11
6623	176	ACGTCAACGC	129, 1566	10
6624	176	CAACGACGTC	1561, 2808	10
6625	176	CACCGACGAC	2733, 3141	10
6626	176	CAGCGCCCGG	1384, 2146	10
6627	176	CCCGCCTGGT	1577, 2984	10
6628	176	CCCGGCCAAG	1490, 2679	10
6629	176	CCGAGATCTC	307, 2390	10
6630	176	CGCCGGCGCG	1903, 4576	10
6631	176	CGCGGCGAGG	601, 3844	10
6632	176	CGGTGCGGAC	462, 2109	10
6633	176	GAACGCGCGG	2020, 2128	10
6634	176	GACCTCGTCG	2179, 4759	10
6635	176	GCCCTCGCCC	2005, 2260	10
6636	176	GCCGCCGATC	1735, 1775	10
6637	176	GCGCCGCTGG	1088, 3541	10
6638	176	GCGGCGCCGA	440, 848	10
6639	176	GCGTTCGCCC	47, 1700	10
6640	176	GCTGCGCATC	1069, 1543	10
6641	176	GCTGCGGCAG	1673, 3810	10
6642	176	GGCCAACAAG	3225, 3279	10

6643	176	GGCGACCATC	1801, 3927	10
6644	176	GTCCAGCTCG	2629, 2890	10
6645	176	TCCGCCGGCG	838, 4574	10
6646	176	TTGCTCGACG	1004, 1394	10
6647	177	GGGCCTGCCCAAGG	3590, 4222	14
6648	177	CGGGTGCGCTGTC	3290, 3451	13
6649	177	GACCCGGCCGCGC	315, 3843	13
6650	177	GTCGAGGTCGTCG	2607, 2889	13
6651	177	CCGATCAGCGCC	1996, 2800	12
6652	177	CCTTCGGCGAGC	1777, 3305	12
6653	177	CGCCGTCGCCGC	3530, 4626	12
6654	177	GCTCGGGTGCGC	635, 3448	12
6655	177	TGCGCAGCACCG	1067, 3751	12
6656	177	CAGCGCGGCGA	1230, 2067	11
6657	177	CAGCGCGTCGA	549, 2859	11
6658	177	CCAGCAGCGCC	1334, 1718	11
6659	177	CGACACCACCG	828, 2376	11
6660	177	CGACGTCGTCG	1406, 2354	11
6661	177	CGATCGCGGCC	668, 1688	11
6662	177	CGGTGCGGCGG	1050, 2277	11
6663	177	GCCGGATCGAC	1024, 1511	11
6664	177	GCGGCCGTCGC	2341, 4684	11
6665	177	GTCCAGCGGCG	1923, 4611	11
6666	177	TGGGGCACCGG	1435, 2506	11
6667	177	TTGCGCAGCAC	394, 1066	11
6668	177	ACCAGCAGCG	508, 2854	10
6669	177	AGTCGCGCAG	206, 860	10
6670	177	ATGCCGAGCA	1903, 3099	10
6671	177	CACCTCGAGC	705, 2589	10
6672	177	CCACGCGCTC	3145, 4357	10
6673	177	CCCGGTCACC	2211, 3357	10
6674	177	CCGGCGCCGC	136, 2534	10
6675	177	CCGGCGGCCG	2119, 2338	10
6676	177	CCTGGGCCAG	227, 1328	10
6677	177	CGACCACGCG	3255, 4354	10
6678	177	CGCAGCGCGG	1228, 2716	10
6679	177	CGCCGTGCCC	1545, 4853	10
6680	177	CGTAGCCAGC	263, 4825	10
6681	177	CTCCGGCGGC	2336, 4120	10
6682	177	CTCGGCGTCG	2466, 2637	10
6683	177	GACGTCGACG	1173, 4100	10
6684	177	GACTCGTCGA	2200, 4733	10
6685	177	GATCGATGAC	3857, 4090	10
6686	177	GGAAGCACCA	148, 4769	10
6687	177	GGCCGCCGGA	1020, 1203	10
6688	177	GGCGTCGGGA	1977, 3431	10
6689	177	GGGCGGCGCG	992, 3119	10
6690	177	GGTGGCCACG	939, 1566	10
6691	177	GTGATGCGGT	613, 841	10
6692	177	TCCTCGAGCA	406, 2656	10
6693	177	TCGCCGACGG	4050, 4784	10
6694	178	CGGCCAGCACGTC	1891, 2098	13
6695	178	CCAGATGATGTT	2809, 3001	12
6696	178	CGCTCAACGCCG	1909, 4554	12
6697	178	GCCGTGCTCGAG	1982, 4730	12

6698	178	GCGGCATCAGCG	2682, 4377	12
6699	178	GCGGCCGCCTGG	704, 1451	12
6700	178	GGAGCGGGCGGC	251, 341	12
6701	178	GGCGGGCGGGGC	202, 4859	12
6702	178	TTGAAGATCGCG	4535, 4613	12
6703	178	ACGGCGACCCC	1316, 4250	11
6704	178	CCAGCAACGGC	2308, 2547	11
6705	178	CCAGCTCGCCG	1395, 2383	11
6706	178	CCAGGCCGGCC	420, 2068	11
6707	178	GCGCGGCCAGC	1377, 1888	11
6708	178	GGCGTCCTGCG	355, 3120	11
6709	178	GTCCAGCGAGA	2162, 4810	11
6710	178	ACCGATCGCG	1535, 2776	10
6711	178	ACGTCCGCTC	805, 3272	10
6712	178	CCGATCACGG	952, 2117	10
6713	178	CCGCCGAACA	2987, 3428	10
6714	178	CCGGCCGGCG	888, 1791	10
6715	178	CGAAGGGCGC	4363, 4762	10
6716	178	CGACGATCGT	2736, 2856	10
6717	178	CGCGAAAACC	1143, 4502	10
6718	178	GAAAACGGCG	1256, 4463	10
6719	178	GAGCTCGACG	3586, 4283	10
6720	178	GATCCCCGCG	1842, 4576	10
6721	178	GCCGCCACCG	574, 733	10
6722	178	GCCGGGGCGC	380, 1414	10
6723	178	GCGCAGGTCA	1172, 3710	10
6724	178	GGCGACGGCT	32, 3625	10
6725	178	GGCGGCGGCC	700, 2084	10
6726	178	GGGGGAGCGG	230, 248	10
6727	178	GTCCAGGCCC	2183, 2423	10
6728	178	TCGGCTCCGA	4221, 4515	10
6729	178	TCTCGTCACG	836, 1099	10
6730	178	TGCCGACCGG	141, 3194	10
6731	178	TGGTAGCCGT	623, 1977	10
6732	178	TGGTCTCGTC	1096, 1302	10
6733	179	CCCACCCCCTACCGCCGAGCGTGGCCCACCCC	1771, 1867	32
6734	179	CGACGCTGGGCGCGGAGGGTG	2918, 2940	21
6735	179	CGCCCCCGGCGATGG	4356, 4371	15
6736	179	AGCGCGACGCGGC	4619, 4712	13
6737	179	GTTGTGGCGGGAA	1915, 2880	13
6738	179	AACGCCGTGCGC	2466, 4589	12
6739	179	ATCGGTGCCGAG	397, 2764	12
6740	179	AGGCCTACGAG	479, 1319	11
6741	179	CACCGAGGCCG	1413, 3874	11
6742	179	CCACCCGCATC	821, 1622	11
6743	179	CCGCTGCGCCG	2111, 3993	11
6744	179	CGGGTCCACCG	3062, 3119	11
6745	179	CTGCACCAGGC	2333, 3203	11
6746	179	GAAGTTGCCGA	3964, 3976	11
6747	179	GACGCCCCCGG	1713, 4354	11
6748	179	GTGACGGCTAC	845, 4292	11
6749	179	TGGCCAGCGCG	4509, 4764	11
6750	179	AACGACTTCG	1144, 4487	10
6751	179	CCACGGCGCC	3215, 3696	10
6752	179	CCGCCAGCGT	2644, 3903	10
6753	179	CCGGAGCGGC	1222, 3399	10

6754	179	CCGTGGGCCA	901, 4809	10
6755	179	CGCCGCGGCG	171, 4741	10
6756	179	CGCGGCCGGC	123, 2566	10
6757	179	GATGCGCGCC	2134, 2186	10
6758	179	GCAGGGGTTC	369, 4188	10
6759	179	GCCACGATGG	751, 2351	10
6760	179	GCCGCCGCGG	169, 2628	10
6761	179	GGAGCTCGCC	3049, 3277	10
6762	179	GGCGCCGTCG	4555, 4846	10
6763	179	GGCGTTGCGG	27, 3388	10
6764	179	GTCACCAACG	1138, 3833	10
6765	180	GCGGGCCGCGCCTAG	3803, 3850	15
6766	180	CTGCGCAGCGCCGC	7, 2636	14
6767	180	CGCACCGTCGCCG	165, 3049	13
6768	180	CGCCGCGGCGACC	1997, 4400	13
6769	180	CCGCCGCGGCGA	1996, 3603	12
6770	180	CCGCGCCGTCCG	1169, 3174	12
6771	180	CGCCGGCAGCG	2057, 2910	11
6772	180	CGCGGCTGCGC	2, 3465	11
6773	180	CGCTGGCCGAG	2466, 3311	11
6774	180	CTGGCGGCCGC	2992, 3794	11
6775	180	GAGTCGGCGTG	3298, 3756	11
6776	180	GCCCGCCGCCG	756, 2118	11
6777	180	GCCGGAGTCGG	1251, 3752	11
6778	180	GGTGATCGCCG	2802, 3033	11
6779	180	ACCGAGCAGC	1919, 2618	10
6780	180	ACGCCGCGGC	1987, 4399	10
6781	180	AGCTGTCGGC	1022, 2361	10
6782	180	CCGAGCAGCA	1307, 3894	10
6783	180	CCGGGGCCGA	667, 2544	10
6784	180	CCTCGCCGGC	1514, 2907	10
6785	180	CGAAGGTCGG	1726, 4054	10
6786	180	CGACCCGCAG	804, 4852	10
6787	180	CGCCCTGGCG	2410, 3790	10
6788	180	CGCCGGGCGC	2134, 2403	10
6789	180	CGCGTGCACC	2042, 3641	10
6790	180	CGGCACCGAC	2871, 3438	10
6791	180	CGGCCGAGCT	3239, 4869	10
6792	180	GCCGTCGGGC	2784, 3659	10
6793	180	GCGATGAACG	2028, 3509	10
6794	180	GCGCCGACCC	3155, 3695	10
6795	180	GCGGCCGAGC	1216, 4868	10
6796	180	GCGGCGGTTG	1635, 4314	10
6797	180	GGCCGCGTCG	4079, 4511	10
6798	180	GGGTGCGCGT	1652, 3416	10
6799	180	GGTGATCGCG	2455, 3819	10
6800	180	TCCGGCTCCG	85, 3960	10
6801	180	TCGTGGCCGC	611, 3675	10
6802	180	TGGGCGGCGG	4311, 4732	10
6803	180	TGGTGATCGC	2801, 3818	10
6804	181	GGCGTCGACGTCG	37, 4225	13
6805	181	CGGCCACGAGCG	2821, 3043	12
6806	181	TTGGCCGGCACC	2963, 4804	12
6807	181	CACCGACATCG	1375, 3133	11
6808	181	CAGCCCGCCGT	184, 382	11

6809	181	CCCGAAGCGGC	323, 937	11
6810	181	CGCGGCCGACC	3280, 3301	11
6811	181	GCCGCGGCGTC	32, 731	11
6812	181	GGACCGGCTGC	3970, 4098	11
6813	181	GGCGCGCCGCC	2620, 3490	11
6814	181	GGTGCCCAACG	1594, 3583	11
6815	181	ACCACGCCGT	2898, 4142	10
6816	181	AGCCGGCGCC	3939, 4044	10
6817	181	CACCGAGAAC	793, 1411	10
6818	181	CACCGCGGAC	3106, 4536	10
6819	181	CAGCGCGGCG	352, 4354	10
6820	181	CCACCGCGGA	657, 4535	10
6821	181	CCATCCTGAT	1557, 4497	10
6822	181	CCCGACGACC	2927, 3116	10
6823	181	CCCGGCCGCG	2266, 4013	10
6824	181	CCGGCACCGA	789, 3477	10
6825	181	CGACCACGCC	3145, 4140	10
6826	181	CGCCGACGTC	1693, 2574	10
6827	181	CGGCAAGCCG	2212, 3538	10
6828	181	CGGCCCAGTC	174, 3213	10
6829	181	CGGCCTGATG	1570, 2158	10
6830	181	CGGCGCCTGC	847, 2446	10
6831	181	GAAGCTGGCG	2584, 4219	10
6832	181	GATGTGGCGG	1510, 2188	10
6833	181	GCAAGATCAA	1494, 2202	10
6834	181	GCCCGGCGCG	3200, 3634	10
6835	181	GCCGCTGACC	1987, 2632	10
6836	181	GCGCCCGATC	2768, 4393	10
6837	181	GTCCGGGCGC	4378, 4872	10
6838	181	TCCCGGCCGC	2265, 2601	10
6839	181	TGGTGGCCGA	3726, 4121	10
6840	182	GGCTGCCCGGCATCG	1678, 2437	15
6841	182	GCAGCACCCGCGCC	67, 199	14
6842	182	GCGCGCCGCCGTC	763, 4811	13
6843	182	ACCGGCTACACC	2921, 4055	12
6844	182	GCGGCGACGACG	1920, 3804	12
6845	182	GGGCGTGACGCC	4749, 4866	12
6846	182	ACGTCGACGCG	606, 2410	11
6847	182	CGTCGCCGGTG	166, 619	11
6848	182	CTCATCGCGCC	237, 1502	11
6849	182	GCAACGCCCAC	691, 3189	11
6850	182	GGCTCGGCGAT	783, 1032	11
6851	182	GGGCCAGCTGG	517, 1426	11
6852	182	TCGCCGACCGC	1455, 2359, 2613	11
6853	182	TCGCGCTGGGC	2895, 3963	11
6854	182	TCTACACCGGC	3903, 4050	11
6855	182	ACGGATTTCC	2235, 3177	10
6856	182	AGCATCGCCA	585, 3824	10
6857	182	CAAGGAGCAG	3100, 3769	10
6858	182	CACGCCTTCG	812, 2493	10
6859	182	CCATCGCCGC	3321, 4184	10
6860	182	CCCGGACAAC	2548, 3370	10
6861	182	CGACACCGCC	2818, 4212	10
6862	182	CGCGGACTGG	476, 1024	10
6863	182	CGCGTCGCCG	164, 2609	10
6864	182	CGGCTCGGCG	647, 782	10

6865	182	CGTCGCCGAA	703, 4526	10
6866	182	CGTGGCGCGG	31, 370	10
6867	182	CTACCGGCCG	892, 3422	10
6868	182	GCCGCGCGCG	158, 470	10
6869	182	GCGCAGCGCG	758, 4506	10
6870	182	GCTCGGCGGC	331, 3799	10
6871	182	GCTGTACCCC	1955, 3883	10
6872	182	GGACAGCGCC	257, 494	10
6873	182	GGACCCCGAG	940, 3445	10
6874	182	GGCGCCTGGG	1373, 2073	10
6875	182	GGCGCGCAGC	62, 2626	10
6876	182	GGCGGTGGCG	56, 533	10
6877	182	GGTGTCCAGC	548, 4296	10
6878	182	GTCGCCGAGC	1094, 4839	10
6879	182	TCAACGCGCT	3225, 3579	10
6880	182	TGGCCAAGCT	3306, 3792	10
6881	183	ACCAGCTCGGCCAG	2866, 3664	14
6882	183	CGGCGGCGAAGTC	1790, 2627	13
6883	183	GACGCGGGTGGCG	1410, 1539	13
6884	183	ACCGCGACCCGG	2437, 4558	12
6885	183	CAGCGCCGGCCC	3603, 3726	12
6886	183	CAGCTCGTCGAG	4125, 4860	12
6887	183	CCCGGCCGGTCC	3926, 3988	12
6888	183	CCGCGCACCGCC	1188, 1903	12
6889	183	CTGGGCCAGGCG	1623, 1953	12
6890	183	GCGATAGCGCAC	23, 2148	12
6891	183	CAGCCGCTCGG	422, 3315	11
6892	183	CCACCGCGTCG	283, 811	11
6893	183	CGACGTCGACG	2738, 3431	11
6894	183	CGCCGTGCCGC	3962, 4157	11
6895	183	CGGCCCGGGCG	748, 4074	11
6896	183	GACCAGGTAGG	638, 4335	11
6897	183	GCCGCGCCGAT	483, 2014	11
6898	183	GCGCAGCTCGT	1770, 4122	11
6899	183	GCGCGGGACAG	2668, 3367	11
6900	183	GCTCGGTCCAG	1838, 4105	11
6901	183	GGATGACGTCG	1676, 4139	11
6902	183	GGCCAGCGCCA	3780, 3894	11
6903	183	GTTCGGCGGCG	3491, 4502	11
6904	183	TGATCGGTGTC	3502, 4783	11
6905	183	TGCGCGCGGGA	2274, 2665	11
6906	183	ACCACCACCG	3868, 4552	10
6907	183	AGACGTCGAC	1520, 2606	10
6908	183	CACCAGCTCG	2649, 3663	10
6909	183	CACGATCGCG	2409, 3045	10
6910	183	CAGCACCAGC	1179, 3753	10
6911	183	CAGCCGGACG	659, 4365	10
6912	183	CCACCGTCGC	2105, 3256	10
6913	183	CCAGCCGCTC	2348, 3314	10
6914	183	CGAATCCCAC	2528, 4020	10
6915	183	CGACAGCTCG	1401, 1611	10
6916	183	CGCAACGCCT	3079, 4842	10
6917	183	CGCAGCGCGG	2992, 4620	10
6918	183	CGCCCGGCCG	3834, 3986	10
6919	183	CGCGCAGCGC	2990, 3338	10

6920	183	CGGCCGCACC	871, 1971	10
6921	183	CTCGTCCAGG	2331, 4707	10
6922	183	CTGGGCGATC	2070, 3681	10
6923	183	GACCGCGGTC	2679, 3099	10
6924	183	GCAGCGCTTT	1026, 2846	10
6925	183	GCCGCCGCGC	339, 2011	10
6926	183	GCCGTGCAGC	1230, 3561	10
6927	183	GCGCTGCAGC	1851, 3297	10
6928	183	GCGGCCGCCG	336, 536	10
6929	183	GGACGCCGCG	1343, 4000	10
6930	183	GGCGGCCAGC	3309, 3777	10
6931	183	GGGCCCGCCG	2562, 2759	10
6932	183	GGTGCCGCCG	161, 4428	10
6933	183	GGTGTGCAGC	2547, 4359	10
6934	183	GGTTGCGCCG	684, 3957	10
6935	183	GTCGACCGCG	1455, 3096	10
6936	183	TCATGGCCGC	9, 2492	10
6937	183	TCCAGGCCCG	2917, 4609	10
6938	183	TCGGCGGCGA	2626, 3355	10
6939	183	TCGTCGAGGC	216, 2287	10
6940	183	TGACCAGGTA	637, 2891	10
6941	183	TGCTTTCCAG	358, 1208	10
6942	183	TGTCCAGGGT	1718, 3653	10
6943	184	GTCGACGAGCTCAC	1268, 2484	14
6944	184	CGCGCCGGACGCC	220, 697	13
6945	184	CGCCGGCGCGGA	52, 2397	12
6946	184	GCCGACGTGGCC	356, 4590	12
6947	184	GGTGGTGCTCGA	3689, 4646	12
6948	184	GTCGCCGACAAG	2548, 4761	12
6949	184	TCGGCGCCCACC	711, 1194	12
6950	184	AACCCGTGGTG	1709, 3640	11
6951	184	ACGACGGCGCC	483, 3149	11
6952	184	CCGTCGCCGAC	2237, 4759	11
6953	184	ACCCTGACCG	2935, 4248	10
6954	184	ACGGCGCGCC	693, 2909	10
6955	184	ACTGGGGTCC	2987, 3122	10
6956	184	AGACGCTGGA	3604, 4684	10
6957	184	CAACCGCATC	385, 3971	10
6958	184	CCCGAGGAGG	1508, 3681	10
6959	184	CCGCCGCCGA	1055, 4342	10
6960	184	CGACGCGGTG	1090, 3827	10
6961	184	CGCCACCAAG	3878, 4442	10
6962	184	CGCGGGCCGA	875, 1361	10
6963	184	CGGCGACACC	1390, 3213	10
6964	184	CGGGTGCTCA	470, 983	10
6965	184	CGTGCTGGCG	3509, 4325	10
6966	184	CTGACCGACG	3235, 3366	10
6967	184	CTGTCCGACG	401, 2446	10
6968	184	GATCGCCGCG	730, 2685	10
6969	184	GATCGCCGGC	430, 640	10
6970	184	GATCGGCGCC	148, 1423	10
6971	184	GATCGTGCTG	19, 3506	10
6972	184	GCGCACCGGG	2760, 4615	10
6973	184	GCGCCGGCGC	51, 3862	10
6974	184	GCGCGACGGT	1260, 2123	10
6975	184	GCGCTGGTGG	959, 4641	10

6976	184	GCGGGCTGGC	924, 4053	10
6977	184	GCGGGTCGCC	3389, 4268	10
6978	184	GCTGGTGGGC	844, 4577	10
6979	184	GGGCCAGCGC	1672, 4163	10
6980	184	TCATCGAGCT	105, 3706	10
6981	184	TCCCGCTGAC	885, 3230	10
6982	184	TCGGGCCGCA	1035, 2806	10
6983	184	TGGGGGCGGC	2846, 4492	10
6984	184	TGGTGCCGAT	1557, 4093	10
6985	184	TGTCGGCGCC	1192, 4751	10
6986	185	CCCGTCGCCGGG	1797, 3526	12
6987	185	GCCCGTGGCCGC	3032, 3751	12
6988	185	GCGGCCGGCGCC	1045, 2034	12
6989	185	GGCGGCGCGGTG	2298, 2833	12
6990	185	TAGACGCCGTCC	3662, 4760	12
6991	185	TCGACCGCGGCC	545, 4793	12
6992	185	TCGGCGCCGGCC	841, 2633	12
6993	185	ACCGCGACGTC	2098, 3156	11
6994	185	CGATGGGGGCC	90, 3340	11
6995	185	CGTCGGTGCGG	2823, 4626	11
6996	185	GCGCGACGCCG	2312, 4476	11
6997	185	GTGCTGCTGGC	248, 2653	11
6998	185	TCGCCCGCGCC	1549, 1696	11
6999	185	TCGTGGCGGTG	1499, 2864	11
7000	185	ACCCGAGGGC	1305, 2216	10
7001	185	ACGTCGGTGC	2822, 4499	10
7002	185	ATCGAGCGGG	203, 3949	10
7003	185	CCGAATACGG	2807, 4425	10
7004	185	CGCCGCCGCG	757, 1455	10
7005	185	CGGGATGTCG	4105, 4240	10
7006	185	CGTCGCCGTC	3996, 4695	10
7007	185	GATCGTGGCG	2751, 2862	10
7008	185	GCACGATCTC	3178, 3987	10
7009	185	GCCGCGGCGC	2898, 4075	10
7010	185	GCTGCCGTAG	3230, 3655	10
7011	185	GCTGGGCGCC	751, 1655	10
7012	185	GGGCCTGGCG	646, 1955	10
7013	185	GTCGGCGGCC	3167, 3778	10
7014	185	GTGCTGGTCG	3053, 3386	10
7015	185	TCGCGCCCGT	3000, 3028	10
7016	185	TCGTCGACGA	977, 4274	10
7017	185	TTCGGCGCCG	2250, 2632	10
7018	186	CAGCGCGGTGTC	907, 2077	12
7019	186	CCGGGCGAACAT	781, 1057	12
7020	186	GCGACGCCGGGG	993, 2374	12
7021	186	ATCCGCACGGC	3103, 4155	11
7022	186	CGCCGACGGCG	1481, 2841	11
7023	186	CGCGTCGAGCA	154, 394	11
7024	186	CGGCGACGCCG	2372, 3344	11
7025	186	CGGCGGCCGCT	1759, 2150	11
7026	186	GCCGCGTCGAG	152, 4807	11
7027	186	GCGCGCGTCGG	517, 808	11
7028	186	GCGTCGGCGGC	2146, 4829	11
7029	186	TGACCAGGCGC	2319, 3978	11
7030	186	TGTCCATCCGC	2999, 4150	11

7031	186	ATCGGCGGGC	1292, 3783	10
7032	186	CCCGGGCCGG	284, 1731	10
7033	186	CCGGAGGCGC	176, 968	10
7034	186	CCGTCGGCTC	1338, 4703	10
7035	186	CGCAGCCGGC	605, 2249	10
7036	186	CGCCGCGGCC	838, 3747	10
7037	186	CGGGTGCGGC	3338, 4716	10
7038	186	CTCCGCGCCG	2394, 2445	10
7039	186	GACGGGACCT	1360, 3566	10
7040	186	GCAGCAACGA	2804, 3437	10
7041	186	GCCGAACGCC	2514, 2535	10
7042	186	GCGCGGCGGT	858, 1271	10
7043	186	GTCAGCGCGG	1157, 2075	10
7044	186	TCCGGGTGCG	1521, 3336	10
7045	186	TCGATGGGCA	2968, 3836	10
7046	186	TGGGGTTGCG	687, 1584	10
7047	187	GCGATGTACGGCTACGCCGG	761, 2026	20
7048	187	GAGTCGGTGATCTCGAAC	452, 1717	18
7049	187	CAATGGATTTCGGAG	315, 1580	15
7050	187	GCGCCGTATGCGGC	521, 1786	14
7051	187	GCCGCCGCGGCG	395, 509	12
7052	187	GGTCGAATGTAC	353, 1618	12
7053	187	TGGCCGCCACCG	1434, 2579	12
7054	187	CCGCCGCCGCG	393, 818	11
7055	187	CGGCGGCCGCC	786, 1293	11
7056	187	CGGCGTTGCGG	3912, 3993	11
7057	187	GCCGCCGCCGC	392, 971	11
7058	187	GCCGGCTGGCG	1922, 3079	11
7059	187	GCGCGGGTGCC	2606, 2636	11
7060	187	GCGCGGTCGCC	2129, 2705	11
7061	187	GGCCGCCGCCG	391, 859	11
7062	187	ACGCCGCCGC	432, 2018	10
7063	187	AGATGTGGGC	738, 2003	10
7064	187	AGCCGGTCAG	3972, 4709	10
7065	187	ATCCGGCCGG	837, 1310	10
7066	187	ATCGCGGCCA	641, 1975	10
7067	187	CAGGTCCGGC	3802, 3832	10
7068	187	CATCGCCGGG	162, 4556	10
7069	187	CCCAGTTGCA	423, 3152	10
7070	187	CCGGTGGCGA	1897, 3317	10
7071	187	CGACCGCCGC	546, 1811	10
7072	187	CGCCGAGAGC	1209, 4599	10
7073	187	CGCGGCATCG	76, 3857	10
7074	187	CGCGGCCGGC	1918, 2628	10
7075	187	CGGCGCTGGG	1155, 4113	10
7076	187	CGGGCGCGCC	1423, 1486	10
7077	187	CGGGTCTGGC	1413, 2516	10
7078	187	CGGTGCCGCC	624, 1275	10
7079	187	GATCCGGCCG	196, 1309	10
7080	187	GCCGATGATG	18, 3982	10
7081	187	GCCGTTGGCG	2295, 2476	10
7082	187	GCGACACCGG	3311, 3542	10
7083	187	GCGTAGCTGC	3172, 3812	10
7084	187	GCTGGCCGCC	388, 2577	10
7085	187	GGCCGCGGCG	1656, 1854	10
7086	187	GGCCTTGTCG	2175, 4387	10

7087	187	GGCGAATGTG	1111, 3337	10
7088	187	GGGCGTCGAA	1078, 3891	10
7089	187	GTCAAGCATC	3440, 3462	10
7090	187	TCAGCTTCAG	3825, 4272	10
7091	187	TTCGGCGATC	2049, 3649	10
7092	187	TTGGATCAGC	2863, 4521	10
7093	188	CGGCCAGCCGGCC	215, 3409	13
7094	188	TCGGCGGCGTCGG	567, 1563	13
7095	188	ATCAGCGGCAAG	1512, 2007	12
7096	188	CGGATCGGCGGC	563, 2039	12
7097	188	CGGCGGCGCGGA	3697, 3915	12
7098	188	GATCGCGGCGAC	1106, 1808	12
7099	188	GCACCAGGCCCG	1237, 1699	12
7100	188	GGGCACAGGTCC	2217, 3330	12
7101	188	TCACCTACAACG	4505, 4826	12
7102	188	ATCGCGTTGAC	2064, 3309	11
7103	188	CCGATCGCGAT	1395, 4495	11
7104	188	CCGGTGATGAC	1419, 3819	11
7105	188	CGCGGGAATCG	2057, 4362	11
7106	188	CGGTCAGCCGG	385, 3346	11
7107	188	GCGGCCCGGTG	2842, 3993	11
7108	188	GCGGCGCGCCA	584, 905	11
7109	188	GTGACCTCGAT	1881, 2166	11
7110	188	ACGACAGCAC	817, 4184	10
7111	188	ACTGCGGCCC	175, 2839	10
7112	188	AGCTCGGCGG	2913, 3639	10
7113	188	CACCAGCCCG	1334, 2093	10
7114	188	CCGCCGACGA	196, 1551	10
7115	188	CGAGGCGATG	2333, 3746	10
7116	188	CGCCAGGTCG	440, 3662	10
7117	188	CGCGCCGGTA	397, 2776	10
7118	188	CGGCCAGCGC	2593, 2815	10
7119	188	CGGTGGCCAC	505, 2086	10
7120	188	CTCGAGCGCC	2789, 4266	10
7121	188	GACGGGGATG	3038, 3089	10
7122	188	GATCATGGCG	950, 3863	10
7123	188	GCAGGTAGCC	301, 1744	10
7124	188	GCCGCGGCGG	2304, 3911	10
7125	188	GCCGGCCAGC	2591, 3632	10
7126	188	GCTTGATCAG	781, 2689	10
7127	188	GGCGGTGCCG	3380, 3503	10
7128	188	GGTGACCTCG	2165, 2381	10
7129	188	GGTGGGGTCG	851, 4756	10
7130	188	GTGCCGGCCA	1140, 2589	10
7131	188	TCGGGCGCGG	208, 4357	10
7132	188	TGTACACCGC	1255, 2827	10
7133	189	TGGCGACCTACGAGATCGTGTG	136, 1502	22
7134	189	CCGGTTCCTACCCGATCGTG	115, 1481	20
7135	189	GCCTTCTTGGCGGCGGTCTT	3683, 3713	20
7136	189	TTCCTGCAAAGCACCATCGG	201, 1567	20
7137	189	CGATCAACGCCATC	295, 1661	14
7138	189	CGATCGTGGTCGC	1817, 2879	13
7139	189	GCGCCGCGACGAT	2689, 2813	13
7140	189	CAGGGCAACGAC	63, 1429	12
7141	189	CCGACGCGTTCA	422, 1631	12

7142	189	CTTGGCCAGCAG	3820, 4201	12
7143	189	GTCGCCGCGGCC	1795, 3595	12
7144	189	ACGCCGCCGCG	902, 1172	11
7145	189	CGATCGCCATC	962, 4170	11
7146	189	CGCGCTGGTGC	1992, 2850	11
7147	189	CGTTCGGCCGC	2399, 2485	11
7148	189	GGTGGTGGACC	2082, 2921	11
7149	189	TCGCGCTGGTG	2849, 3212	11
7150	189	TCGGTGATGAT	2269, 4355	11
7151	189	TCGTTCTACAA	93, 1459	11
7152	189	AGGCCCTCAA	713, 1073	10
7153	189	AGGTCGGCGC	178, 4782	10
7154	189	ATCAGCGCCG	12, 1378	10
7155	189	CAGCAACCAG	318, 1706	10
7156	189	CCGACCCGGC	105, 4860	10
7157	189	CCGGCGGCGG	2231, 2793	10
7158	189	CCGGTGCCAC	43, 1085	10
7159	189	CGACGTCGCG	382, 404	10
7160	189	CGCCGTGCTG	588, 3018	10
7161	189	CGGTCGGGGT	2914, 3953	10
7162	189	CGTCGGCGGC	1259, 2552	10
7163	189	GAAGCGGCGC	2341, 2722	10
7164	189	GCGAGACCGT	2456, 4798	10
7165	189	GCGCTGGGCG	2353, 2449	10
7166	189	GCTGGTCGGC	500, 3429	10
7167	189	GGATTCCTGC	1564, 3532	10
7168	189	GGGGTGCGGC	630, 3548	10
7169	189	TCCAGATGGA	2333, 4566	10
7170	189	TCGACGGCCC	733, 1007	10
7171	189	TCGTCGTCGG	679, 4349	10
7172	190	CGCGTCGGCACC	977, 2802	12
7173	190	GCGCGGCGTCAC	282, 3790	12
7174	190	ACGGCGCCGCG	119, 698	11
7175	190	CAGCCGGGCCG	1139, 2643	11
7176	190	CCGACCGCGAC	659, 2821	11
7177	190	CCGGGATCGGC	1261, 2492	11
7178	190	CGAGTGGACCC	1127, 2963	11
7179	190	CGCCGAAAAAG	809, 2009	11
7180	190	CTGATCGCCGG	1254, 4142	11
7181	190	GCCCGGCGACG	1165, 2558, 3808	11
7182	190	GTCGTCATCGG	2700, 3722	11
7183	190	GTGCTCGACGG	691, 2346	11
7184	190	TGGCACCGCTG	2854, 3216	11
7185	190	TGGGCGTTCGA	2196, 2439	11
7186	190	ACGCCGAAAA	808, 1987	10
7187	190	ATGGCGCAGA	1215, 4385	10
7188	190	CACCCAGGAA	2288, 2690	10
7189	190	CAGCAGCGGC	424, 2738	10
7190	190	CCACGGCGCC	117, 3417	10
7191	190	CCGACGGCGG	777, 4110	10
7192	190	CCGCGCGCGG	4042, 4329	10
7193	190	CGCGCGGCGT	2911, 3789	10
7194	190	CGGCAGCAAG	1295, 2042	10
7195	190	CGGCGACCTG	1619, 4609	10
7196	190	CTGCTCGATC	3140, 4301	10
7197	190	GAATACGGCG	1905, 1941	10

7198	190	GCCAACTGGG	3576, 4661	10
7199	190	GCGCGTCGGC	1613, 2801	10
7200	190	GCGGTCGCGA	2264, 2275	10
7201	190	GCTCACCGCG	4210, 4228	10
7202	190	GGCCGGCGTG	73, 520	10
7203	190	GGCGCTGGAG	1088, 2576	10
7204	190	GGCTGCACGA	3897, 4068	10
7205	190	GTCGCTGCGC	247, 1568	10
7206	190	TCGACGACCC	1048, 1558	10
7207	190	TCGCCGGGCG	901, 2794	10
7208	190	TGTTCGACCT	1183, 4182	10
7209	191	ACCGCCGCTTGCCTGATGGCGGCGGCCCGCTGCGCCCGGC TTGCGCCGCGCTTGCG	1979, 2035	56
7210	191	AGGCCGGAGACGTCGA	3161, 3650	16
7211	191	CCAGCGTGGCGGT	601, 4454	13
7212	191	CCAGCGGCTCGG	886, 2154	12
7213	191	CGGATGGGCAGC	2693, 4750	12
7214	191	GAAAACAGGTCG	189, 4150	12
7215	191	CCTCGAGCCGC	2226, 3258	11
7216	191	CGATCGGTCGC	1060, 4538	11
7217	191	CGGGGTCACCG	2352, 4875	11
7218	191	GCAGACGAAGA	1728, 3559	11
7219	191	GCCGTCGGGGG	1423, 2242	11
7220	191	GCCGTCGTGCC	3242, 4731	11
7221	191	GCTTCCGGCGA	348, 4440	11
7222	191	TCGCGGCGAGC	2649, 3926	11
7223	191	TGAGCTTGGCC	2322, 3342	11
7224	191	ACGCCGGCGG	2682, 3278	10
7225	191	CACGCCGTCG	486, 3742	10
7226	191	CAGCAGCCGC	428, 4190	10
7227	191	CCACCAACGC	2676, 3324	10
7228	191	CCCTCGGCCA	318, 474	10
7229	191	CGCGATCCCG	3514, 3966	10
7230	191	CGCGTCGGCG	2407, 3427	10
7231	191	CGGAAGCCGC	3193, 3675	10
7232	191	CGGCGAACCG	385, 4353	10
7233	191	CGGCGATCGA	3762, 4799	10
7234	191	CGGGCCACGG	664, 2601	10
7235	191	GATCGGGACG	2431, 3837	10
7236	191	GCCCACACCG	2617, 3092	10
7237	191	GCCGGCTTCT	1510, 2299	10
7238	191	GCCGTCCAGG	2377, 2788	10
7239	191	GCGCCCACGC	972, 4237	10
7240	191	GCGGAAGCCG	2191, 3674	10
7241	191	GCGTCGACGA	1184, 3146	10
7242	191	GCTCAGCGCG	1206, 4218	10
7243	191	GCTCGATCGG	3833, 4535	10
7244	191	GGATGACGGC	3666, 4432	10
7245	191	GGCGCGCACC	878, 4584	10
7246	191	GGCGTCGACG	1183, 4830	10
7247	191	GGCTGGCCAA	77, 1355	10
7248	191	GTAGGCGTCG	2254, 4473	10
7249	191	GTCGCCGTCG	1420, 3239	10
7250	191	TCCGCACCGC	109, 4050	10
7251	191	TCGGCGATCG	2864, 4798	10

7252	191	TTCTCCAGCG	339, 2150	10
7253	192	CGCCGGCATCGGCAA	2836, 3492	15
7254	192	CCAGCGGGCCGAGG	145, 2247	14
7255	192	TCCCCGGCATCACC	1985, 4250	14
7256	192	CCCGTCAAGGTG	269, 3754	12
7257	192	CCGGCAAGCTGT	2904, 3524	12
7258	192	CCCGGGCGGGC	2538, 3883	11
7259	192	CCTCGGCGGTG	4064, 4388	11
7260	192	CGACGCGCTCG	23, 1050	11
7261	192	CGCTGGTGCAG	3575, 3920	11
7262	192	CGGCGATGCGG	647, 2100	11
7263	192	CGGCTTCGACC	136, 2647	11
7264	192	GATCACCACCG	5, 2061	11
7265	192	GCGCGCAGATC	1782, 1835	11
7266	192	TGCGCGTGCAC	2613, 3030	11
7267	192	ACAGGCCGAT	4673, 4802	10
7268	192	ACCTCGGTGA	84, 596	10
7269	192	ACCTGGTGGC	1700, 3563	10
7270	192	ATCGCCGGCA	2206, 2834	10
7271	192	CAGCGACGAG	2857, 3453	10
7272	192	CATCACCGAC	2815, 4794	10
7273	192	CCGCGCTGGA	3401, 3593	10
7274	192	CCGGGCGTCA	3379, 3670	10
7275	192	CGAAGATCTC	933, 2880	10
7276	192	CGCCGATCAG	945, 2436	10
7277	192	CGCCGTCGCG	819, 1809	10
7278	192	CGGCACCCAG	281, 2668	10
7279	192	CGTCGCCGCG	3396, 3861	10
7280	192	CTGGCCGACC	2348, 3149	10
7281	192	GAAGCCGGCG	440, 625	10
7282	192	GCCGCGAAAC	251, 4600	10
7283	192	GCCGCGGCGC	565, 2573	10
7284	192	GGCCGGCACC	2665, 4437	10
7285	192	GGCGGGTGTG	34, 4281	10
7286	192	GGTCGAGCAG	418, 1541	10
7287	192	GGTCGGCGAA	2043, 2217	10
7288	192	GTCCTGGCCG	404, 3146	10
7289	192	GTCGGCCAGG	2984, 4221	10
7290	192	TCCCGGCCGA	1197, 4777	10
7291	192	TTGGACTCCA	4570, 4705	10
7292	193	ACGCGCTCGCCGAC	2673, 4747	14
7293	193	CGGCGCGCGGCGG	66, 4487	13
7294	193	GGTGTTGATGTG	470, 4773	12
7295	193	ACGCGCTGGAC	2175, 3246	11
7296	193	CAACCACGCCC	2296, 3889	11
7297	193	CCGGTCGGCGC	4171, 4482	11
7298	193	CGCCGAAGGCG	1217, 2590	11
7299	193	CGCCGACGCGG	786, 2077	11
7300	193	CGTCGCCGAGG	40, 2656	11
7301	193	CGTGGCGGTGT	2209, 2938	11
7302	193	GCCGCGGCCGA	1802, 2990	11
7303	193	GGCGTCCAGCC	1057, 4004	11
7304	193	GGGTCGAAGAA	1442, 3807	11
7305	193	GGTGCTCGAAC	3115, 3757	11
7306	193	GGTGGCGCCGA	662, 4211	11
7307	193	TCGGCGTGCCG	4807, 4889	11

7308	193	ACGGCACCGG	1422, 4637	10
7309	193	AGCCGCAGCG	432, 546	10
7310	193	AGCGCGCCGG	3674, 4140	10
7311	193	ATCGTCGCCG	781, 1685	10
7312	193	ATGAGTGTGC	1347, 1574	10
7313	193	CAGCACCGCC	449, 4040	10
7314	193	CAGGTGTGGG	838, 4686	10
7315	193	CCGGGCCCGC	1846, 3529	10
7316	193	CCGGGCGCCG	1212, 2562	10
7317	193	CGACGCCGAC	1951, 2401	10
7318	193	CGGCGGCTGT	1967, 3539	10
7319	193	CTGCGCAACC	333, 1898	10
7320	193	GAACTGGCGC	647, 3170	10
7321	193	GATCGGTCCG	4261, 4475	10
7322	193	GCGGCGGCTG	1966, 3502	10
7323	193	GCTCAACCCG	1633, 3592	10
7324	193	GCTGATCGAC	2065, 3847	10
7325	193	GGGACGGGTC	3802, 3868	10
7326	193	GGGCGAGAAA	3952, 4228	10
7327	193	TCGCCGCGGC	2021, 2988	10
7328	194	CACCCCGGGCGAG	142, 2667	13
7329	194	CCAAGGTCATCGT	2276, 2879	13
7330	194	AAGGCCGGCACC	2338, 2377	12
7331	194	CGGCCACGCCGA	1731, 4080	12
7332	194	CGGCGGCGGCGC	319, 3332	12
7333	194	CTGCTGGACCGC	1387, 4108	12
7334	194	GGCGGGCGCGGC	494, 1225	12
7335	194	TCGAGGACCGCG	1745, 3692	12
7336	194	ACAACGTCGAC	2126, 2609	11
7337	194	ACCCGCTGGTG	1646, 2168	11
7338	194	ACCGCACCTAC	1403, 4755	11
7339	194	CACCGTCGCCG	2442, 3399	11
7340	194	CATCGGTGGTG	1929, 2808	11
7341	194	CCATCGCCGAG	337, 1535	11
7342	194	CCGAGGAGCGC	2918, 4055	11
7343	194	CGCCGACCGGG	716, 3645	11
7344	194	CTCGGCCTCGG	4137, 4813	11
7345	194	GACCCCGTCGG	2404, 3102	11
7346	194	GCGGCGACGCC	1258, 2909	11
7347	194	GCTGCTGGTCG	1335, 2550	11
7348	194	GGTCGGCATCA	74, 1824	11
7349	194	TCGTGGTCCGG	2105, 4709	11
7350	194	TGCCGGTGTTC	2429, 4232	11
7351	194	AAGGCCGGCG	1237, 2138	10
7352	194	ACACCGTCGC	3398, 3734	10
7353	194	ACCGCGCTGA	2065, 2323	10
7354	194	AGGAAGCGCT	3065, 4160	10
7355	194	AGGACCCCGA	2867, 4655	10
7356	194	AGGCGCAGGT	1208, 3590	10
7357	194	AGGTCGTCGA	522, 540	10
7358	194	CAACAACTAC	3462, 4329	10
7359	194	CATCGACGCC	2532, 2841	10
7360	194	CCTGACCTAC	2751, 4125	10
7361	194	CGAACACGCC	1281, 3242	10
7362	194	CGACGACGTC	415, 4191	10

7363	194	CGAGATCGGC	1182, 2901	10
7364	194	CGCCGACTAC	2931, 3819	10
7365	194	CGCCGTCGCC	2043, 3378	10
7366	194	CGGCGAGCCG	4686, 4785	10
7367	194	CGGTCGAGGG	1427, 3677	10
7368	194	CGGTGGCCGC	175, 4025	10
7369	194	GAAGGCCGGC	2137, 2337	10
7370	194	GCCACGCTGG	1627, 4741	10
7371	194	GCCGCGCTGC	3346, 4009	10
7372	194	GCGCCGCGCC	3497, 3792	10
7373	194	GCGGCGCTCG	446, 3118	10
7374	194	GCTGGACGGC	1683, 1812	10
7375	194	GCTGGTGGCC	296, 3975	10
7376	194	GGCCATCGAC	2529, 3390	10
7377	194	GGCCGCGCTG	365, 3345	10
7378	194	GGCGAACAAG	2091, 2226	10
7379	194	GGCGACCGCG	355, 1521	10
7380	194	GTCACCGAGT	3556, 3931	10
7381	194	GTCGGCGTGG	287, 3259	10
7382	194	GTCGGTGGCC	3477, 4023	10
7383	194	TGTGGGCGGC	307, 2355	10
7384	195	GCGAGCCGGGGTCTCGTGTGGCGCAGGGCGATGCCGCGG AG	3200, 3242	41
7385	195	CATCGGTGGCCGCG	1159, 2091	14
7386	195	CGCCGGAGAGATG	383, 660	13
7387	195	CGGTGATCACCGA	2285, 4660	13
7388	195	GCAGTACGGCCAG	1468, 1495	13
7389	195	TTGCCCTATTGGC	2413, 3459	13
7390	195	ATCGCCGCGGTC	1388, 1984	12
7391	195	CCGCGTCCGCCG	2444, 3050	12
7392	195	CGCTGCTGCCGG	2120, 3918	12
7393	195	GCCGCCGCCGTC	3493, 3650	12
7394	195	GCTATCACCGAG	1301, 3797	12
7395	195	GGCGGCCAGCAG	1529, 1544	12
7396	195	GTCGCCGTCGGC	2626, 3683	12
7397	195	GTGCTCGACGGC	4055, 4410	12
7398	195	TGGACTCCGGCG	1413, 4564	12
7399	195	CCGACCGCGAC	3696, 3774	11
7400	195	CCGCGAAGAAC	4342, 4636	11
7401	195	CGCCACCGCGG	450, 898	11
7402	195	CGGTCGTCGGG	535, 560	11
7403	195	CGTCGGCGCTG	113, 2219	11
7404	195	GATCGCCGCGG	1387, 3721	11
7405	195	GCGTGGCGGTG	2783, 4654	11
7406	195	GCTGGCCGCGG	4301, 4694	11
7407	195	GGCCGCCGGGG	778, 2859	11
7408	195	TCGGCGAGTTC	331, 2558	11
7409	195	TGCTGGCCGCG	1206, 4693	11
7410	195	ACGCCGAGGC	364, 4486	10
7411	195	CAGCGCCCGC	264, 1836	10
7412	195	CATCGCGGTG	1276, 4781	10
7413	195	CCACCGCCGC	983, 3810	10
7414	195	CCGACCCAGG	1598, 3024	10
7415	195	CCTCGCCGTC	790, 2259	10
7416	195	CGACGATCGG	3099, 4176	10
7417	195	CGACGCGTCG	549, 2274	10

7418	195	CGCGCAGCAC	1669, 2156	10
7419	195	CGGCGCCCAG	494, 1630	10
7420	195	GCGGCGCCCA	157, 493	10
7421	195	GCGTCGGCGC	2218, 3590	10
7422	195	GCGTGCTGGC	2393, 4690	10
7423	195	GGCGGCACGT	316, 998	10
7424	195	GGGCGCGCAT	3526, 3937	10
7425	195	GTGGTGGCGT	1966, 2212	10
7426	195	TGAGCCGGGG	3144, 3159	10
7427	196	TGCAGATCATCGCC	2535, 4606	14
7428	196	CTGCTGCGCCGCG	2789, 3222	13
7429	196	GGCGCAACAACTT	3304, 4241	13
7430	196	GCGGCATCGTCG	1667, 2235	12
7431	196	GGCCGAGCAGCT	55, 4016	12
7432	196	ACCGACGGCGA	3280, 4770	11
7433	196	CACCGAAGTCA	274, 319	11
7434	196	CCGCCGCCGAG	2622, 4645	11
7435	196	CCGCTTCGGCG	2428, 3407	11
7436	196	CCTGCCGGTGG	2065, 2935	11
7437	196	CGAATGCGATC	229, 591	11
7438	196	CGCCGCCGCGC	2992, 3773	11
7439	196	CGTGCGCTGGT	1943, 4137	11
7440	196	CTGGCCAAGCG	3029, 3759	11
7441	196	GCGCTGGCGCT	4557, 4704	11
7442	196	GGCGCCACCAT	617, 4044	11
7443	196	GGGGGACCCCG	520, 2188	11
7444	196	GTCGGCGCAGG	1052, 2509	11
7445	196	ACGCGCTGGC	4072, 4702	10
7446	196	AGGCGGTGCT	2772, 3355	10
7447	196	ATCGCCGCCG	605, 2618	10
7448	196	CGAGCTGCTG	3218, 3743	10
7449	196	CGCGCCGCCG	1733, 2990	10
7450	196	CGCGGCCAAC	265, 829	10
7451	196	CGCGGTCGCG	706, 4484	10
7452	196	CGGGTGCTGG	389, 2822	10
7453	196	GAACGGCCAC	1011, 4396	10
7454	196	GACCATCGCC	2200, 4853	10
7455	196	GACGAGGCGG	3351, 4095	10
7456	196	GCAGGCCGCG	7, 4478	10
7457	196	GCCGCCACCC	2045, 4732	10
7458	196	GCGCCTGCCG	2341, 2932	10
7459	196	GCGGCGCGGT	702, 4259	10
7460	196	GCTCGGCGAG	1906, 4214	10
7461	196	GGATGCCTTG	3827, 4598	10
7462	196	GGCCGCCGCC	2641, 3770	10
7463	196	GGCGGCGCGG	3902, 4258	10
7464	196	GGGTGTCGGC	2598, 2898	10
7465	196	GTGATCGCCG	602, 1979	10
7466	196	TTCGTCGGCC	190, 1114	10
7467	197	GCCGTTGATGGCGG	2242, 3469	14
7468	197	GGGTGGCCGCTTC	1804, 2763	13
7469	197	CGCCGCGGCGCG	1422, 2086	12
7470	197	GGGCGACCAGCG	2481, 3977	12
7471	197	ACGGTCGAACC	1897, 4000	11
7472	197	ATCGGCCGCGC	1353, 1594	11

7473	197	CCGTCGGCGAT	3404, 4627	11
7474	197	CTCCTCGGTGC	3232, 3439	11
7475	197	GCGTCGGCGAG	4339, 4534	11
7476	197	GGTCTTGACGT	2953, 3828	11
7477	197	TCGGCCACCGC	3698, 4825	11
7478	197	ACCGGGGCGA	2657, 4312	10
7479	197	AGCGCGGCGA	380, 854	10
7480	197	ATCGACACCG	313, 4612	10
7481	197	CAACCAGATC	666, 3900	10
7482	197	CACCCACGAG	1657, 2208	10
7483	197	CAGCGCGAAC	2164, 2980	10
7484	197	CATCACCAGC	396, 3532	10
7485	197	CCGCGGCCGT	1184, 1917	10
7486	197	CGAGCACGGC	723, 3252	10
7487	197	CGCGGCGTCG	1996, 4335	10
7488	197	CGCGTCGGCG	2047, 4533	10
7489	197	CGGCCTCGGC	269, 2802	10
7490	197	CGGCGGCCAT	2013, 3516	10
7491	197	CGGCGTGTCG	597, 4561	10
7492	197	CGTCGAGGAA	3108, 4451	10
7493	197	CGTCGCTGGG	1035, 3087	10
7494	197	CTCGGCCGCG	1549, 1990	10
7495	197	CTCGGCGGCC	2011, 2965	10
7496	197	GAAGCCGCGA	1108, 1940	10
7497	197	GACGGTGTCG	211, 3927	10
7498	197	GCGCGCCGCG	439, 2083	10
7499	197	GCGGCGCCGA	3943, 4394	10
7500	197	GCGGGCCTCG	1840, 2152	10
7501	197	GCGTTCGGGG	3025, 3130	10
7502	197	GGCGCCGTCG	4446, 4818	10
7503	197	GGTGCTCGAC	771, 4883	10
7504	197	TCAGGTAGTA	2073, 3663	10
7505	197	TCGCTGCCGT	3314, 3674	10
7506	198	GTCGGCGCCGCCGG	2005, 4864	14
7507	198	CTCCTCGGTGTCG	1600, 4252	13
7508	198	CCGGCGAGCTGG	2694, 4766	12
7509	198	CGCCCGCCAGCG	2328, 3265	12
7510	198	CTTGGCCTCGAT	494, 4357	12
7511	198	GCGACGTGCGGC	717, 4055	12
7512	198	ACCCCCGCCGC	3078, 3431	11
7513	198	CGATGCGGCGG	1674, 2743	11
7514	198	CGCCGGTGGCC	1788, 4716	11
7515	198	CGCGCACGCCG	3130, 4458	11
7516	198	CGCGGGCCACG	1083, 2731	11
7517	198	CGGCCTCCTCG	1041, 4248	11
7518	198	CGGTGACGACG	3112, 4851	11
7519	198	CGGTGACGGTG	2172, 4578	11
7520	198	CGTAGCCGGGG	607, 4083	11
7521	198	CGTCGGCCAGG	37, 1144	11
7522	198	CGTCGGCGCCG	3415, 4863	11
7523	198	CTCCTCGATGA	158, 3290	11
7524	198	GCGTCGGCCTC	705, 1037	11
7525	198	GGCGCCGGCGA	3913, 4762	11
7526	198	GGGCGGTGACG	4521, 4575	11
7527	198	GGTGGGCAGGT	266, 1996	11
7528	198	TCGCCGCTGCG	3513, 3902	11

7529	198	TCTCGGCGCCG	1752, 2935	11
7530	198	AACACCAAAG	1907, 3998	10
7531	198	CAATTCGCGC	359, 3497	10
7532	198	CATCCGGAAG	853, 3954	10
7533	198	CCCCGCCCGC	381, 391	10
7534	198	CCGCCGATGC	2529, 4409	10
7535	198	CCGCCGCCCG	2324, 3225	10
7536	198	CCGCGTCGGC	1035, 3412	10
7537	198	CGCCGCGTCG	98, 1033	10
7538	198	CGGCGGCCGC	571, 4809	10
7539	198	CGTCGGCGTG	4041, 4266	10
7540	198	CTCGGCGTCG	2350, 2404	10
7541	198	CTTGATCATC	1285, 1843	10
7542	198	GAAGCCGGGA	3041, 4141	10
7543	198	GACCGTCGGG	2116, 4488	10
7544	198	GACTCCTCGC	1403, 1781	10
7545	198	GATTTCGGTG	2513, 4690	10
7546	198	GCCCTCGACC	1012, 2152	10
7547	198	GCGGCCGCGC	1076, 3649	10
7548	198	GCTGGACGGC	565, 2608	10
7549	198	GGATCCGTCG	1522, 4636	10
7550	198	GGGATGTCGA	3635, 4022	10
7551	198	GTCCGCCGCC	2322, 3787	10
7552	198	GTCGGTGCGG	3859, 4276	10
7553	198	GTCGGTGTAG	743, 2972	10
7554	198	GTGATCACCG	3176, 4625	10
7555	198	TCCACCAGCG	138, 2021	10
7556	198	TCGTCGTCGG	33, 1772	10
7557	198	TGCGGCCGAG	3924, 4174	10
7558	198	TTGGCCAGCT	1370, 2342	10
7559	199	CCGCCGCTGACCTGA	861, 886	15
7560	199	CCGCGGTCGCGGGC	184, 2252	14
7561	199	CTGATCGCCAGCG	1122, 1918	13
7562	199	ACGTCGTCGCGG	3938, 4589	12
7563	199	CCGACGTGGTCG	2579, 2684	12
7564	199	CGACCGAGGCGG	2183, 3176	12
7565	199	TCGGCGGCGCGG	46, 3698	12
7566	199	TGCTGATCGCCA	1495, 1916	12
7567	199	CAGCGACATCG	1878, 4104	11
7568	199	CCGCGACCGAG	3140, 3173	11
7569	199	CCGGGATCGTC	3893, 4241	11
7570	199	CGACCGCCGCT	858, 938	11
7571	199	CGGCCAGGTAG	296, 3159	11
7572	199	CGGCGGCCCGC	879, 904	11
7573	199	CGTCGCACGCC	2879, 4523	11
7574	199	GAACCCGGAGC	375, 2530	11
7575	199	GCCGGCCAGGT	2650, 3157	11
7576	199	GCTGCTGATCG	1914, 2217	11
7577	199	GGCCATGCCCG	420, 2979	11
7578	199	GGCGGCGGGCG	564, 3751	11
7579	199	GGCGTTGGCCA	162, 3666	11
7580	199	GGGCGTTGGCC	2414, 3665	11
7581	199	GGTCGTCGACG	1019, 4581	11
7582	199	AAGGTCGTGC	3019, 3832	10
7583	199	ACCAGCGACA	4102, 4552	10

7584	199	AGCTGCTGAT	1913, 2510	10
7585	199	CACCGGTGAG	332, 448	10
7586	199	CACGGTGACC	4044, 4230	10
7587	199	CCAAGGTCGT	3830, 4577	10
7588	199	CCGCCGGCGA	2108, 3124	10
7589	199	CCTCGGGCAA	1570, 3740	10
7590	199	CGCCGGCCTG	1271, 2781	10
7591	199	CGCCGGGCGG	2837, 4010	10
7592	199	CGCGATCCGG	84, 390	10
7593	199	CGCGCACCGA	1468, 2330	10
7594	199	CGGCGGCGGG	3750, 4749	10
7595	199	CGGGTCGCCG	1266, 4206	10
7596	199	GACCAGCGAC	594, 4551	10
7597	199	GCAGCAGCCG	3378, 3462	10
7598	199	GCCGCCGGCG	156, 3123	10
7599	199	GCCGGCGGCC	877, 3192	10
7600	199	GCGGACCCCG	177, 1942	10
7601	199	TCCCGGCCCA	268, 636	10
7602	199	TCGACGACGG	2965, 4818	10
7603	199	TTCGCGATCC	388, 4462	10
7604	200	GCACGATGTCGAAG	2247, 3511	14
7605	200	CGCGAGATCCTCG	230, 245	13
7606	200	ACCCGGCCGGCG	2766, 3988	12
7607	200	CGTCGGCGGCGG	1679, 4759	12
7608	200	GTGCGTTCGGTG	4037, 4865	12
7609	200	CACCGTGTTCG	1073, 1263	11
7610	200	CCAGGGCGGCC	2611, 2626	11
7611	200	CTTCTTGCCGG	2113, 3131	11
7612	200	GACGAGGTCGA	116, 1452	11
7613	200	GACTTCTCGCT	2009, 2045	11
7614	200	GCGCAACGCGG	1912, 2960	11
7615	200	GCGCAGCGTCG	1761, 4204	11
7616	200	GCTCGACTTCT	2005, 2107	11
7617	200	GGCGGCCGAAG	3212, 4852	11
7618	200	GGTCGCCGCCG	106, 887	11
7619	200	TCGCGGCTGTC	2126, 3435	11
7620	200	TGCTGCCGGTG	3166, 4302	11
7621	200	ACGAGGACCA	33, 1345	10
7622	200	ACGCGCCGTC	1466, 3682	10
7623	200	AGCTCGGCGA	931, 4884	10
7624	200	ATCGCGGAGG	260, 3807	10
7625	200	CATCCCCGGC	1439, 4607	10
7626	200	CCCGGAGGCC	217, 1130	10
7627	200	CCGCCTCGGC	1529, 2902	10
7628	200	CCGGTTTCGA	497, 4167	10
7629	200	CGACGCCGGT	304, 4726	10
7630	200	CGACGCGGTC	639, 4408	10
7631	200	CGAGGACGCG	3452, 3677	10
7632	200	CGCCGAGGCG	3206, 4111	10
7633	200	CGCCGCGGCG	1062, 3698	10
7634	200	CGCGGTGCGC	4198, 4528	10
7635	200	CGGACGTCGG	1558, 1675	10
7636	200	CGGGACGTCG	2444, 4441	10
7637	200	GACGGCGTCG	1356, 3264	10
7638	200	GCCCAGCCGC	2564, 4342	10
7639	200	GCCCGCCGCG	1059, 1797	10

7640	200	GCCGAGGTCA	4736, 4814	10
7641	200	GCCGGCCAGC	1593, 1776	10
7642	200	GCCGGGCCGG	1588, 2693	10
7643	200	GTCGGCGGTG	2735, 4841	10
7644	200	TGCCCGACGA	3475, 3616	10
7645	200	TGGTGGCCGC	1968, 3199	10

Supplementary Table S3. 51 Selected sequence (16-92 bp) from 7,645 repeat sequences

No.	Sequences	Number of the range having blast bit score[a] more than 32.2(16) in NCBI with *M. intracellulare* ATCC 13950[b]	Length (bp)
1	AGGCCGGAGACGTCGA	2	16
2	GCGGCGCCGGCCGGGCC	3	17
3	TTCTTGGCGGGCGCCTT	3	17
4	CAGCAGCACCACCAGCG	2	17
5	GGCGGGCCGTATTGAGG	2	17
6	CGCTTGGTGCCGAACCG	2	17
7	CGCGGCGGCAAGGGCGTC	3	18
8	AGCACCTTGACCGCGACG	2	18
9	GAAACGCAGGTTGCGCCA	2	18
10	GTTGATCAGGAAGATCCA	2	18
11	GAGTCGGTGATCTCGAAC	2	18
12	TGCCCCTGGTCGGGGTAGCC	2	20
13	GGCACCCGCGGCGCCCGGCA	6	20
14	AAACGTCGGCGGAGCCGACC	2	20
15	AAGCCCGAGCCGAAGCCCGA	4	20
16	GCCGCGCTTGCGATCGCCAC	65	20
17	CCCGCCGCAGGGCGGGCAGA	2	20
18	GGCCAGAACGGCCCGCCGGC	4	20
19	TCGGCCTGGTCGACGAGCTC	5	20
20	GCGATGTACGGCTACGCCGG	10	20
21	CCGGTTCCTACCCGATCGTG	2	20
22	GCCTTCTTGGCGGCGGTCTT	6	20
23	TTCCTGCAAAGCACCATCGG	2	20
24	CCGGCTTGACGTCGCGGTGGA	6	21
25	CGGCAGCAGCATGCCGCCGCC	2	21
26	CGACGCTGGGCGCGGAGGGTG	4	21
27	TTTCGCGCAACAAGTCGACGTT	9	22
28	TGGCGACCTACGAGATCGTGTG	2	22
29	GTACGCCCTCAACCCCGGCCGAA	3	23
30	GGAGCCGGGCGCAGCGGGTCGCC	93	23
31	GGGCGCGGCGGGTCGCCACCATCG	48	24
32	TCGATGAAGGTGCGTTGCGCGACG	2	24

33	TCTTGACCCCATACACCGAGTAATT	5	25
34	CGATTGAACTGATTGGTCAGGATCA	2	25
35	CAGCGCATCTGGACCAAGGACCACCCG	2	27
36	AATGGTCCGCCGGCGCAGGGTGGCCAG	6	27
37	CGCGCTTCGCGCGCGCTTACCTCGCTCGG	2	29
38	CGCAGGGTGGCCAGAACGGTCCGCCGGCGCA	7	31
39	CGGGCTAGTGGCGATCGCAAGCGCGGCGAAGC	34	32
40	CCCACCCCCTACCGCCGAGCGTGGCCCACCCC	6	32
41	ACCCGCTGCGCCCGGCTACGGCCTATAGCGGCG	49	33
42	GCGAGCCGGGGTCTCGTGTGGCGCAGGGCGATG CCGCGGAG	3	41
43	AGTGGCGCCTGATGCGTGCTACGACGATGCAGA GCGAAGCGATGAGGAGG	49	50
44	CCGATGGGTGCCGACCCGCTTCGCCCGGCTCCG CCGCGCTCGCGATCGACACCGGA	58	56
45	ACGGCGAGCGCCGCATGGCGCGAGTGAGGAGC CGGGCAATCGGGCCTAGCCCGGCG	18	56
46	ACCGCCGCTTGCCTGATGGCGGCGGCCCGCTGC GCCCGGCTTGCGCCGCGCTTGCG	81	56
47	ACACCGGACCCGATGGGCGCCGACCCGCTTCGC CCGGCTCCGCCGCGCTCGCGATCG	55	57
48	AGCTGGCGATCGCCGCGGAGCAGATGGTGGCG ACCCGCTGCGCCCCGGCGCTGCCGG	88	57
49	ACCCTAGCGTGGCGACGGTGAGCGCCGGAAGC GGCGCGAGCAAGGAGCGCGCAATCG	13	57
50	CCACTAGGTATCGATGGTGGCGACCCGCTTCGC CCGGCTCCGCCGCGCTCGCGATCGCCACT	95	62
51	CGCTCCTCCCCATCGCTGCGCTCTGGATCGTCGC CGGCGGGGGTCATGTGCGCCGCTCCTCCCCATC GCTGCGCTCTGGATCGTCGCCGGCG	54	92

[a]Blast bit score (S') : S' = (λ × S − lnK)/ ln2 where λ is the Gumble distribution constant, S is the raw alignment score, and K is a constant associated with the scoring matrix.
Normalized score considering optional alignment and gap panalty. For generating more than 16 bp-scaled primer, 32.2 bit were selected as a cutoff.
[b]*M. intracellulare* ATCC 13950: accession No. CP003322

Supplementary Table S4. Sequences, length, primer, and coding region of candidates repeats for generating primers

Candidates sequences for primers	Length (bp)	Primer	Coding region in ATCC 13950
AGTGGCGCCTGATGCGTGCTACGACGA TGCAGAGCGAAGCGATGAGGAGG	50	Primer 3	PbpA protein (https://www.ncbi.nlm.nih.gov/nuccore/CP003322.1?from=21282&to=22760)
CCACTAGGTATCGATGGTGGCGACCCG CTTCGCCCGGCTCCGCCGCGCTCGCGA TCGCCACT	62	Primer 4 and 5	rfbE (https://www.ncbi.nlm.nih.gov/nucleotide/CP003322.1?report=genbank&log$=nuclalign&blast_rank=12&RID=Z9X5FVW9013&from=256879&to=256940)
GCCGCGCTTGCGATCGCCAC	20	Primer 2	-Hypothetical protein, ABC transporter efflux protein, DrrB family protein, lipoprotein LpqB etc. (https://blast.ncbi.nlm.nih.gov/Blast.cgi#alnHdr_378797084)<br>-In this study, it is referred to as 'Mycobacteria specific sequences': characteristics shared in *M. tuberculosis, M. kansasii, M. ostraviense, M. avium, M. malmoense, M. ulcerans, M. shottsii* (https://blast.ncbi.nlm.nih.gov/Blast.cgi#alnHdr_2181124751)
AGCTGGCGATCGCCGCGGAGCAGATG GTGGCGACCCGCTGCGCCCCGGCGCTG CCGG	57	Primer 1	Hypothetical protein (https://www.ncbi.nlm.nih.gov/nucleotide/CP003322.1?report=genbank&log$=nuclalign&blast_rank=39&RID=Z9YRFEU0013&from=458333&to=458389)

Supplementary Table S5. The Locus[a], Identity[b], and Strand[c] of designed primers aligned with NCBI genome sequences of *M. intracellulare* ATCC 13950[d]

Primer 1 (31 loci)			Primer 2 (63 loci)			Primer 3 (45 loci)			Primer 4 (53 loci)			Primer 5 (34 loci)		
Locus	Identity	Strand	Locus	Identity	Strand	Locus	Identity	Strand	Locus	Identity	Strand	Locus	Identity	Strand
291865	18/20	+/-	256749	18/20	+/+	214353	23/23	+/+	256748	21/22	+/+	256726	24/24	+/+
291899	19/20	+/-	256806	19/20	+/+	214403	23/23	+/+	256805	22/22	+/+	256783	24/24	+/+
307314	19/20	+/+	256863	19/20	+/+	214453	23/23	+/+	256862	22/22	+/+	256840	24/24	+/+
458355	20/20	+/+	256920	19/20	+/+	502064	22/23	+/+	256919	22/22	+/+	256897	24/24	+/+
458412	20/20	+/+	281372	19/20	+/+	507553	21/23	+/-	281371	19/21	+/+	291861	23/24	+/-
918038	18/20	+/+	307409	18/18	+/+	602131	23/23	+/+	344055	20/21	+/+	291893	22/23	+/-
918094	18/20	+/+	307430	18/18	+/+	656635	21/23	+/-	344113	19/20	+/+	344035	21/22	+/+
918150	18/20	+/+	344056	20/20	+/+	656688	21/23	+/-	444323	21/22	+/-	356033	22/22	+/+
956200	18/20	+/+	344113	20/20	+/+	656741	21/23	+/-	444378	21/22	+/-	356090	22/22	+/+
982135	18/20	+/+	444322	20/20	+/-	656794	21/23	+/-	866038	21/22	+/-	356147	22/22	+/+
982322	18/20	+/+	444377	20/20	+/-	773287	22/23	+/-	982163	19/21	+/+	356204	22/22	+/+
1110617	19/20	+/+	449077	18/18	+/-	908996	22/23	+/+	982273	21/21	+/+	947846	21/21	+/+
1110672	19/20	+/+	866037	18/20	+/-	926688	23/23	+/-	982383	21/21	+/+	982251	21/21	+/+
1110727	19/20	+/+	871124	18/18	+/+	1046030	22/23	+/+	986881	19/21	+/-	982361	21/21	+/+
1261142	20/20	+/+	982164	19/20	+/+	1052759	22/23	+/-	1110645	20/22	+/+	1261148	23/24	+/+
1261199	20/20	+/+	982274	19/20	+/+	1525437	23/23	+/-	1110700	20/22	+/+	1261205	23/24	+/+
1261255	20/20	+/+	982384	19/20	+/+	1600606	23/23	+/-	1110755	20/22	+/+	1261261	23/24	+/+
1786101	19/20	+/+	986880	19/20	+/-	1624100	21/23	+/+	1261170	21/22	+/+	1799231	22/24	+/-

2385671	19/20	+/-	1110646	18/20	+/+	1625004	22/23	+/+	1261227	21/22	+/+	1799286	22/24	+/-
3086640	19/20	+/-	1110701	18/20	+/+	1701263	23/24	+/+	1261283	21/22	+/+	3001191	21/21	+/-
3086694	19/20	+/-	1110756	18/20	+/+	1701316	23/24	+/+	1799209	21/22	+/-	3404520	23/24	+/-
3086748	19/20	+/-	1261171	20/20	+/+	1701369	23/24	+/+	1799264	20/21	+/-	3914223	21/21	+/-
3262857	19/20	+/-	1261228	20/20	+/+	1701422	23/24	+/+	1889236	21/22	+/-	3914280	21/21	+/-
3397907	20/20	+/-	1261284	20/20	+/+	1715807	20/23	+/-	2513049	21/22	+/-	3975532	24/24	+/+
3483417	19/20	+/-	1282507	18/18	+/+	1715860	20/23	+/-	2513104	21/22	+/-	3975586	24/24	+/+
3786884	20/20	+/-	1282563	18/18	+/+	1750934	23/23	+/-	2746798	19/19	+/+	4154017	22/24	+/+
4390882	20/20	+/+	1282578	18/18	+/+	1750984	23/23	+/-	3001169	19/19	+/-	4154072	22/24	+/+
4804948	19/20	+/-	1282633	18/18	+/+	1751034	23/23	+/-	3086613	21/22	+/-	4154127	22/24	+/+
4804980	19/20	+/-	1799208	20/20	+/-	1870612	22/23	+/+	3086667	21/22	+/-	4292317	24/24	+/-
4838565	19/20	+/-	1799263	20/20	+/-	3066961	20/22	+/-	3086721	21/22	+/-	4374801	23/24	+/+
5241537	19/20	+/+	1889235	20/20	+/-	3125792	22/23	+/-	3231199	20/21	+/-	4738570	23/24	+/+
	-		2513048	20/20	+/-	3180951	23/23	+/+	3258678	20/21	+/+	4789371	21/22	+/+
	-		2513103	20/20	+/-	3461914	21/23	+/-	3451024	19/19	+/+	4789425	21/22	+/+
	-		3086612	20/20	+/-	3715009	23/23	+/-	3786856	20/21	+/-	5176293	21/21	+/-
	-		3086666	20/20	+/-	3763190	22/23	+/-	3836131	20/21	+/-		-	
	-		3086720	20/20	+/-	3763243	22/23	+/-	3888301	20/21	+/+		-	
	-		3231199	19/20	+/-	3763296	22/23	+/-	3914204	21/22	+/-		-	
	-		3258679	20/20	+/+	3865783	23/23	+/+	3914261	21/22	+/-		-	
	-		3397864	18/18	+/-	3865884	22/23	+/+	3975554	21/21	+/+		-	
	-		3409624	18/18	+/+	4113481	23/23	+/+	3975608	20/20	+/+		-	

-	3483388	18/18	+/-	4550151	21/23	+/-	4154039	20/21	+/+	-
-	3513594	18/18	+/+	4550204	21/23	+/-	4154094	20/21	+/+	-
-	3715864	18/18	+/-	4786682	23/23	+/-	4154149	21/22	+/+	-
-	3786855	20/20	+/-	5150908	21/22	+/-	4232306	20/21	+/-	-
-	3836130	20/20	+/-	5150961	21/22	+/-	4280351	21/22	+/+	-
-	3888301	18/20	+/+		-		4292295	19/21	+/-	-
-	3914203	18/20	+/-		-		4374823	21/22	+/+	-
-	3914260	18/20	+/-		-		4390910	20/21	+/+	-
-	3975555	19/20	+/+		-		4738592	21/22	+/+	-
-	3975609	18/20	+/+		-		4838536	19/20	+/-	-
-	4154040	20/20	+/+		-		5058039	18/18	+/+	-
-	4154095	20/20	+/+		-		5288336	18/18	+/+	-
-	4154150	20/20	+/+		-		5349832	19/19	+/-	-
-	4232306	19/20	+/-		-			-		-
-	4232326	19/20	+/-		-			-		-
-	4280352	20/20	+/+		-			-		-
-	4292294	19/20	+/-		-			-		-
-	4374824	18/20	+/+		-			-		-
-	4390911	20/20	+/+		-			-		-
-	4738593	19/20	+/+		-			-		-
-	4838513	18/18	+/-		-			-		-

-	4838536	20/20	+/-	-	-	-	
-	5241567	18/18	+/+	-	-	-	

[a]Locus: the genome location in NCBI information in which the primer aligned

[b]Identity: matching ratio (designed primer / genome sequences)

[c]Strand: +/+ is forward direction in the genome, +/- is reverse direction in the genome

[d]*M. intracellulare* ATCC 13950: https://www.ncbi.nlm.nih.gov/nuccore/NC_016946.1

Supplementary Table S6. *In silico* PCR amplicons using various mycobacteria species

Mycobacteria species	Length of *in silico* PCR amplicons (bp)									
	primer 1 and 2	Primer 1 and 3	Primer 1 and 4	Primer 1 and 5	Primer 2 and 3	Primer 2 and 4	Primer 2 and 5	Primer 3 and 4	Primer 3 and 5	Primer 4 and 5
Mycobacterium africanum GM041182	7984	-	7986	8036	7984	7986	8007	7986	-	8008
Mycobacterium avium 104	-	-	-	-	8808	-	-	-	-	-
Mycobacterium avium subsp. *paratuberculosis* MAP4	-	-	-	-	8809	-	-	8810	-	-
Mycobacterium avium subsp. *paratuberculosis* str. k10	-	-	-	-	8809	-	-	8810	-	-
Mycobacterium bovis BCG str. Korea 1168P	7984	-	7986	8036	7984	7986	8007	7986	-	8008
Mycobacterium bovis BCG str. Mexico	7984	-	7986	8036	7984	7986	8007	7986	-	8008
Mycobacterium bovis BCG str. Pasteur 1173P2	7984	-	7986	8036	7984	7986	8007	7986	-	8008
Mycobacterium bovis BCG str. Tokyo 172	7984	-	7986	8036	7984	7986	8007	7986	-	8008
Mycobacterium bovis subsp. *bovis* AF2122/97	7984	-	7986	8036	7984	7986	8007	7986	-	8008
Mycobacterium canettii CIPT 140010059	7984	-	7986	-	7984	7986	8007	7986	-	8008
Mycobacterium canettii CIPT 140060008	7984	-	7986	-	7984	7986	8007	7986	-	8008
Mycobacterium canettii CIPT 140070008	7985	-	7987	8037	7985	7987	8008	7987	-	8009
Mycobacterium canettii CIPT 140070010	8040	8042	8042	8042	8040	8042	8040	8042	-	8042
Mycobacterium canettii CIPT 140070017	7984	-	7986	8036	7984	7986	8007	7986	-	8008
Mycobacterium indicus pranii MTCC 9506	4118, 4663	4665, 5490, 6729, 8986	4119, 4693	4659	4663, 5490, 6729, 9012	4664	4663	5490, 6729	4653, 5490, 6729	4687

Mycobacterium intracellulare ATCC 13950	4179, 4746	5490, 6730, 8651	4180	-	4717, 5490, 6730, 8677	4717	4717	5490, 6730	5490, 6730	-
Mycobacterium intracellulare MOTT-02	4179, 6241	5490, 6730, 8538	4180	-	5490, 6212, 6730, 8564	6212	6212	5490, 6730	5490, 6730	-
Mycobacterium intracellulare MOTT-64	4663	5490, 6730, 8538	-	-	4663, 5490, 6730, 8508	4664	4663	5490, 6730	5490, 6730	-
Mycobacterium sp. JDM601	1496	-	-	1519	1467	1468	1490	-	1513	1513
Mycobacterium sp. MOTT36Y	-	5491	-	-	5491	-	-	5491	5491	-
Mycobacterium tuberculosis 7199-99	7984	-	7986	8036	7984	7986	8007	7986	-	8008
Mycobacterium tuberculosis CAS NITR204	7956	-	7958	8008	7956	7958	7979	7958	-	7980
Mycobacterium tuberculosis CCDC5079	7984	-	7986	8036	7984	7986	8007	7986	-	8008
Mycobacterium tuberculosis CCDC5079	7984	-	7986	8036	7984	7986	8007	7986	-	8008
Mycobacterium tuberculosis CCDC5180	7984	-	7986	8036	7984	7986	8007	7986	-	8008
Mycobacterium tuberculosis CDC1551	7983	-	7985	8035	7983	7985	8006	7985	-	8007
Mycobacterium tuberculosis CTRI-2	7984	-	7986	8036	7984	7986	8007	7986	-	8008
Mycobacterium tuberculosis EAI5	7984	-	7986	8036	7984	7986	8007	7986	-	8008
Mycobacterium tuberculosis EAI5 NITR206	7984	-	7986	8036	7984	7986	8007	7986	-	8008
Mycobacterium tuberculosis F11	7984	-	7986	8036	7984	7986	8007	7986	-	8008
Mycobacterium tuberculosis H37Ra	7984	-	7986	8036	7984	7986	8007	7986	-	8008
Mycobacterium tuberculosis H37Rv	7984	-	7986	8036	7984	7986	8007	7986	-	8008
Mycobacterium tuberculosis H37Rv	7984	-	7986	8036	7984	7986	8007	7986	-	8008
Mycobacterium tuberculosis KZN 1435	8375	-	8377	8371	8375	8377	8375	8377	-	8377
Mycobacterium tuberculosis KZN 4207	8375	-	8377	8371	8375	8377	8375	8377	-	8377

Mycobacterium tuberculosis KZN 605	8487	-	8489	8483	8487	8489	8487	8489	-	8489
Mycobacterium tuberculosis RGTB327	7992	-	7994	8044	7992	7994	8015	7994	-	8016
Mycobacterium tuberculosis RGTB423	7993	-	7995	8086	7993	7995	8016	7995	-	8017
Mycobacterium tuberculosis UT205	7984	-	7986	8036	7984	7986	8007	7986	-	8008
Mycobacterium tuberculosis str. Beijing NITR203	7984	-	7986	8036	7984	7986	8007	7986	-	8008
Mycobacterium tuberculosis str. Erdman = ATCC 35801 DNA	7984	-	7986	8036	7984	7986	8007	7986	-	8008
Mycobacterium tuberculosis str. Haarlem	7984	-	7986	8036	7984	7986	8007	7986	-	8008
Mycobacterium tuberculosis str. Haarlem NITR202	7993	-	7995	8045	7993	7995	8016	7995	-	8017
Mycobacterium yongonense 05-1390	-	5490	-	-	5490	-	-	5490	5490	-

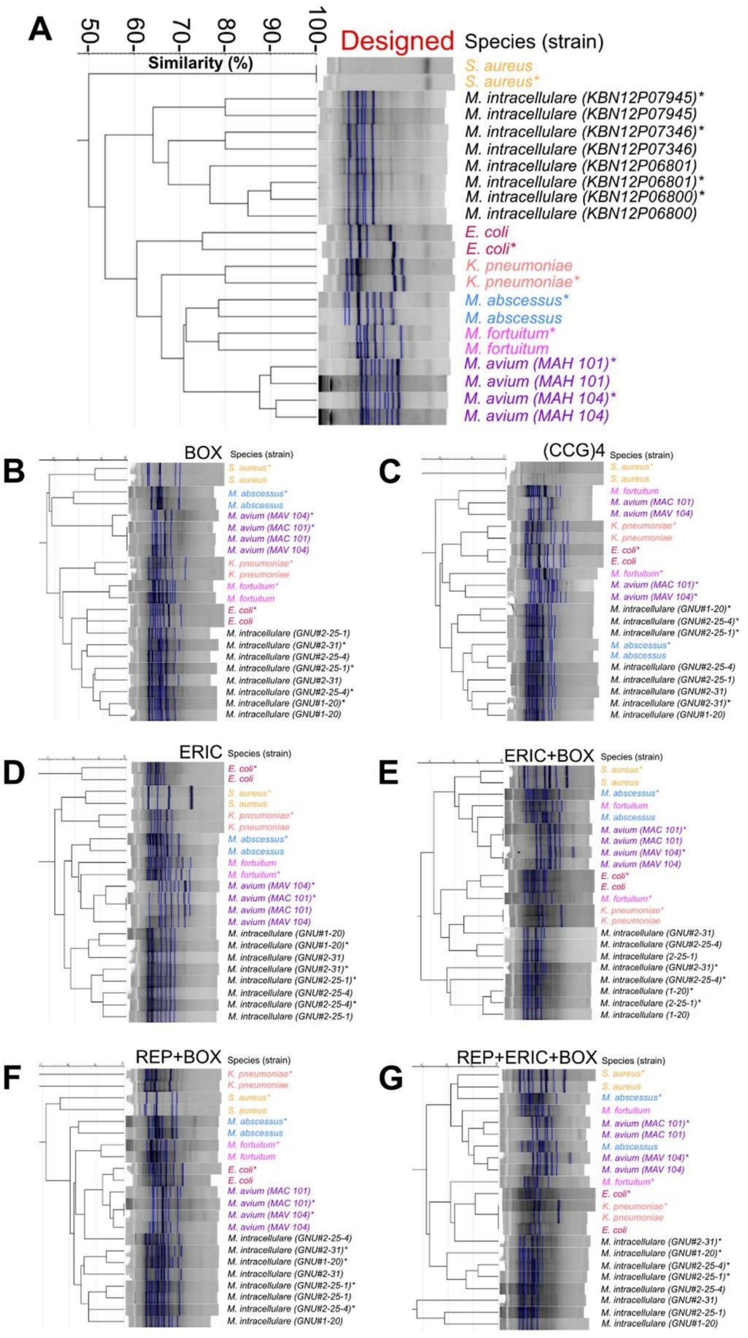

Supplementary Figure S1. Comparison of reproducibility between novel and previously published rep -PCRs. The reproducibility tests for novel and previous published rep-PCRs. (A - G) The results of rep-PCR using the first DNA template (no asterisk) and second DNA template (with an asterisk, DNA extracted after a subculture of the strains). 4 *M. intracellulare* strains, *S. aureus*, *E. coli*, *K. pneumoniae*, *M. abscessus*, *M. fortuitum*, and 2 *M. avium* strains were used. (A) In the case of designed rep-PCR, all the patterns were reproduced. (B) In the case of BOX -PCR, patterns of MAH 104 and *M . fortuitum* were not reproduced. (C) In the case of (CCG)4-PCR, patterns of *M. fortuitum* and 2 *M. avium* strains were not reproduced. (D) In the case of ERIC – PCR, the patterns of MAH 104 were not reproduced. (E) In the case of ERIC + BOX -PCR, the patterns of *M. abscessus*, *M. fortuitum*, and MAH 104 were not reproduced. (F) In the case of REP + BOX -PCR, the patterns of MAH 104 and *M. intracellulare* (KBN12P07346) were not reproduced. (G) In case of REP + ERIC + BOX-PCR, the patterns of *M. abscessus*, *M. fortuitum*, MAH 104, and *E. coli* were not reproduced. %Similarity: Dice.

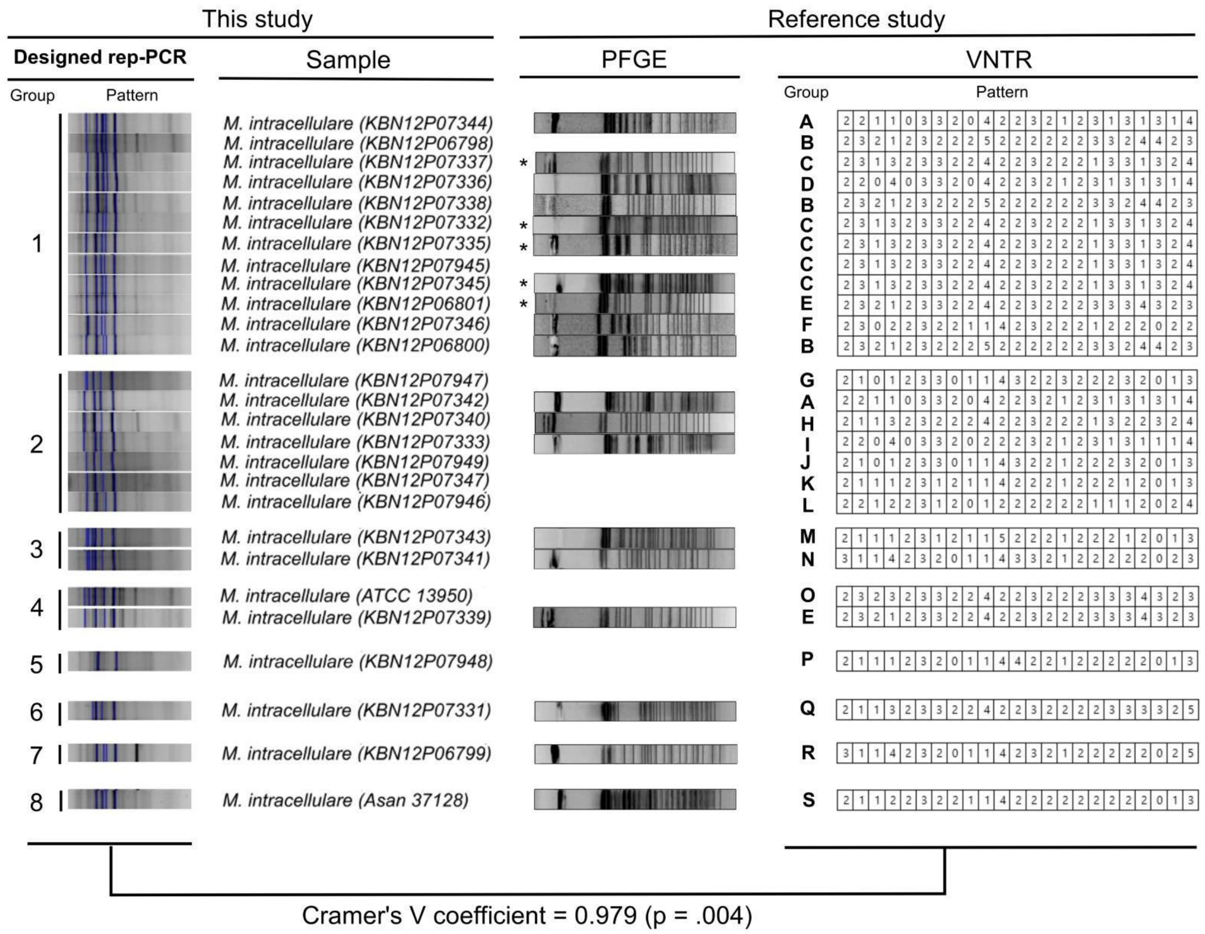

Supplementary Figure S2. Analysis of correlation between the designed rep-PCR and other molecular epidemiological (PFGE and VNTR) techniques using 27 *M. intracellulare* strains. In previous studies , the VNTR experime nt was performed on 27 strains and PFGE was performed on 19 strains. 27 *M. intracellulare* produced 8 groups of bands patterns. Group 1 had almost the same pattern of PFGE in 5 strains (with an asterisk). Group 1 also has B (n = 3) and C (n = 5) types of VNTR. The VNTR type was determined according to the VNTR numerical patterns, the patterns mean copy number of VNTR 1 to 16 and VNTR -MIRU 3, 18 , 19 , 20 , 22 , 31 , 33 . There was a significant correlation between the groups of designed rep-PCR and VNTR, showing Cramer's V coefficient = 0.979 (p = .004). It can suggest the rep-PCR can reflect the epidemiological meaning. KBN12P06800 in group 1 and KBN12P06801 in group 1 were originated from a patient A; KBN12P07345 in group 1 and KBN12P07347 in group 2 were originated from a patient B; KBN 12P06798 in group 1 and KBN 12P06799 in group 7 were originated from a patient C. This method can be used to monitor whether redeveloped MAC -PD caused by relapse or re-infection.

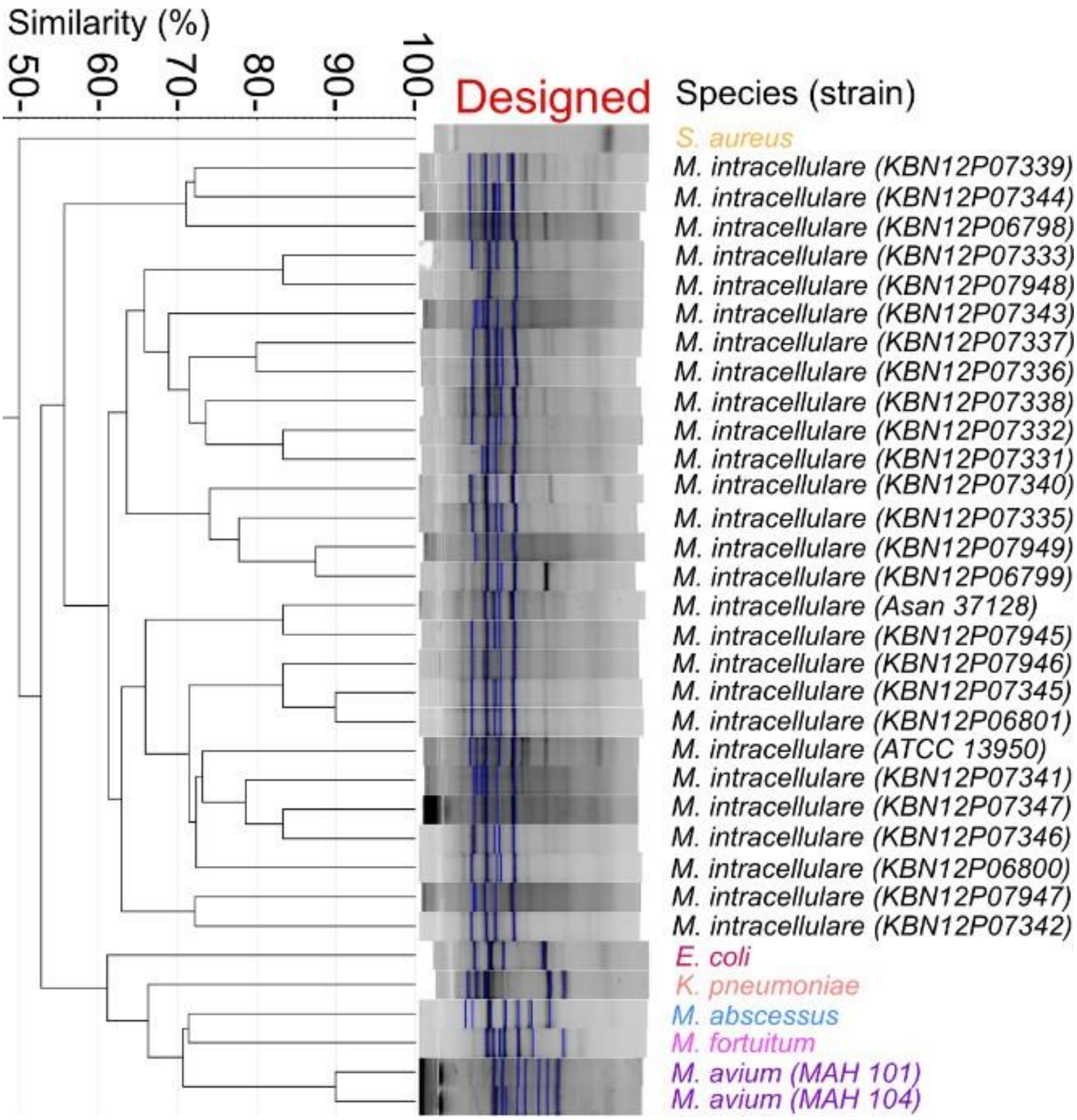

Supplementary Figure S3. Applications of designed rep-PCR on *M. intracellulare, S. aureus, E. coli, K. pneumoniae, M. fortuitum, M. abscessus*, and *M. avium*. 27 *M. intracellulare* strains showed bands of various patterns with a similarity of 56–90% on the dendrogram. In addition, despite the band variation, the band patterns between *M. intracellulare* and the other six species could be distinguished by two clusters with 50% similarity. Designed rep-PCR using *E. coli, K. pneumoniae, M. abscessus, M. fortuitum,* and *M. avium* showed different band patterns, whereas *S. aureus* showed no bands. %Similarity: Dice.

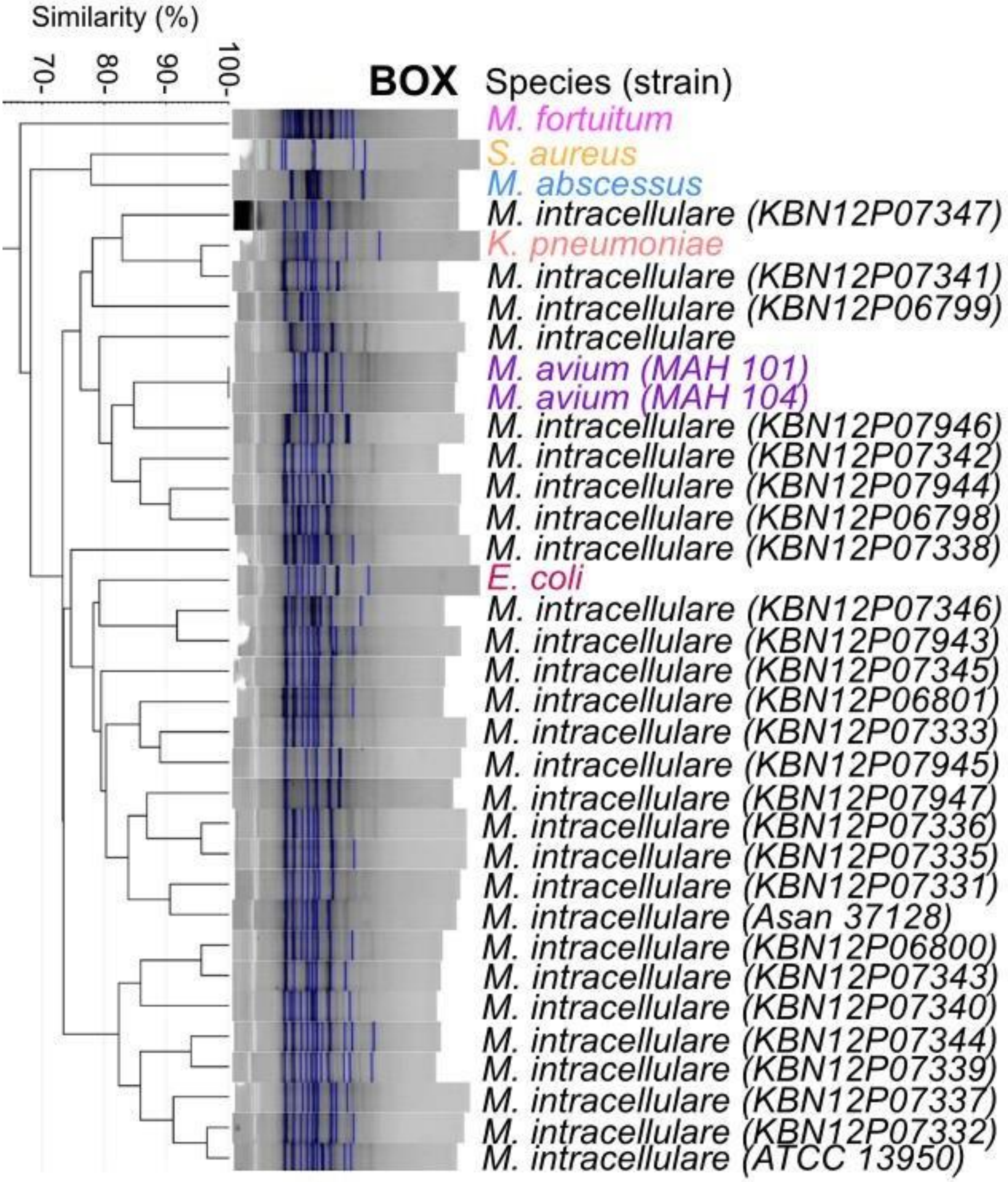

Supplementary Figure S4. Applications of previous published rep-PCR (BOX-PCR) on *M. intracellulare, S . aureus, E. coli, K. pneumoniae, M. fortuitum, M. abscessus*, and *M. avium.* BOX-A1R primer was used. unlike the designed rep-PCR, *M. intracellulare*, *K. pneumoniae, M. avium,* and *E. coli* were included in the same cluster on the dendrogram and showed a high similarity of 74–96% between species. *M. fortuitum*, *S. aureus*, and *M. abscessus* were included in different clusters, with approximately 67% similarity to *M. intracellulare.* %Similarity: Dice.

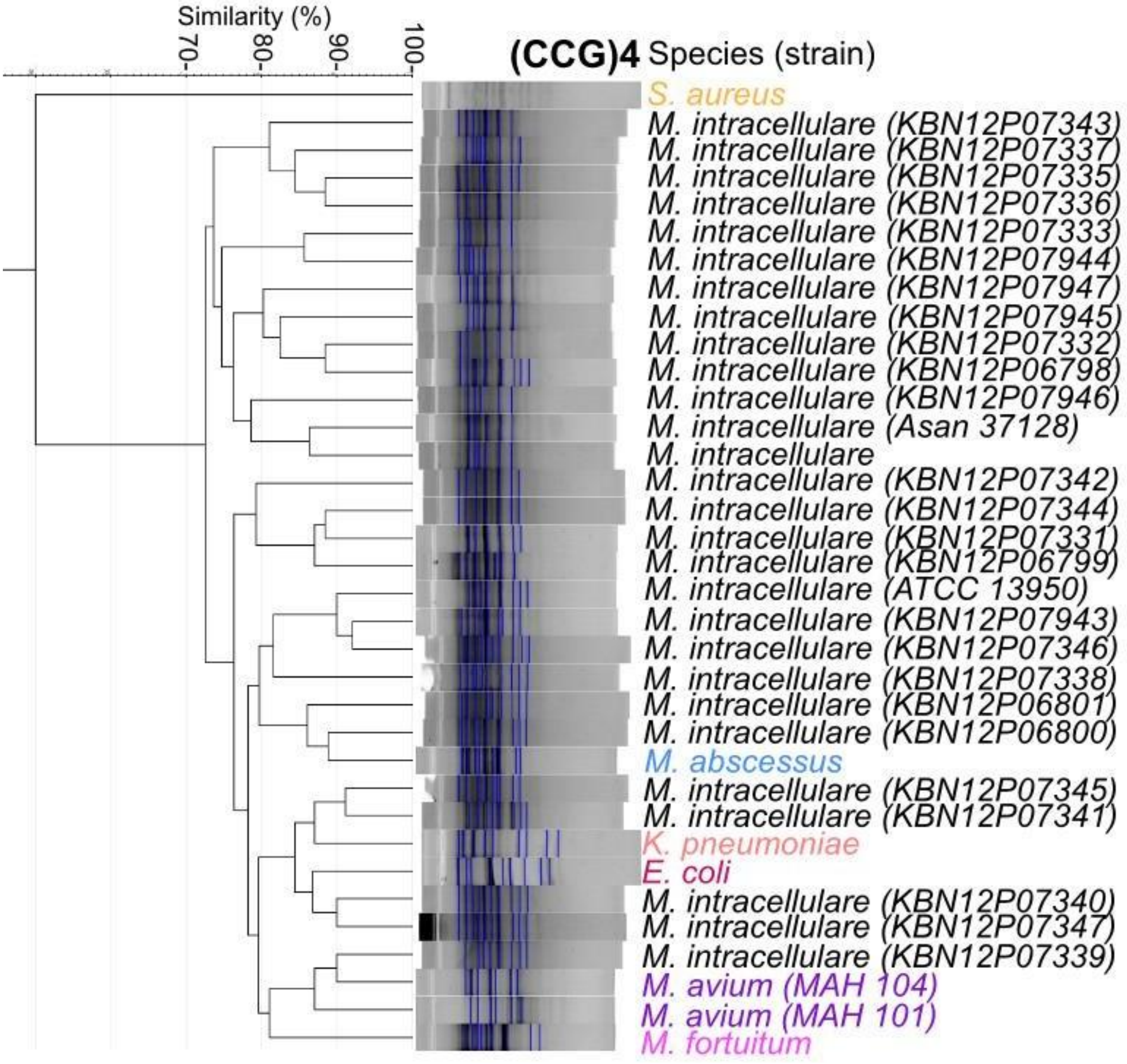

Supplementary Figure S5. Applications of previous published rep -PCR ((CCG)4-PCR) on *M. intracellulare, S. aureus, E. coli, K. pneumoniae, M. fortuitum, M. abscessus*, and *M. avium.* (CCG)4 primer was used. It was difficult to distinguish all the species on the dendrogram because they all were included in one cluster (78–87% similarity between species). Additionally, *S. aureus* did not show a definite PCR band. %Similarity: Dice.

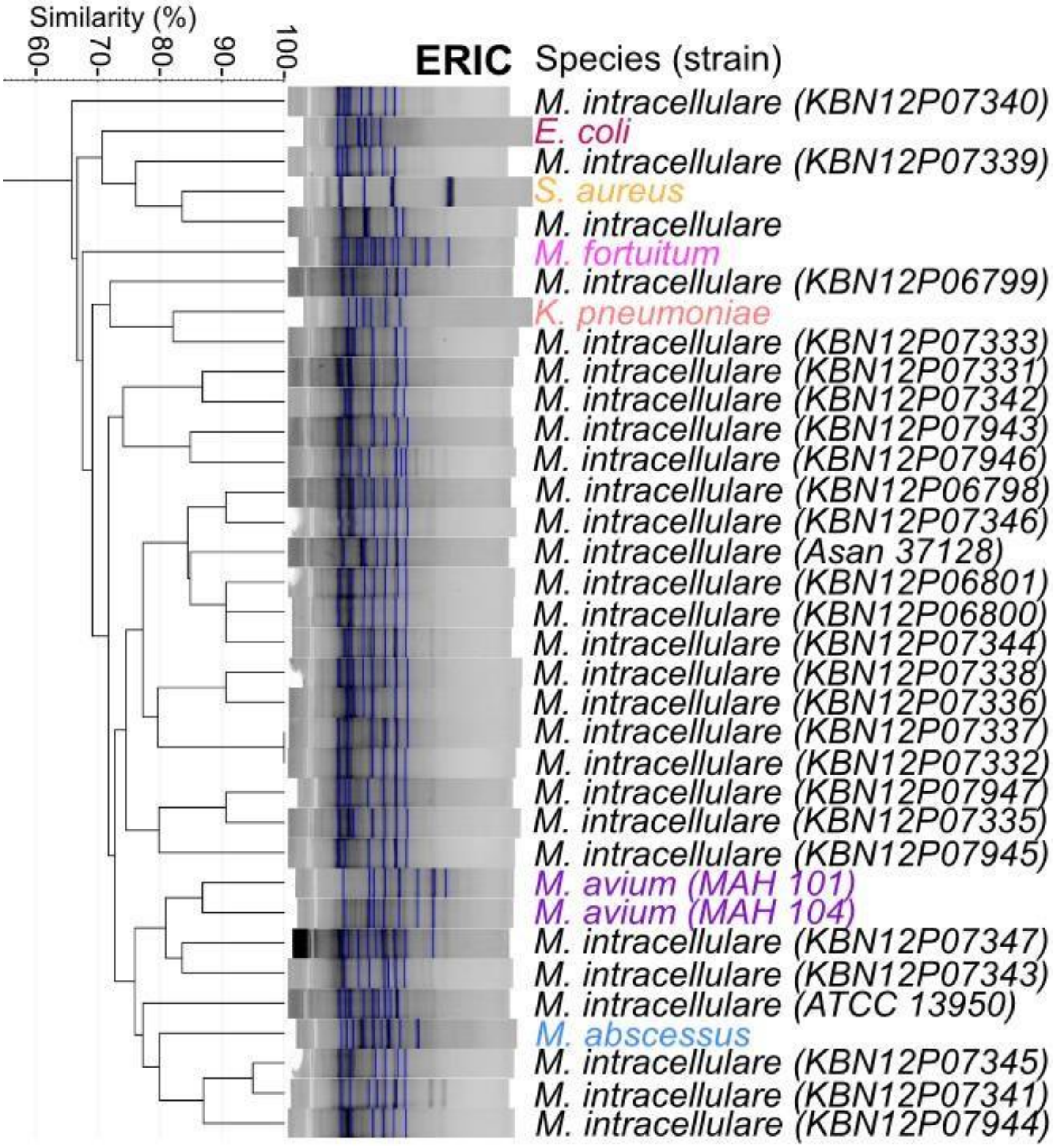

Supplementary Figure S6. Applications of previous published rep-PCR (ERIC-PCR) on *M. intracellulare, S. aureus, E. coli, K. pneumoniae, M. fortuitum, M. abscessus,* and *M. avium.* ERIC 1R and ERIC 2 primers were used. It was difficult to distinguish all the species on the dendrogram . This is because the similarity between *M. intracellulare* and the other six species was 66–84%, which was the same as the minimum similarity between *M. intracellulare* strains. %Similarity: Dice.

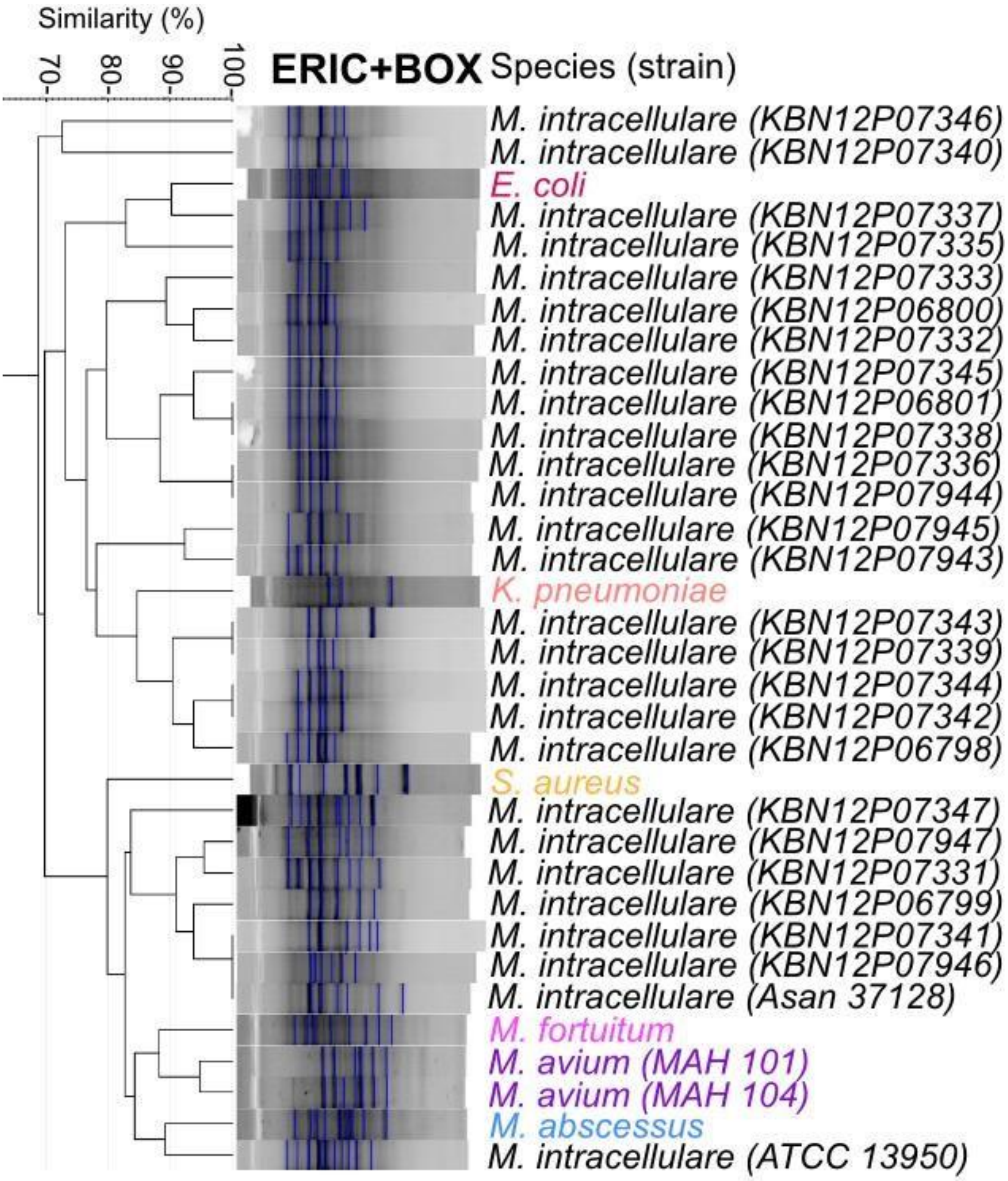

Supplementary Figure S7. Applications of previous published rep -PCR (ERIC + BOX –PCR) on *M . intracellulare* , *S. aureus* , *E. coli* , *K. pneumoniae* , *M . fortuitum* , *M . abscessus* , and *M . avium.* BOX A1R, ERIC 1R, and ERIC 2 primers were used showing a messy background. Since *M. intracellulare* showed 69–100% similarity between strains within the species, and the maximum similarity between *M. intracellulare* and other species was 90%, it was difficult to distinguish between species (e.g., *M. intracellulare* and *E. coli*). %Similarity: Dice.

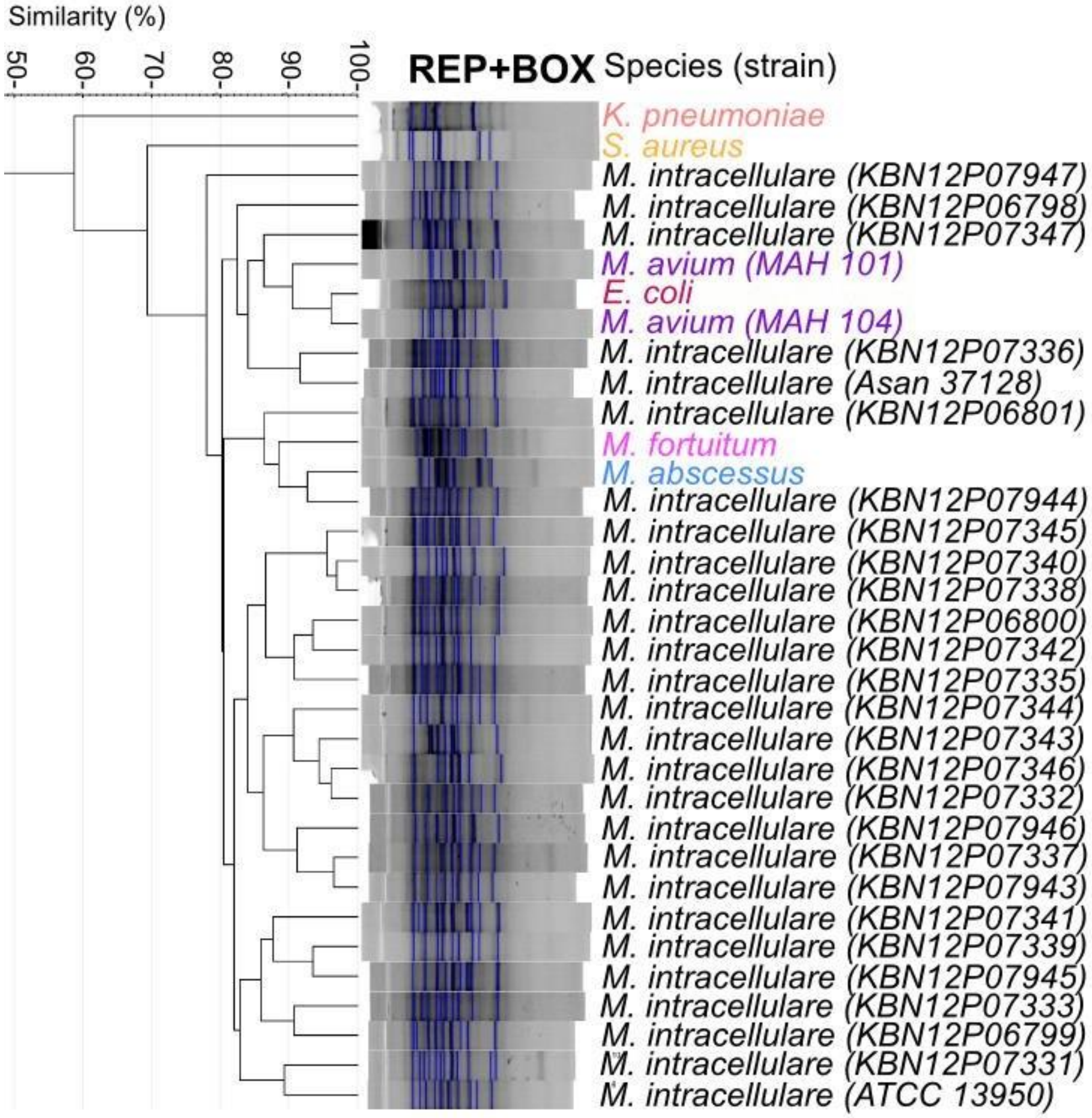

Supplementary Figure S8. Applications of previous published rep -PCR (REP + BOX – PCR) on *M . intracellulare* , *S. aureus* , *E. coli* , *K. pneumoniae* , *M . fortuitum* , *M . abscessus* , and *M . avium.* BOX A1R, REP 1RI, and REP 2I primers were used. *M. intracellulare* intraspecies similarity was 78–97%, and the similarity between *M. intracellulare* and other species was 59–87%, making it difficult to distinguish between species. %Similarity: Dice.

frontiers

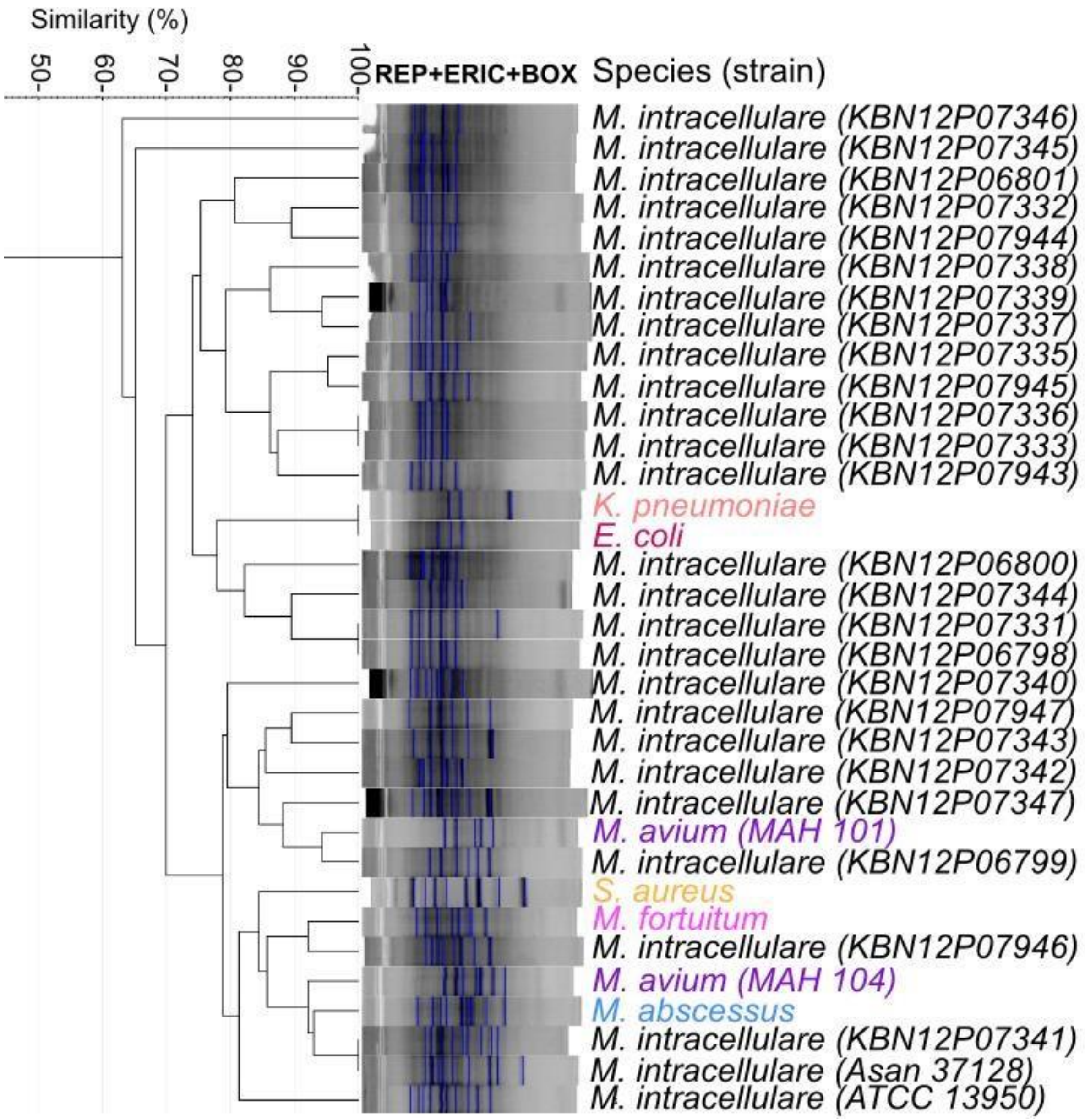

Supplementary Figure S9. Applications of previous published rep -PCR (REP + ERIC + BOX –PCR) on *M. intracellulare* , *S. aureus* , *E. coli* , *K. pneumoniae* , *M . fortuitum* , *M . abscessus* , and *M . avium.* BOX A1R, ERIC 1R, ERIC 2, REP 1RI, and REP 2I primers were used with a messy background . It was difficult to distinguish species because the similarity between *M. intracellulare* strains was 63–100%, and that between *M. intracellulare* and other species was 63–77%. %Similarity: Dice.
